# Deciphering the mosaic genome of sugarcane cultivars through polyploid admixture inference with AdmixPoly

**DOI:** 10.1186/s13059-026-04162-3

**Published:** 2026-06-19

**Authors:** Simon Rio, Franck Gauthier, Olivier Garsmeur, George Piperidis, Jean-Yves Hoarau, German Serino, Raul Castillo Torres, Shailesh Vinay Joshi, Yoshifumi Terajima, Jershon Lopez-Gerena, María Francisca Perera, Andrew Stoute, Goolam Badaloo, Dongliang Huang, Kerrie Barry, Jeremy Schmutz, Tristan Mary-Huard, Angélique D’Hont

**Affiliations:** 1 CIRAD, UMR AGAP Institut, Montpellier, F-34398 France; 2 UMR AGAP Institut, Univ Montpellier, CIRAD, INRAE, Institut Agro, Montpellier, F-34398 France; 3 Université Paris-Saclay, INRAE, CNRS, AgroParisTech, UMR GQE-Le Moulon, Gif-sur-Yvette, 91190 France; 4 Sugar Research Australia, Brisbane, QLD 4000 Australia; 5 eRcane, Sainte-Clotilde, France; 6 Chacra Experimental Agrícola Santa Rosa, Camino Vecinal No. 8 Km 6, Colonia Santa Rosa, 4531 Argentina; 7 Centro de Investigación de la Caña de Azúcar del Ecuador - CINCAE, Guayaquil, Ecuador; 8 South African Sugarcane Research Institute, Private Bag X02, Mount Edgecombe, 4300 Durban, KwaZulu-Natal South Africa; 9 School of Agriculture and Science, College of Agriculture, Engineering and Science, University of KwaZulu-Natal, South Africa; 10 Tropical Agriculture Research Front, JIRCAS, 1091-1 Maezato-Kawarabaru, Ishigaki, 907-0002 Japan; 11 Centro de Investigación de la Caña de Azúcar de Colombia (CENICAÑA), Cali, Colombia; 12 Instituto de Tecnología Agroindustrial del Noroeste Argentino (ITANOA), Estación Experimental Agroindustrial Obispo Colombres (EEAOC) - Consejo Nacional de Investigaciones Científicas y Técnicas (CONICET), Las Talitas, Argentina; 13 West Indies Central Sugar Cane Breeding Station, Groves, St. George Barbados; 14 Mauritius Sugarcane Industry Research Institute, Mauritius Cane Industry Authority, 1 Moka Road, Réduit, Mauritius; 15 Sugarcane Research Institute, Guangxi Academy of Agricultural Sciences, Nanning, China; 16 DOE Joint Genome Institute, Lawrence Berkeley National Laboratory, Berkeley, 94720 CA USA; 17 HudsonAlpha Institute for Biotechnology, 601 Genome Way Northwest, Huntsville, 35806 AL USA; 18 Université Paris-Saclay, AgroParisTech, INRAE, UMR MIA-Paris, Palaiseau, 91120 France

**Keywords:** Population structure, Admixture, Polyploidy, *Saccharum* spp, Sugarcane, Crop evolution

## Abstract

**Background:**

Characterizing population structure and admixture events between ancestral groups plays a key role in understanding the evolutionary history of species and crops. Most tools for inferring admixture have been developed for diploids and are not suitable for polyploids, in particular those with high and mixed ploidy such as *Saccharum*.

**Results:**

Here we present AdmixPoly, an R-package designed to infer admixture in polyploid species both at the genome-wide scale and locally along chromosomes. We compare AdmixPoly with state-of-the-art methods using simulations, demonstrating its precision and computational efficiency. Notably, local admixture inference in complex scenarios, such as high ploidy levels, large numbers of ancestral groups and alleles per marker is enabled through efficient approximations of emission and transition probabilities within a hidden Markov model framework. We apply this approach to characterize the contributions of wild *Saccharum* species to the complex polyploid genome of modern sugarcane cultivars. A panel of wild and cultivated *Saccharum* accessions is genotyped for 80K genomic regions, each revealing approximately 50 read-scale haplotypes.

**Conclusions:**

The results reveal that most of the approximately 12 copies of each basic chromosome in modern cultivars are derived from the domesticated species *Saccharum officinarum*, with one to four copies typically contributed by distinct subgroups of the wild species *Saccharum spontaneum*. In addition, contributions from an unknown wild *Saccharum* group originating from the Pacific were identified in most cultivars. The conserved pattern of these introgressions suggests that they can be traced back to the early stages of sugarcane breeding approximately a century ago.

**Supplementary Information:**

The online version contains supplementary material available at https://doi.org/10.1186/s13059-026-04162-3.

## Background

Population structure and admixture play critical roles in shaping genetic diversity and evolutionary trajectories of species and crops. Population structure refers to the non-random distribution of genetic variation across populations, resulting from factors such as geographic isolation, genetic drift, and selection. Admixture, on the other hand, describes the interbreeding between previously isolated populations, leading to the exchange and mixing of genetic material. Studying population structure and admixture provides valuable insights into the evolutionary history of species, including the origins of populations or crops, patterns of gene flow, and the impact of historical events on genetic diversity. For instance, these processes have been pivotal in understanding the genetic makeup of humans [1], domestic animals like cattle [2] and dogs [3], or important crops such as rice [4] and banana [5].

Admixture inference methods can be divided into two categories: i) genome-wide approaches that aim to estimate overall ancestry proportions from different source populations (referred to here as genome-wide admixture), and ii) local approaches, which examine individual chromosomes to detect ancestry switches and identify genomic segments inherited from different ancestral populations (referred to here as local admixture). Based on a similar genetic model, several renowned tools have been proposed for inferring genome-wide admixture which notably differ in the inference strategy: Bayesian inference using either a Markov chain Monte Carlo (MCMC) algorithm for Structure [6], a variational approach for fastStructure [7], or frequentist maximum likelihood approaches for Frappe [8] and Admixture [9]. For local admixture inference, most methods rely on a hidden Markov model (HMM) to take into account the dependence between the origins of adjacent loci along the chromosomes, as first proposed in an updated implementation of Structure [10]. This modeling was further extended to take account of the linkage disequilibrium (LD) between markers in ancestral populations (background LD), e.g. Saber [11]. However, all these tools were developed for diploid species and are generally not suitable for polyploid species.

Polyploid organisms are characterized by genomes with more than two complete sets of chromosomes. Polyploidy is very common in wild plants and crops such as wheat, potato or sugarcane, and it can also be found in some animal species such as goldfish or salamanders. From version 2 of Structure [10], any polyploid population could be analyzed, including the case of a population of individuals with variable ploidy levels (i.e. mixed-ploidy), provided that specific coding of the data is respected [12]. Another tool proposed by Shastry et al. [13], Entropy, has been developed for mixed-ploidy populations with ploidy levels ranging from one to six. This last approach notably proposes taking allele read depths of bi-allelic markers as inputs to calculate genotype likelihoods, which can be beneficial in the context of low-depth sequencing data where allele dosages are difficult to estimate.

Regarding local admixture inference, the type of genotypic information available conditions the method that can be applied. If the genotypic data are phased along the genome, i.e. the genotype of each chromosome copy is known, Structure can be applied for any ploidy level [10]. However, most next-generation sequencing data provide access to unphased genotyping, i.e. consisting of an allele dosage over chromosome copies at each marker, without knowledge of haplotype arrangement along chromosomes. To the best of our knowledge, only Ancestry HMM can be applied to polyploids in the presence of unphased genotyping [14, 15], although it has difficulty managing high ploidy and a large number of ancestral groups. Recently, Martin [16] proposed a methodology to infer ancestral recombination graphs along the genome using unphased genotyping data for any ploidy level. It provides sets of trees that can be used to detect ancestry switches or recombination events, but it does not give direct access to local ancestry dosages or proportions along the genome.

Sugarcane is a major crop for sugar and energy production, and has one of the most complex genomes of all cultivated plants. Modern sugarcane cultivars were derived from a few interspecific hybridizations performed by breeders a century ago between the sugar-rich domesticated group *Saccharum officinarum* (2n=8x=80, x=10) and the wild species *Saccharum spontaneum* (2n=5x=40 to 16x=128; x=8, with many aneuploids). These hybridizations aimed to introduce resistance to overcome the vulnerability of *S. officinarum* to disease and insects. They were followed by backcrossing with *S. officinarum* to restore sugar content, a process referred to as “nobilization”. Modern cultivars are thus complex polyploids, with around 100 to 120 chromosomes and an average of twelve copies for each set of chromosomes [17], of which 15 to 25% are derived from *S. spontaneum*, including recombinants with *S. officinarum* [18, 19]. The recent availability of genome assemblies for *S. spontaneum* accessions [20, 21] and modern cultivars [22–25] constitutes key resources for improving our understanding of the genome architecture in the *Saccharum* genus.

Recently, in Garsmeur et al. [26], we performed a diversity analysis on a large panel of 390 accessions of *Saccharum* species and related genera, using a multifaceted approach involving chloroplast phylogeny, nuclear SNP analysis and an innovative analysis based on repeated k-mers as a proxy for transposable elements (TEs). Notably, we confirmed that *S. officinarum* was domesticated from the wild species *Saccharum robustum* (mainly 2n=6x=60 or 8x=80 and up to 200, with x=10 and many aneuploids) and suggested that the *S. robustum* involved in the domestication process were admixed between subgroups of *S. robustum*. We also identified three main genetic groups in the wild species *S. spontaneum* related to their geographical origin: Continental Asia gathering accessions mainly from India to Thailand and China, Southeast Asia with accessions mainly from Indonesia, and Northeast Asia comprising accessions from Japan, Taiwan and Eastern China. Finally, we identified the contribution of an unknown *Saccharum* ancestor, most likely originating from the Pacific islands, to the genome of most modern cultivars. However, with the exception of cultivars for which a genome assembly has been obtained at the chromosome level or for which molecular cytogenetic observations have been applied, these contributions remain to be characterized and quantified along the genome.

The objectives of this study were to (i) develop an approach for admixture inference, both genome-wide and locally along chromosomes, generalized to mixed-ploidy populations, (ii) evaluate the approach using simulations in terms of the precision of parameter estimates, and (iii) apply the method to the population of 390 accessions of *Saccharum* and a few related genera, as described in Garsmeur et al. [26], to characterize the contribution of ancestral *Saccharum* species to the genome of interspecific sugarcane cultivars.

## Results

### AdmixPoly overview

**Fig. 1:**
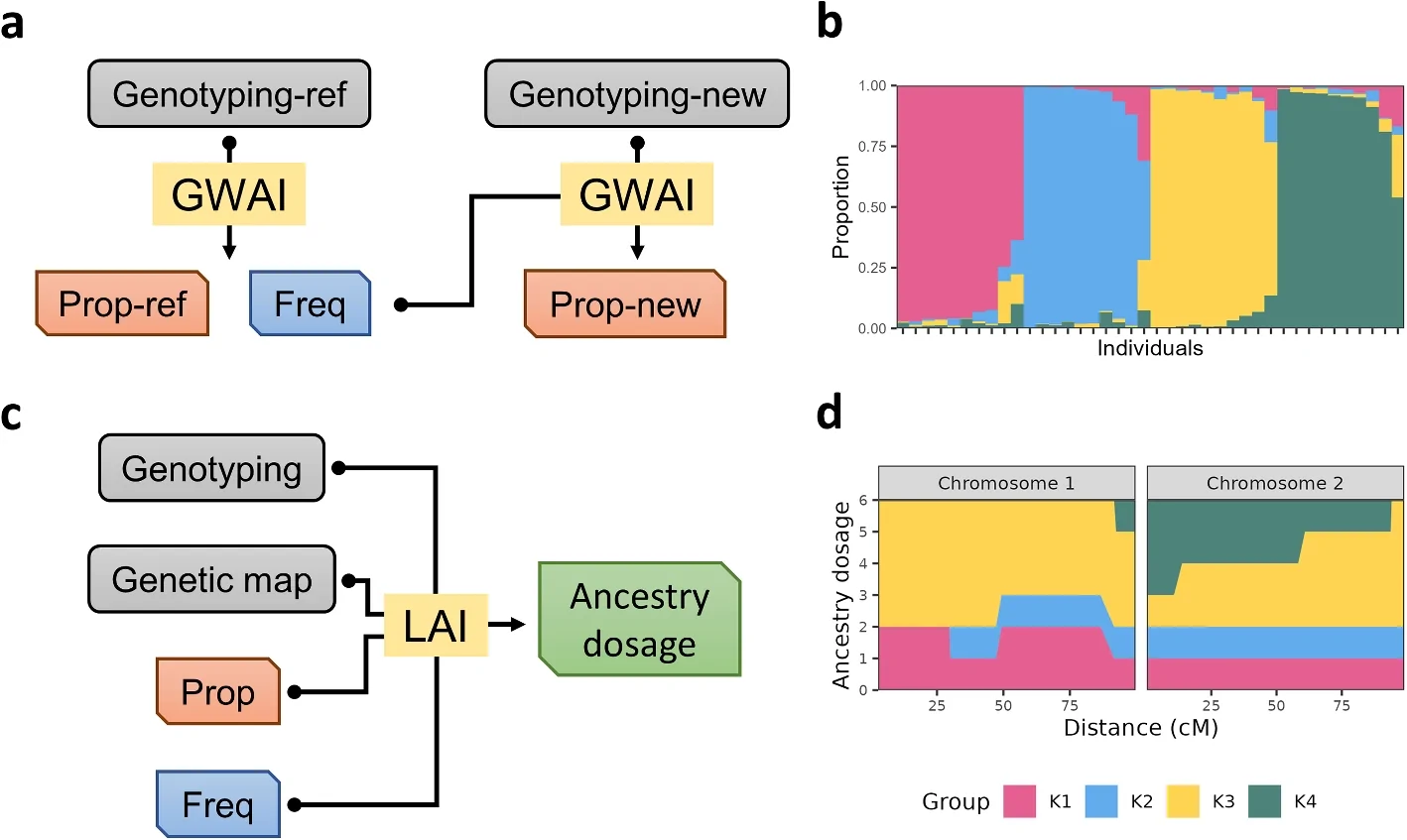
Overview of the AdmixPoly workflow. **a** Genome-wide admixture inference (GWAI). This step involves analyzing a genotyped reference panel (Genotyping-ref) to infer admixture proportions (Prop-ref) and ancestral allele frequencies (Freq). These frequencies can then be used to infer admixture proportions (Prop-new) for new genotyped accessions (Genotyping-new). **b** Example of admixture barplot displaying admixture proportions for a set of individuals. **c** Local admixture inference (LAI). This step utilizes genotyped accessions (Genotyping) with mapped markers (Genetic map), along with previously estimated admixture proportions (Prop) and ancestral allele frequencies (Freq), to infer local ancestry along chromosomes (Ancestry dosage). **d** Example of local admixture plot for one individual with ploidy of six, along two chromosomes displaying local ancestry dosages

A new R-package, AdmixPoly, for admixture inference in populations with mixed-ploidy levels was developed. It allows two types of analyses (Fig. 1): i) inferring genome-wide admixture at the population level and ii) using those estimates to infer the local admixture of each individual. It can accommodate individuals of any ploidy level, each genotyped for bi-allelic or multi-allelic markers.

For genome-wide admixture inference, alleles are assumed to be independent across individuals, loci (referred to here as markers) and chromosome copies. The ancestry of each allele is modeled with a distribution that reflects the genome-wide admixture proportions of the individual across ancestral groups. Conditionally on allele ancestry, alleles are observed with a probability that corresponds to the allele frequency in the ancestral group. An expectation-maximization (EM) algorithm is employed to estimate model parameters and uses an acceleration method and parallelization over markers to enhance computational efficiency. Since parameter values can be initialized and the EM can be constrained to update only one type of parameter (i.e. either admixture proportions or ancestral allele frequencies), a two-step approach can be applied in which ancestral allele frequencies are inferred from a reference panel of accessions and then considered as fixed parameters to infer admixture proportions in a new admixed population of interest (Fig. 1a).

Local admixture along chromosomes is modeled using a HMM adapted from Falush et al. [10] and generalized to arbitrary ploidy levels, which accounts for the dependency between ancestral states at adjacent markers. It requires knowledge of the ordering and genetic distance (or a proxy for it) between adjacent markers along chromosomes (Fig. 1c). Several computational and algorithmic tricks were implemented to make the inference computationally efficient for high ploidy levels, including i) the use of an approximation of the emission probability distribution when the number of alleles is high, and ii) two alternative approximations for calculating transition probabilities when the number of ancestral groups is high. The first approximation of transition probabilities consists of a simplification of genetic distances between adjacent markers, while the second consists of limiting the number of recombinations authorized to move from one ancestry state to another between adjacent markers. A forward-backward algorithm is used to estimate local ancestry dosages at each marker from the probability of being in a particular ancestry state given the observed allele dosages over all markers.

### Evaluation of genome-wide admixture precision

The impact of various population parameters and data characteristics on the precision of genome-wide admixture inference was evaluated through simulation using a reference scenario of 4 groups, 600 individuals, a ploidy of 8, 1,000 markers distributed over 10 chromosomes, and 10 alleles per marker. Genotypic data for a reference population were simulated by modulating each of the parameters around the reference scenario. The precision of admixture proportion and ancestral allele frequency estimates was evaluated using the root mean square error (RMSE) of estimation (Additional file 1: Fig. S1). In general, the RMSE of model parameters increased with the number of groups but decreased with the number of individuals, markers per chromosome, alleles per marker, and ploidy level. In addition, a “mixed-ploidy” scenario was investigated by randomly sampling ploidy levels of individuals, which also showed a low RMSE (Additional file 1: Fig. S1c).

To evaluate the precision of the two-step approach (Fig. 1a), 600 additional individuals were simulated using the same ancestral allele frequencies used to simulate the reference population. Admixture proportions of the new individuals were inferred using the ancestral allele frequency estimated using the reference population and showed similar trends in terms of RMSE (Additional file 1: Fig. S1).

The ability of the Bayesian information criterion (BIC) to identify the number of simulated groups was evaluated using the same simulations described above, comparing the BIC values of the tested number of groups (Additional file 1: Fig. S2). As expected, the minimum BIC value was obtained for a tested number of groups equal to the simulated one.

The impact of relatedness on the precision of model parameter estimates was investigated by simulating 300 pairs of admixed individuals (600 in total) with varying degrees of relatedness. The RMSE increased with increasing relatedness, reaching values similar to those of a population of 300 unrelated individuals (Additional file 1: Fig. S3). These results showed that relatedness reduces the effective amount of independent information for the inference.

Two contrasting simulation scenarios involving sequencing data with variable depths were evaluated (ranging from 1x to 100x): Scenario 1 with a ploidy of 6 and 2 alleles per marker, and Scenario 2 with a ploidy of 12 and 10 alleles per marker. Allele dosages were inferred from sequencing data by applying a proper genotype call (only in Scenario 1 due to prohibitive computing time in Scenario 2), or by applying a proxy based on allele depth ratios (allele depth to the total read depth) multiplied by the ploidy. The RMSE of admixture proportions and ancestral allele frequencies estimated by AdmixPoly was low, even in presence of low-depth sequencing data, and very similar between the genotype call and the proxy (Additional file 1: Fig. S4a and S4b).

**Fig. 2:**
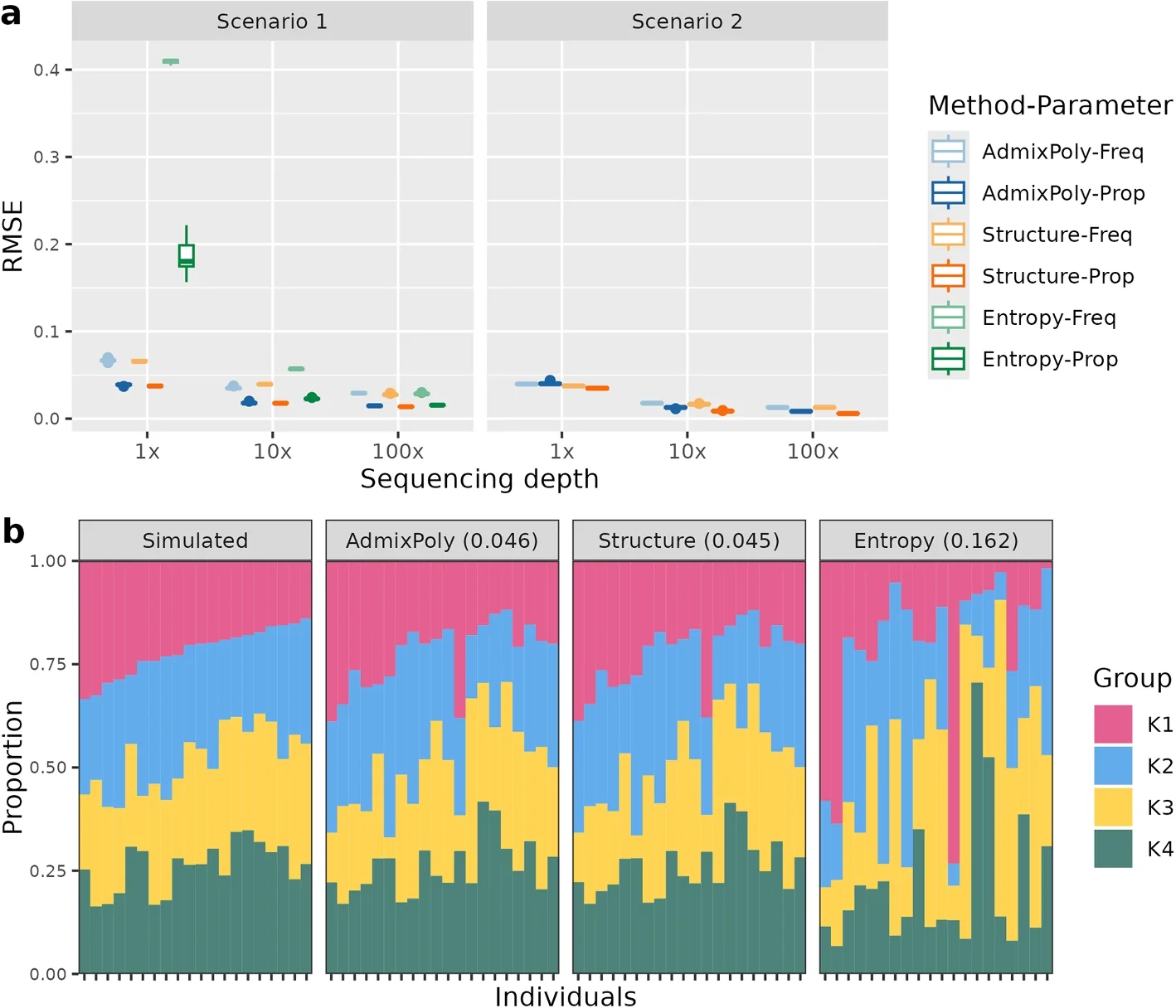
**a** Comparison of the RMSE of genome-wide admixture proportions (Prop) and ancestral allele frequencies (Freq) between AdmixPoly, Entropy and Structure considering two contrasting scenarios: Scenario 1 with a ploidy of 6 and 2 alleles per marker, and Scenario 2 with a ploidy of 12 and 10 alleles per marker. Different sequencing depths were considered at each marker: 1x, 10x and 100x. Note that Entropy cannot be applied in Scenario 2. **b** For a subset of 20 individuals of Scenario 1 with 1x depth, the admixture proportions estimated using each of the methods are compared to the expected admixture proportions (Simulated), with corresponding RMSE between brackets

The genome-wide admixture inference approach implemented in AdmixPoly was compared to two other methods commonly used for genome-wide admixture inference in polyploid species: Entropy and Structure. The three methods were first compared using Scenario 1 cited above (Fig. 2a). The results showed low and comparable RMSE for AdmixPoly and Structure, but high RMSE for Entropy in the case of 1x sequencing depth. These RMSE values were illustrated using a sample of admixture proportions in the low sequencing depth scenario (Fig. 2b), which confirmed both the high similarity of the estimates between AdmixPoly and Structure, which were close to the expected proportions, and large errors in admixture proportion estimates for Entropy.

In Scenario 2, only AdmixPoly and Structure could be compared, as Entropy is limited to a ploidy of 6 and 2 alleles per marker. Again, the RMSE of AdmixPoly and Structure was comparable and very low (Fig. 2a).

The three approaches were also compared for their computing time using Scenario 1 (Table 1), which revealed that AdmixPoly was on average 46x faster than Structure and 2,063x faster than Entropy using a single CPU. While Structure cannot be parallelized and must be run using a single MCMC chain, Shastry et al. [13] recommend three independent chains for Entropy that can be parallelized, making it three times faster using three or more CPUs. In comparison, AdmixPoly showed a significant speedup when utilizing multiple CPUs, demonstrating its ability to scale efficiently with additional computational resources.

**Table 1:** Comparison of the average computing time (in seconds) and standard errors (Std Error) of genome-wide admixture inference between AdmixPoly, Structure and Entropy, considering Scenario 1 over all sequencing depths (1x, 10x and 100x). Different numbers of central processing units (CPUs) were compared and “-” was reported when the method did not benefit from this number of CPUs. For Entropy and Structure, the MCMC settings are those recommended in their documentation

	1 CPU		3 CPUs		10 CPUs	
Method	Average	Std error	Average	Std error	Average	Std error
AdmixPoly^a^	91.2	4.1	31.4	1.2	15.9	1.0
Structure^b^	4 205.2	178.7	-	-	-	-
Entropy^c^	188 195.3	377.4	63 986.3	217.9	-	-

^a^EM algorithm with $10^{-6}$ log-likelihood convergence criterion

^b^MCMC with 10000 burnin iterations + 10000 samples

^c^MCMC with three independent chains of 10000 burnin iterations + 20000 samples/10 (thinning)

### Evaluation of local admixture precision

The impact of population parameters and data characteristics on the precision of local admixture inference was evaluated through simulation using a reference scenario of 4 groups, 600 individuals, a ploidy of 6, 1,000 markers distributed over 10 chromosomes, 10 alleles per marker, and 1 generation since admixture. Genotypic data were simulated by modulating each of the parameters around the reference scenario. The precision of local ancestry proportions was evaluated using the RMSE of 10 accessions among the 600 (Additional file 1: Fig. S5). The RMSE increased with the number of groups and the number of generations since admixture, but decreased with the number of individuals, the ploidy level, the number of markers per chromosome, and the number of alleles per marker. The posterior probabilities obtained from the forward-backward algorithm showed a lower RMSE compared to those obtained from the Viterbi algorithm, and were therefore used for the rest of the analyses.

The impact of the time since admixture parameter on the RMSE was evaluated using the same simulations (Additional file 1: Fig. S6). For a given number of markers, the lowest RMSE was obtained when the simulated and tested time since admixture were equal. However, as the number of markers per chromosome increased, the differences in RMSE between tested time since admixture became smaller. The use of a genetic map proxy (e.g. based on physical distances), with a nonlinear relationship to the true genetic map, also showed limited impact, in particular for a large number of markers (Additional file 1: Fig. S7).

The precision of local ancestry proportion estimates was evaluated using the two scenarios involving variable sequencing depths (Additional file 1: Fig. S4c). The RMSE proved to be moderate to high only for very low read depths (1x and 3x). The method used to account for sequencing data (i.e. genotype call or proxy) did not produce major differences in RMSE.

**Fig. 3:**
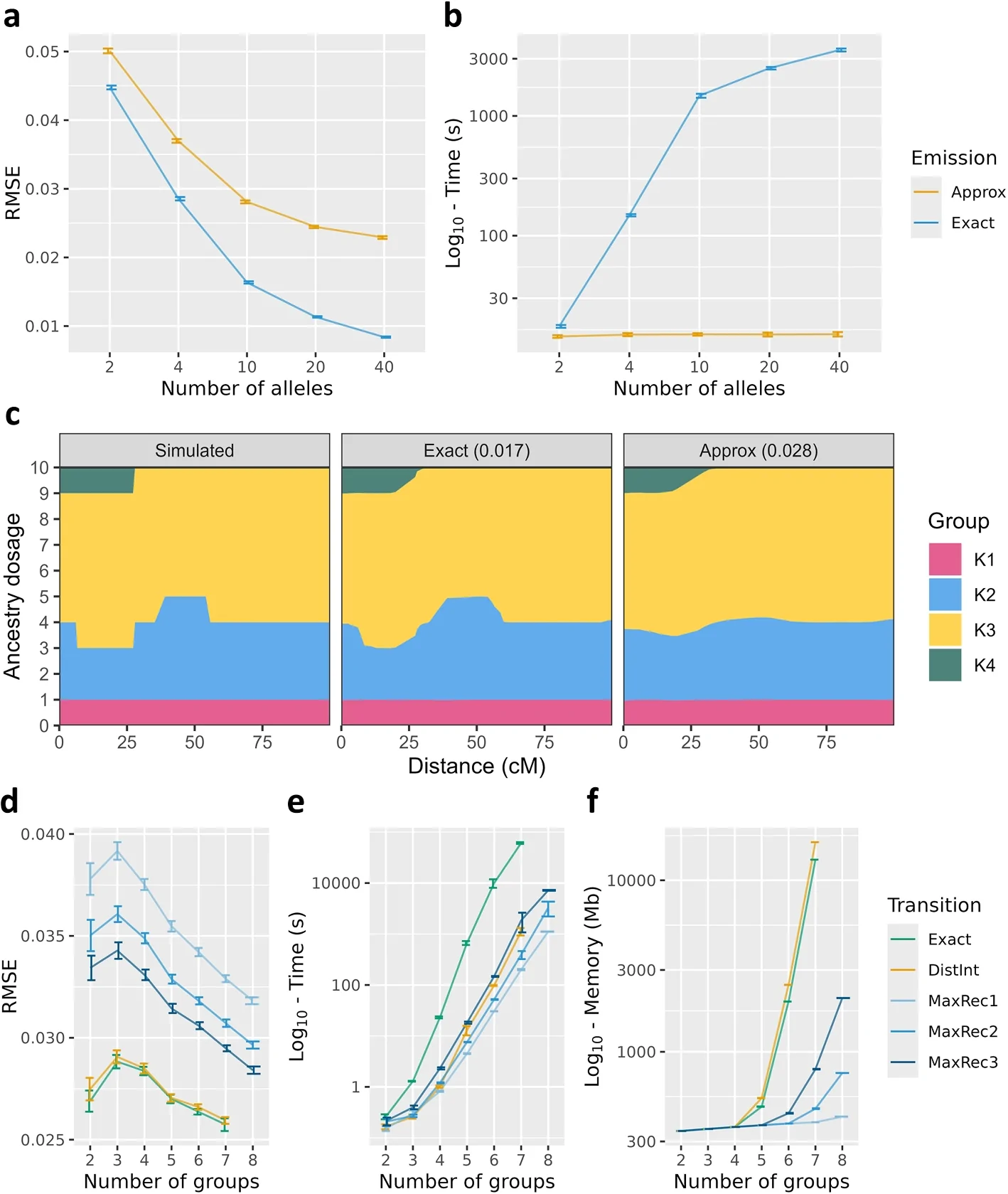
Comparison of the exact (Exact) and approximate (Approx) emission probability distribution for the local admixture inference in terms of **a**) RMSE and **b**) computing time (in seconds, log_10_ scale) according to the number of alleles per marker, and **c**) comparison between the simulated local admixture of one chromosome and those inferred using the exact and approximate emission probability distributions (10 alleles per marker), with the corresponding RMSE indicated between brackets. Comparison of methods to compute transition probabilities in terms of **d**) RMSE, **e**) computing time (in seconds, log_10_ scale) and **f**) memory (in MB, log_10_ scale) according to the number of groups. Methods include exact (Exact) and approximate transition probabilities based on a simplification of genetic distances between adjacent markers (DistInt), or by limiting the number of recombinations authorized to move from one ancestry state to another between adjacent markers (MaxRec1 to MaxRec3). The reference set of simulation parameters is: 4 groups, a ploidy of 10, 100 markers per chromosome, 10 alleles per marker, and 1 generation since admixture. For all subplots but **c**), standard error bars are represented

Calculating the exact emission probability distribution can be prohibitively time-consuming as the ploidy and number of alleles increase. We compared the RMSE of the exact emission probability distribution to that of an approximation. The simulation consisted of inferring the local admixture of 10 individuals with a ploidy of 10 and genotyped for markers with a variable number of alleles per marker (Fig. 3a). The approximation showed an RMSE slightly higher than the exact emission probability distribution, but also proved to be several orders of magnitude faster to calculate, particularly for 10 or more alleles per marker (Fig. 3b). The limited impact of using the approximation was illustrated for one chromosome using a local admixture plot (Fig. 3c).

Similarly, calculating exact transition probabilities can be prohibitively time-consuming and memory-intensive. This is due to the storage of large transition matrices as the ploidy and the number of groups increase, e.g. 18,564 ancestry dosage states for a ploidy of 12 and 7 ancestral groups. We compared the RMSE, computing time and memory usage of the exact transition probability to those of approximate transition probabilities based on a simplification of genetic distances between adjacent markers (DistInt), or by limiting the number of recombinations (one in MaxRec1, two in MaxRec2 and three in MaxRec3) authorized to move from one ancestry state to another between adjacent markers (Fig. 3d). The analysis consisted of estimating the local admixture of 10 individuals with a ploidy of 10 considering a variable number of groups. While the DistInt approximation had an RMSE similar to the exact transition probabilities, the MaxRec approximations showed a higher RMSE, especially for a low number of allowed recombinations like MaxRec1. However, MaxRec1 was also the fastest method to calculate and used very limited memory even for high numbers of ancestral groups (Fig. 3e and f).

The local admixture inference approach implemented in AdmixPoly was compared to Ancestry HMM, another admixture method designed for polyploids. The two methods were compared for variable ploidy levels and number of ancestral groups, but considering only bi-allelic markers, which are the only markers usable by Ancestry HMM. Although AdmixPoly was applied using exact emission and transition probabilities, it showed faster computing time, less memory usage but also lower RMSE when compared to Ancestry HMM (Additional file 1: Fig. S8). Furthermore, Ancestry HMM was unable to handle complex cases with reasonable computing resources, e.g. high ploidy levels (≥ 10) and number of ancestral groups (≥ 5).

### Genome-wide admixture of *Saccharum*

A set of 390 accessions of wild and cultivated *Saccharum* groups and a few representatives from related genera, as described in Garsmeur et al. [26], was analyzed in this study. For all accessions, high-depth whole genome sequencing (WGS) data were available, and they were genotyped for 80K haplotypic regions targeting genes using HaploCharmer [27], revealing a total of 3,651,114 haplotypes (43.52 haplotypes per region on average, Additional file 1: Fig. S9).

**Fig. 4:**
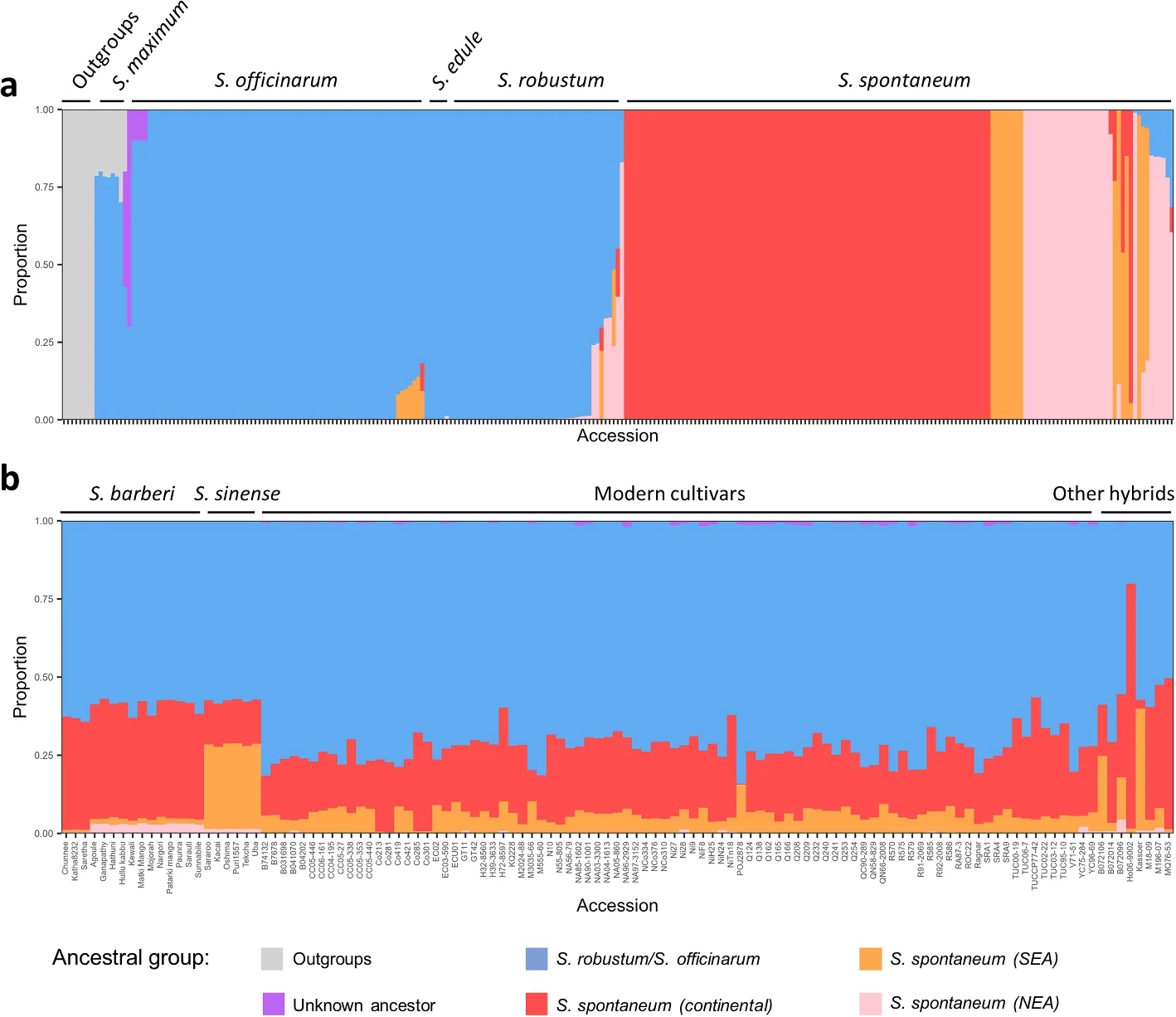
**a** Admixture barplot of *Saccharum* accessions (excluding hybrid cultivars) and related genera. Admixture is estimated considering six ancestral groups: a group formed of *Tripidium*, *Miscanthus*, *Narenga* and *E. fulvus* (outgroups), the unknown ancestral contributor, a group formed of *S. robustum*, *S. officinarum*, and *S. edule*, Continental Asia *S. spontaneum*, Southeast Asia (SEA) *S. spontaneum,* and Northeast Asia (NEA) *S. spontaneum*. **b** Admixture barplot of modern and former interspecific hybrid cultivars (*S. barberi* and *S. sinense*). “Other hybrids” include the energycane cultivar Ho06-9002 and base-broadening germplasm

Genome-wide admixture was first investigated on all accessions excluding interspecific hybrid cultivars (modern cultivars, *S. barberi* and *S. sinense*). Following Garsmeur et al. [26], five *Saccharum* ancestral groups were considered (Fig. 4a): a group including *S. officinarum*, *S. robustum* and *S. edule* accessions, three *S. spontaneum* subgroups (Continental Asia, Southeast Asia and Northeast Asia), and an unknown ancestral group. In addition, a sixth composite outgroup was considered that included accessions from other genera: *Tripidium*, *Miscanthus*, *Narenga*, and *Erianthus fulvus*.

This analysis confirmed that a few of the analyzed *S. officinarum* and *S. robustum* accessions were hybrids or introgressed with *S. spontaneum*, and the other way round for a few *S. spontaneum* accessions. Results were also in line with *S. maximum* accessions being hybrids between *Miscanthus* and *S. officinarum* or *S. robustum*. In Garsmeur et al. [26], only a few accessions in the dataset (i.e. RaiateaX, NC100, NC29, NC30, and NC93) showed significant contributions from an unknown ancestor, with no pure representative. To take this group into account, it was necessary to force its appearance by i) first performing admixture inference without accounting for it, and ii) adding its contribution to the above-mentioned accessions using estimates obtained from cytogenetic observations (Additional file 1: Fig. S10, see Materials and Methods for more details).

Ancestral allele frequencies estimated from this first panel of accessions were used to infer the genome-wide admixture proportions of hybrid cultivars (Fig. 4b). Admixture proportion estimates confirmed that modern cultivars inherited on average 73% of their genome from *S. officinarum* (varying from 56% to 83%) and 26% from *S. spontaneum* (varying from 16% to 44%). Most of the modern cultivars had *S. spontaneum* contributions from Continental Asia and Southeast Asia. Exceptions included a few cultivars from Coimbatore (India) that only had Continental Asia *S. spontaneum* contributions, such as Co281, or the famous cultivar POJ2878 from Java (Indonesia) that only had Southeast Asia *S. spontaneum* contributions. Only a few cultivars had *S. spontaneum* contributions from Northeast Asia, including cultivars from Japan like Ni28. Finally, around 80% of the modern cultivars had a contribution from the unknown ancestral group, which represented on average 1% of their genome. Additional hybrids were included in this panel that corresponded to “energycane” cultivars like Ho06-9002 with a 78% Continental Asia *S. spontaneum* contribution or other base-broadening germplasm with increased *S. spontaneum* contributions as compared to modern cultivars.

Admixture proportions of *S. barberi* and *S. sinense* were estimated in the same way as for modern cultivars (Fig. 4b). Three main profiles could be identified that varied in terms of the contributing *S. spontaneum* subgroups: i) a first group of three *S. barberi* accessions (i.e. Chunnee, Katha 8232 and Saretha) with only a contribution from Continental Asia *S. spontaneum*, ii) a second group comprising the remaining *S. barberi* accessions with a contribution mainly from Continental Asia *S. spontaneum* and minor contributions from the two other *S. spontaneum* subgroups, and iii) a third group comprising the *S. sinense* accessions with contributions mainly from Southeast Asia *S. spontaneum* and Continental Asia *S. spontaneum*.

### Local admixture along modern cultivar genomes

**Fig. 5:**
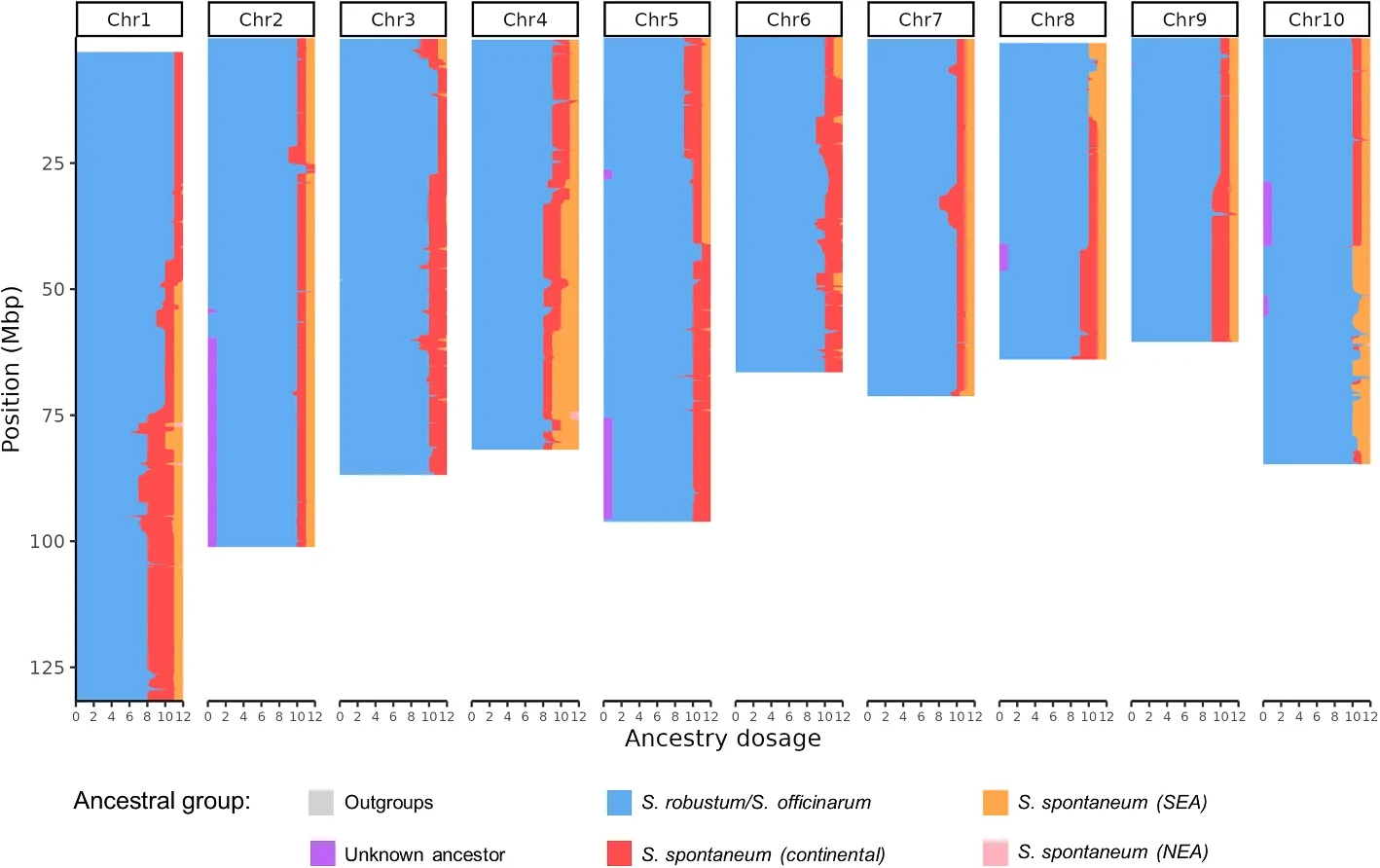
Local admixture of the modern cultivar R570. Admixture is estimated considering six ancestral groups: a group formed of *Tripidium*, *Miscanthus*, *Narenga,* and *E. fulvus* (outgroups), the unknown ancestral contributor, a group formed of *S. robustum* and *S. officinarum*, Continental Asia *S. spontaneum*, Southeast Asia (SEA) *S. spontaneum* and Northeast Asia (NEA) *S. spontaneum*

Estimates of ancestral allele frequencies and admixture proportions of the cultivar R570 were used to estimate its ancestry dosages along chromosomes (Fig. 5). These ancestry dosages were compared to ancestry dosages deduced from a genetic map obtained from the R570 self-progeny, as illustrated for chromosome 5 (Additional file 1: Fig. S11). The results showed a very good consistency of the local admixture obtained from both approaches, which confirmed the accuracy of AdmixPoly in the context of highly polyploid sugarcane. Regarding the contribution of the unknown ancestor to the genome of R570, Garsmeur et al. [26] identified genomic segments on chromosomes 2 and 10 by mapping repeated k-mers specific to the unknown ancestor onto the R570 primary polyploid genome assembly [23]. These segments were also identified here, along with additional segments on chromosomes 5 and 8. These latter segments corresponded to unanchored scaffolds that were not included in the primary assembly of Healey et al. [23].

Local admixture was applied to all other modern cultivars (Additional file 2). The average local admixture over modern cultivars showed little variation in *S. officinarum* and *S. spontaneum* contributions, with on average 8.7 and 3.2 ancestry dosages along the genome, respectively (Additional file 1: Fig. S12). These average estimates concealed significant variability of admixture proportions among accessions, with a standard deviation of around 1.1 for both contributing species along the genome.

Analysis of the contribution of the *S. spontaneum* subgroups to the genome of modern cultivars revealed different patterns according to the subgroup (Additional file 1: Figs S13, S14, S15, and Additional file 2). Nearly all modern cultivars showed a contribution from Continental Asia *S. spontaneum*, representing up to 7 copies of chromosome segments (2.5 on average). A notable exception was POJ2878, which had no significant contribution from this subgroup. Modern cultivars generally had a lower contribution from Southeast Asia *S. spontaneum*, with 0 to 5 (0.7 on average) copies of chromosome segments. Finally, very few cultivars presented substantial contributions from Northeast Asia *S. spontaneum*. The only accessions that showed segments of the order of one chromosome arm size were Ni28 and NiN24 from Japan, B031698 and B041070 from Barbados, and YC75-284.

**Fig. 6:**
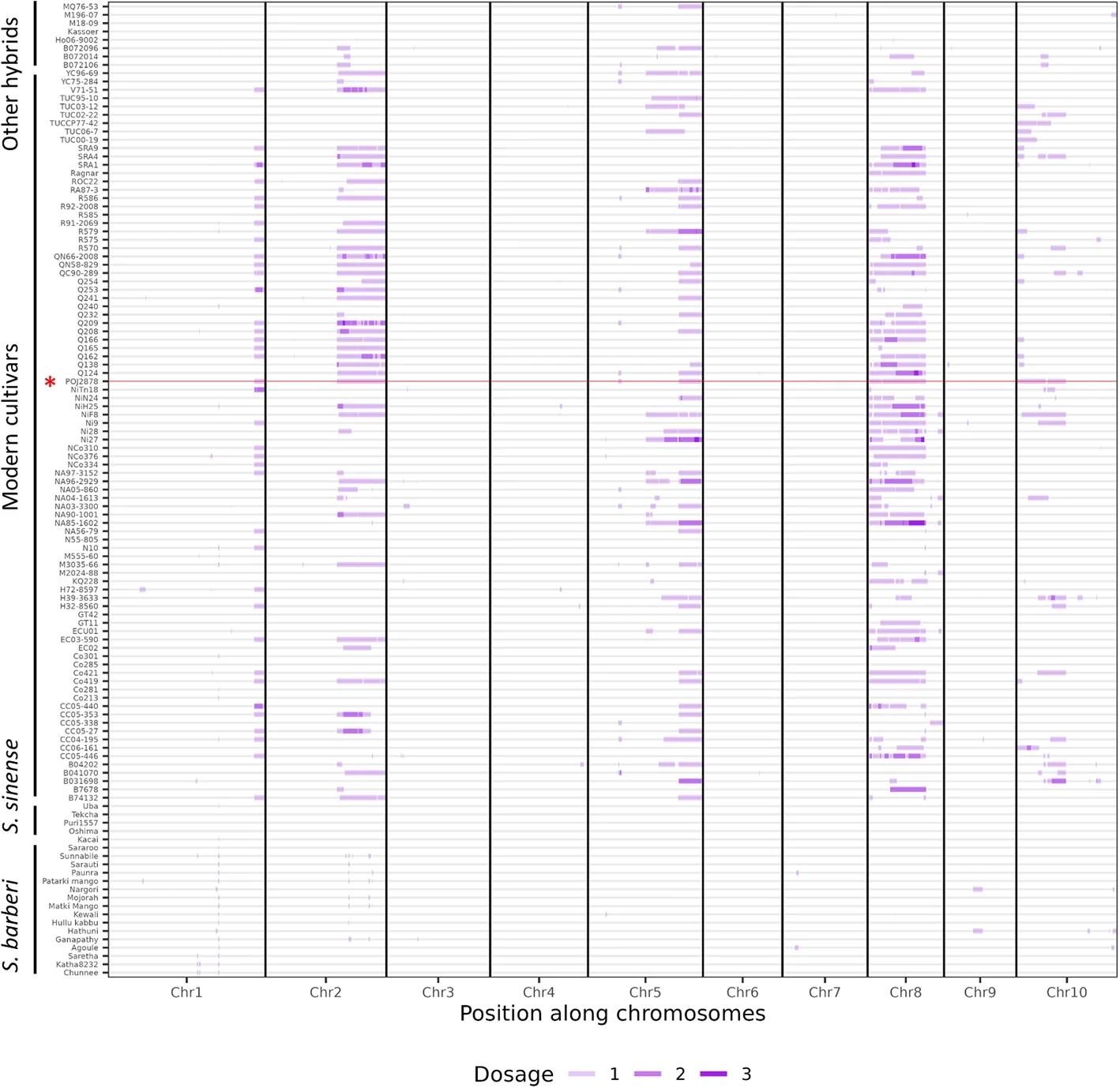
Shared contributions from the unknown ancestor between modern and former interspecific hybrid cultivars. The positioning of POJ2878 is indicated by a red star and a horizontal red line

Analysis of the contribution of the unknown ancestor to the genome of modern cultivars revealed a pattern of shared genomic segments (Fig. 6), notably on chromosomes 1, 2, 5, 8, and 10. Most of these contributions were single copies in the cultivars, but they could go up to 3 copies, (e.g. on chromosome 8 of NA85-1602). The accession POJ2878 is known to have contributed to the pedigree of most cultivars. Interestingly, it showed almost all of the significant segments found in other cultivars, with the exception of a part of the chromosome 5 segment.

## Discussion

### Polyploid admixture inference with AdmixPoly

While admixture inference has become a standard approach in genetic diversity studies based on genomic data, its application to polyploid species has suffered from a lack of suitable tools, in particular to analyze large genome-wide datasets [28, 29].

Regarding genome-wide admixture inference, Structure has been able to deal with polyploid datasets since version 2 [10], as well as mixed-ploidy datasets following the encoding presented by Stift et al. [12]. In our simulations, we confirmed that Structure accurately infers admixture proportions as well as ancestral allele frequencies. However, it suffers from a prohibitive computation time as the number of markers increases, making it impractical for modern datasets with millions of markers. The highly efficient software developed afterwards, such as Admixture [9] or fastStructure [7], have strictly focused on bi-allelic diploid genotypic coding. They were shown to be subject to biased inference when applied to polyploid datasets with diploidized genotypic coding [12]. Recently, Entropy has been developed specifically for species with a ploidy level up to six [13]. However, in addition to a prohibitive computation time, we have shown that Entropy can exhibit significant estimation biases when sequencing depth is low. This result was surprising as Entropy was developed to account for uncertainty in genotypic data through the use of genotype likelihoods.

Our new R-package AdmixPoly has demonstrated a precision similar to Structure for genome-wide admixture inference in polyploid species. In terms of computation time, its efficient EM algorithm, which uses an acceleration method and parallelization over markers, delivers computation time gains of several orders of magnitude compared to Structure. In this study, it allowed the analysis of a large dataset comprising 390 accessions genotyped for 80K genomic regions involving around 3.7M haplotypes.

Another main advantage is the ability to perform semi-supervised inference by constraining the EM algorithm to update only one type of parameter (i.e. either admixture proportions or ancestral allele frequencies). As already proposed in Admixture [9], semi-supervised inference enables training the model on a reference population and using estimated ancestral allele frequencies as fixed parameters to infer admixture proportions of new admixed individuals. This is particularly useful when a population of admixed individuals shows very similar admixture profiles, which tends to reveal the existence of a false ancestral group. This phenomenon has already been observed in the study by Yang et al. [30] with the definition of an ancestral group composed of modern sugarcane cultivars, as this group is not an ancestral group in itself, given that modern cultivars are man-made interspecific hybrids between *S. officinarum* and *S. spontaneum*. In this study, we applied this procedure by excluding modern cultivars, *S. barberi,* and *S. sinense* from the reference population used to estimate ancestral allele frequencies and then estimating their admixture proportions in a second step.

Semi-supervised analyses also enable us to account for an ancestral group that is poorly sampled in the reference training population, i.e. rare and with no pure representatives, as illustrated with an unknown *Saccharum* contributor using an admixture analysis based on repeated k-mers [26] and in this study using haplotype-based genotyping.

An important aspect of genome-wide admixture inference concerns the choice of the number of ancestral groups *K* to be considered. For results generated by Structure, Evanno et al. [31] have proposed considering the rate of change in the likelihood between successive *K* values. Admixture [9] and fastStructure [7] have implemented a cross-validation approach. In AdmixPoly, we propose using the Bayesian information criterion (BIC), which can be easily computed from the marginal likelihood, and we showed that it accurately identifies the simulated *K* values in the simulations. However, one should keep in mind that the choice of *K* should be mainly guided by knowledge of a population’s history [9].

To the best of our knowledge, Ancestry HMM is the only tool developed for local admixture inference based on unphased genotypic data in polyploid species [14, 15]. Based on our simulations, we not only confirmed its accuracy but also highlighted some limits in terms of computing time and memory usage when the ploidy and the number of ancestral groups increase. Other drawbacks include the restriction to bi-allelic markers, which can be insufficiently informative in a polyploid context, and the need to provide admixture proportions and ancestral allele frequencies computed using other software.

Thanks to several computational and algorithmic tricks, AdmixPoly demonstrated its ability to perform local admixture inference in complex scenarios with high ploidy levels, numbers of ancestral groups, and alleles per marker. The approximate emission probability distribution showed very little loss of precision, while enabling calculations that would otherwise be prohibitively time-consuming in a multi-allelic context. Regarding transition probabilities, we suggest applying the approximation based on a simplification of genetic distances between adjacent markers for moderately complex scenarios, as it has demonstrated similar accuracy to exact transition probabilities while allowing major computation time gains. For more complex scenarios with high ploidy and a large number of ancestral groups, we recommend using the approximation limiting the number of recombination events, as it dramatically reduces the amount of memory required to calculate large square matrices. In this study, AdmixPoly enabled inference of the local admixture of modern sugarcane cultivars with a ploidy of twelve, genotyped for highly multi-allelic markers and assuming six ancestral groups. The high accuracy of the local admixture estimates in this complex scenario was confirmed using an alternative local admixture approach based on genetic mapping applied to chromosome 5 of R570 (Additional file 1: Fig. S10).

One challenging aspect of the local admixture HMM model is determining the rate *t* at which breakpoints between blocks of homogeneous ancestry occur per unit of genetic distance. This rate can be interpreted as the number of generations since the first admixture occurred [10]. In AdmixPoly, this parameter is treated as fixed, and we suggest using it as a smoothing parameter that controls the size of the ancestry blocks at the haplotype level. In practice, when the number of markers is large, the impact of this rate *t* is relatively minor, provided that its value is within an acceptable range (Additional file 1: Fig. S6). Thanks to the computational efficiency of our procedure, the data likelihood may be computed for different values of *t*, allowing the application of a golden section search [14].

While AdmixPoly theoretically requires a genetic map to be applied, a proxy based on physical distance is a convenient option for most applications. Even though physical distances have a non-linear relationship with genetic distance along chromosomes, their use as a proxy has little impact in practice, in particular when the number of markers is large (Additional file 1: Fig. S7).

### The mosaic genome of sugarcane cultivars

In this study based on around 3.7M read-scale haplotypes, we detected a population structure consistent with that found by Garsmeur et al. [26], who used the same dataset and an analysis based on repeated k-mers.

Similarly, all *S. robustum*, *S. officinarum,* and *S. edule* accessions were gathered into a single ancestral group, which made sense as *S. officinarum* and *S. edule* were likely domesticated from *S. robustum* [26]. Although *S. officinarum* likely originated from a domestication process involving complex admixture between *S. robustum* subgroups, it does not exhibit significant subdivision into subgroups [26]. This suggests that admixture profiles are similar across *S. officinarum* accessions and that the delineation of a single ancestral group of *S. officinarum/S. robustum/S. edule* had little impact for the study of the contribution of parental species to the genome of modern cultivars.

The average genome-wide *S. officinarum* (73%) and *S. spontaneum* (26%) contributions to the genome of modern cultivars were consistent with previous findings based on chromosome *in situ* painting [17–19, 32] and repeated k-mer based admixture [26]. In addition, AdmixPoly allowed access to local admixture estimates and thus to an *in silico* chromosome painting. Until now, this type of information at the chromosomal level was limited to a few cultivars analyzed by molecular cytogenetics or for which genome assembly was available.

Average estimates of local admixture along chromosomes showed some variation (Additional file 1: Fig. S12), which may result from selection toward *S. officinarum* or *S. spontaneum* ancestry, or simply from shared pedigree relationships. A significant variability in the number of *S. officinarum* and *S. spontaneum* chromosome copies was revealed among modern cultivars, which could result from selection and adaptation to different cultivation areas. The variability of local admixture estimates along chromosomes reflects the occurrence of interspecific chromosome recombination, confirming previous cytogenetic [17–19, 32] or genetic mapping [22, 27, 33, 34] studies.

In this study, as in Garsmeur et al. [26], three main *S. spontaneum* subgroups were considered: Continental Asia, Southeast Asia, and Northeast Asia. However, a substructure likely exists within the Continental Asia accessions, as already reported by Aitken et al. [35] and Zhang et al. [20, 21].

In some modern cultivars, and several *S. barberi* and *S. sinense* accessions, the local admixture estimates showed small segments from the three *S. spontaneum* subgroups with frequent variation along chromosomes. These variations may reflect a high level of recombination between the distinct *S. spontaneum* subgroups in the ancestor of these accessions and/or imprecision in the local admixture estimates due to an insufficient number of *S. spontaneum* contributor representatives in our sample.

In most modern cultivars, only the Continental Asia and Southeast Asia *S. spontaneum* subgroups were involved, as already shown in Garsmeur et al. [26]. These results are consistent with interspecific crosses carried out in the early 1900s in Indonesia and India, which led to the first modern sugarcane cultivars.

At the Java Sugar Experiment Station (Proefstation Oost Java, Indonesia), breeders exploited a natural hybrid (Kassoer) supposed to involve a local *S. spontaneum* accession (Glagah) from the Southeast Asia subgroup. This led to the development of successful cultivars, including POJ2878, the ‘wonder cane’, which was quickly adopted worldwide and subsequently involved in the pedigree of most modern cultivars [36, 37]. In line with this pedigree, we confirmed that POJ2878 had a *S. spontaneum* contribution only from the Southeast Asia subgroup. As the Southeast Asia *S. spontaneum* subgroup has contributed to nearly all cultivars and presents a low genetic diversity [26], there is limited potential for harnessing new alleles from this subgroup through prebreeding strategies.

At the Sugarcane Breeding Institute (Coimbatore, India) in the early 1900s, breeders exploited local forms of Continental Asia *S. spontaneum* and *S. barberi* from the Chunnee/Saretha subgroup [38]. We showed that the Chunnee/Saretha subgroup had only a *S. spontaneum* contribution from Continental Asia. Consistently, several cultivars originating from this breeding program (e.g. Co213, Co281, Co285, Co301) presented only (or nearly only) a *S. spontaneum* contribution from Continental Asia. Although Continental Asia *S. spontaneum* is already the main contributor to the *S. spontaneum* component of most cultivars' genome, its large diversity [26] likely makes it an almost inexhaustible source of alleles for sugarcane prebreeding.

In general, a lower proportion of Southeast Asia *S. spontaneum* was observed in cultivars compared with Continental Asia *S. spontaneum*. This difference is probably due to contrasting *S. spontaneum* contributions in the initial hybrids, as illustrated with POJ2878 which has a lower *S. spontaneum* proportion than the Coimbatore cultivars (Fig. 4 and S8).

The Northeast Asia *S. spontaneum* subgroup, as opposed to the other two subgroups, showed very limited contributions to the genome of modern sugarcane cultivars. The most significant contributions were found in cultivars originating from a Japanese breeding program, such as Ni28, but were limited to a few chromosome segments. Surprisingly, a few Japanese cultivars like Ni9, NiF8, or NiH25 showed no significant contribution from this *S. spontaneum* subgroup, which was in contradiction with the admixture obtained from repeated k-mers using the same sequencing data [26]. These few differences may be linked to the fact that repeated k-mers capture TE variations that are not necessarily associated with allelic diversity. The Northeast Asia *S. spontaneum* subgroup constitutes an interesting target to mine adaptive or disease resistance alleles. The interest of this untapped genetic diversity has recently been demonstrated with the identification of smut resistance alleles from a mapping population involving an ancestor from the Northeast Asia *S. spontaneum* subgroup [39]. Among the set of hybrid accessions analyzed, a few base-broadening germplasm accessions (e.g. B072096 or M196-07) showed substantial contributions from the Northeast Asia *S. spontaneum* subgroup. This is consistent with the strategies of the breeding programs from Barbados and Mauritius, which use *S. spontaneum* and other wild species accessions in their base-broadening programs [40, 41].

The last contribution to the genome of modern cultivars analyzed in this study concerned an unknown *Saccharum* ancestor, likely originating from East Melanesia or Polynesia [26]. It was taken into account following the approach of Garsmeur et al. [26], i.e. by manually fixing admixture proportions defined based on cytogenetic observations in a few hybrid accessions. We confirmed its contribution to the genome of most modern cultivars, with an average contribution of 1% of the total genome representing approximately one chromosome. Interestingly, we found that the few chromosome segments inherited from the unknown ancestor had a well-conserved pattern across cultivars. This suggests that these chromosome segments were all inherited from one or a few of the early ancestors of modern cultivars. POJ2878, the ‘wonder cane’ from Java, one of these early ancestors, also had an exceptional breeding value, and was involved in the pedigree of almost all modern cultivars [37]. Interestingly, it almost had the complete set of chromosome segments contributed by the unknown ancestor, making it likely that most modern cultivars inherited this contribution from POJ2878. Interspecific hybridization of *S. officinarum* with the wild species *S. spontaneum* performed a century ago was a major breakthrough in sugarcane breeding, solving some of the disease problems and providing additional benefits in increasing yield, ratooning ability, and resistance to various abiotic stresses [42]. These characteristics were attributed to *S. spontaneum*. However, the discovery of contributions from the unknown ancestor during these early phases of modern breeding suggested that some of these traits could have been inherited from this unknown ancestor. Further research will help determine the value of the unknown ancestor alleles in modern breeding germplasm. Identification of pure accessions of this unknown ancestor or additional hybrids with large contributions from it could pave the way for prebreeding strategies to exploit this new source of diversity.

## Conclusions

We have developed AdmixPoly, an efficient R-package for inferring admixture in polyploid species at genome-wide and local chromosome scales. Using AdmixPoly, we characterized the contributions of wild *Saccharum* species to the complex polyploid genome of modern sugarcane cultivars. This R-package will enable researchers to analyse large genomic datasets, thereby helping to advance our understanding of the evolutionary history, hybridisation and admixture in polyploid species.

## Methods

### Genome-wide admixture

Let us consider a population of N individuals with mixed-ploidy levels, where $P_{i}$ is the ploidy level of individual i. The population, initially divided into K ancestral genetic groups, was subject to cross-breeding between ancestors from different groups, resulting in admixture. Individuals are genotyped for a set of M markers, each consisting of $L_m$ alleles.

#### Genetic model

The latent ancestry $A_{imc}$ of the allele carried by the hom(e)ologous chromosome copy c at marker m for individual i is assumed to be drawn from a multinomial distribution:

$$\begin{aligned} A_{imc}\sim \mathcal {M}\left( 1; \pi _{i1},...,\pi _{ik},...,\pi _{iK}\right) \ \textrm{independent,} \end{aligned}$$

where $\pi _{ik}$ corresponds to the genome-wide admixture proportion of individual i in group k with $\sum ^K_{k=1}\pi _{ik}=1$.

The genotype $G_{imc}$ for the hom(e)ologous chromosome copy c at marker m for individual i is also assumed to be drawn from a multinomial distribution conditionally on the allele ancestry:

$$\begin{aligned} \left( G_{imc}|A_{imc}=k\right) \sim \mathcal {M}\left( 1; \gamma _{mk1},...,\gamma _{mkl},...,\gamma _{mkL_{m}}\right) \ \textrm{independent,} \end{aligned}$$

where $\gamma _{mkl}$ is the frequency of allele l at marker m in group k with $\sum ^{L_{m}}_{l=1}\gamma _{mkl}=1$.

In practice $G_{imc}$ is unobserved, and one has only access to the allele dosages $S_{im}=\left\{ S_{im1},...,S_{imL_m}\right\}$ for individual i at marker m, where:

$$\begin{aligned} S_{iml} = \sum \limits ^{P_{i}}_{c=1}G_{imcl} \end{aligned}$$

Let us introduce $A_{imck}=1\!\!1_{\{A_{imc}=k\}}$ and $G_{imcl}=1\!\!1_{\{G_{imc}=l\}}$ with $1\!\!1_{\{\}}$ being the indicator function. The joint log-likelihood is noted $\mathcal {L}_1=\textrm{log}\ p_{\theta }(A,G)$, where θ stands for the set of all unknown parameters, and has the following expression:

$$\begin{aligned} \mathcal {L}_1 = \sum \limits ^N_{i=1}\sum \limits ^M_{m=1}\sum \limits ^{P_{i}}_{c=1}\sum \limits ^K_{k=1}A_{imck}\left[ \textrm{log}\left( \pi _{ik}\right) + \sum \limits ^{L_{m}}_{l=1}G_{imcl}\textrm{log}\left( \gamma _{mkl}\right) \right] \end{aligned}$$

#### Inference

The inference of model parameters is done using an expectation-maximization (EM) algorithm [43]. For each iteration of the EM algorithm, an expectation step is performed in which the expectation $\mathbb {E}_{\theta }\left( \mathcal {L}_1|S\right)$ is calculated and can be simplified as (see proof in Additional file 3: Section 1):

$$\begin{aligned} \mathbb {E}_{\theta }\left( \mathcal {L}_1|S\right) = \sum \limits ^N_{i=1}\sum \limits ^M_{m=1}\sum \limits ^{L_{m}}_{l=1}S_{iml}\sum \limits ^K_{k=1}\tau _{imkl}\left[ \textrm{log}\left( \pi _{ik}\right) + \textrm{log}\left( \gamma _{mkl}\right) \right] \end{aligned}$$

where:

$$\begin{aligned} \tau _{imkl}=\frac{\pi _{ik}\gamma _{mkl}}{\sum ^K_{k'=1}\pi _{ik'}\gamma _{mk'l}} \end{aligned}$$

In the maximization step, model parameters estimates are updated using the following formulas (see proof in Additional file 3: Section 1):

$$\begin{aligned} \hat{\pi }_{ik} = \frac{1}{MP_i}\sum \limits ^M_{m=1}\sum \limits ^{L_{m}}_{l=1}S_{iml}\tau _{imkl} \end{aligned}$$

and

$$\begin{aligned} \hat{\gamma }_{mkl} = \frac{\sum ^N_{i=1}S_{iml}\tau _{imkl}}{\sum ^N_{i=1}\sum ^{L_{m}}_{l'=1}S_{iml'}\tau _{imkl'}} \end{aligned}$$

Note that the following estimators correspond to those of Tang et al. [8] and Alexander et al. [9] generalized to mixed-ploidy levels and more than two alleles.

Storing posterior probabilities $\tau _{imkl}$ may be costly in terms of memory as the number of individuals, markers, alleles per marker, and groups increase. Also, the variable ploidy levels and number of alleles per marker makes their storing inconvenient. These two issues were handled by updating directly model parameters $\pi _{ik}$ and $\gamma _{mkl}$, while calculating $\tau _{imkl}$ on the fly (see pseudo-code of Additional file 3: Section 1), following the framework of De Walsche et al. [44].

The EM algorithm iterates until convergence, which is reached when the marginal log-likelihood of Eq. (1) - defined up to a constant - shows no improvement from one iteration to the next by a certain threshold.

1 $$\begin{aligned} \textrm{log}\ p_{\theta }(S) = \sum \limits ^N_{i=1}\sum \limits ^M_{m=1}\sum \limits ^{L_{m}}_{l=1}S_{iml}\textrm{log}\left( \sum \limits ^K_{k=1}\pi _{ik}\gamma _{mkl}\right) \end{aligned}$$

The EM algorithm was also accelerated using a squared iterative method [45]. Let us consider $\theta ^{t-2}$, $\theta ^{t-1}$, and $\theta ^{t}$ as the EM parameter estimates obtained at iteration t-2, t-1, and *t* of the EM algorithm, respectively. At iteration *t*, one computes:

$$\begin{aligned} \theta ^{t'} = \theta ^{t-2} - 2\alpha r + \alpha ^2v \end{aligned}$$

where $r = \theta ^{t-1} - \theta ^{t-2}$ and $v = \theta ^{t} - \theta ^{t-1} - r$. If the marginal likelihood $\textrm{log}\ p_{\theta }(S)$ of Eq. (1) for $\theta ^{t'}$ is improved compared to $\theta ^{t}$, the accelerated parameter estimates are accepted.

### Local admixture

#### Genetic model

The local admixture along chromosomes can be investigated using latent ancestry dosages $A_{im}=\{A_{im1},...,A_{imK}\}$ at marker *m* for individual *i*, defined as the number of hom(e)ologous copies originating from each of the ancestral groups. For a tetraploid individual and considering two ancestral groups, an example of allele ancestry dosage is $\left\{ 1,3\right\}$, i.e. one dose from the first group and three doses from the second group. These ancestry dosages can be modelled using a Markov Chain:

$$\begin{aligned} A_{im} \sim \mathcal{M}\mathcal{C}\left( \nu _{i},\Omega _{im}\right) \end{aligned}$$

where $$\nu _{i}$$ is the initial probability vector whose elements $$\nu _{iu}=\mathbb {P}\left( A_{i1} = u\right)$$ correspond to the probability to be in state *u*, and $$\Omega _{im}$$ is the transition probability matrix whose elements $$\Omega _{imuu'}=\mathbb {P}\left( A_{im} = u|A_{im-1} = u'\right)$$ correspond to the transition probabilities from a state $u^{\prime }$ to *u* between markers m-1 and *m*. The number of ancestry dosage states can be calculated as $$U_{iK}=\left( {\begin{array}{c}P_i + K-1\\ P_i\end{array}}\right)$$.

The initial probabilities $\nu _{iu}$ can be calculated from the admixture proportions:

$$\begin{aligned} \nu _{iu} = \left( {\begin{array}{c}P_i\\ u_{1},...,u_{k},...,u_{K}\end{array}}\right) \prod ^K_{k=1}\pi _{ik}^{u_{k}} \end{aligned}$$

where $u_{k}$ is the ancestry dosage coming from group *k* in state *u*.

The calculation of the transition probabilities $\Omega _{imuu'}$ relies on the transition probabilities $\Omega _{imkk'}$ between marker m-1 and *m* defined at the haplotype level, as proposed by Falush et al. [10]:

$$\begin{aligned} \Omega _{imkk'} = \left\{ \begin{array}{ll} 1-\rho _{mt} + \rho _{mt}\pi _{ik'} & \text {if } k'=k \\ \rho _{mt}\pi _{ik} & \text {otherwise } \\ \end{array}\right. \end{aligned}$$

where $\rho _{mt} = 1-\textrm{e}^{-d_{m}t}$ is the probability of recombination between marker *m* and m-1, $d_{m}$ is the genetic distance between markers *m* and m-1 (in Morgan), and *t* is the “number of generations” since admixture.

Each ancestry dosage state *u* is associated with a set of phased haplotype ancestries. Supposing the same example of a tetraploid state $u=\left\{ 1,3\right\}$, the set of phased haplotype ancestries is: $\left\{ \{1,2,2,2\},\{2,1,2,2\},\{2,2,1,2\},\{2,2,2,1\}\right\}$.

From an ancestry dosage state $u^{\prime }$ to *u* between marker m-1 and *m*, transitions probabilities $$\Omega _{imuu'}$$ can be calculated from transition probabilities defined at the haplotype level $$\Omega _{imkk'}$$ as following (see proof and pseudo-code in Additional file 3: Section 2):

2 $$\begin{aligned} \Omega _{imuu'} = \frac{1}{J_{u'}}\sum \limits _{j\equiv u}\sum \limits _{j'\equiv u'}\prod _{k=1}^K\prod _{k'=1}^K\left( \Omega _{imkk'}\right) ^{n_{kk'}^{jj'}} \end{aligned}$$

where $$J_{u}=\left( {\begin{array}{c}P_i\\ u_{1},...,u_{k},...,u_{K}\end{array}}\right)$$ is the number of phased ancestries for state *u*, j≡u means that the phased ancestry state *j* is compatible with the ancestry dosage state *u*, and $$n_{kk'}^{jj'}$$ corresponds to the number of chromosome copies with ancestry $k^{\prime }$ in the phased ancestry state $j^{\prime }$ that switch to ancestry *k* (or remain in ancestry $k^{\prime }$ if $k=k^{\prime }$) in state *j*.

The emission probability $\psi _{imus}=\mathbb {P}\left( S_{im}=s|A_{im}=u\right)$, i.e. the conditional probability of the observed vector of allele dosages $S_{im}$ on the ancestry dosage $A_{im}$, can be written as (see proof and pseudo-code in Additional file 3: Section 3):

$$\begin{aligned} \psi _{imus} = \sum \limits _{w\equiv u,s}\prod ^K_{k=1} \left( {\begin{array}{c}u_k\\ w_{k1},...,w_{kL_{m}}\end{array}}\right) \prod ^{L_{m}}_{l=1}\gamma _{mkl}^{w_{kl}} \end{aligned}$$

where w≡u,s corresponds to any state *w* characterising the distribution of allele dosages between ancestral groups compatible with the ancestry dosage *u* and the observed allele dosage *s*. These states can be represented as allele count matrices of dimension $K\times L_m$ whose rows sum to *u* and columns sum to *s*.

The joint log-likelihood $\mathcal {L}_2=\textrm{log}\ p_{\theta }(A,S)$ has the following expression:

$$\begin{aligned} \mathcal {L}_2= & \sum \limits ^N_{i=1}\left[ \sum \limits _{u}A_{i1u}\textrm{log}\left( \nu _{iu}\right) +\sum \limits ^M_{m=2}\sum \limits _{u}\sum \limits _{u'}A_{im-1u'}A_{imu}\textrm{log}\left( \Omega _{imuu'}\right) \right. \\ & \left. + \sum \limits ^M_{m=1}\sum \limits _{u}A_{imu}\textrm{log}\left( \psi _{imus}\right) \right] \end{aligned}$$

where $$A_{imu}=1\!\!1_{\{A_{im}=u\}}$$.

#### Inference

For the local ancestry inference, we are interested in estimating ancestry dosages at each marker, which can be obtained from the calculation of the posterior probability $\tau _{imu}=\mathbb {P}\left( A_{im}=u|S\right)$ to be in ancestry state *u* at a marker *m*. This quantity can be calculated using an EM algorithm, but a difficulty should be noted for the implementation. While the expectation step can be efficiently applied separately to each chromosome class of each target individual, the maximization step necessarily involves calculating quantities from the expectation step for all individuals, which is prohibitive for many application scenarios. We circumvented this issue by only applying the expectation step, similarly as in Corbett-Detig and Nielsen [14], using a forward-backward algorithm [46] and estimates of admixture proportions $\pi _{ik}$ and allele frequencies $\gamma _{mkl}$ obtained from the genome-wide admixture:

$$\begin{aligned} \alpha _{i1u}\propto & \nu _{iu}\psi _{i1us}\\ \alpha _{imu}\propto & \psi _{imus}\sum \limits _{u'}\alpha _{im-1u'}\Omega _{imuu'} \end{aligned}$$

where $\sum ^{U_{iK}}_{u=1}\alpha _{imu}=1$ for all *m*, and:

$$\begin{aligned} \tau _{iMu}= & \alpha _{iMu}\\ \tau _{imu}= & \alpha _{imu}\sum \limits _{u'}\Omega _{im+1u'u}\dfrac{\tau _{im+1u'}}{\sum _{u''}\alpha _{imu''}\Omega _{im+1u'u''}} \end{aligned}$$

From $\tau _{imu}$, one can obtain estimates of the local ancestry dosages using:

$$\begin{aligned} \widehat{A}_{imk} = \sum \limits _{u}\tau _{imu} u_k \end{aligned}$$

Alternatively, the most likely ancestry dosage path $\phi _{i}$ can be obtained from the Viterbi algorithm [47] using the following forward-backward recursion:

$$\begin{aligned} \beta _{i1u}= & \nu _{iu}\psi _{i1us}\\ \beta _{imu}= & \max _{u'} \ \psi _{imus}\beta _{im-1u'}\Omega _{imuu'} \\ \delta _{im-1}(u)= & \arg \max _{u'} \ \psi _{imus}\beta _{im-1u'}\Omega _{imuu'} \end{aligned}$$

and:

$$\begin{aligned} \phi _{iM}= & \arg \max _{u} \ \beta _{iMu}\\ \phi _{im}= & \delta _{im}(\phi _{im+1}) \end{aligned}$$

From $\tau _{imu}$, one can obtain estimates of the local ancestry dosages using:

3 $$\begin{aligned} \widehat{A}_{imk} = \sum \limits _{u}1\!\!1_{\{\phi _{iM}=u\}} u_k \end{aligned}$$

##### Transition probability

As calculating transition probabilities $\Omega _{imuu'}$ can be time-consuming and memory-intensive when the number of states *u* is large (Eq. 2), two approximations were considered. For instance, there are 18,564 ancestry dosage states for a ploidy of 12 and 7 ancestral groups.

The first consisted of a simplification of genetic distances between adjacent marker where genetic distances (in cM) comprised within each of these intervals were averaged:]0; 0.1], [0.1; 0.5[, [0.5; 1[, [1; 5[, [5; 10[, [10; 50[, [50; 100[, [100;+∞].

The second consisted of limiting the number of recombinations authorized to move from one ancestry state to another between adjacent markers. To this end, transitions probabilities can be reformulated as (see proof and pseudo-code in Additional file 3: Section 2):

$$\begin{aligned} \Omega _{imuu'}= & \sum \limits _{r=0}^{P_i}\underbrace{\mathbb {P}\left( R_{im}=r\right) }{}\underbrace{\dfrac{1}{J_{u'}\left( {\begin{array}{c}P_i\\ r\end{array}}\right) }\sum \limits _{j\equiv u}\sum \limits _{j'\equiv u'}\sum \limits _{s\equiv r,j,j'}\prod ^{K}_{k=1}\left( \pi _{ik}\right) ^{n^{jj'}_{sk}}}{}\\= & \sum \limits _{r=0}^{P_i}\quad C_{irm} \quad \times \quad \quad \quad \quad M_{uu'}^{ir} \end{aligned}$$

where $$R_{im}$$ is the number of recombinations between marker *m* and m-1, $$\left( {\begin{array}{c}P_i\\ r\end{array}}\right)$$ is the number of combinations of *r* recombining chromosome copies among the total set of copies $$P_i$$, *s* is a set of positions pointing to the recombining copies between phased ancestry states *j* and $j^{\prime }$, and $$n^{jj'}_{sk}$$ corresponds to the number of copies among the set *s* that switch to ancestry *k*. In the last equality, $$C_{irm}=\left( {\begin{array}{c}P_i\\ r\end{array}}\right) \left. \rho _{mt}\right. ^{r}\left( 1-\rho _{mt}\right) ^{P_i-r}$$ is a scalar and the only coefficient that depends on marker *m*. The $$M_{uu'}^{ir}$$ coefficient corresponds to the general term of a matrix $$M_{uu'}^{ir}$$ that is common to all markers and can be computed beforehand. As *r* takes values in $$\{0,...,P_i\}$$ this reduces the number of matrices to compute to $$P_i+1$$ instead of M-1. One can further reduce this number by restricting the number of allowed recombination *r* to the set $$\{0,...,\textrm{MaxRec}\}$$, with $$\textrm{MaxRec}<P_i$$.

##### Emission probability

As calculating the emission probability $\psi _{imus}$ can be time consuming, the following multinomial approximation can be considered with limited loss in precision:

4 $$\begin{aligned} \widetilde{\psi }_{iums} = \left( {\begin{array}{c}P_i\\ s_1,...,s_{L_{m}}\end{array}}\right) \prod ^{L_{j}}_{l=1}\left( \sum \limits ^K_{k=1}\frac{u_k}{P_i}\gamma _{mkl}\right) ^{s_l} \end{aligned}$$

To illustrate the differences between the two distributions, let us consider K=2 and a bi-allelic marker *m*. The probabilities corresponding to three ancestry dosages in combination with three genotype dosages are presented in Table 2.

**Table 2:** Exact ($\psi _{iums}$) and approximate ($\widetilde{\psi }_{iums}$) emission probabilities, assuming two ancestral populations, for an individual *i* with ancestry dosage $A_{im}$ and allele dosage $S_{im}$ at a bi-allelic marker *m*

$A_{im}$	$S_{im}$	Exact ($\psi _{iums}$)	Approximate ($\widetilde{\psi }_{iums}$)
(2, 0)	(2, 0)	$\gamma _{m11}^2$	$\gamma _{m11}^2$
(2, 0)	(1, 1)	$2\gamma _{m11}\gamma _{m12}$	$2\gamma _{m11}\gamma _{m12}$
(2, 0)	(0, 2)	$\gamma _{m12}^2$	$\gamma _{m12}^2$
(1, 1)	(2, 0)	$\gamma _{m11}\gamma _{m21}$	$\left( \frac{1}{2}\gamma _{m11} + \frac{1}{2}\gamma _{m21}\right) ^2$
(1, 1)	(1, 1)	$\gamma _{m11}\gamma _{m22}$ + $\gamma _{m21}\gamma _{m12}$	$2\left( \frac{1}{2}\gamma _{m11} + \frac{1}{2}\gamma _{m21}\right) \left( \frac{1}{2}\gamma _{m12} + \frac{1}{2}\gamma _{m22}\right)$
(1, 1)	(0, 2)	$\gamma _{m12}\gamma _{m22}$	$\left( \frac{1}{2}\gamma _{m12} + \frac{1}{2}\gamma _{m22}\right) ^2$
(0, 2)	(2, 0)	$\gamma _{m21}^2$	$\gamma _{m21}^2$
(0, 2)	(1, 1)	$2\gamma _{m21}\gamma _{m22}$	$2\gamma _{m21}\gamma _{m22}$
(0, 2)	(0, 2)	$\gamma _{m22}^2$	$\gamma _{m22}^2$

### Simulation

Admixed populations were simulated to study the precision of inferring admixture proportions, ancestral allele frequencies, and local ancestry proportions in various scenarios. Genotypic data were simulated according to the local admixture model and by modulating the following parameters: number of ancestral groups, number of individuals, ploidy level, number of markers, number of alleles per marker, and number of generations since admixture. Admixture proportions and ancestral allele frequencies were sampled from a Dirichlet distribution with a concentration parameter equal to 1 and $\frac{1}{L_m}$, respectively. The choice of this parameter value for ancestral allele frequencies aimed to reflect that, as the number of alleles increases, they tend to be more specific to ancestral groups. Marker distances were sampled from the 80K regions distributed across 10 chromosomes used in the *Saccharum* dataset, by assuming that each chromosome was 100 cM long and that the genetic distance between adjacent markers was proportional to their physical distance. All modulated simulation parameters are summarized in Additional file 4: Table S1.

Sequencing data were simulated by sampling allele read depths from a multinomial distribution conditional on the true allele dosages, assuming an error rate of 1%. Different genotyping approaches were compared to obtain allele dosages from sequencing data: either inferring the most likely allele dosage from the same multinomial distribution (genotype call), or calculating allele depth ratios (allele depth to the total read depth) multiplied by the ploidy.

To investigate the impact of strongly related individuals in the reference panel, we simulated related pairs of individuals, where the two individuals were first simulated independently with identical admixture proportions. Relatedness was then generated by assigning the allele dosages of the first individual to the second individual, with a proportion of markers corresponding to the target level of relatedness.

When compared to other genome-wide admixture methods, the proxy based on allele depth ratios was used for AdmixPoly. For Entropy [13], genotype likelihoods were calculated from the above-mentioned multinomial distribution, and formatted in the required “mpgl” format. As Structure [6, 10] only accepts discrete allele dosages, a genotype call was used in Scenario 1 with bi-allelic markers. In Scenario 2, with 10 alleles per marker, the computational time required to perform a genotype call was prohibitive. Instead, allele dosages were sampled from a multinomial distribution with probabilities equal to allele depth ratios.

As a linkage map might not be available for the species under study, we evaluated the impact of using a physical distance proxy in place of the true genetic distances during the inference step. To mimic the non-linear relationship between true genetic distances and a physical distance proxy, distance proxies were calculated from simulated genetic distances using the sigmoid function $\dfrac{100}{\left( 1+e^{-\lambda (d-50)}\right) }$ where *d* is the true genetic distance in cM and λ is a parameter that increases the non-linearity of the relationship between the genetic distance and the proxy.

For the local admixture comparison between AdmixPoly and Ancestry HMM, the true allele dosages were considered for both approaches. As they both require estimates of admixture proportions and ancestral allele frequencies, they were first estimated using the genome-wide admixture approach of AdmixPoly. The input format of Ancestry HMM requires allele read counts for each ancestral group in a reference panel. Those were generated by sampling 100 reads in each ancestral group using simulated ancestral allele frequencies. A single admixture pulse dated to one generation since admixture was considered for Ancestry HMM, to be in accordance with the simulated *t* value.

### R-package

All methods were implemented in the new R-package AdmixPoly, which relies on Rcpp to interface R with optimized C++ code. Linear algebra operations and matrix computations are performed using the Armadillo library available from RcppArmadillo. Parallelization is implemented via OpenMP, enabling multi-threaded execution on modern multi-core processors.

### Computational Resources

All benchmarks were performed on the MESO@LR HPC cluster, using nodes equipped with dual Intel Xeon E5-2680 v4 (2.4 GHz, 28 cores each) and 128 GB RAM per node. Jobs were submitted via the Slurm scheduler, requesting a single core per job. For the comparison of AdmixPoly with Structure and Entropy, additional analyses were run with 3 and 10 cores per job. Computing time was measured using the R “system.time()” function, and memory usage (maximum resident set size) was recorded using the “/usr/bin/time -v” command.

### Sugarcane data

A panel of 390 accessions of the genus *Saccharum* and related genera was considered in this study [26]. It consisted of 43 *S. robustum*, 6 *S. edule*, 72 *S. officinarum*, 135 *S. spontaneum*, 16 *S. barberi*, 5 *S. sinense*, 96 interspecific hybrids including modern cultivars and base-broadening germplasm, 8 *S. maximum* and 8 representatives of close genera (*Tripidium*, *Miscanthus*, *Narenga,* and *Erianthus fulvus*).

All accessions were sequenced for their whole genome at high depth using Illumina paired-end sequencing. The genotyping consisted of characterizing accessions for small haplotypes within 86,681 regions of 80 bp distributed over the genome using the HaploCharmer workflow [27]. To reduce the computational time required for aligning high-depth sequencing data, the reads corresponding to all regions were recovered through *in silico* capture using probe sequences as targets with Mirabait [48]. The haplotype calling was performed using a monoploid reference sequence derived from the polyploid reference sequence of R570 [23] by choosing the longest scaffolds from each hom(e)ology group, but excluding scaffolds representing rearranged chromosomes [26]. The following parameters were used for HaploCharmer (Remove_duplicate: True, Mapping_quality: 20, VCF_filter: “–min_gt_depth 10 –max_gt_depth 1000 –min_hap_depth 3 –min_hap_freq 0.04”, VCF_to_HapPresAbs: “–max_abs_freq 0.01 –min_pres_depth 3 –min_pres_freq 0.04”).

Raw genotypic data were filtered for haplotypic regions with more than 50% missing values and with more than 100 haplotypes, as they likely resulted from reads from different loci mapping to single locus on the reference. After filtering, a total of 83,890 haplotypic regions were conserved, which gathered 3,651,114 haplotypes, with an average of 43.52 haplotypes per haplotypic region (Additional file 1: Fig. S6). As local admixture requires genetic distances between adjacent markers, a proxy was considered by assuming that each chromosome was 100 cM long and that the genetic distance between adjacent markers was proportional to their physical distance.

The monoploid reference used for the mapping of reads represented the ancestral genome structure observed in *S. officinarum*. For local admixtures focusing on the *S. spontaneum* contributions, the genomic regions were projected on another monoploid reference sequence in which chromosomes Chr5A, Chr6A, Chr7A, Chr8A, Chr9A, and Chr10A were replaced by rearranged chromosomes Chr5_9A, Chr6_9A, Chr7_10A, and Chr8_10A. The projection of genomic coordinates was done by mapping probes, which were initially used to define the set of 86,681 regions [27], on this new monoploid reference using BWA-mem [49].

In addition, a genetic map obtained from the self-progeny of the modern cultivar R570 was used to validate the local admixture of R570. It was built using single-dose haplotypes obtained from the same 80K haplotypic regions and physically mapped on the R570 monoploid assembly, as described in Rio et al. [27]. Single-dose haplotypes also genotyped in the WGS dataset were characterized as private from a group *k* if their estimated ancestral allele frequency was at least 0.01 and 100 times higher than ancestral allele frequencies in other groups. Note that the genetic distances obtained from this map were highly inflated due to genotyping errors, which explains why they were not used for the local admixture inference.

### Sugarcane admixture analysis

The genome-wide admixture inference was applied following the multi-step procedure described in Garsmeur et al. [26]. In the first step, model parameters were estimated considering five ancestral groups, excluding hybrids (i.e. *S. barberi*, *S. sinense,* and modern cultivars). Given the limited contribution of the unknown ancestor to the population of ancestral accessions, with no pure representative, it requires some prior information to be accounted for. In the second step, admixture proportions of accessions with substantial contribution from this unknown contributor (i.e. NC100, NC24, NC29, NC30, and RaiateaX) were set manually based on cytogenetic observations (Additional file 1: Fig. S10). In the third step, ancestral allele frequencies were re-estimated considering fixed admixture proportions, including those corresponding to the unknown ancestral group. In the last step, estimates of ancestral allele frequencies were treated as fixed in a new model used to estimate the admixture proportions of hybrids.

The local admixture inference was applied using the approximate emission probability distribution and the approximate transition probabilities by considering simplified genetic distances between adjacent markers.

## Supplementary information


Additional file 1. Supplementary figures. Fig. S1. RMSE of genome-wide admixture inference parameters according to simulation parameters. Fig. S2. BIC of the genome-wide admixture inference according to the simulated and tested number of groups. Fig. S3. RMSE of genome-wide admixture inference parameters according to the relatedness between 300 pairs of simulated individuals. Fig. S4. RMSE of admixture inference parameters according to the sequencing depth. Fig. S5. RMSE of local admixture ancestry proportions according to simulation parameters. Fig. S6. RMSE of local admixture ancestry proportions according the simulated and tested time since admixture. Fig. S7. RMSE of local admixture ancestry proportions according to the genetic map used. Fig. S8. Comparison of AdmixPoly and Ancestry_HMM. Fig. S9. Distribution of number of haplotypes per haplotypic region. Fig. S10. Cytogenetic observations of metaphase chromosomes. Fig. S11. Evaluation of R570 local admixture by comparison with a genetic mapping approach. Fig. S12. Average *S. officinarum* and *S. spontaneum* contributions over the genome of all modern cultivars Fig. S13. Shared contributions from the Continental Asia *S. spontaneum* subgroup between modern and former interspecific hybrid cultivars Fig. S14. Shared contributions from the Southeast Asia *S. spontaneum* subgroup between modern and former interspecific hybrid cultivars Fig. S15. Shared contributions from the Northeast Asia *S. spontaneum* subgroup between modern and former interspecific hybrid cultivars.Additional file 2. Local admixture of sugarcane cultivars.Additional file 3. Derivations and pseudo-codes. Section 1: Expectation-maximization algorithm. Section 2: Transition probabilities. Section 3: Emission probabilities.Additional file 4. Supplementary table. Table S1. Summary of population parameters.

## Data Availability

The WGS data of 290 accessions of the genus *Saccharum* and related genera, as described in Garsmeur et al. [26], are available from NCBI with BioProject PRJNA1228676 [50]. In addition, WGS data of 10 S. robustum, 90 S. spontaneum, S. officinarum LA Purple and R570 cultivar are available from NCBI BioProject PRJNA1209834 [51], PRJNA721787 [52], PRJNA333303 [53] and PRJNA456890 [54], respectively. The reference genome assembly of R570 sugarcane cultivar (v 2.1) [23] is available from https://phytozome-next.jgi.doe.gov/info/SofficinarumxspontaneumR570v21. The raw haplotyping data in haplotype presence-absence (HPA) format, the ploidy levels of accessions, and the genome-wide and local admixture results are available from the CIRAD Dataverse [55]: https://doi.org/10.18167/DVN1/YJLW96. The AdmixPoly R-package is available from the CRAN [56], the CIRAD Gitlab [57] https://gitlab.cirad.fr/agap/seg/admixpoly and Zenodo [58] under the GPL-3.0 license.

## References

[1] Sankararaman S, Mallick S, Dannemann M, Prüfer K, Kelso J, Pääbo S, et al. The genomic landscape of Neanderthal ancestry in present-day humans. Nature. 2014;507(7492):354–7. 10.1038/nature12961. 24476815 PMC4072735

[2] Decker JE, McKay SD, Rolf MM, Kim J, Molina Alcalá A, Sonstegard TS, et al. Worldwide Patterns of Ancestry, Divergence, and Admixture in Domesticated Cattle. PLoS Genet. 2014;10(3):e1004254. 10.1371/journal.pgen.1004254. 24675901 PMC3967955

[3] vonHoldt BM, Pollinger JP, Lohmueller KE, Han E, Parker HG, Quignon P, et al. Genome-wide SNP and haplotype analyses reveal a rich history underlying dog domestication. Nature. 2010;464(7290):898–902. 10.1038/nature08837. 20237475 PMC3494089

[4] Santos JD, Chebotarov D, McNally KL, Bartholomé J, Droc G, Billot C, et al. Fine Scale Genomic Signals of Admixture and Alien Introgression among Asian Rice Landraces. Genome Biol Evol. 2019;11(5):1358–73. 10.1093/gbe/evz084. 31002105 PMC6499253

[5] Martin G, Cottin A, Baurens F, Labadie K, Hervouet C, Salmon F, et al. Interspecific introgression patterns reveal the origins of worldwide cultivated bananas in New Guinea. Plant J. 2023;113(4):802–18. 10.1111/tpj.16086. 36575919

[6] Pritchard JK, Stephens M, Donnelly P. Inference of population structure using multilocus genotype data. Genetics. 2000;155(2):945–59. 10.1093/genetics/155.2.945. 10835412 PMC1461096

[7] Raj A, Stephens M, Pritchard JK. FastSTRUCTURE: variational inference of population structure in large SNP data sets. Genetics. 2014;197(2):573–89. 10.1534/genetics.114.164350. 24700103 PMC4063916

[8] Tang H, Peng J, Wang P, Risch NJ. Estimation of individual admixture: analytical and study design considerations. Genet Epidemiol. 2005;28(4):289–301. 10.1002/gepi.20064. 15712363

[9] Alexander DH, Novembre J, Lange K. Fast model-based estimation of ancestry in unrelated individuals. Genome Res. 2009;19(9):1655–64. 10.1101/gr.094052.109. 19648217 PMC2752134

[10] Falush D, Stephens M, Pritchard JK. Inference of population structure using multilocus genotype data: linked loci and correlated allele frequencies. Genetics. 2003;164(4):1567–87. 10.1093/genetics/164.4.1567. 12930761 PMC1462648

[11] Tang H, Coram M, Wang P, Zhu X, Risch N. Reconstructing genetic ancestry blocks in admixed individuals. Am J Hum Genet. 2006;79(1):1–12. 10.1086/504302. 16773560 PMC1474129

[12] Stift M, Kolář F, Meirmans PG. Structure is more robust than other clustering methods in simulated mixed-ploidy populations. Heredity. 2019;123(4):429–41. 10.1038/s41437-019-0247-6. 31285566 PMC6781132

[13] Shastry V, Adams PE, Lindtke D, Mandeville EG, Parchman TL, Gompert Z, et al. Model-based genotype and ancestry estimation for potential hybrids with mixed-ploidy. Mol Ecol Resour. 2021;21(5):1434–51. 10.1111/1755-0998.13330. 33482035

[14] Corbett-Detig R, Nielsen R. A hidden Markov model approach for simultaneously estimating local ancestry and admixture time using next generation sequence data in samples of arbitrary ploidy. PLoS Genet. 2017;13(1):e1006529. 10.1371/journal.pgen.1006529. 28045893 PMC5242547

[15] Medina P, Thornlow B, Nielsen R, Corbett-Detig R. Estimating the timing of multiple admixture pulses during local ancestry inference. Genetics. 2018;210(3):1089–107. 10.1534/genetics.118.301411. 30206187 PMC6218234

[16] Martin SH. A model-free method for genealogical inference without phasing and its application for topology weighting. Genetics. 2026. 10.1093/genetics/iyaf181. 40916405 PMC12774849

[17] Piperidis N, D’Hont A. Sugarcane genome architecture decrypted with chromosome-specific oligo probes. Plant J. 2020;103(6):2039–51. 10.1111/tpj.14881. 32537783

[18] D’Hont A, Grivet L, Feldmann P, Glaszmann JC, Rao S, Berding N. Characterisation of the double genome structure of modern sugarcane cultivars (*Saccharum* spp.) by molecular cytogenetics. Mol Gen Genet. 1996;250(4):405–13. 10.1007/bf02174028. 8602157

[19] Piperidis G, Piperidis N, D’Hont A. Molecular cytogenetic investigation of chromosome composition and transmission in sugarcane. Mol Genet Genomics. 2010;284(1):65–73. 10.1007/s00438-010-0546-3. 20532565

[20] Zhang J, Zhang X, Tang H, Zhang Q, Hua X, Ma X, et al. Allele-defined genome of the autopolyploid sugarcane *Saccharum spontaneum* L. Nat Genet. 2018;50(11):1565–73. 10.1038/s41588-018-0237-2. 30297971

[21] Zhang Q, Qi Y, Pan H, Tang H, Wang G, Hua X, et al. Genomic insights into the recent chromosome reduction of autopolyploid sugarcane *Saccharum spontaneum*. Nat Genet. 2022;54(6):885–96. 10.1038/s41588-022-01084-1. 35654976

[22] Garsmeur O, Droc G, Antonise R, Grimwood J, Potier B, Aitken K, et al. A mosaic monoploid reference sequence for the highly complex genome of sugarcane. Nat Commun. 2018. 10.1038/s41467-018-05051-5. 29980662 PMC6035169

[23] Healey AL, Garsmeur O, Lovell JT, Shengquiang S, Sreedasyam A, Jenkins J, et al. The complex polyploid genome architecture of sugarcane. Nature. 2024;628(8009):804–10. 10.1038/s41586-024-07231-4. 38538783 PMC11041754

[24] Bao Y, Zhang Q, Huang J, Zhang S, Yao W, Yu Z, et al. A chromosomal-scale genome assembly of modern cultivated hybrid sugarcane provides insights into origination and evolution. Nat Commun. 2024. 10.1038/s41467-024-47390-6. 38589412 PMC11001919

[25] Zhang J, Qi Y, Hua X, Wang Y, Wang B, Qi Y, et al. The highly allo-autopolyploid modern sugarcane genome and very recent allopolyploidization in *Saccharum*. Nat Genet. 2025;57(1):242–53. 10.1038/s41588-024-02033-w. 39753769

[26] Garsmeur O, Rio S, Pompidor N, Lipzen A, Hervouet C, Durand T, et al. The genomic footprints of wild *Saccharum* species trace domestication, diversification, and modern breeding of sugarcane. Cell. 2025. 10.1016/j.cell.2025.09.017. 41092921

[27] Rio S, Abdallah S, Durand T, D’Hont A, Garsmeur O. HaploCharmer: a Snakemake workflow for read-scale haplotype calling adapted to polyploids. Peer Community J. 2025. 10.24072/pcjournal.631.

[28] Kolář F. Tracing evolutionary history and admixture in mixed-ploidy systems. Mol Ecol Resour. 2021;21(5):1413–5. 10.1111/1755-0998.13390. 33749076

[29] Meirmans PG, Liu S, van Tienderen PH. The analysis of polyploid genetic data. J Hered. 2018;109(3):283–96. 10.1093/jhered/esy006. 29385510

[30] Yang X, Song J, Todd J, Peng Z, Paudel D, Luo Z, et al. Target enrichment sequencing of 307 germplasm accessions identified ancestry of ancient and modern hybrids and signatures of adaptation and selection in sugarcane (*Saccharum* spp.), a ‘sweet’ crop with ‘bitter’ genomes. Plant Biotechnol J. 2018;17(2):488–98. 10.1111/pbi.12992. 30051590 PMC6335080

[31] Evanno G, Regnaut S, Goudet J. Detecting the number of clusters of individuals using the software STRUCTURE: a simulation study. Mol Ecol. 2005;14(8):2611–20. 10.1111/j.1365-294x.2005.02553.x. 15969739

[32] Huang Y, Chen H, Han J, Zhang Y, Ma S, Yu G, et al. Species-specific abundant retrotransposons elucidate the genomic composition of modern sugarcane cultivars. Chromosoma. 2019;129(1):45–55. 10.1007/s00412-019-00729-1. 31848693

[33] Grivet L, D’Hont A, Roques D, Feldmann P, Lanaud C, Glaszmann JC. RFLP mapping in cultivated sugarcane (*Saccharum* spp.): genome organization in a highly polyploid and aneuploid interspecific hybrid. Genetics. 1996;142(3):987–1000. 10.1093/genetics/142.3.987. 8849904 PMC1207035

[34] Hoarau JY, Offmann B, D’Hont A, Risterucci AM, Roques D, Glaszmann JC, et al. Genetic dissection of a modern sugarcane cultivar (*Saccharum* spp.). I. Genome mapping with AFLP markers. Theor Appl Genet. 2001;103(1):84–97. 10.1007/s001220000390.

[35] Aitken K, Li J, Piperidis G, Qing C, Yuanhong F, Jackson P. Worldwide genetic diversity of the wild species *Saccharum spontaneum* and level of diversity captured within sugarcane breeding programs. Crop Sci. 2018;58(1):218–29. 10.2135/cropsci2017.06.0339.

[36] Jeswiet J. The history of sugar cane selection work in Java. Proc Int Soc Sugar Cane Technol. 1927;2:115–22.

[37] Stevenson GC. Genetics and Breeding of Sugar Cane. Tropical science series. Longmans; 1965.

[38] Arcenaux G. Cultivated sugarcanes of the world and their botanical derivation. Proc Int Soc Sugar Cane Technol. 1965;12:844–54.

[39] Umeda M, Sakaigaichi T, Tanaka M, Tarumoto Y, Adachi K, Hattori T, et al. Detection of a major QTL related to smut disease resistance inherited from a Japanese wild sugarcane using GRAS-Di technology. Breed Sci. 2021;71(3):365–74. 10.1270/jsbbs.20137. 34776743 PMC8573549

[40] Kennedy AJ. In: Genetic base-broadening in the West Indies sugar cane breeding programme by the incorporation of wild species. International Plant Genetic Resources Institute (IPGRI) Food and Agriculture Organization of the United Nations (FAO) CABI. 2000. pp. 283–294. 10.1079/9780851994116.0283.

[41] Santchurn D, Badaloo MGH, Zhou M, Labuschagne MT. Contribution of sugarcane crop wild relatives in the creation of improved varieties in Mauritius. Plant Genet Resour Charact Util. 2019;17(2):151–63. 10.1017/s1479262118000552.

[42] Roach BT. Nobilization of sugarcane. Proc Int Soc Sugar Cane Technol. 1972;14:206–16.

[43] Dempster AP, Laird NM, Rubin DB. Maximum likelihood from incomplete data via the EM algorithm. J Roy Stat Soc: Ser B (Methodol). 2018;39(1):1–22. 10.1111/j.2517-6161.1977.tb01600.x.

[44] De Walsche A, Gauthier F, Boissot N, Charcosset A, Mary-Huard T. Large-scale composite hypothesis testing procedure for omics data analyses. NAR Genom Bioinform. 2025. 10.1093/nargab/lqaf118. 40918066 PMC12412788

[45] Varadhan R, Roland C. Simple and globally convergent methods for accelerating the convergence of any EM algorithm. Scand J Stat. 2008;35(2):335–53. 10.1111/j.1467-9469.2007.00585.x.

[46] Rabiner LR. A tutorial on hidden Markov models and selected applications in speech recognition. Proc IEEE. 1989;77(2):257–86. 10.1109/5.18626.

[47] Viterbi A. Error bounds for convolutional codes and an asymptotically optimum decoding algorithm. IEEE Trans Inf Theory. 1967;13(2):260–9. 10.1109/tit.1967.1054010.

[48] Chevreux B, Pfisterer T, Drescher B, Driesel AJ, Müller WEG, Wetter T, et al. Using the miraEST Assembler for Reliable and Automated mRNA Transcript Assembly and SNP Detection in Sequenced ESTs. Genome Res. 2004;14(6):1147–59. 10.1101/gr.1917404. 15140833 PMC419793

[49] Li H. Aligning sequence reads, clone sequences and assembly contigs with BWA-MEM. 2013. arXiv:1303.3997.

[50] NCBI Sequence Read Archive. *Saccharum* representative and close genera sequencing. 2025. BioProject: PRJNA1228676. https://www.ncbi.nlm.nih.gov/bioproject/PRJNA1228676. Accessed June 2026.

[51] NCBI Sequence Read Archive. *Saccharum robusum* diversity sequencing. 2025. BioProject: PRJNA1209834. https://www.ncbi.nlm.nih.gov/bioproject/PRJNA1209834. Accessed June 2026.

[52] NCBI Sequence Read Archive. *Saccharum spontaneum* isolate: Np-X (wild sugarcane). 2022. BioProject: PRJNA721787. https://www.ncbi.nlm.nih.gov/bioproject/PRJNA721787. Accessed June 2026.

[53] NCBI Sequence Read Archive. *Saccharum officinarum* strain:LA Purple | cultivar:LA Purple (sugarcane). 2016. BioProject: PRJNA333303. https://www.ncbi.nlm.nih.gov/bioproject/PRJNA333303. Accessed June 2026.

[54] NCBI Sequence Read Archive. *Saccharum hybrid* cultivar R570 cultivar:R570. 2023. BioProject: PRJNA456890. https://www.ncbi.nlm.nih.gov/bioproject/PRJNA456890. Accessed June 2026.

[55] Rio S, Garsmeur O, D’Hont A. Haplotyping and admixture results for a diversity panel of 390 *Saccharum* accessions and related genera. 2026. CIRAD Dataverse. 10.18167/DVN1/YJLW96.

[56] Rio S, Mary-Huard T, Gauthier F. AdmixPoly: Global and Local Admixture Inference in Polyploids. The R Foundation; 2026. 10.32614/cran.package.admixpoly.

[57] Rio S, Gauthier F, Mary-Huard T. AdmixPoly v1.0.0. GitLab; 2026. https://gitlab.cirad.fr/agap/seg/admixpoly. Accessed June 2026.

[58] Rio S, Gauthier F, Mary-Huard T. AdmixPoly v1.0.0. Zenodo; 2026. 10.5281/zenodo.20442536.

