# Supplementary material for: Deciphering the mosaic genome of sugarcane cultivars through polyploid admixture inference with AdmixPoly
Source: Genome Biol. 2026 Jun 19;27:280. doi: 10.1186/s13059-026-04162-3 (PMC13531838; doi:10.1186/s13059-026-04162-3)
Supplement: Supplementary file 1 [file 13059_2026_4162_MOESM1_ESM.pdf]

Additional file 1: Supplementary Figures

Deciphering the Mosaic Genome of Sugarcane Cultivars through Polyploid Admixture Inference with AdmixPoly

Simon Rio[1,2], Franck Gauthier[3], Olivier Garsmeur[1,2], George Piperidis[4], Jean-Yves Hoarau[5,2], German Serino[6], Raul Castillo Torres[7], Shailesh Vinay Joshi[8,9], Yoshifumi Terajima[10], Jershon Lopez-Gerena[11], María Francisca Perera[12], Andrew Stoute[13], Goolam Badaloo[14], Dongliang Huang[15], Kerrie Barry[16], Jeremy Schmutz[16,17], Tristan Mary-Huard[3,18], and Angélique D'Hont[1,2]

[1]CIRAD, UMR AGAP Institut, F-34398 Montpellier, France
[2]UMR AGAP Institut, Univ Montpellier, CIRAD, INRAE, Institut Agro, F-34398, Montpellier, France
[3]Université Paris-Saclay, INRAE, CNRS, AgroParisTech, UMR GQE-Le Moulon, 91190, Gif-sur-Yvette, France
[4]Sugar Research Australia, Brisbane QLD 4000
[5]eRcane, Sainte-Clotilde, La Réunion, France
[6]Chacra Experimental Agrícola Santa Rosa, Camino Vecinal No. 8 Km 6, Colonia Santa Rosa, 4531 Salta, Argentina
[7]Centro de Investigación de la Caña de Azúcar del Ecuador - CINCAE, Guayaquil, Ecuador
[8]South African Sugarcane Research Institute, Private Bag X02, Mount Edgecombe, Durban, 4300, KwaZulu-Natal, South Africa
[9]School of Agriculture and Science, College of Agriculture, Engineering and Science, University of KwaZulu-Natal, South Africa
[10]Tropical Agriculture Research Front, JIRCAS, 1091-1 Maezato-Kawarabaru, Ishigaki, Okinawa, 907-0002, Japan
[11]Centro de Investigación de la Caña de Azúcar de Colombia (CENICAÑA), Cali, Colombia
[12]Instituto de Tecnología Agroindustrial del Noroeste Argentino (ITANOA), Estación Experimental Agroindustrial Obispo Colombres (EEAOC) - Consejo Nacional de Investigaciones Científicas y Técnicas (CONICET), Las Talitas, Tucumán, Argentina
[13]West Indies Central Sugar Cane Breeding Station, Groves, St. George, Barbados
[14]Mauritius Sugarcane Industry Research Institute, Mauritius Cane Industry Authority, 1 Moka Road, Réduit, Mauritius
[15]Sugarcane Research Institute, Guangxi Academy of Agricultural Sciences, Nanning, China
[16]DOE Joint Genome Institute, Lawrence Berkeley National Laboratory, Berkeley, CA, 94720, USA
[17]HudsonAlpha Institute for Biotechnology, 601 Genome Way Northwest, Huntsville, AL 35806, USA
[18]Université Paris-Saclay, AgroParisTech, INRAE, UMR MIA-Paris, 91120, Palaiseau, France

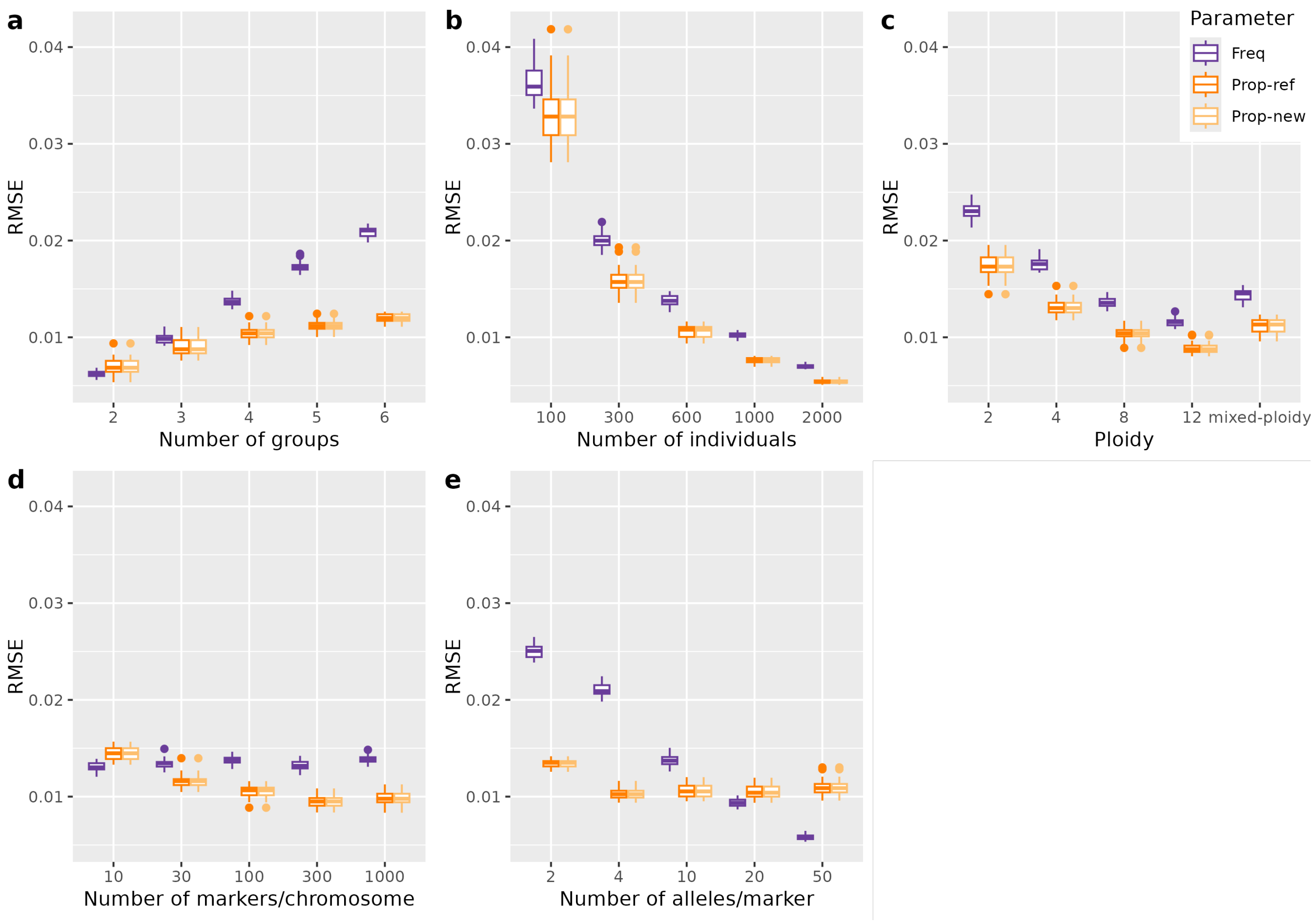

Fig S1: Root mean square error (RMSE) of genome-wide admixture inference parameters according to simulation parameters: a) number of groups, b) number of individuals, c) ploidy level, d) number of markers per chromosome, and e) number of alleles per marker. Genome-wide admixture inference parameters include ancestral allele frequencies (Freq) and admixture proportions (Prop-ref) estimated on a reference population. In a second step, the same ancestral allele frequencies were used to estimate admixture proportions (Prop-new) of a new set of individuals. Each of these parameters are modulated based on a reference simulation scenario of four groups, 600 individuals, a ploidy of eight, 1,000 markers distributed over 10 chromosomes, and 10 alleles per marker. The "mixed-ploidy" scenario consisted of randomly sampling the ploidy of individuals (i.e. 2, 4, 6, 8, 10 or 12).

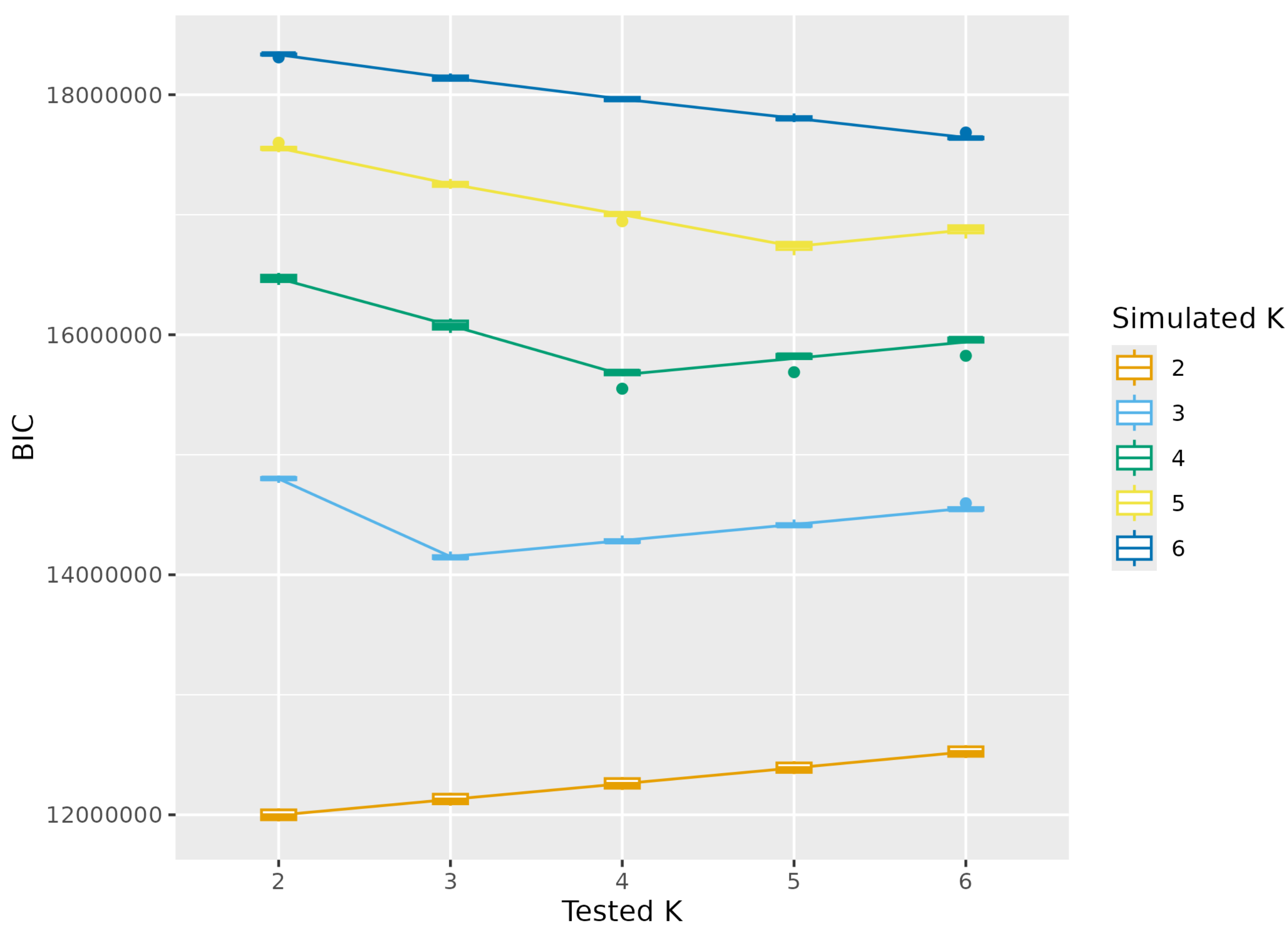

Fig S2: Bayesian information criterion (BIC) of the genome-wide admixture inference according to the simulated (Simulated K) and tested number of groups (Tested K). Other simulation parameters are: 600 individuals, a ploidy of eight, 1,000 markers distributed over 10 chromosomes, and 10 alleles per marker.

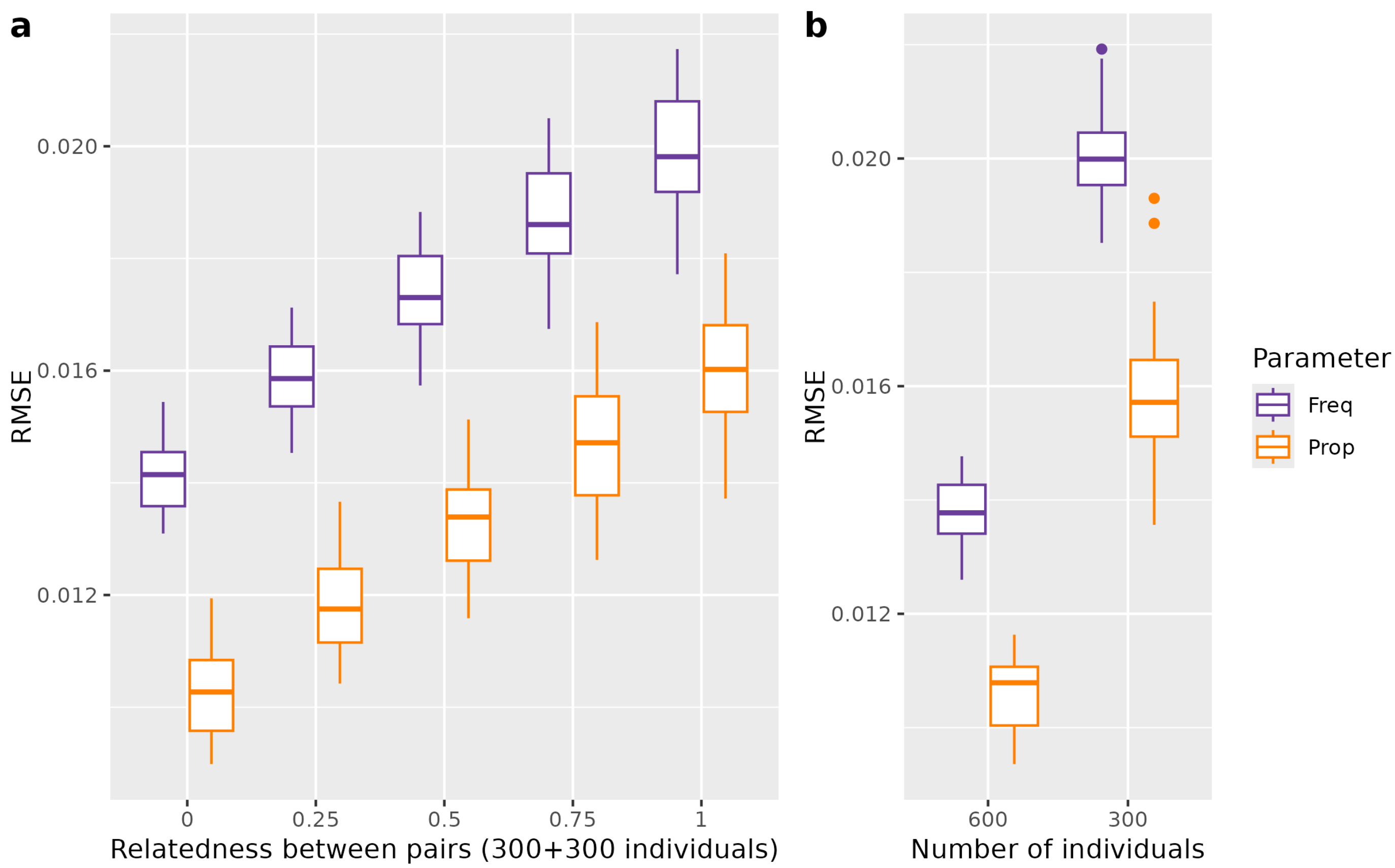

Fig S3: a) Root mean square error (RMSE) of genome-wide admixture inference parameters according to the relatedness between 300 pairs of simulated individuals (600 in total): 0, 0.25, 0.5 , 0.75 and 1. b) RMSE according to the number of unrelated individuals (300 or 600). Other simulation parameter consist of four groups, a ploidy of eight, 1,000 markers distributed over 10 chromosomes, and 10 alleles per marker.

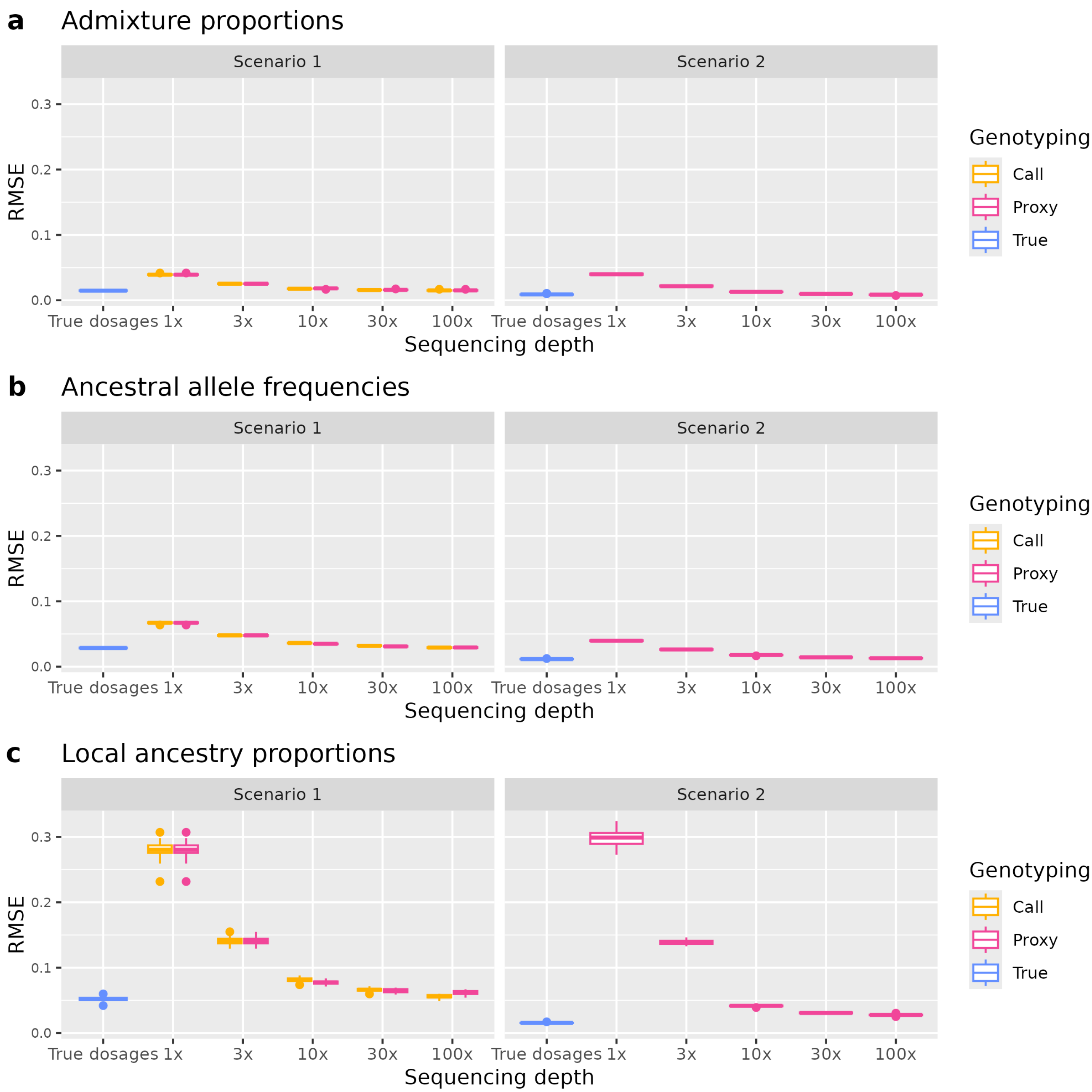

Fig S4: Root mean square error (RMSE) of admixture inference parameters according to the sequencing depth at each marker: 1x, 3x, 10x, 30x and 100x, along with the true allele dosages. Two contrasted scenarios were considered: Scenario 1 with a ploidy of 6 and 2 alleles per marker, and Scenario 2 with a ploidy of 12 and 10 alleles per marker. Admixture inference parameters include a) admixture proportions, b) ancestral allele frequencies and c) local ancestry proportions. When considering simulated sequencing data, the most likely allele dosage was inferred (Call) or a simple proxy was calculated using allele depth ratios (allele depth to the total read depth) multiplied by the ploidy (Proxy). For scenario 2, only "Proxy" was applied due to prohibitive computing time.

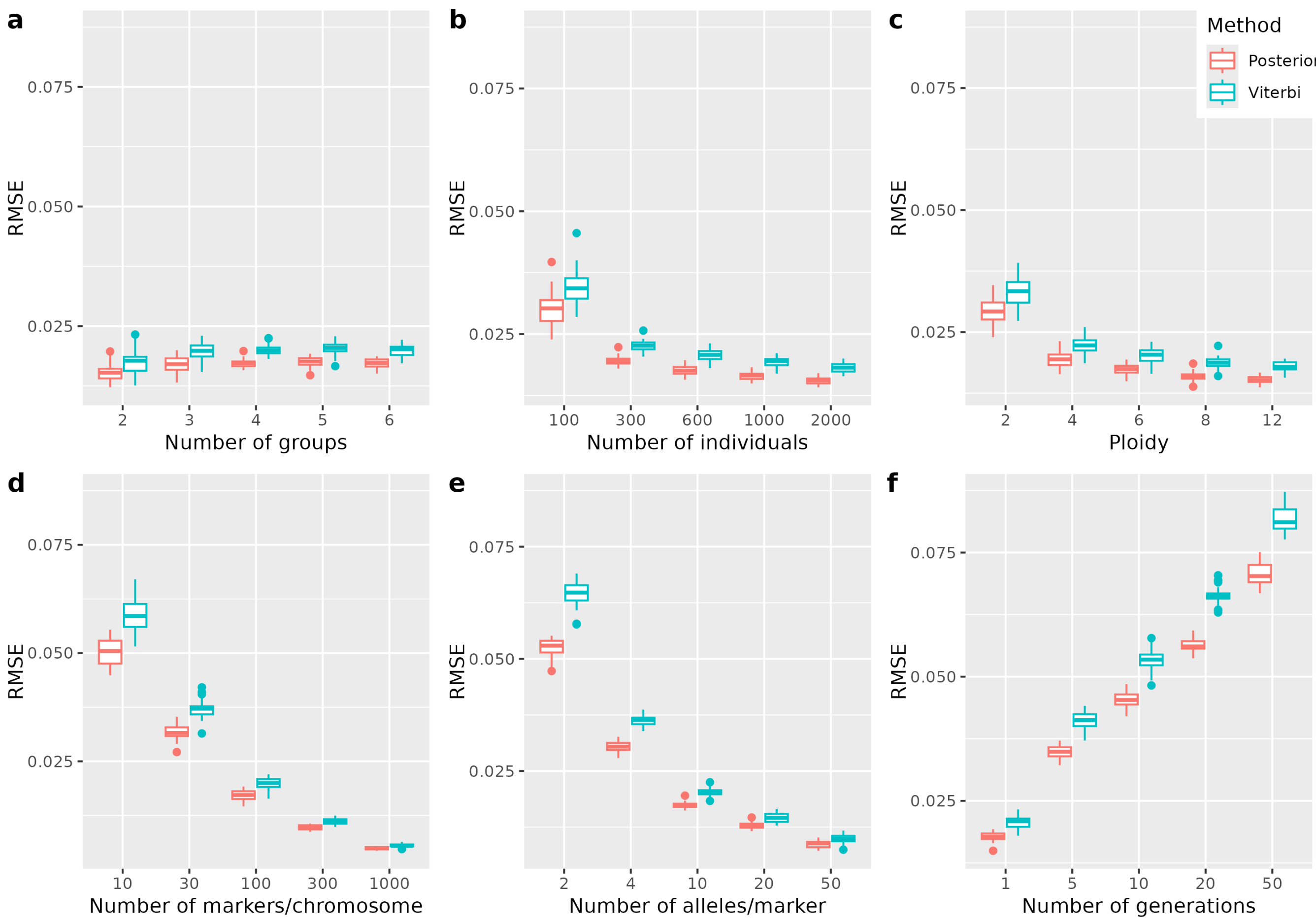

Fig S5: Root mean square error (RMSE) of local admixture ancestry proportions obtained from posterior or Viterbi probabilities according to simulation parameters: a) number of groups, b) number of individuals, c) ploidy level, d) number of markers per chromosome, e) number of alleles per marker, and f) number of generations since admixture. Each of these parameters are modulated based on a reference simulation scenario of four groups, 600 individuals, a ploidy of six, 1,000 markers distributed over 10 chromosomes, 10 alleles per marker, and one generation since admixture. No approximation of transition or emission probability was used for the inference.

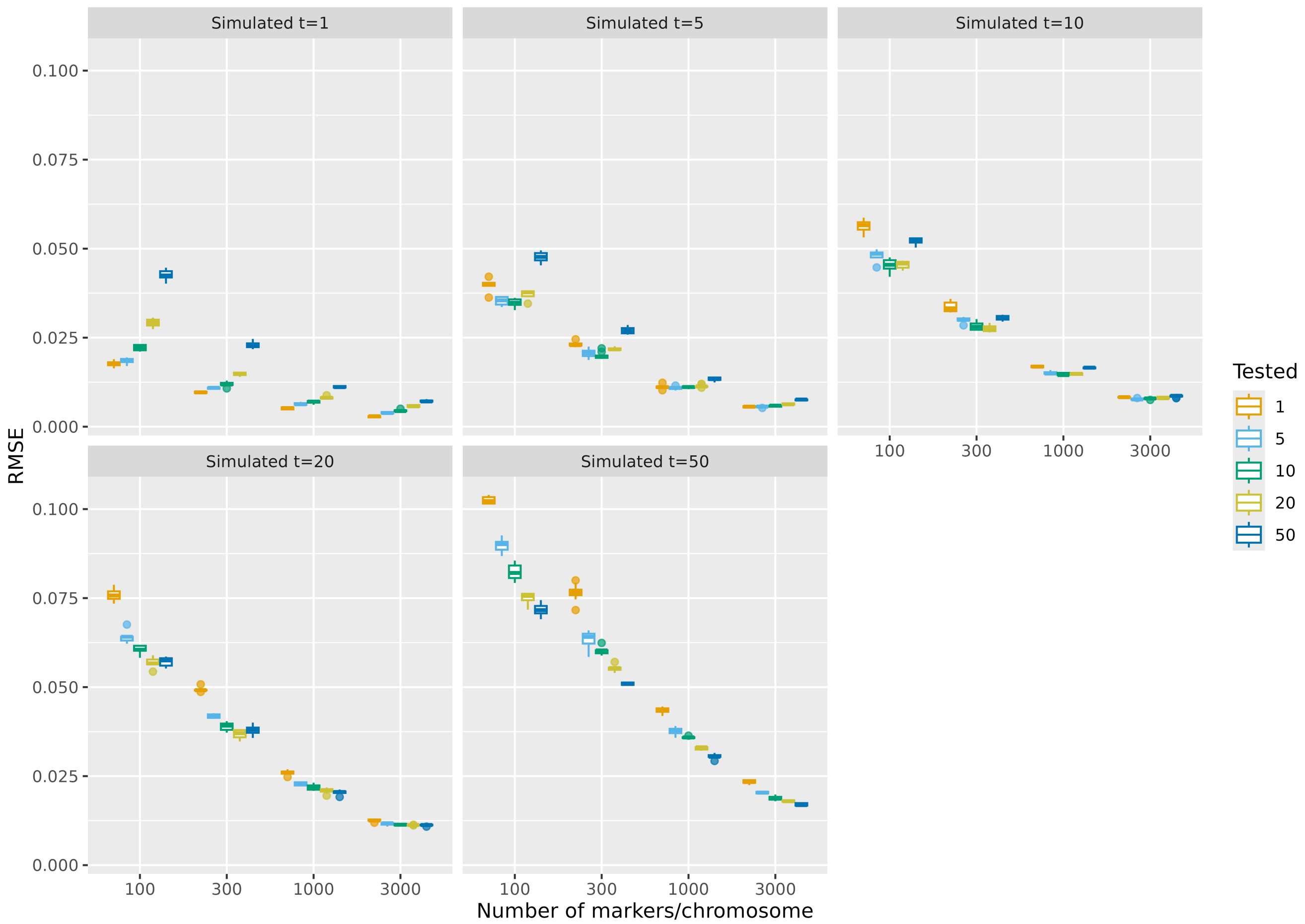

Fig S6: Root mean square error (RMSE) of local admixture ancestry proportions according to the number of markers per chromosome (distributed over 10 chromosomes), the simulated (Simulated t) and tested time (Tested t) since admixture. Each of these parameters are modulated based on a reference simulation scenario of four groups, 600 individuals, a ploidy of six and 10 alleles per marker. No approximation of transition or emission probability was used for the inference.

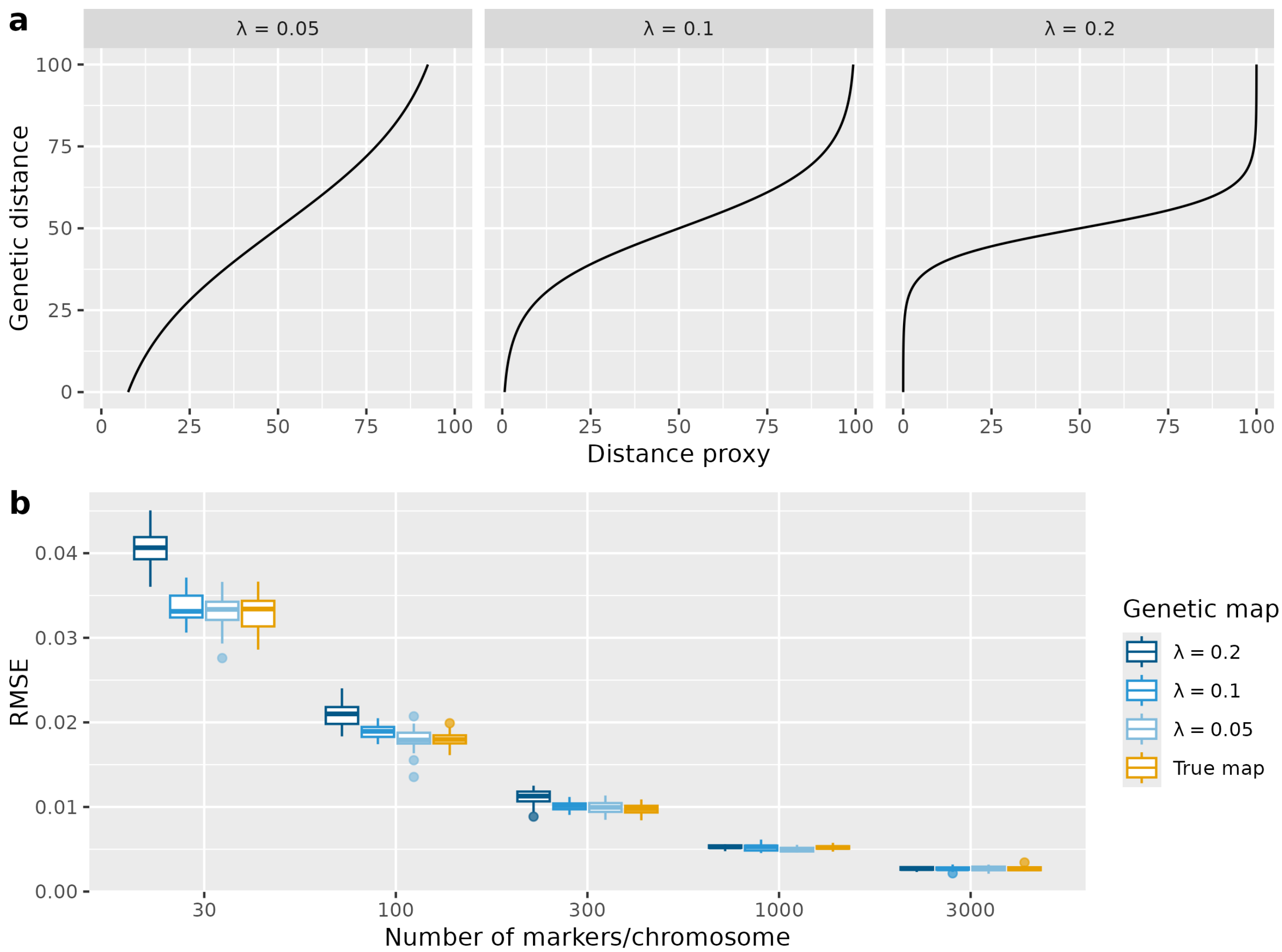

Fig S7: a) Distance proxy calculated from simulated genetic distances using $\frac{100}{1+e^{\lambda(d-50)}}$ where d is the true genetic distance and λ is a parameter increasing the non-linear relationship between the genetic distance and the proxy. b) Root mean square error (RMSE) of local admixture ancestry proportions according to the number of markers per chromosome and genetic map used: true map and proxies using different λ (0.2, 0.1 and 0.05). Simulation parameters consisted of four groups, 600 individuals, a ploidy of six, 10 alleles per marker, and one generation since admixture. No approximation was used for the inference.

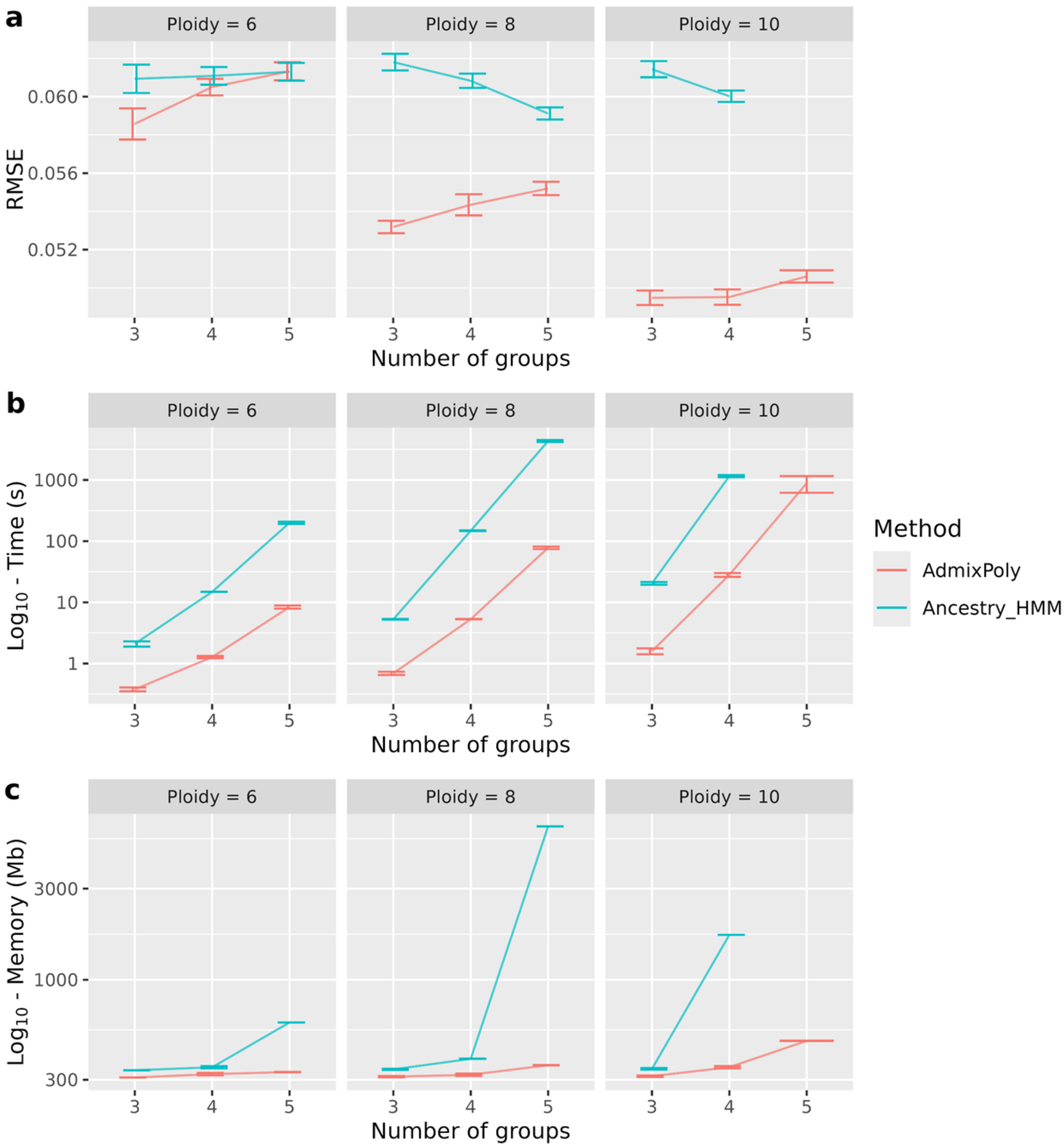

Fig S8: Comparison of a) root mean square error (RMSE) of ancestry proportions, b) computing time (in seconds, log10 scale) and c) and memory (in Mb) of local admixture inference according to the number of groups, the ploidy level and the method: `AdmixPoly` or `Ancestry HMM`. Other simulation parameters are: 100 markers per chromosome, two alleles per marker, and one generation since admixture. For all subplots, standard error bars are represented. For the inference with `AdmixPoly`, no approximation of transition or emission probability was used.

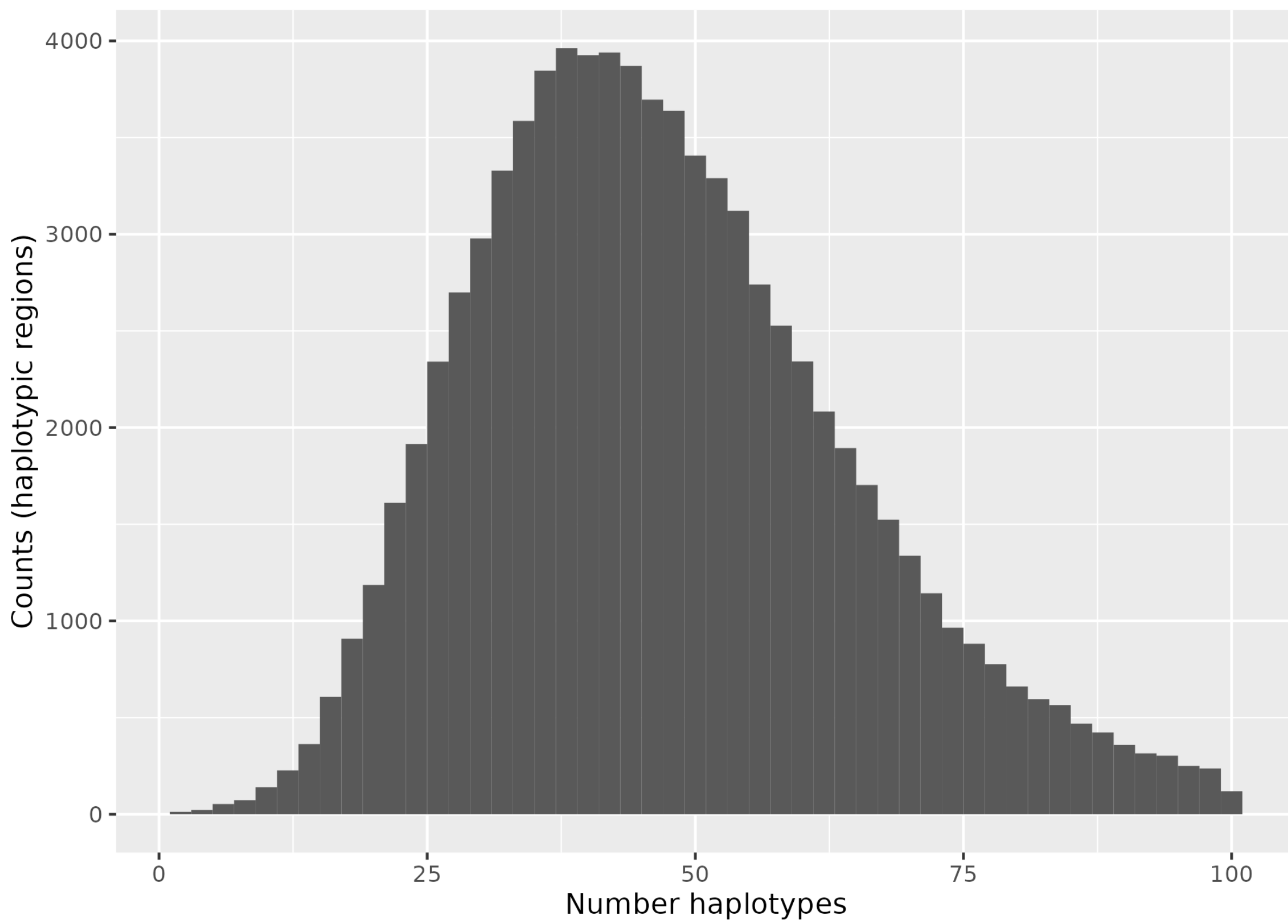

Fig S9: Distribution of number of haplotypes per haplotypic regions over the 80K regions genotyped with `HaploCharmer` for the panel of 390 accessions of *Saccharum* species and related genera. Haplotypic regions with more than 100 haplotypes were filtered out and not represented on this graph.

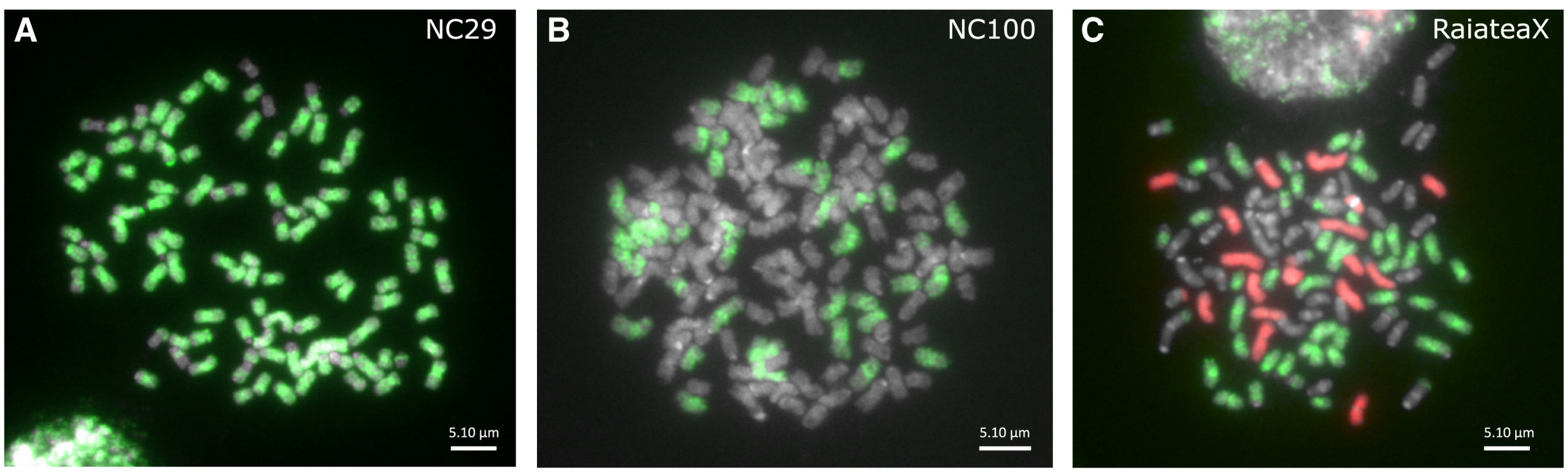

Fig S10: A) NC29, B) NC100, and C) RaiateaX metaphase chromosomes hybridized with a *S. officinarum/S. robustum* oligo-probe detected in green, and for C), total DNA of *Miscanthus* detected in red. Chromosomes were counterstained with DAPI (detected in grey). The proportion of genome originating from the unknown ancestor (in grey) were determined from these observations for NC29 (10% and similarly for NC24, NC30 and NC93), NC100 (70%) and RaiateaX (37%). This figure is adapted from Garsmeur et al. [26].

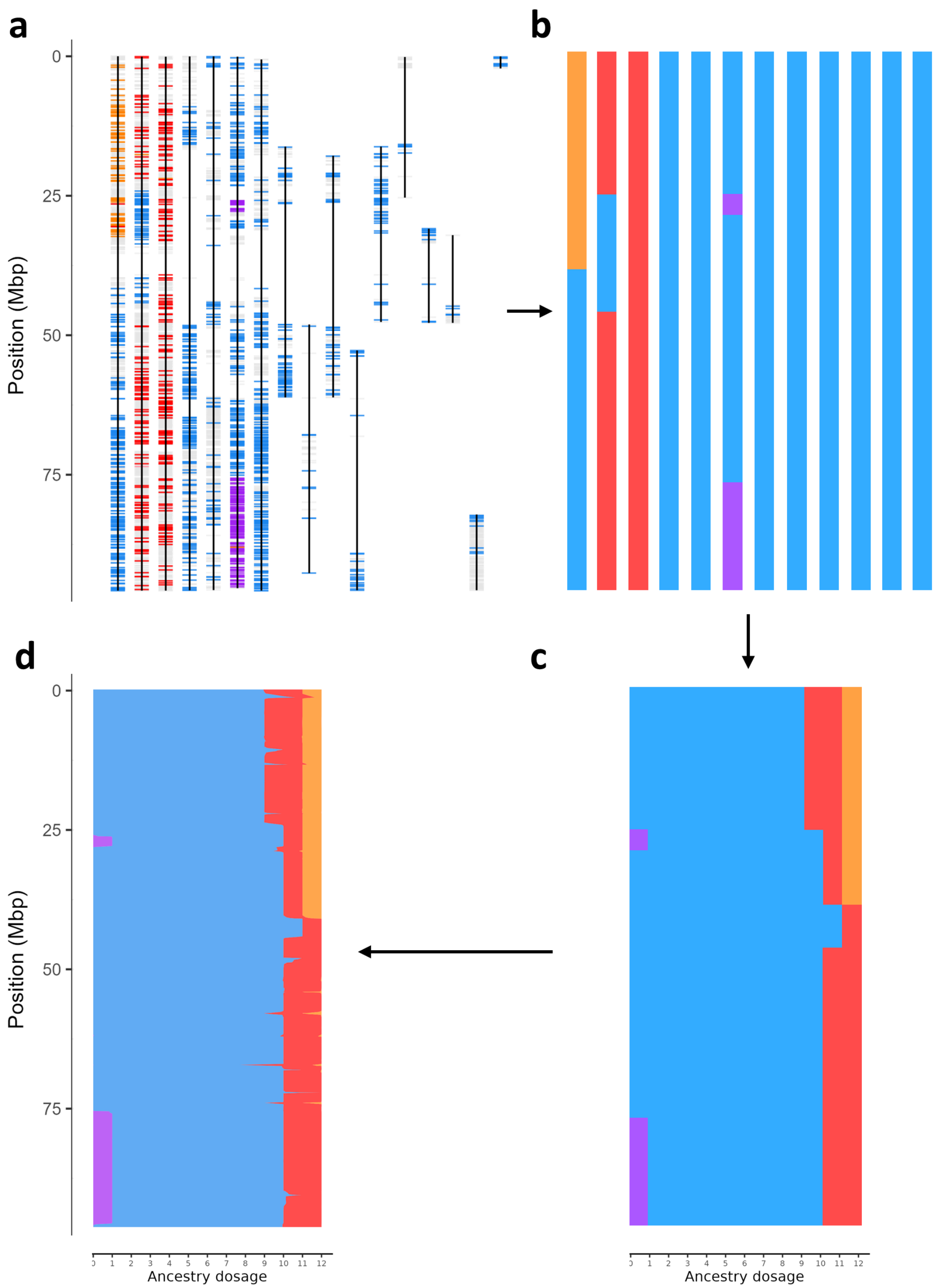

Fig S11: Evaluation of R570 local admixture. a) A genetic map of chromosome 5 from the R570 self-progeny was used as an alternative local admixture inference approach. It was built using single-dose haplotypes obtained from the same 80K haplotypic regions, some of which were defined as private to one of the ancestral groups. The genetic map based on single dose haplotypes is not saturated as it only represents the single-copy fraction of the R570 genome. The remaining duplicated fraction of R570 is expected to be mostly originating from *S. officinarum/S. robustum*. From ancestry assignments of mapped haplotypes and by filling gaps with *S. officinarum/S. robustum* ancestries, the b) phased and c) unphased ancestry dosages could be deduced for chromosome 5. d) The unphased ancestry dosages could then be directly compared to the ancestry dosages obtained from `AdmixPoly` for chromosome 5.

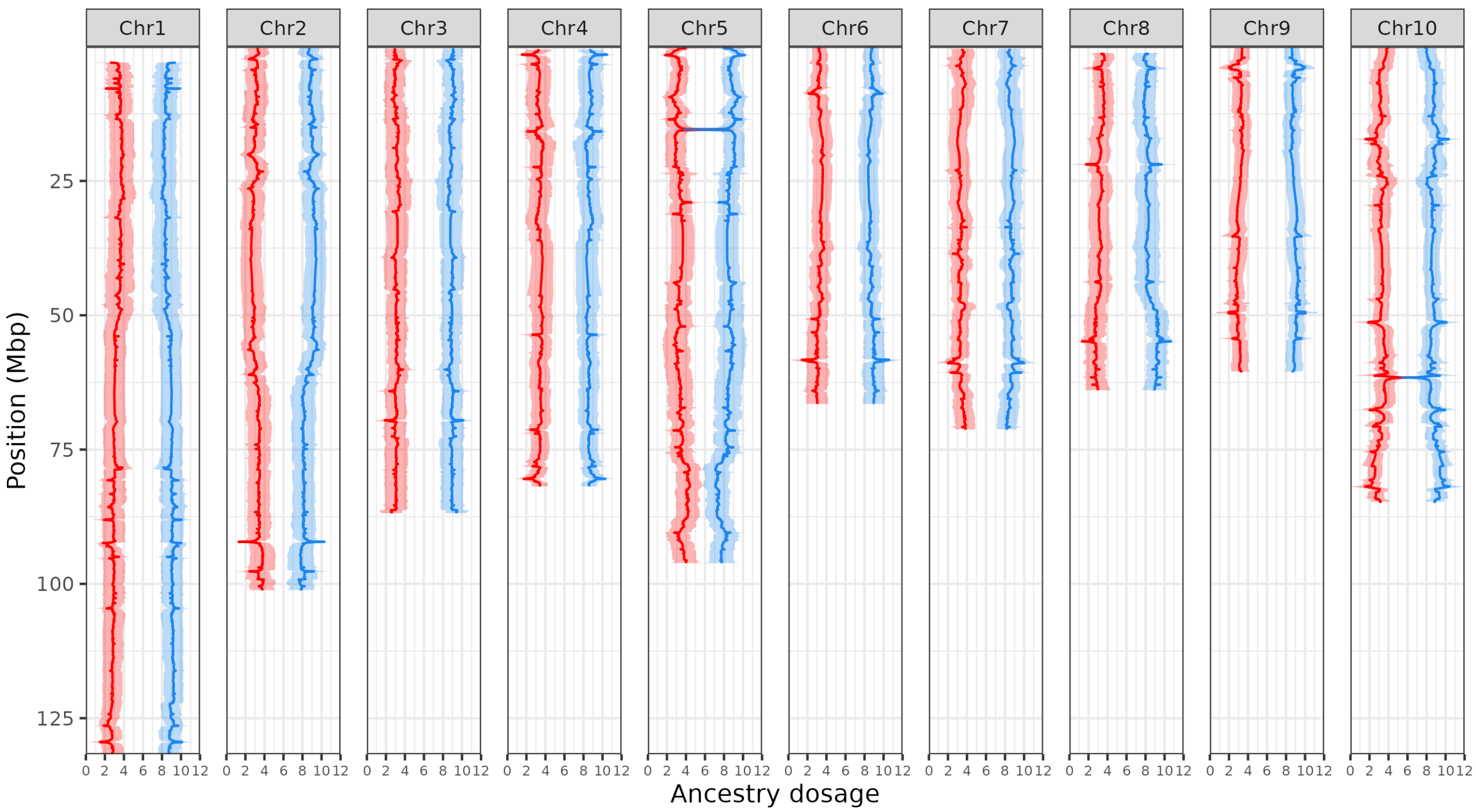

Fig S12: Average *S. officinarum* (in blue) and *S. spontaneum* (in red) contributions over the genome of all modern cultivars. The colored areas correspond to intervals representing the standard deviations from the average contributions. The small spikes likely indicate haplotypic regions for which reads originating from one of the species presented mapping issues, leading to biased haplotype read depth ratios.

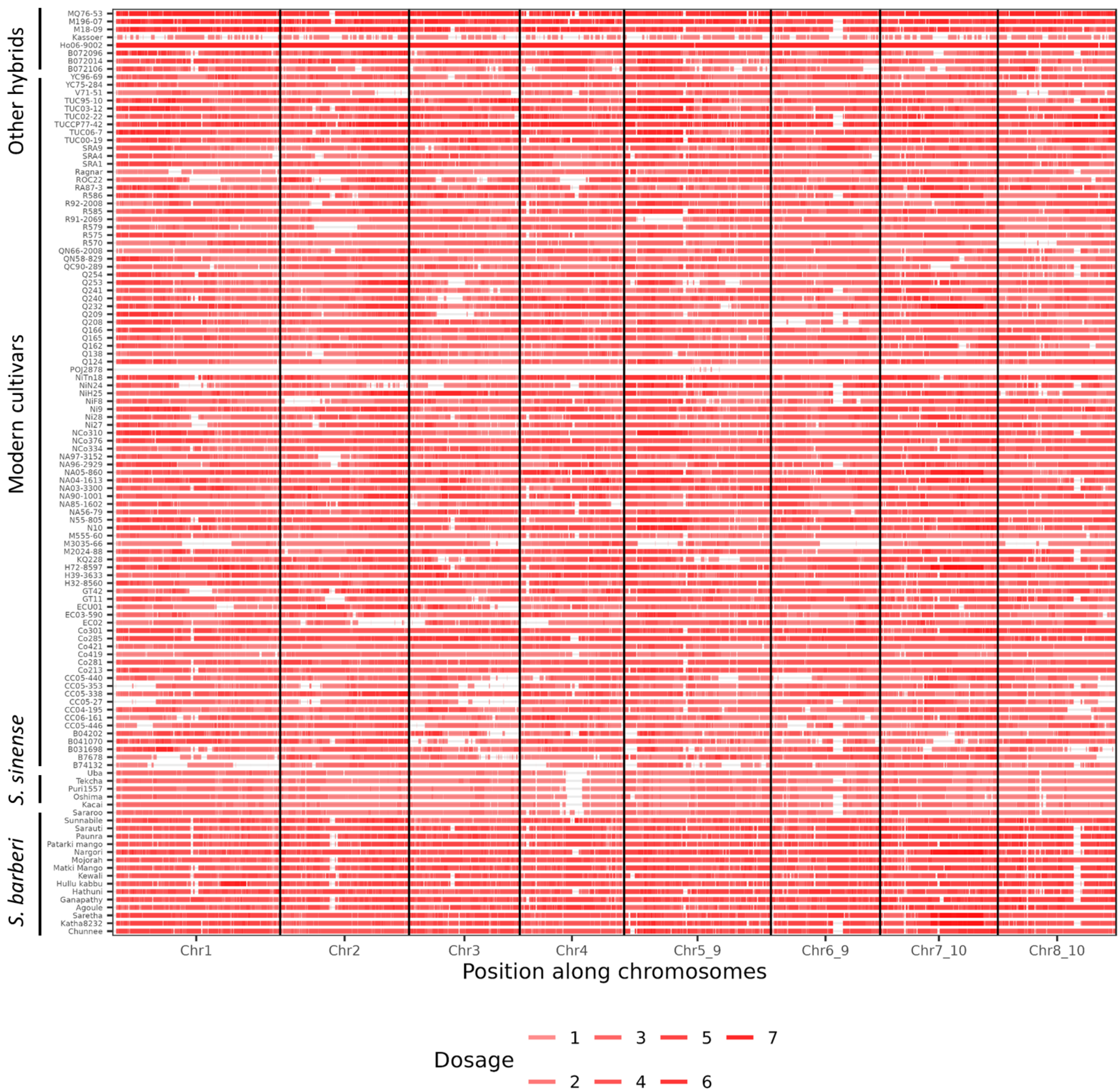

Fig S13: Shared contributions from the Continental *S. spontaneum* subgroup between modern and former interspecific hybrid cultivars, reported on the *S. spontaneum* genome reference structure ($x = 8$).

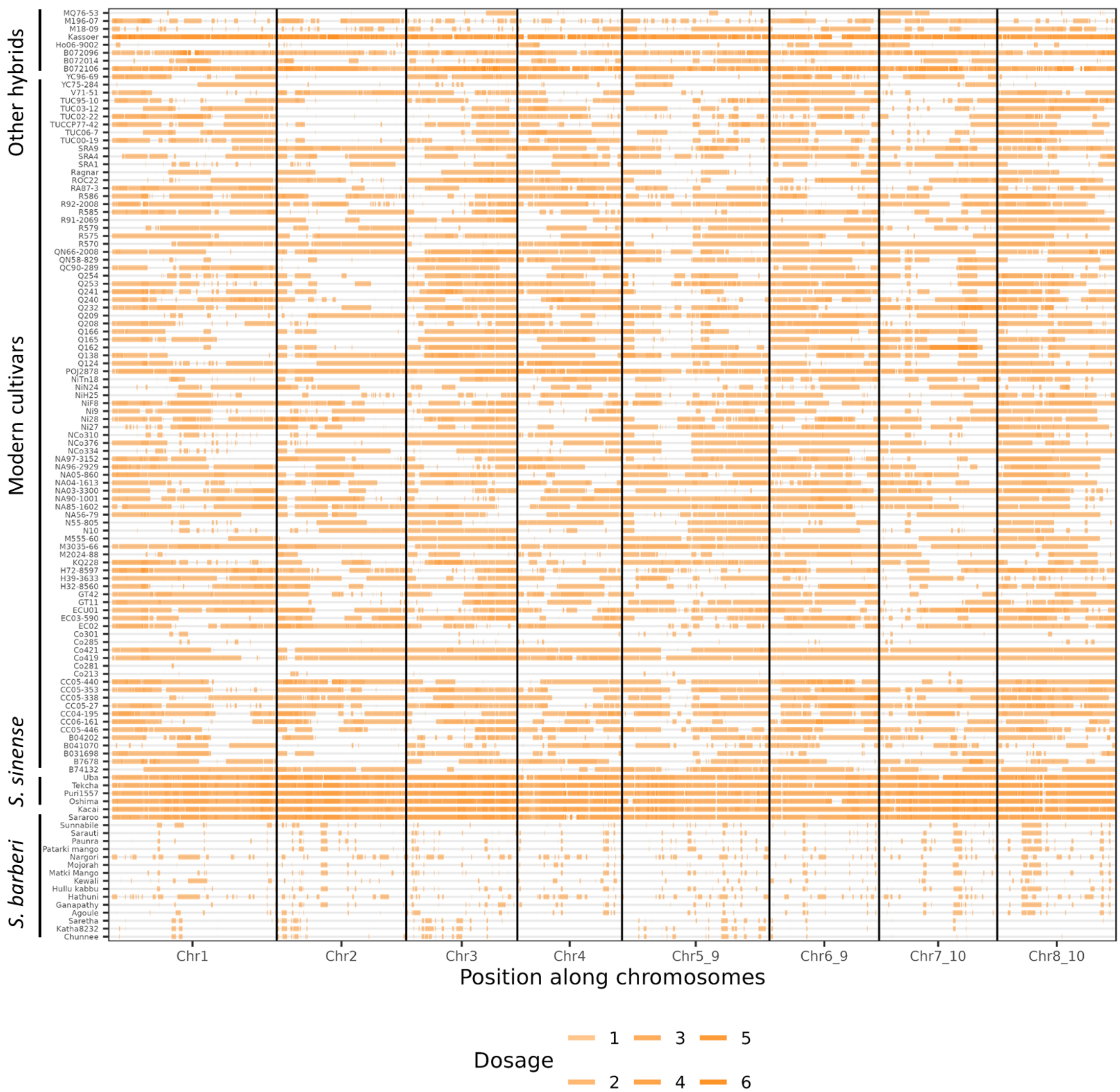

Fig S14: Shared contributions from the Southeast Asia *S. spontaneum* subgroup between modern and former interspecific hybrid cultivars, reported on the *S. spontaneum* genome reference structure ($x = 8$).

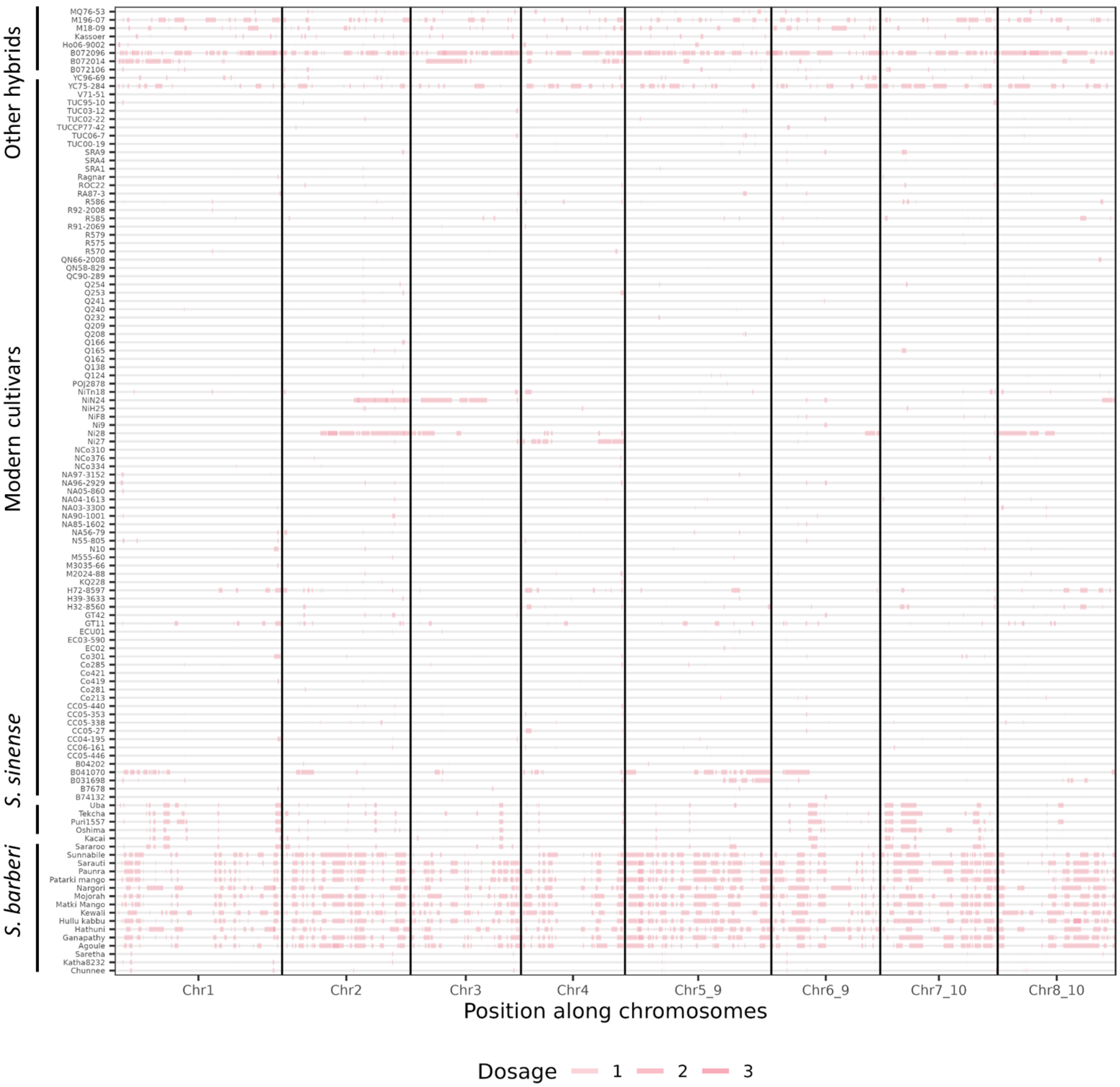

Fig S15: Shared contributions from the Northeast Asia *S. spontaneum* subgroup between modern and former interspecific hybrid cultivars, reported on the *S. spontaneum* genome reference structure ($x = 8$).
