# Supplementary material for: Deciphering the mosaic genome of sugarcane cultivars through polyploid admixture inference with AdmixPoly
Source: Genome Biol. 2026 Jun 19;27:280. doi: 10.1186/s13059-026-04162-3 (PMC13531838; doi:10.1186/s13059-026-04162-3)
Supplement: Supplementary file 2 [file 13059_2026_4162_MOESM2_ESM.pdf]

Additional file 2: Local admixture of sugarcane cultivars

Deciphering the Mosaic Genome of Sugarcane Cultivars through Polyploid Admixture Inference with AdmixPoly

S. Rio[1,2], F. Gauthier[3], O. Garsmeur[1,2], G. Piperidis[4], J.Y. Hoarau[5,2], G. Serino[6], R. Castillo Torres[7], S. V. Joshi[8,9], Y. Terajima[10], J. Lopez-Gerena[11], M. F. Perera[12], A. Stoute[13], G. Badaloo[14], D. Huang[15], K. Barry[16], J. Schmutz[16,17], T. Mary-Huard[3,18,α], A. D'Hont[1,2,α]

[1]CIRAD, UMR AGAP Institut, F-34398 Montpellier, France
[2]UMR AGAP Institut, Univ Montpellier, CIRAD, INRAE, Institut Agro, Montpellier, France
[3]Université Paris-Saclay, INRAE, CNRS, AgroParisTech, UMR GQE-Le Moulon, 91190, Gif-sur-Yvette, France
[4]Sugar Research Australia, Brisbane QLD 4000
[5]eRcane, Sainte-Clotilde, La Réunion, France
[6]Chacra Experimental Agrícola Santa Rosa, Camino Vecinal No. 8 Km 6, Colonia Santa Rosa, 4531 Salta, Argentina
[7]Centro de Investigación de la Caña de Azúcar del Ecuador - CINCAE, Guayaquil, Ecuador
[8]South African Sugarcane Research Institute, Private Bag X02, Mount Edgecombe, Durban, 4300, KwaZulu-Natal, South Africa
[9]School of Agriculture and Science, College of Agriculture, Engineering and Science, University of KwaZulu-Natal, South Africa
[10]Tropical Agriculture Research Front, JIRCAS, 1091-1 Maezato-Kawarabaru, Ishigaki, Okinawa, 907-0002, Japan
[11]Centro de Investigación de la Caña de Azúcar de Colombia (CENICAÑA), Cali, Colombia
[12]Instituto de Tecnología Agroindustrial del Noroeste Argentino (ITANOA), Estación Experimental Agroindustrial Obispo Colombres (EEAOC) - Consejo Nacional de Investigaciones Científicas y Técnicas (CONICET), Las Talitas, Tucumán, Argentina
[13]West Indies Central Sugar Cane Breeding Station, Groves, St. George, Barbados
[14]Mauritius Sugarcane Industry Research Institute, Mauritius Cane Industry Authority, 1 Moka Road, Réduit, Mauritius
[15]Sugarcane Research Institute, Guangxi Academy of Agricultural Sciences, Nanning, China
[16]DOE Joint Genome Institute, Lawrence Berkeley National Laboratory, Berkeley, CA, 94720, USA
[17]HudsonAlpha Institute for Biotechnology, 601 Genome Way Northwest, Huntsville, AL 35806, USA
[18]Université Paris-Saclay, AgroParisTech, INRAE, UMR MIA-Paris, 91120, Palaiseau, France

[α]These authors contributed equally to the work
Corresponding authors: Simon Rio (simon.rio@cirad.fr)

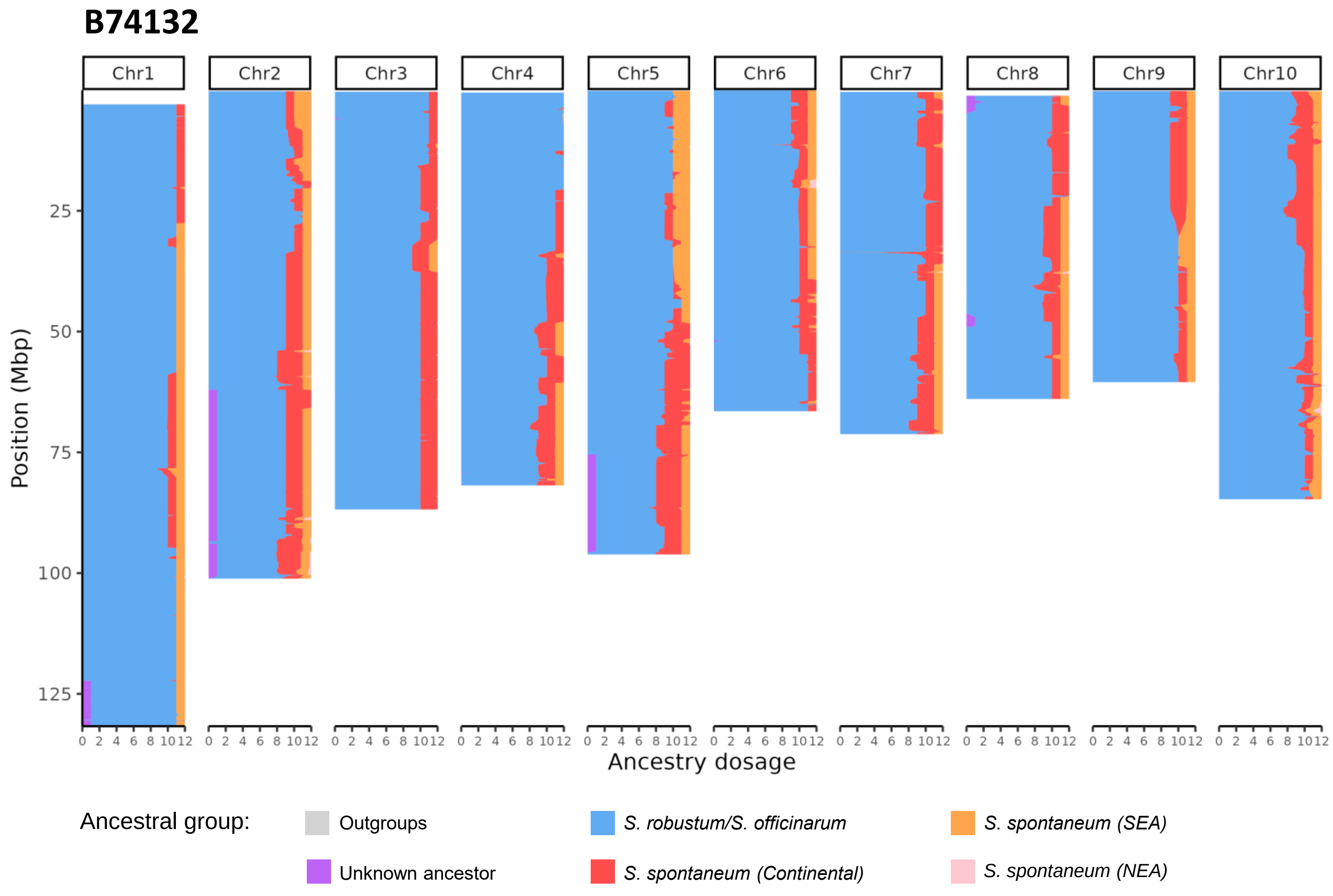

Local admixture of the modern cultivar B74132

B7678

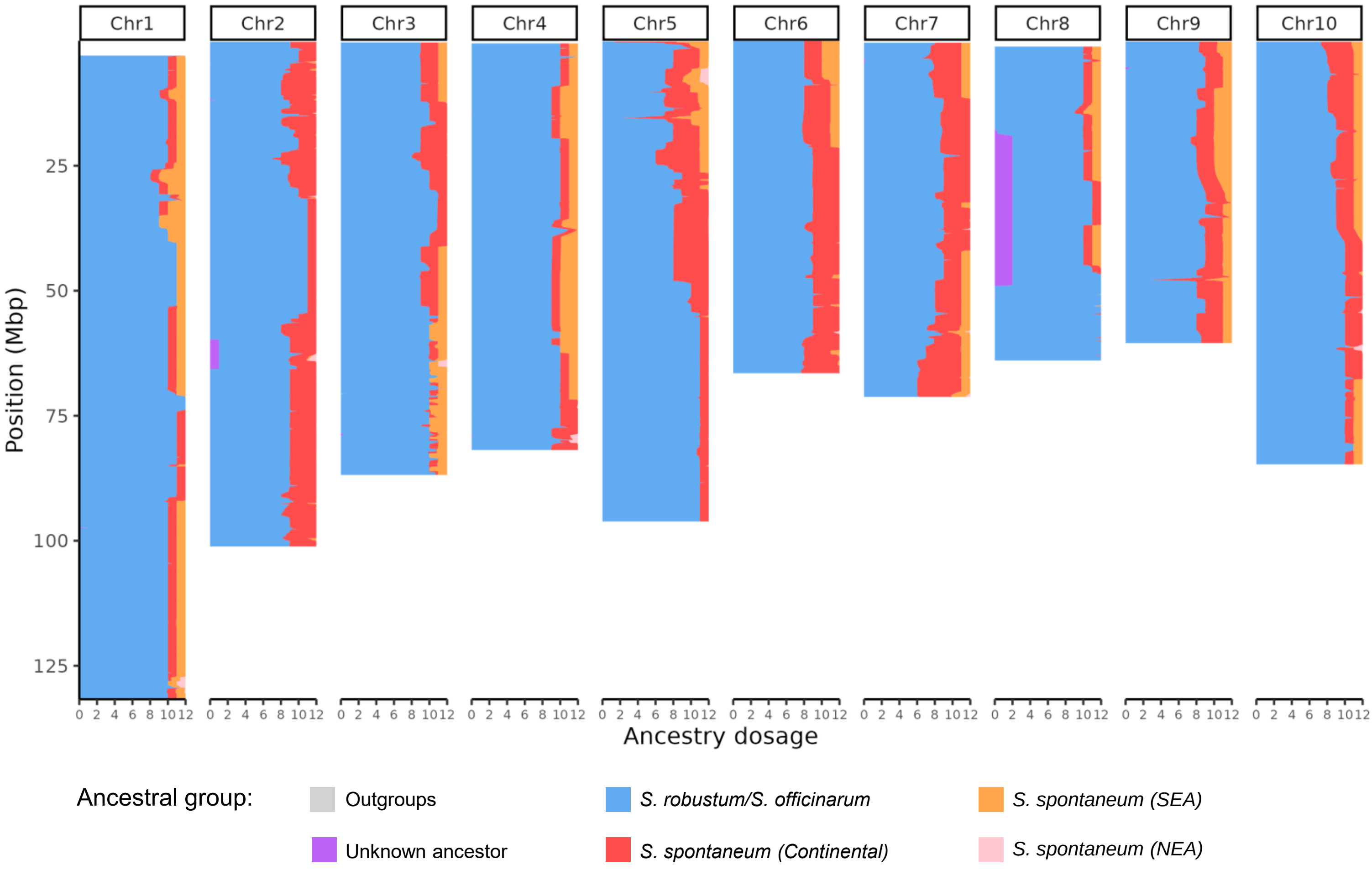

Local admixture of the modern cultivar B7678

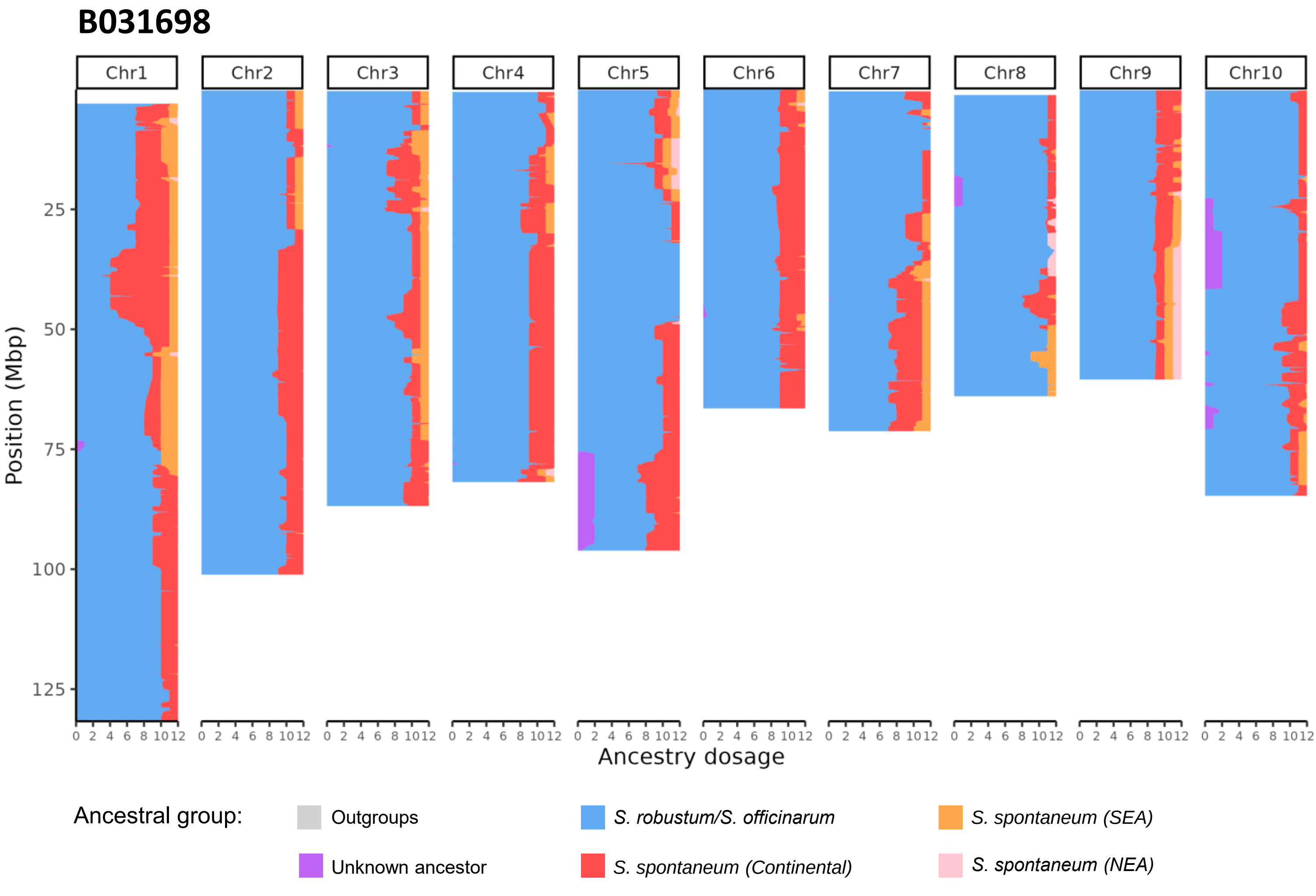

Local admixture of the modern cultivar B031698

B041070

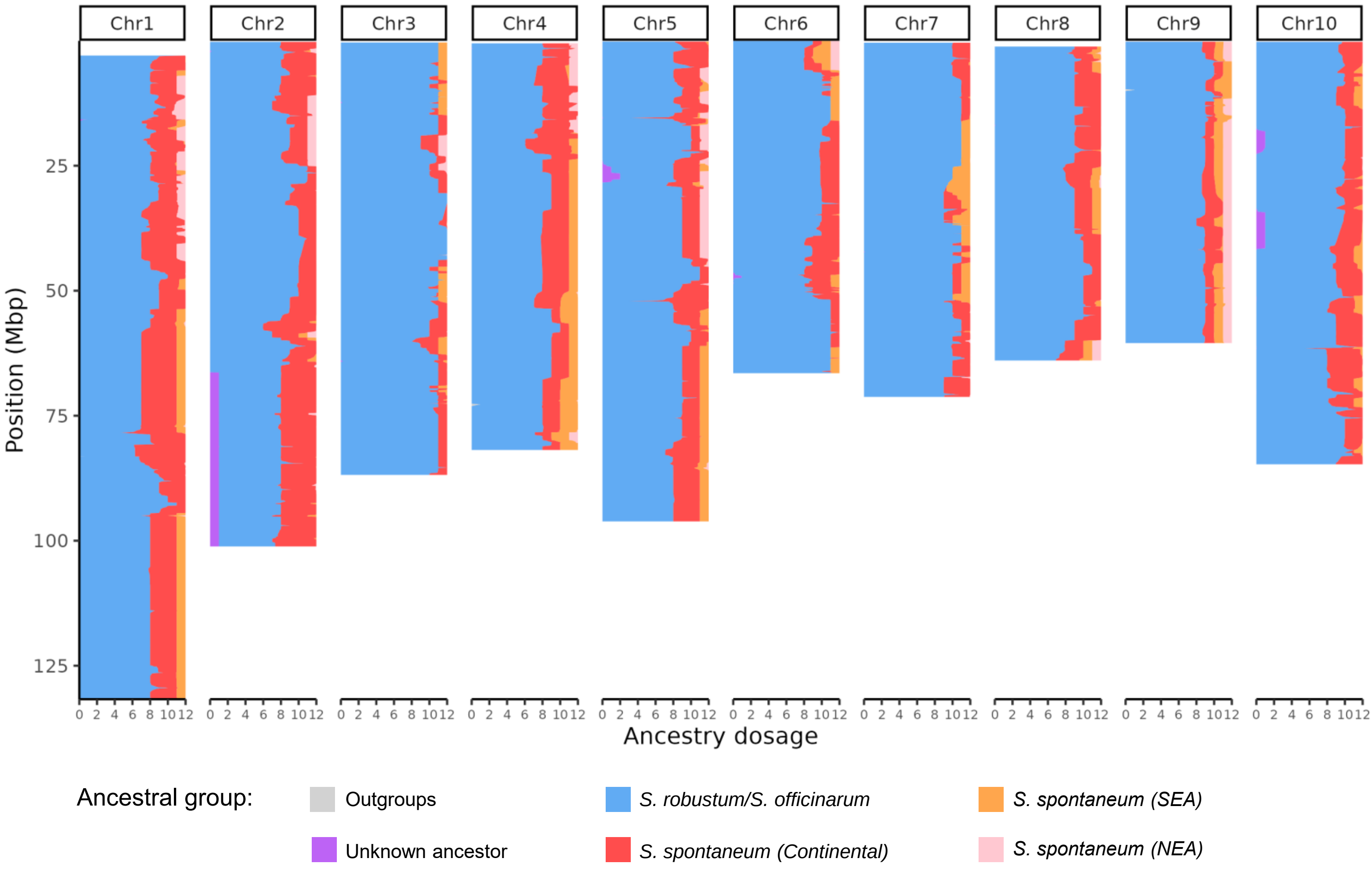

Local admixture of the modern cultivar B041070

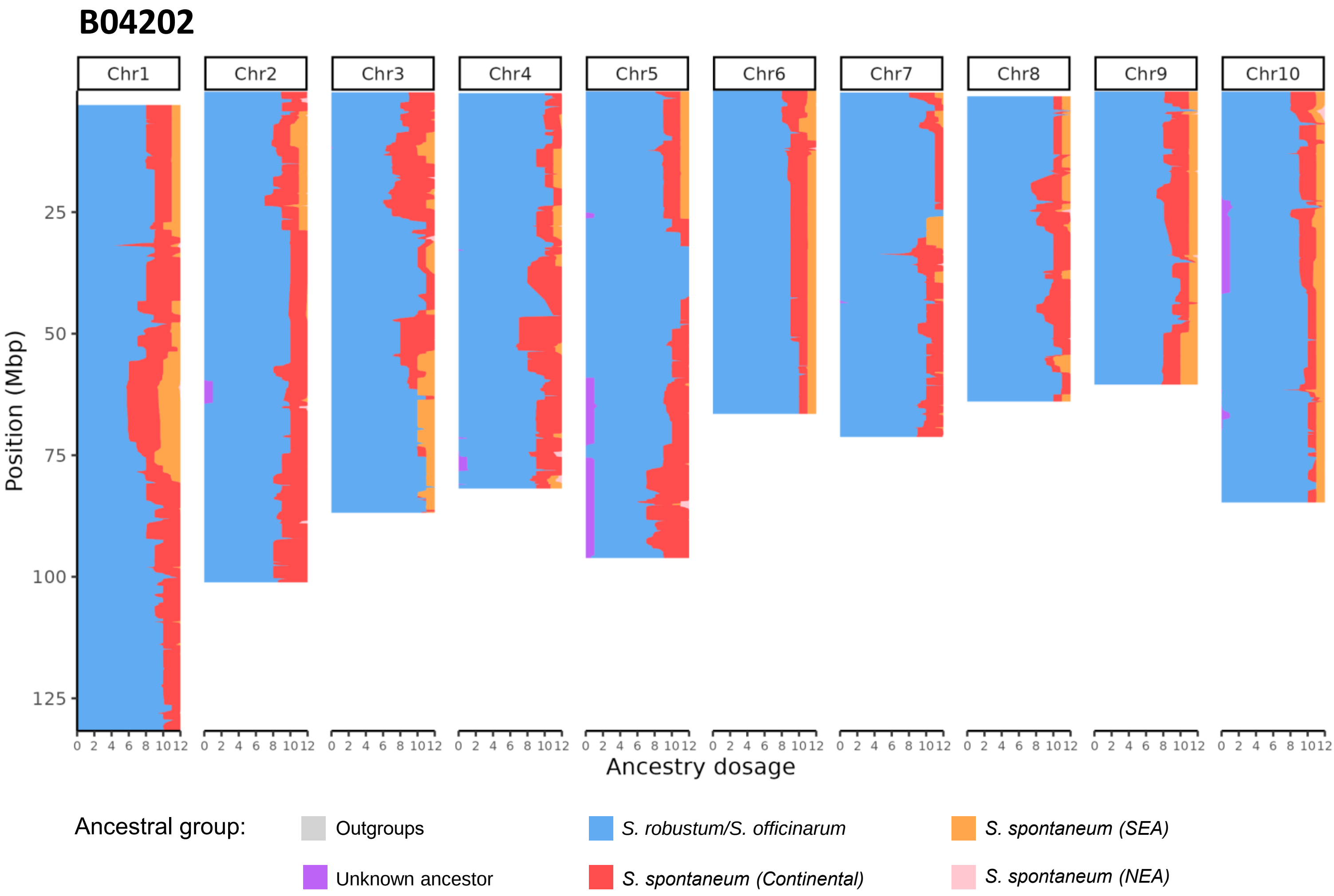

Local admixture of the modern cultivar B04202

CC05-446

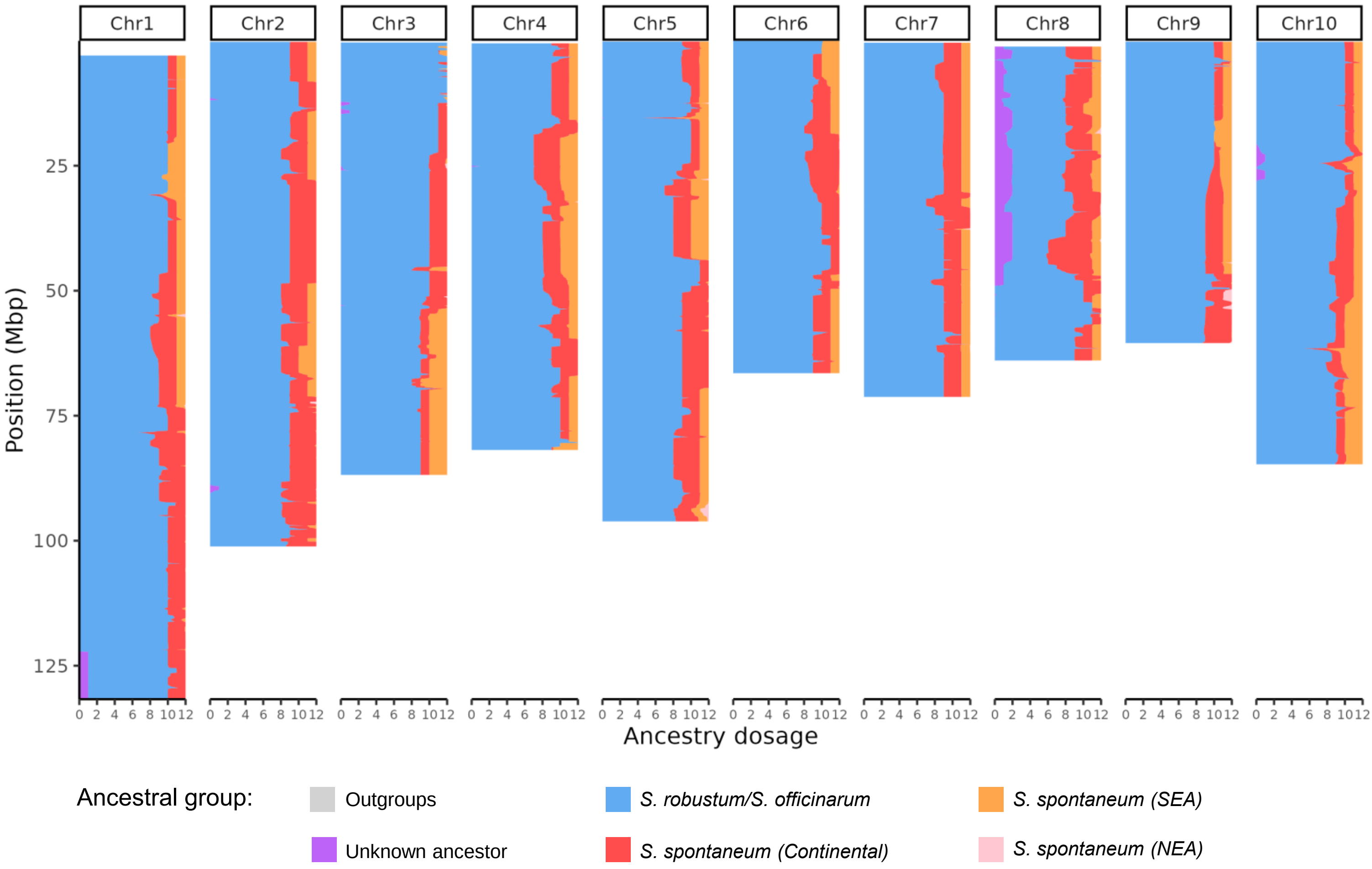

Local admixture of the modern cultivar CC05-446

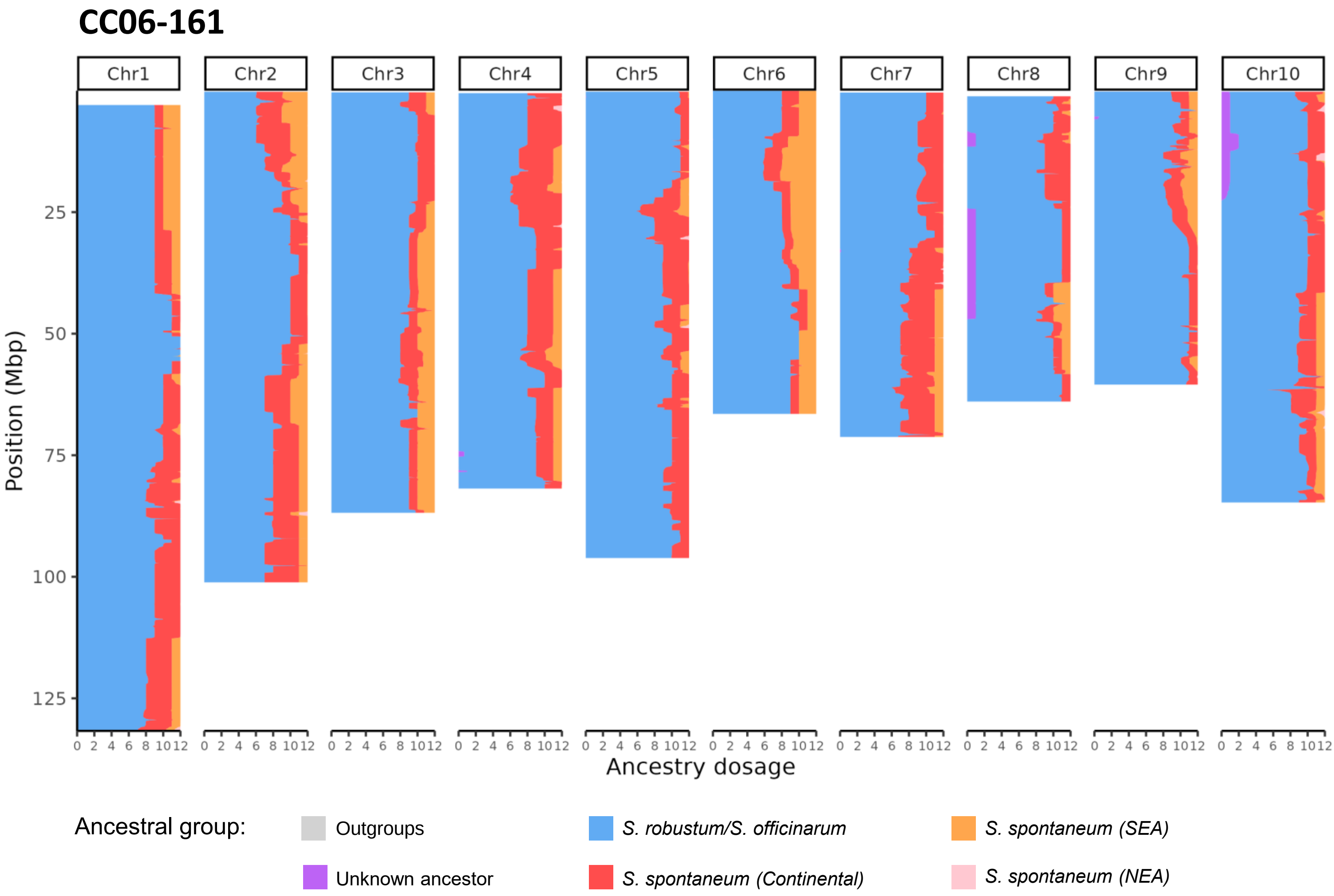

Local admixture of the modern cultivar CC06-161

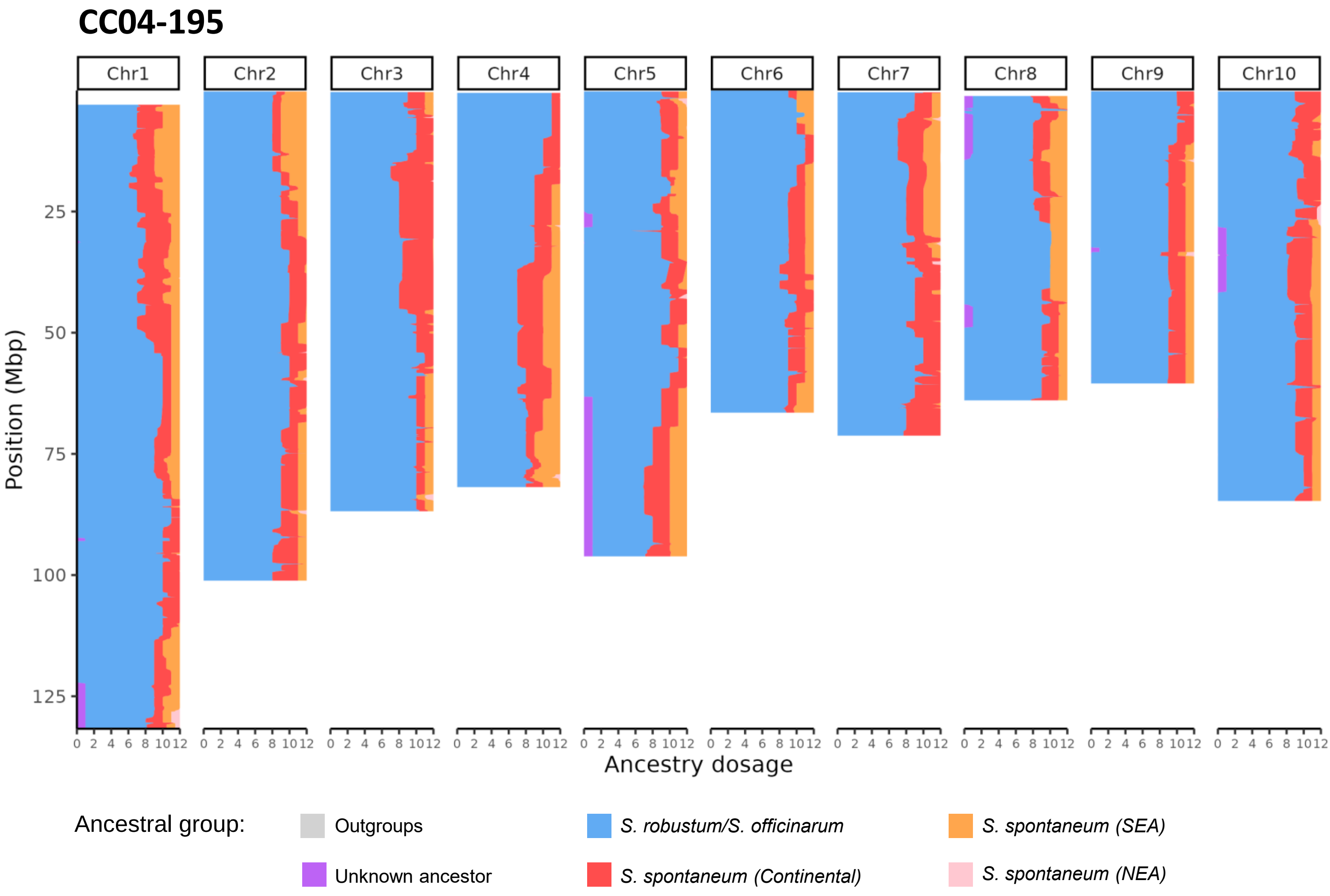

Local admixture of the modern cultivar CC04-195

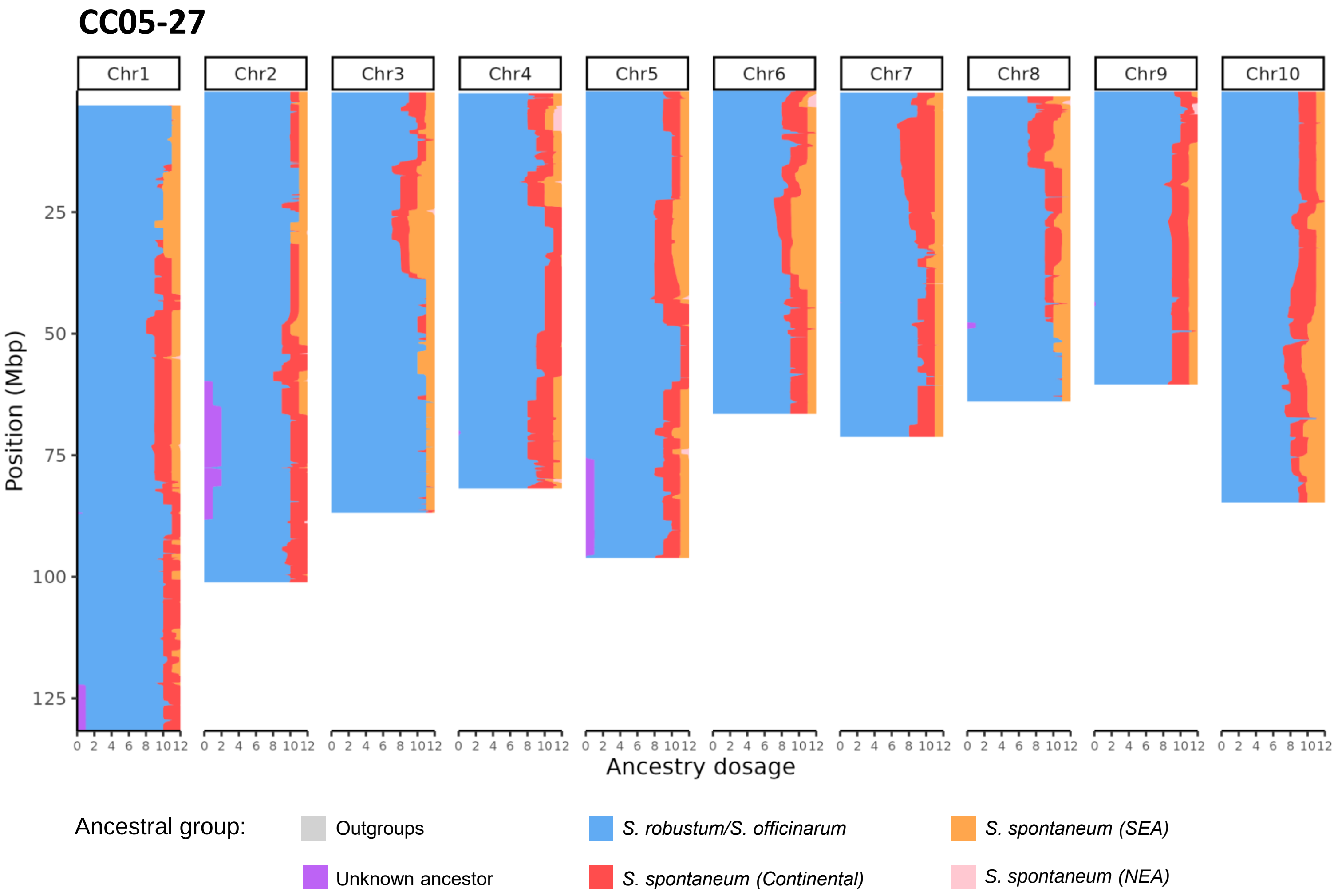

Local admixture of the modern cultivar CC05-27

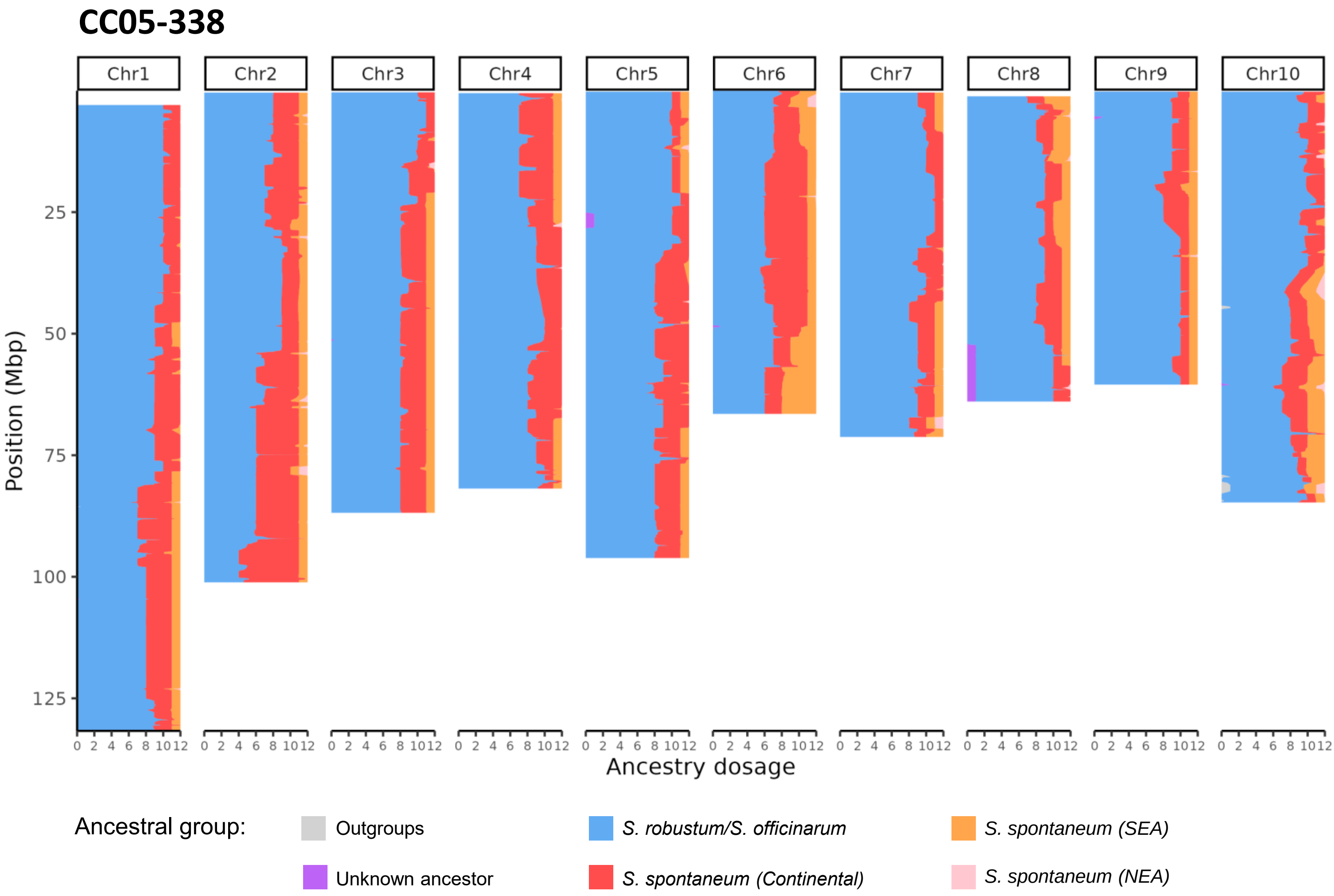

Local admixture of the modern cultivar CC05-338

CC05-353

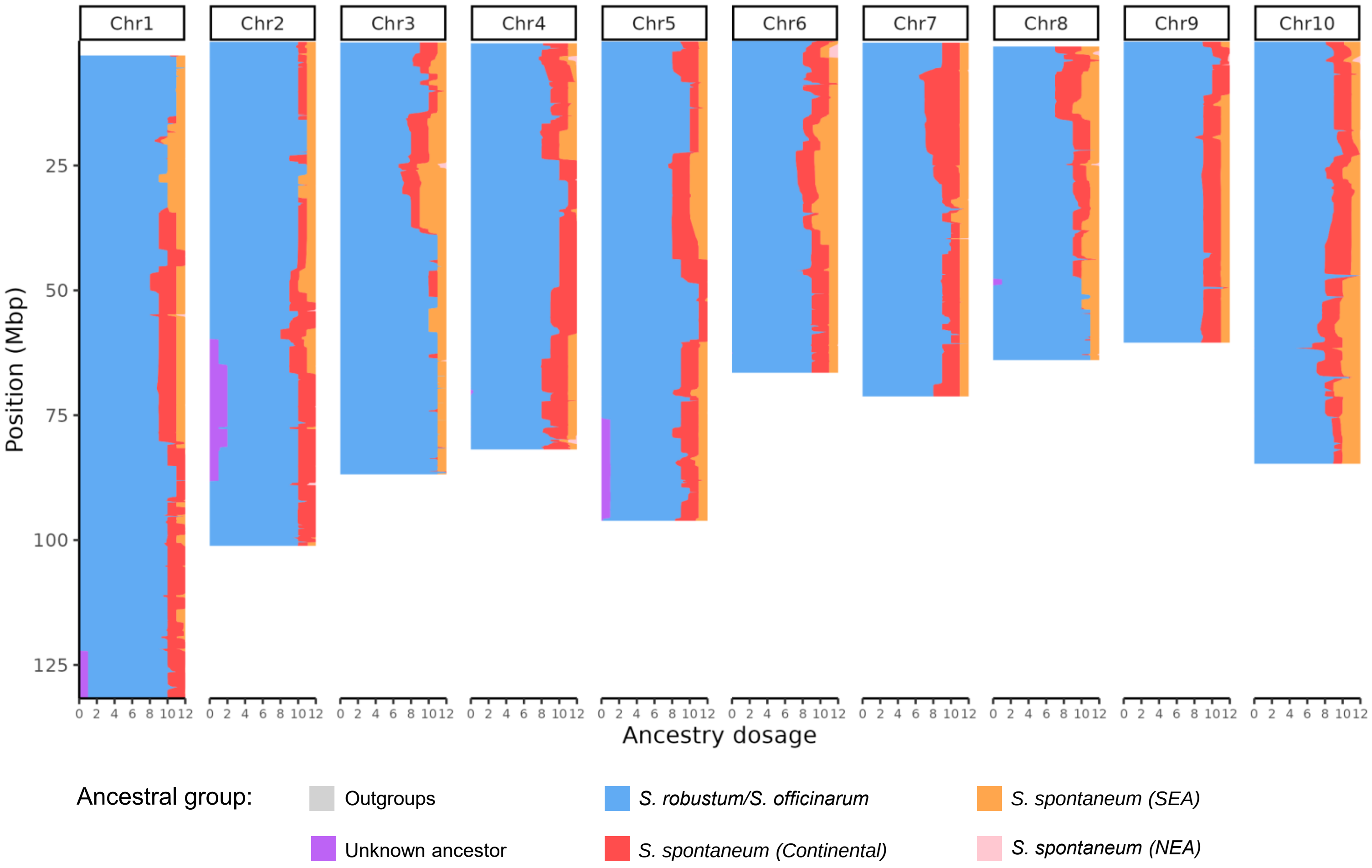

Local admixture of the modern cultivar CC05-353

CC05-440

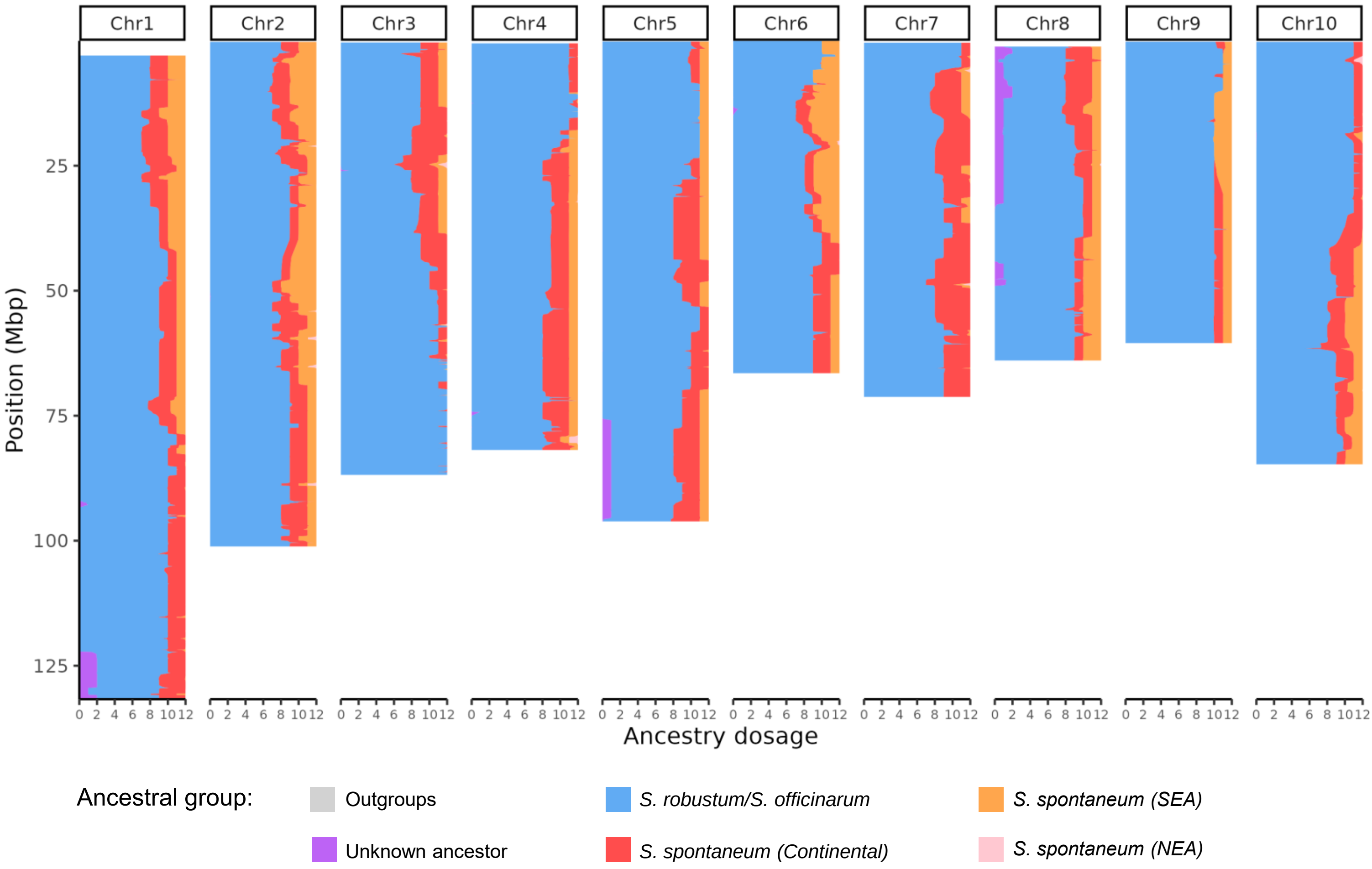

Local admixture of the modern cultivar CC05-440

Co213

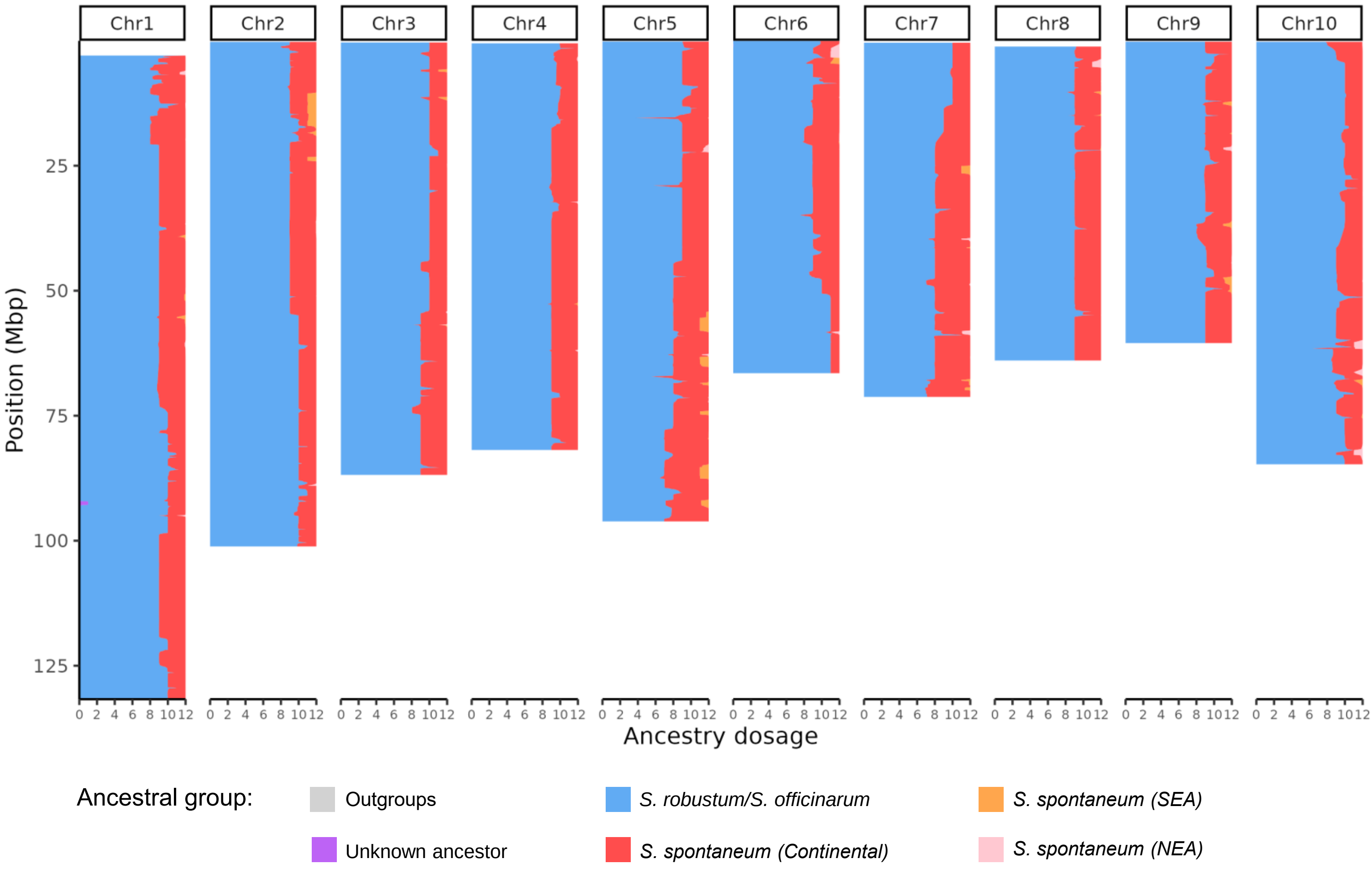

Local admixture of the modern cultivar Co213

Co281

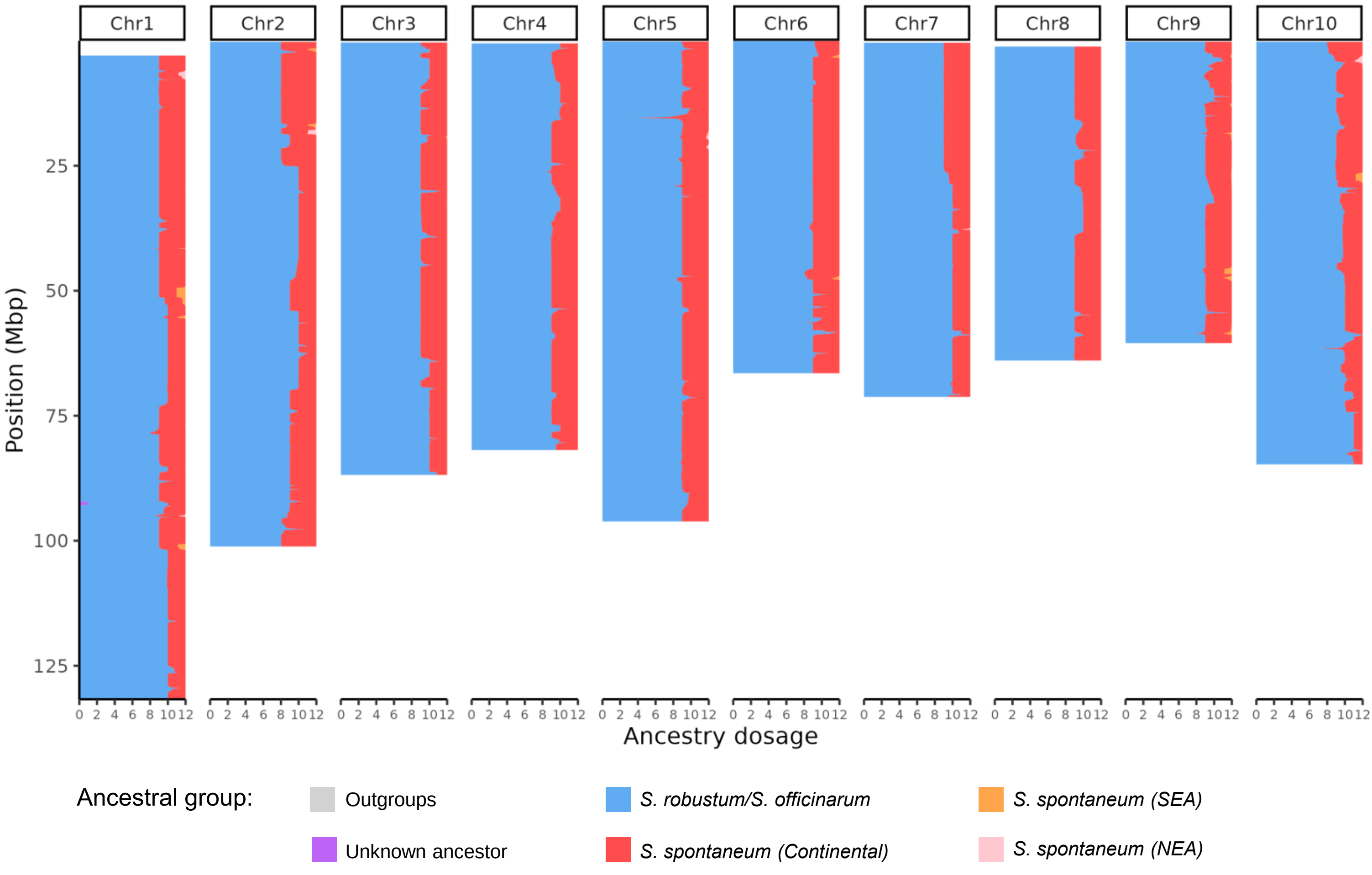

Local admixture of the modern cultivar Co281

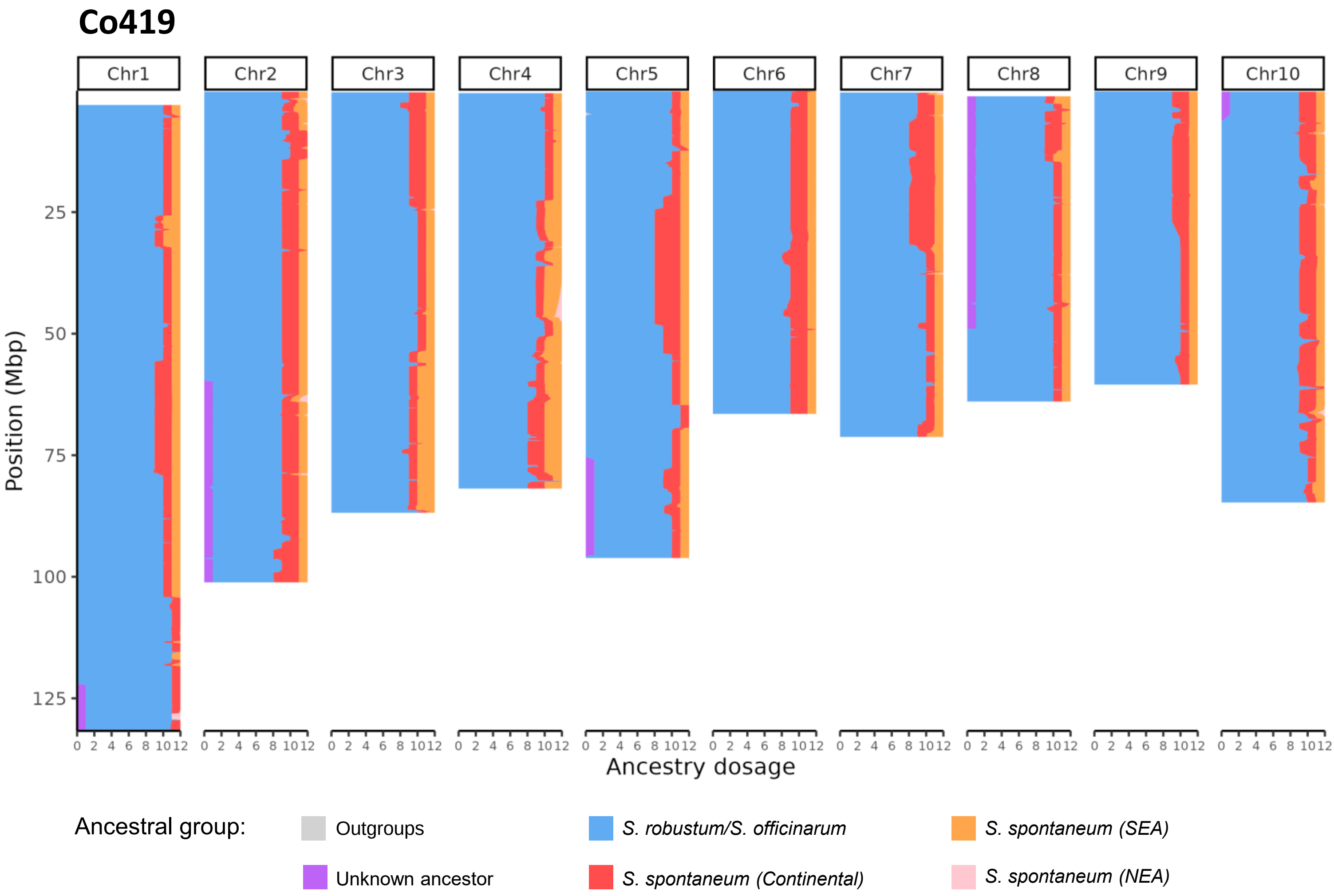

Local admixture of the modern cultivar Co419

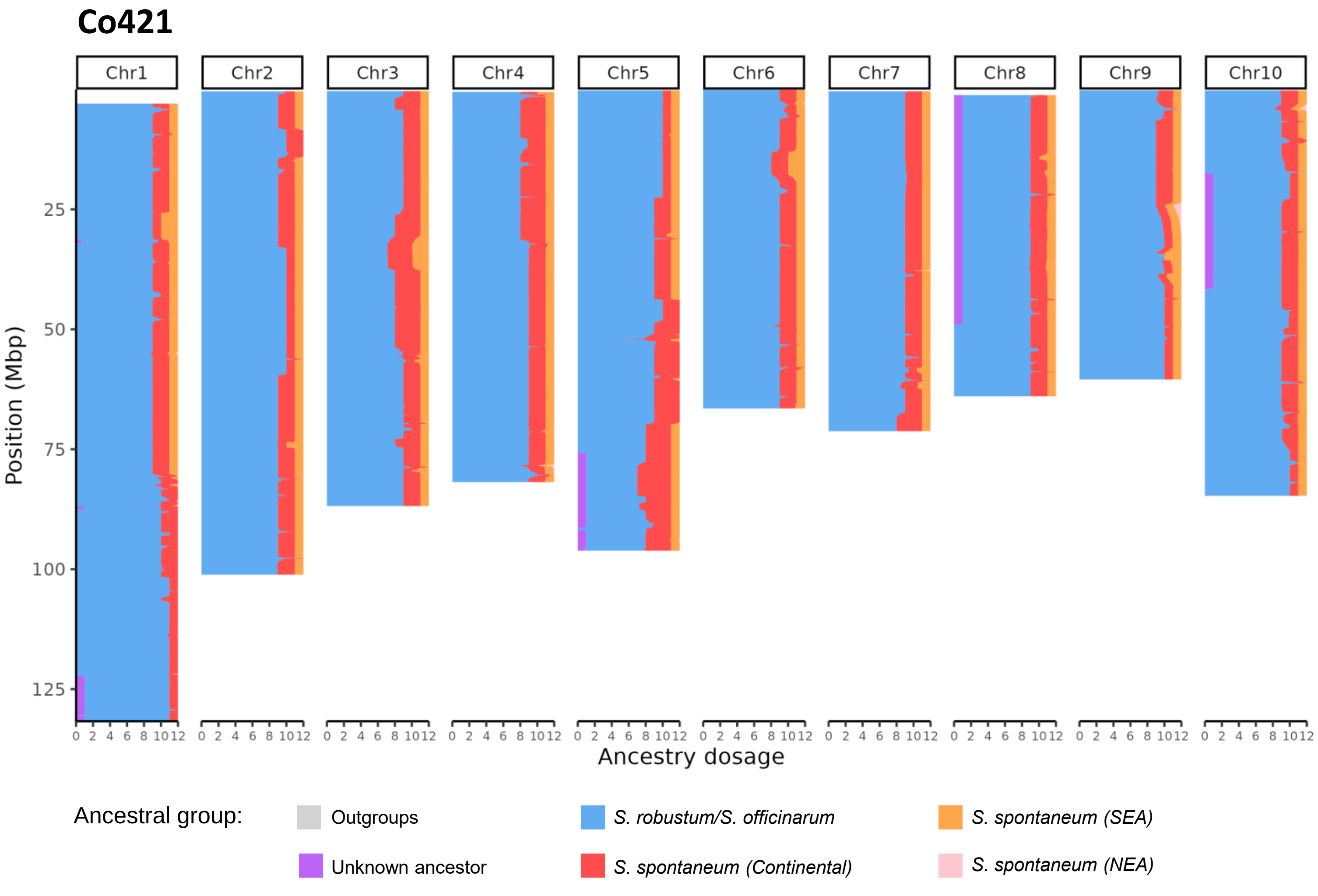

Local admixture of the modern cultivar Co421

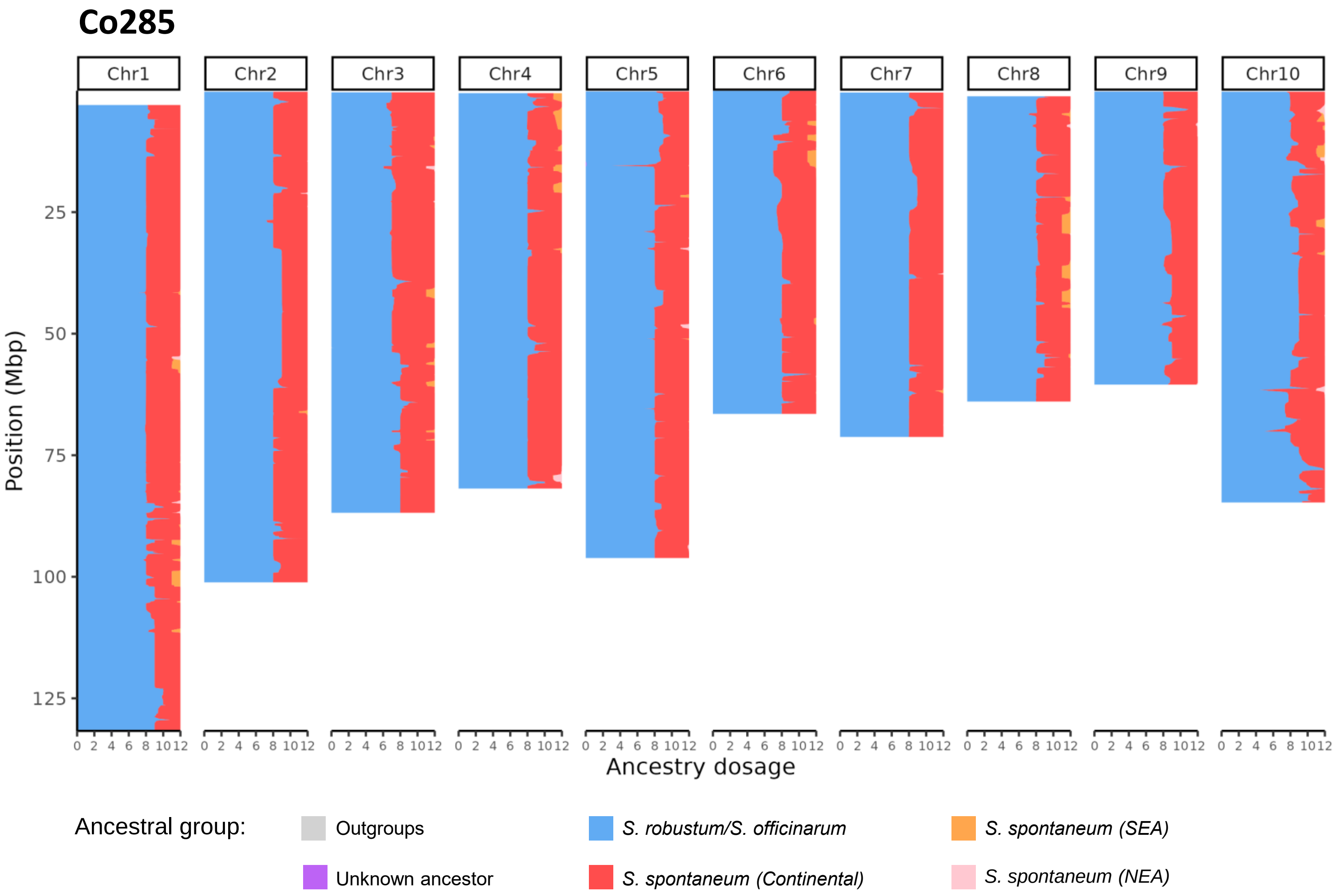

Local admixture of the modern cultivar Co285

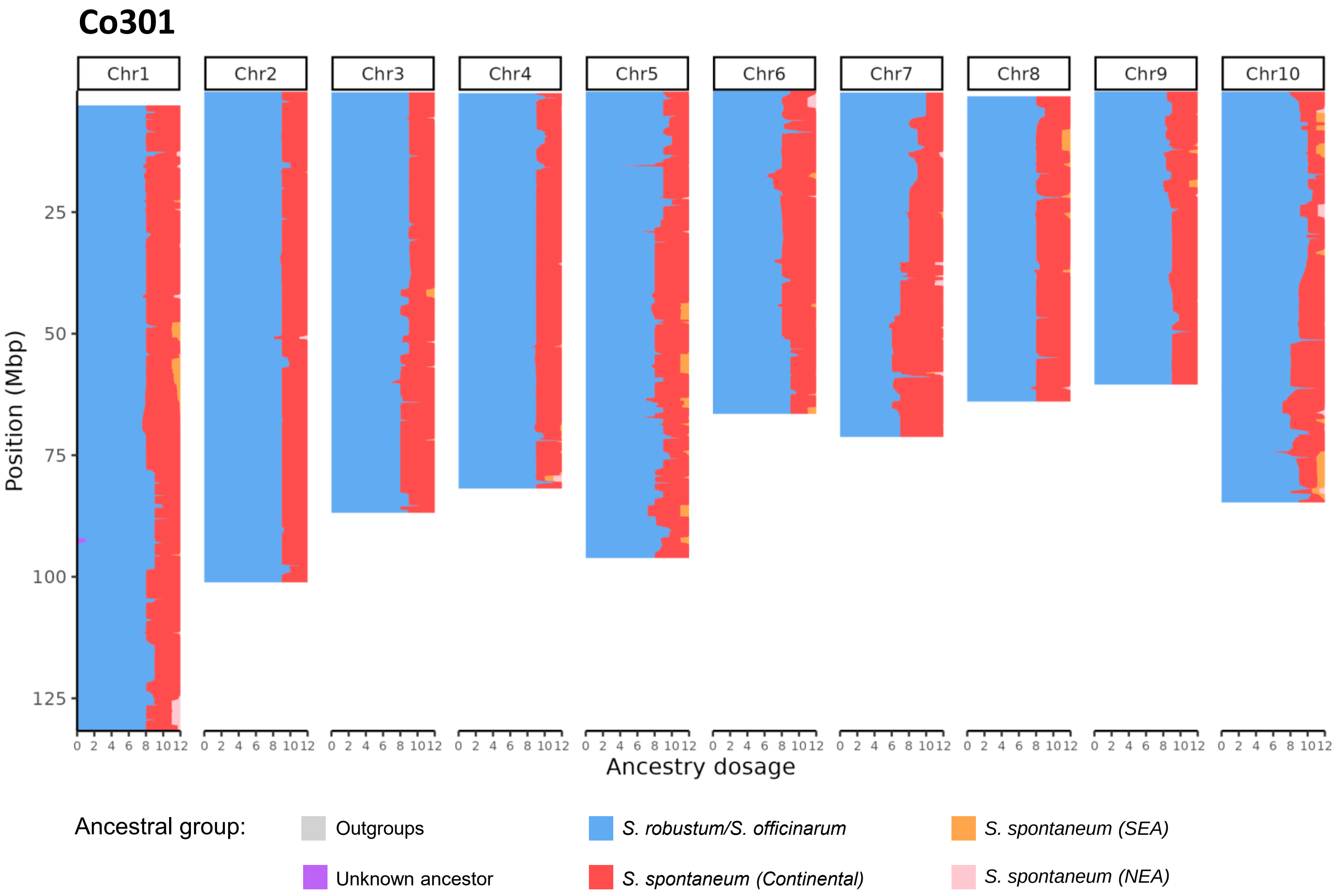

Local admixture of the modern cultivar Co301

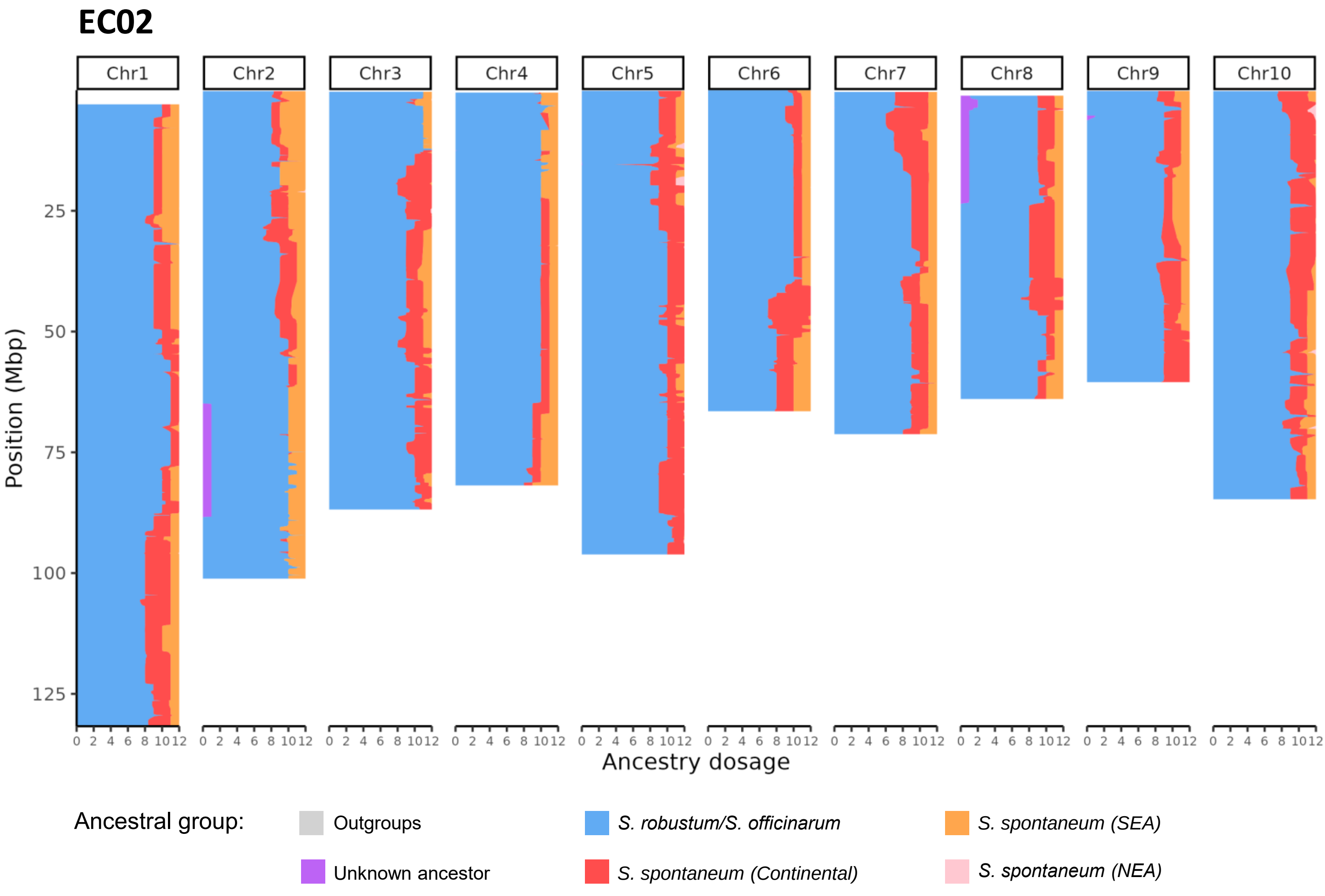

Local admixture of the modern cultivar EC02

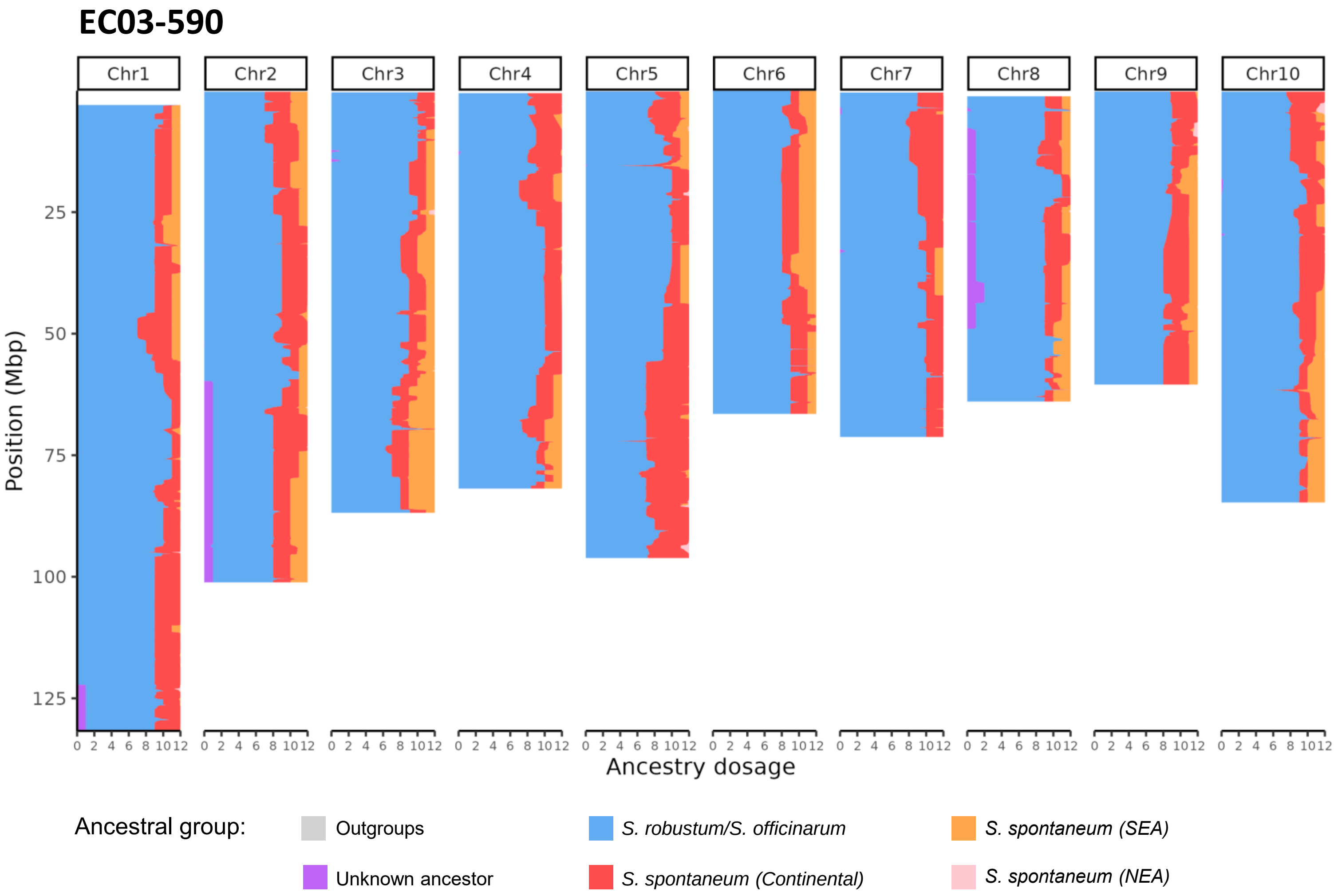

Local admixture of the modern cultivar EC03-590

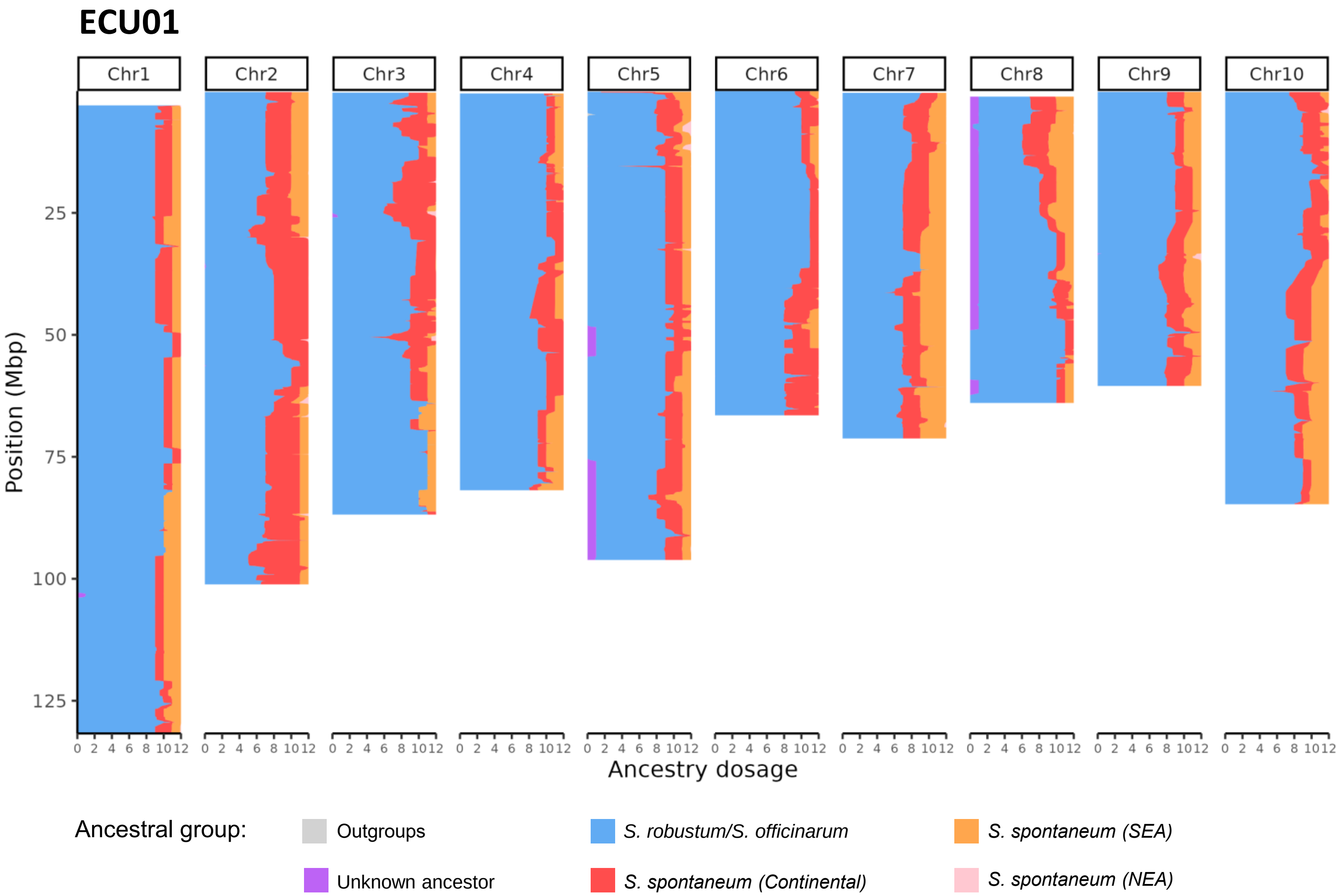

Local admixture of the modern cultivar ECU01

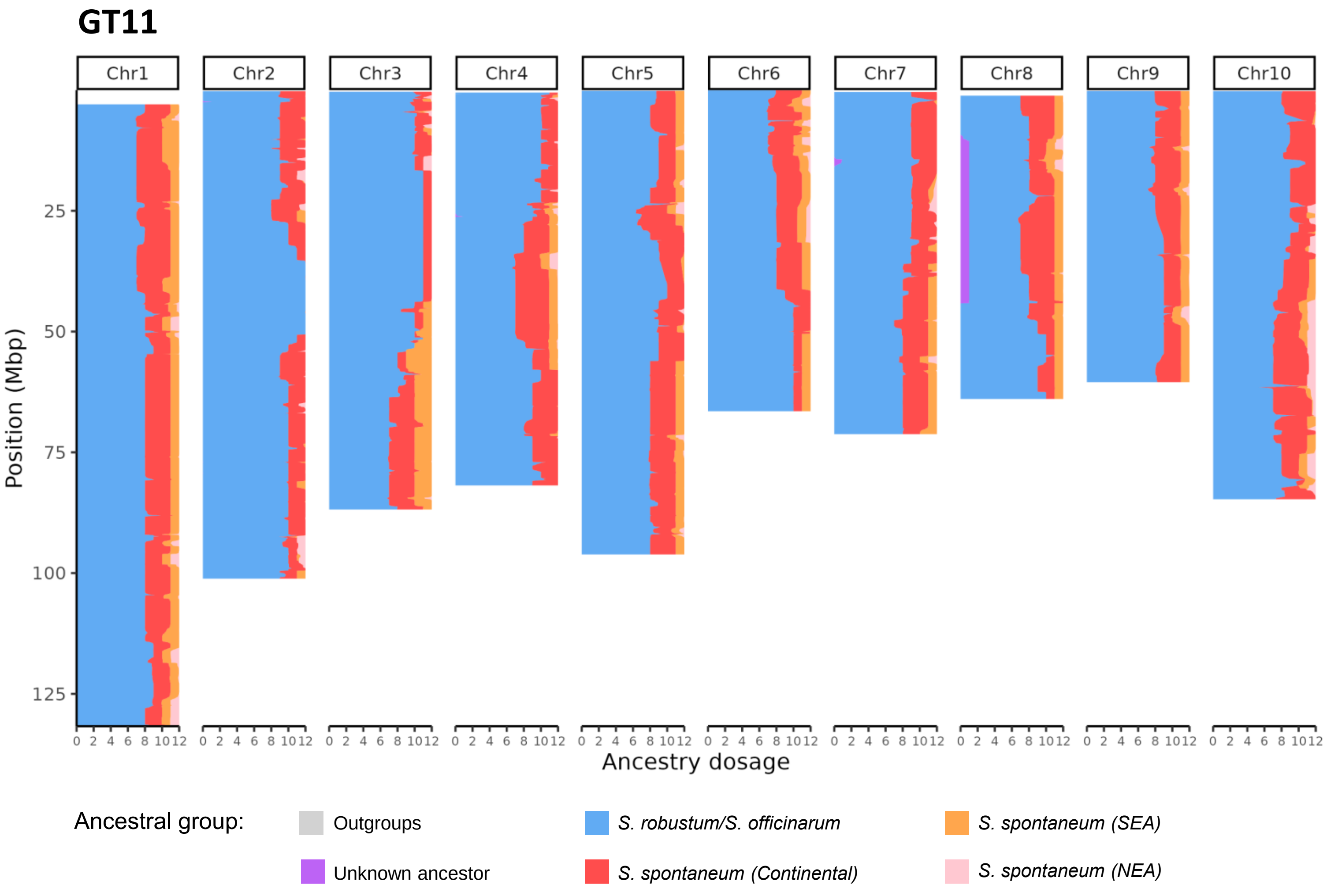

Local admixture of the modern cultivar GT11

GT42

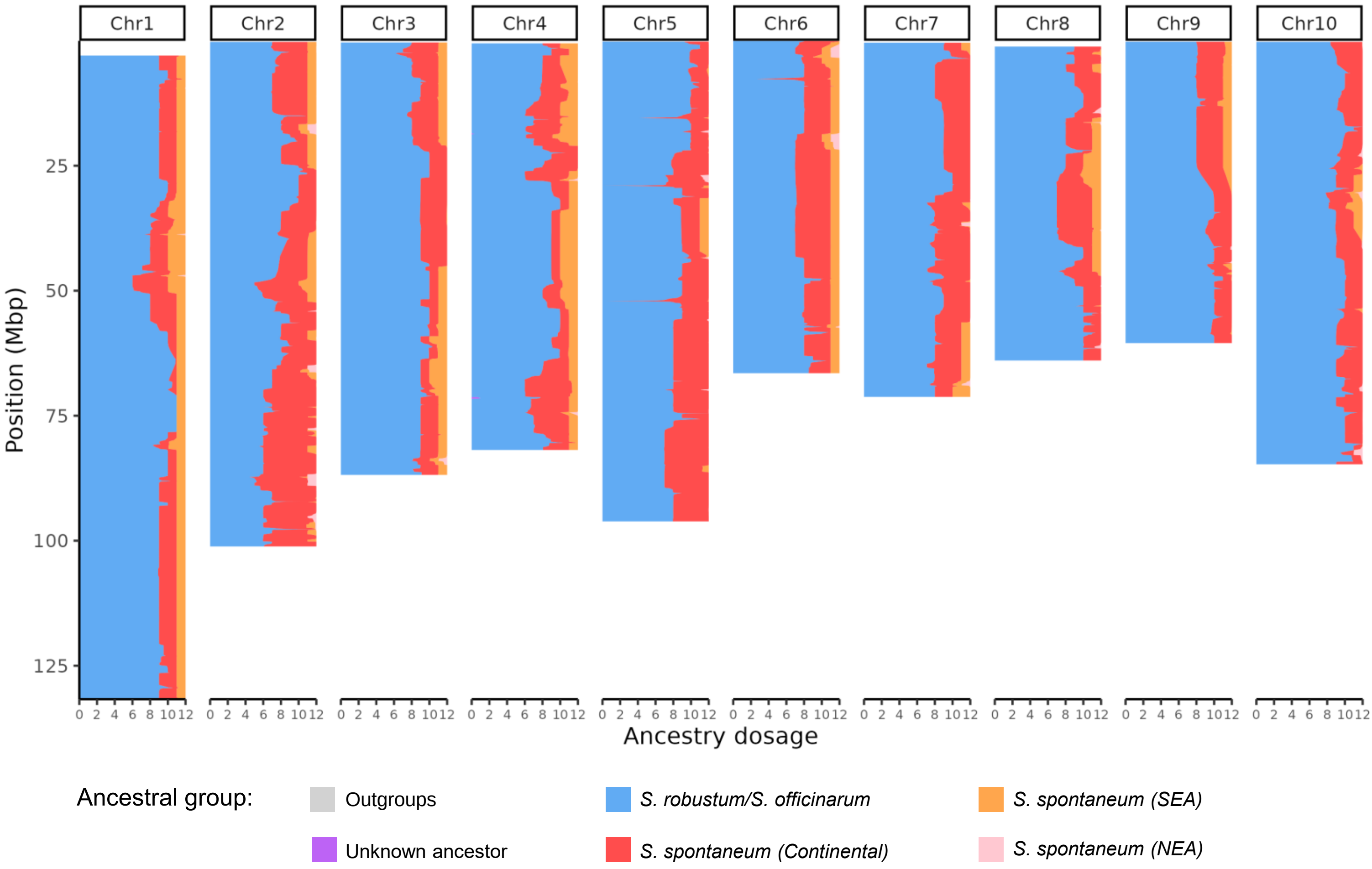

Local admixture of the modern cultivar GT42

H32-8560

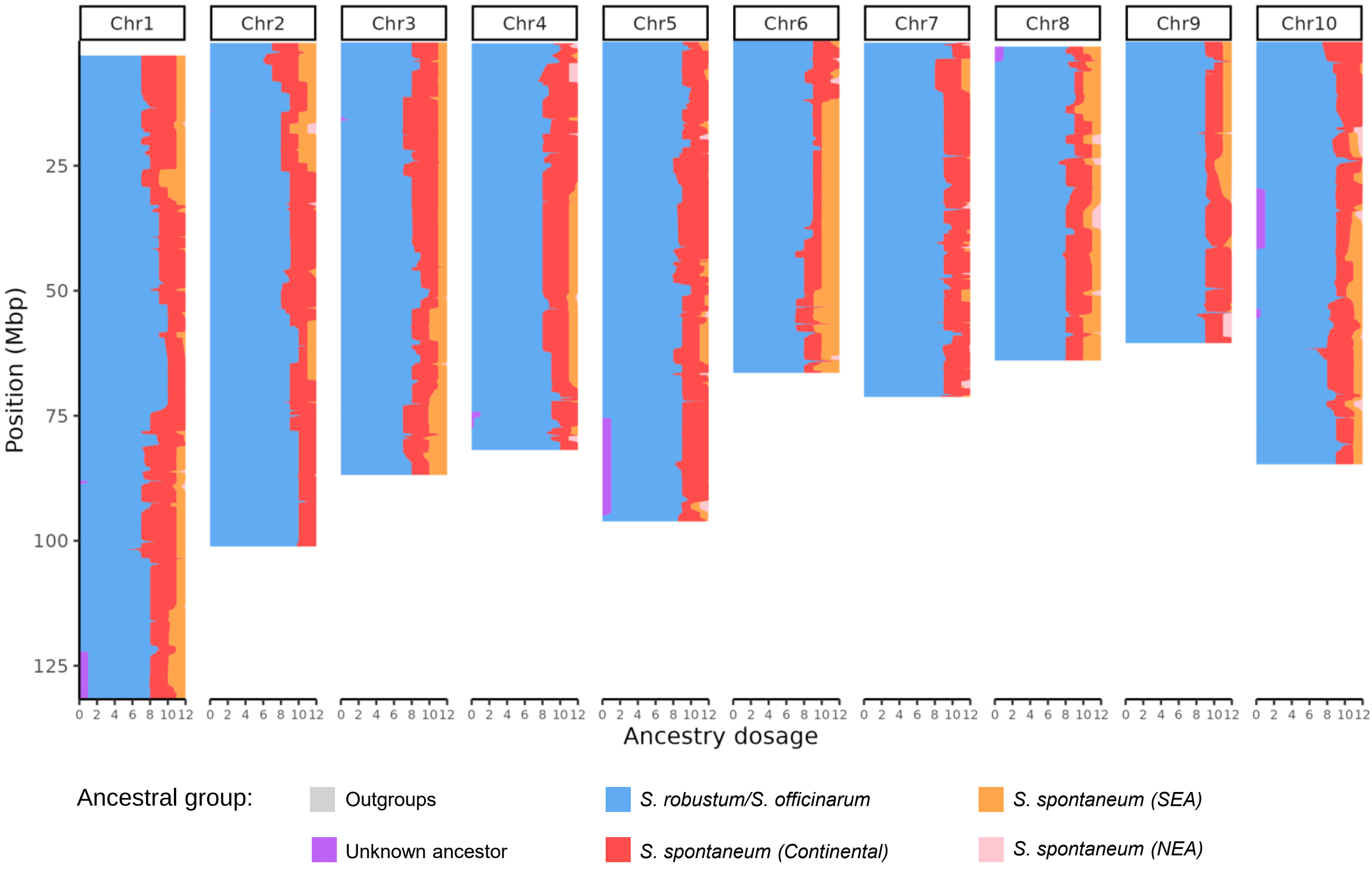

Local admixture of the modern cultivar H32-8560

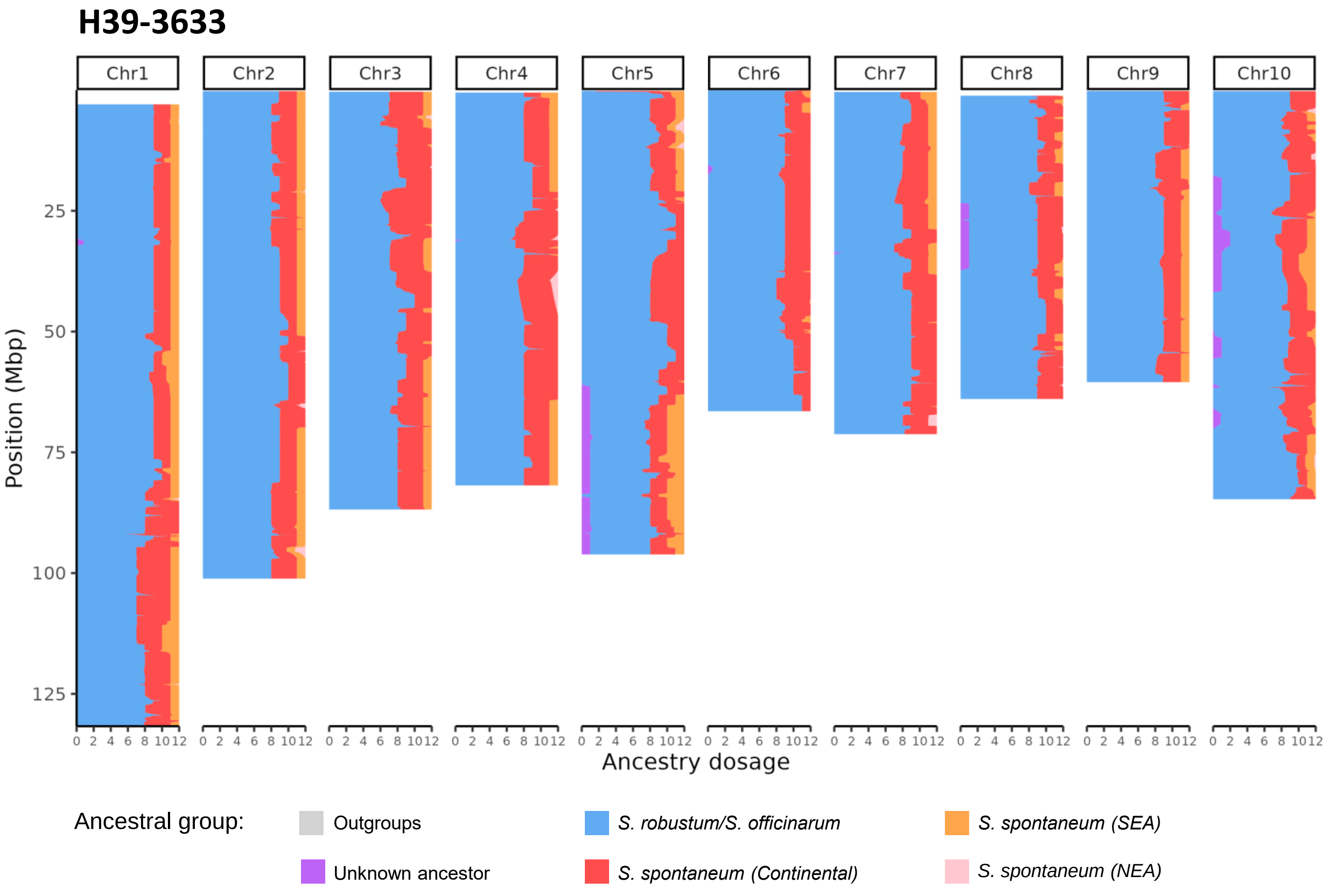

Local admixture of the modern cultivar H39-3633

H72-8597

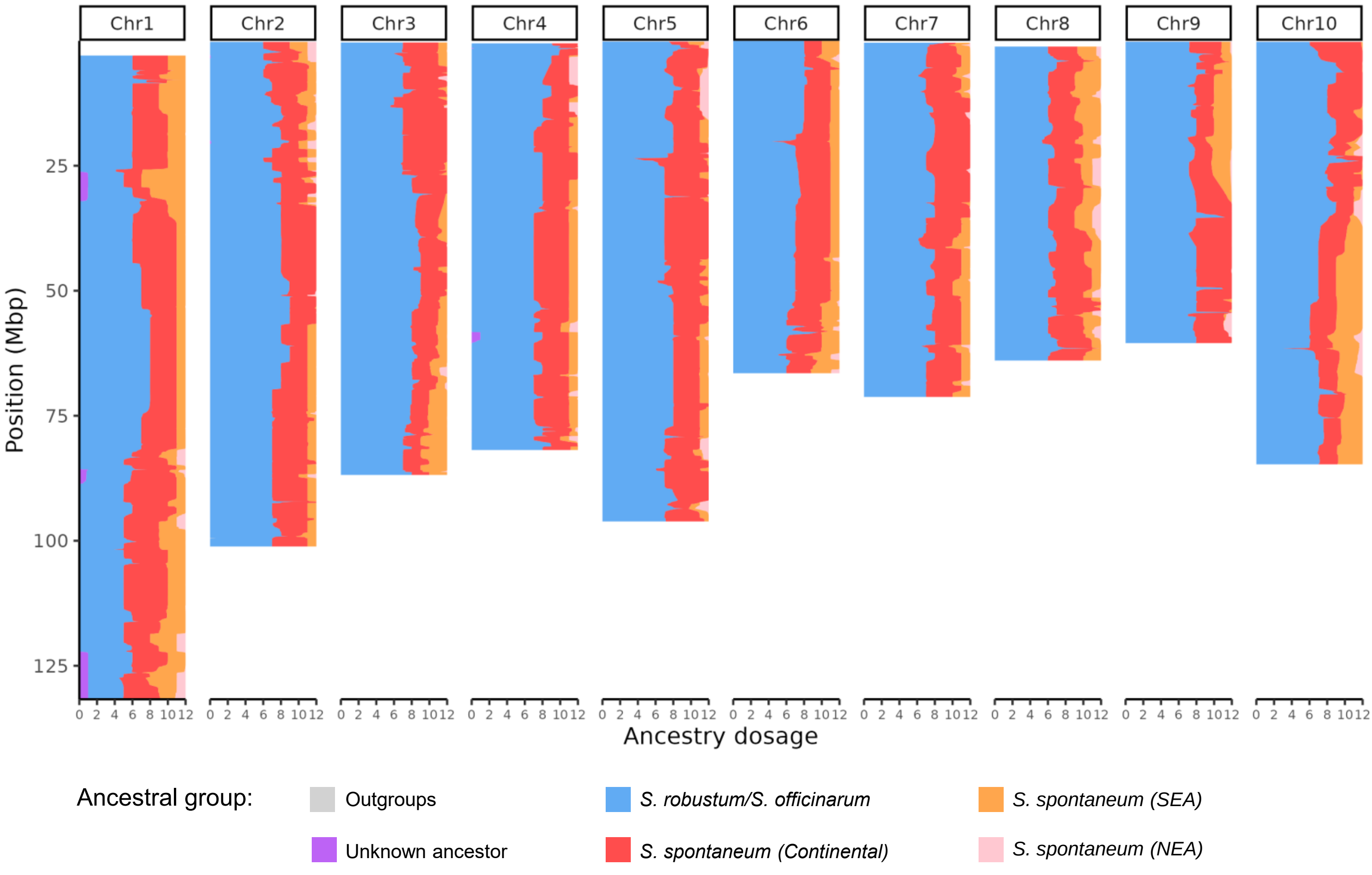

Local admixture of the modern cultivar H72-8597

KQ228

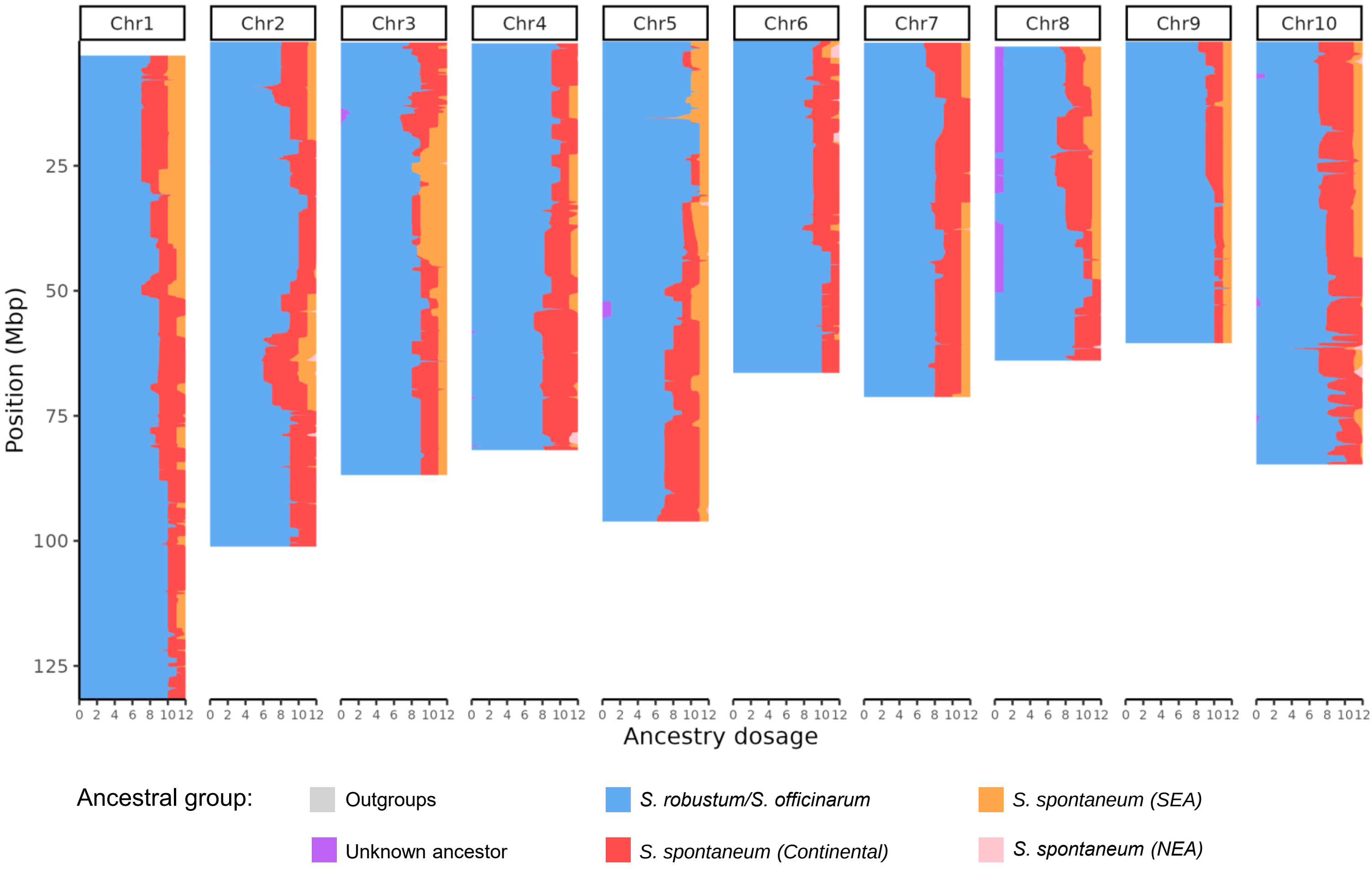

Local admixture of the modern cultivar KQ228

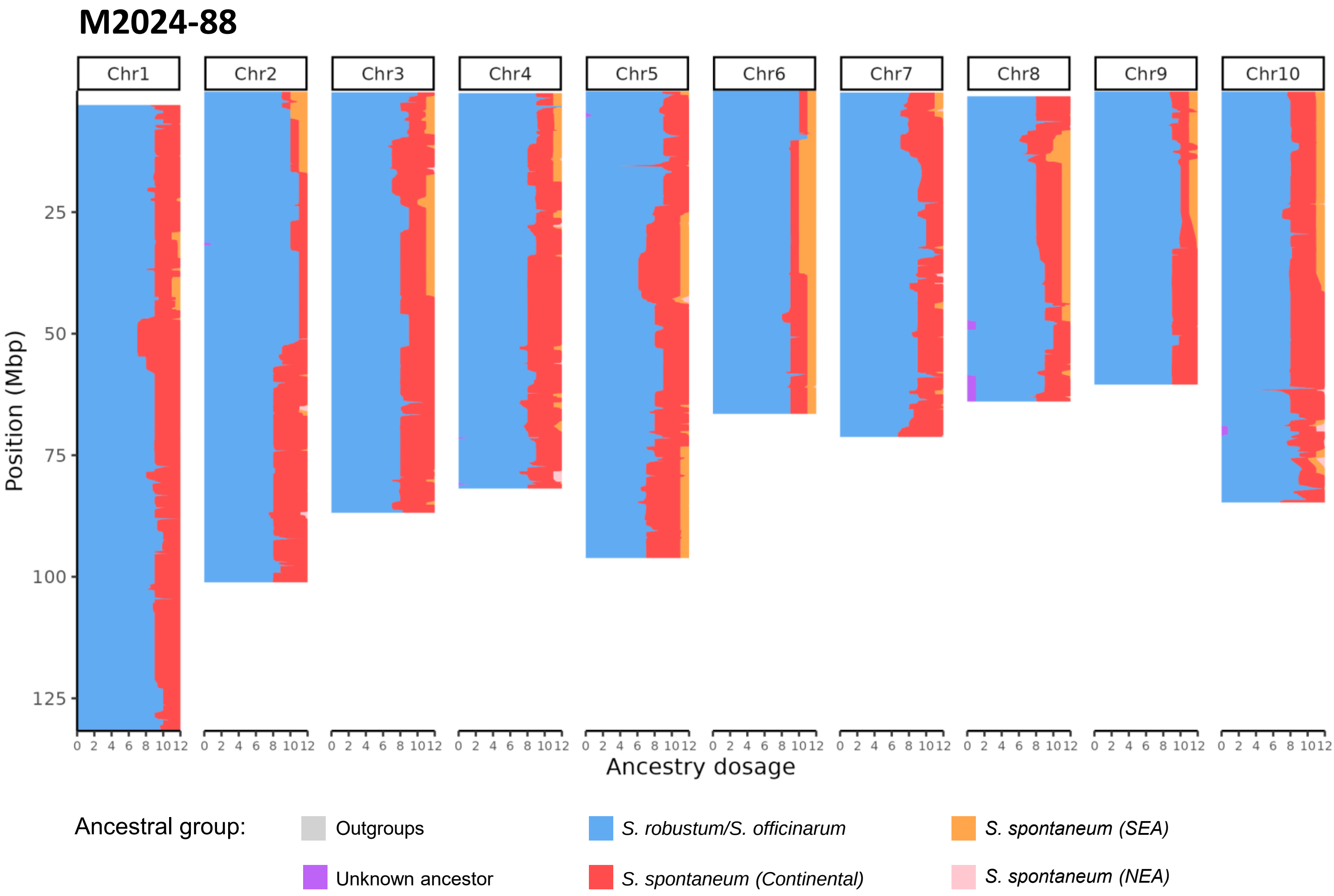

Local admixture of the modern cultivar M2024-88

M3035-66

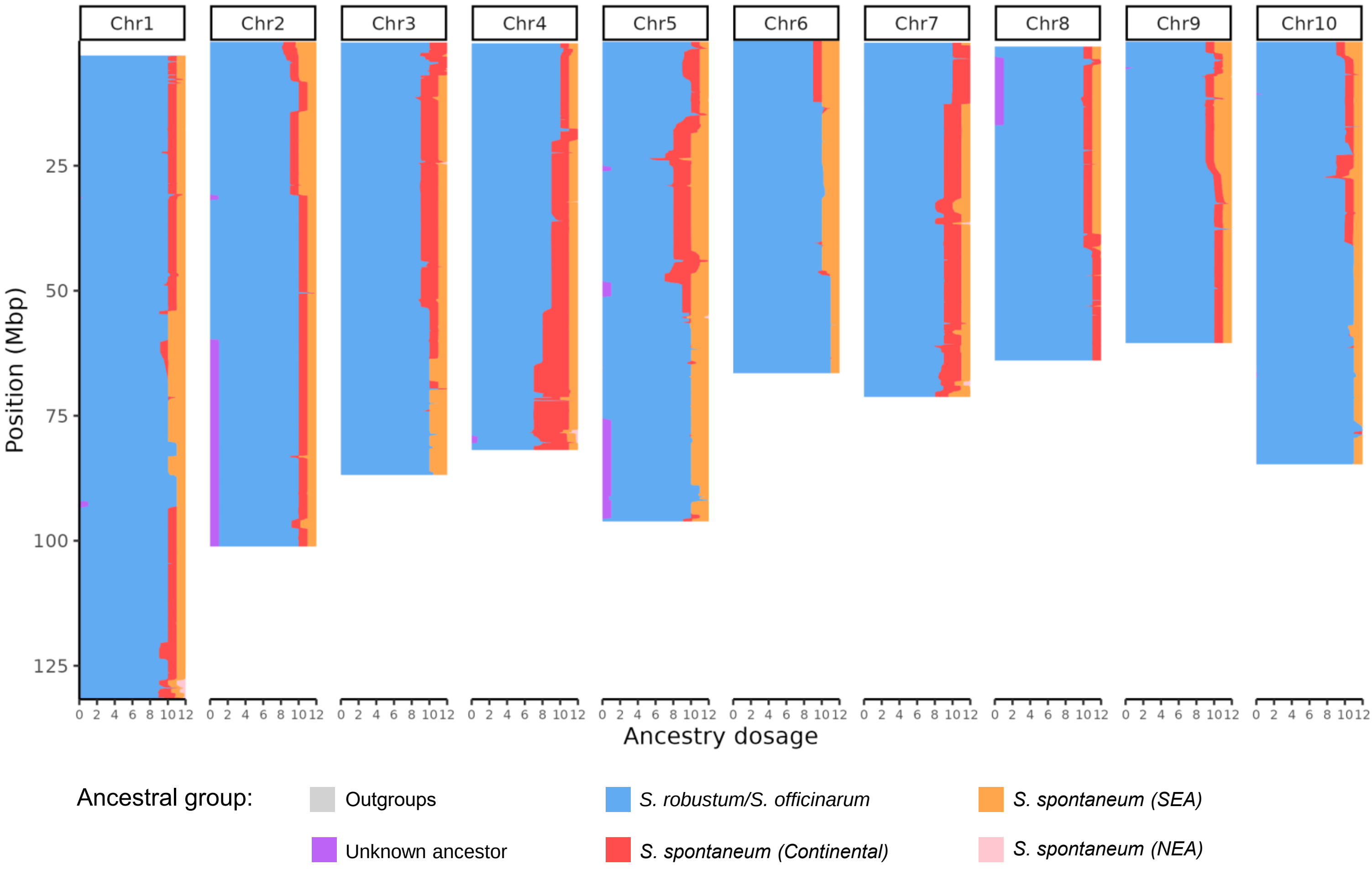

Local admixture of the modern cultivar M3035-66

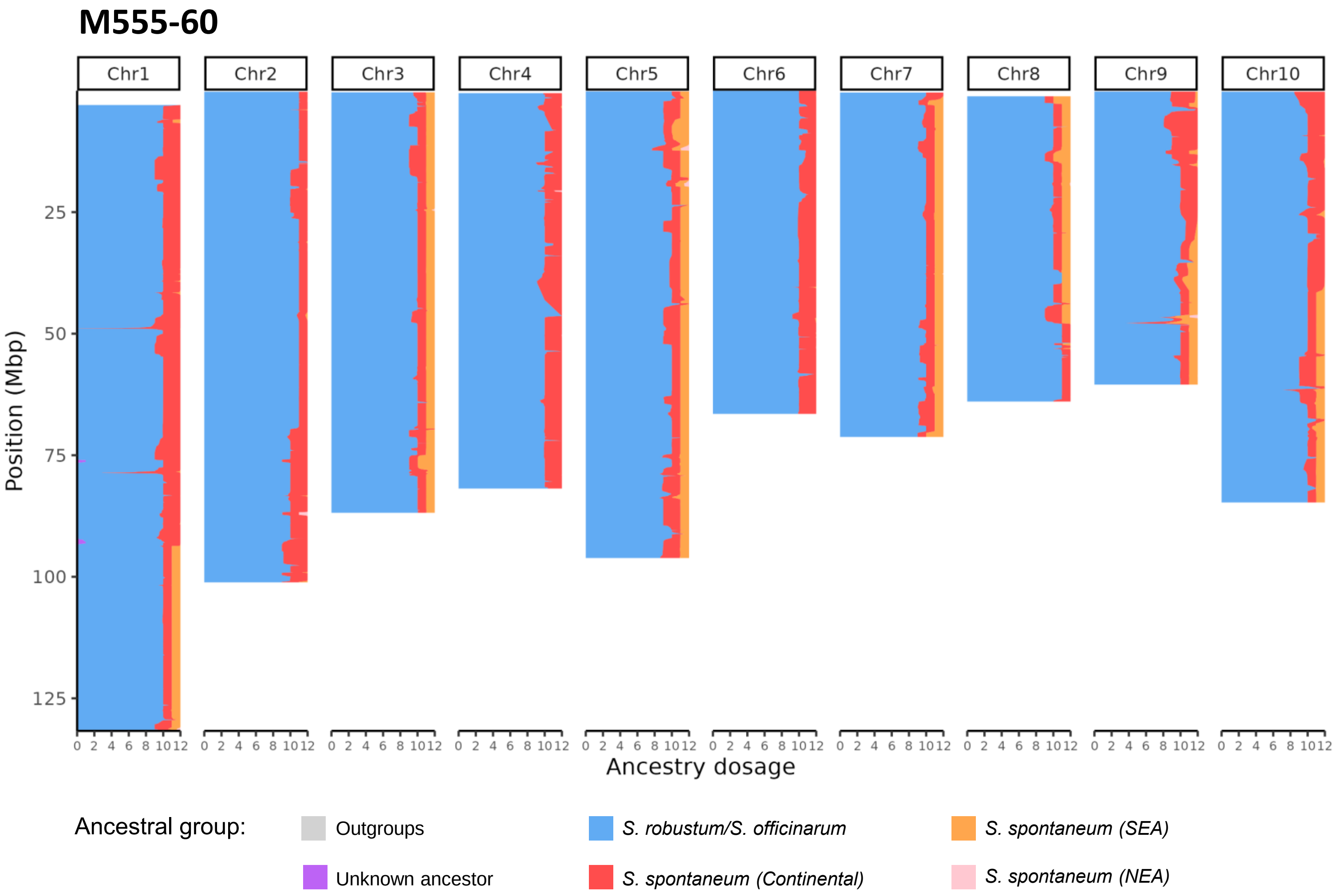

Local admixture of the modern cultivar M555-60

N10

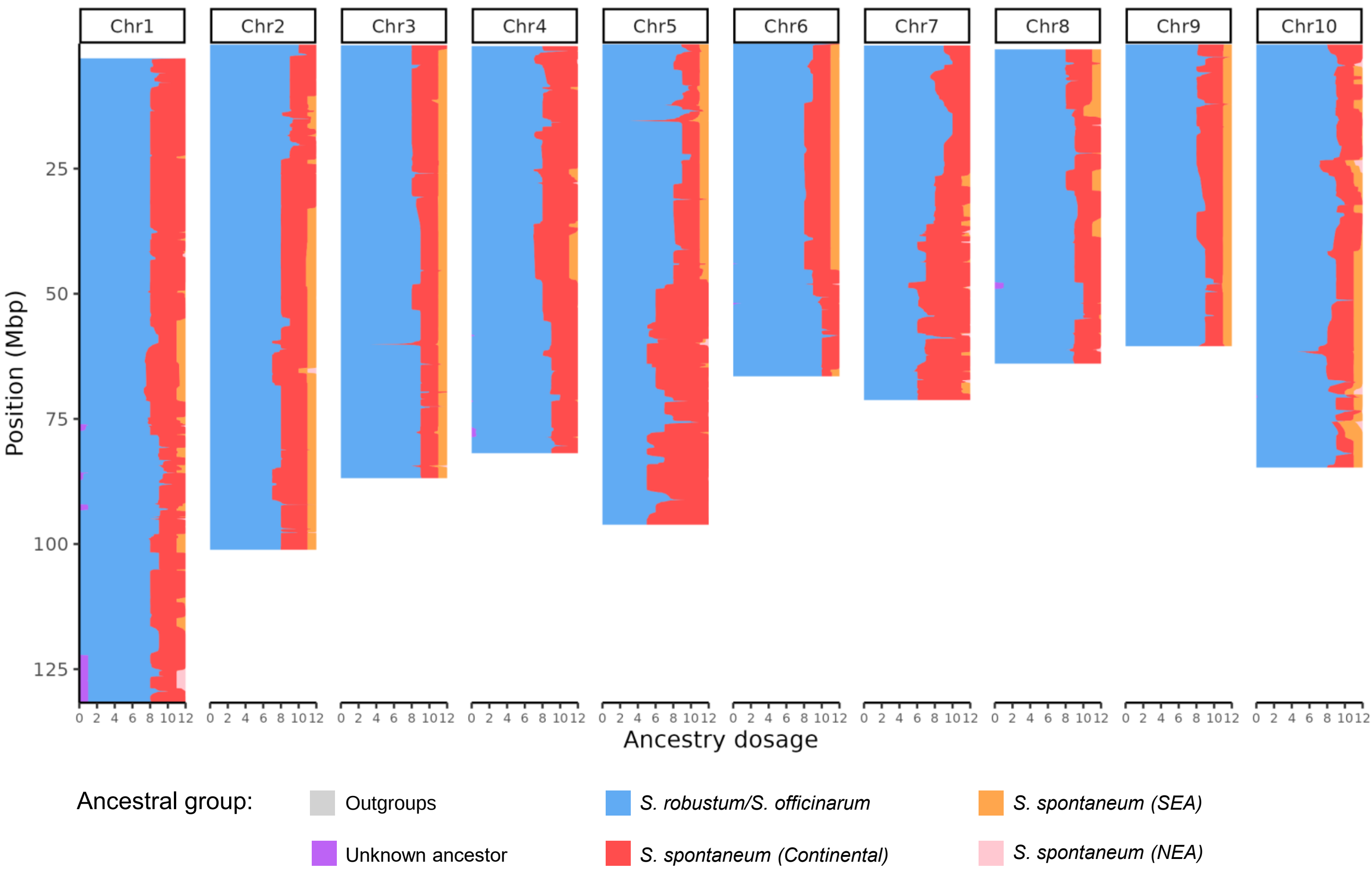

Local admixture of the modern cultivar N10

N55-805

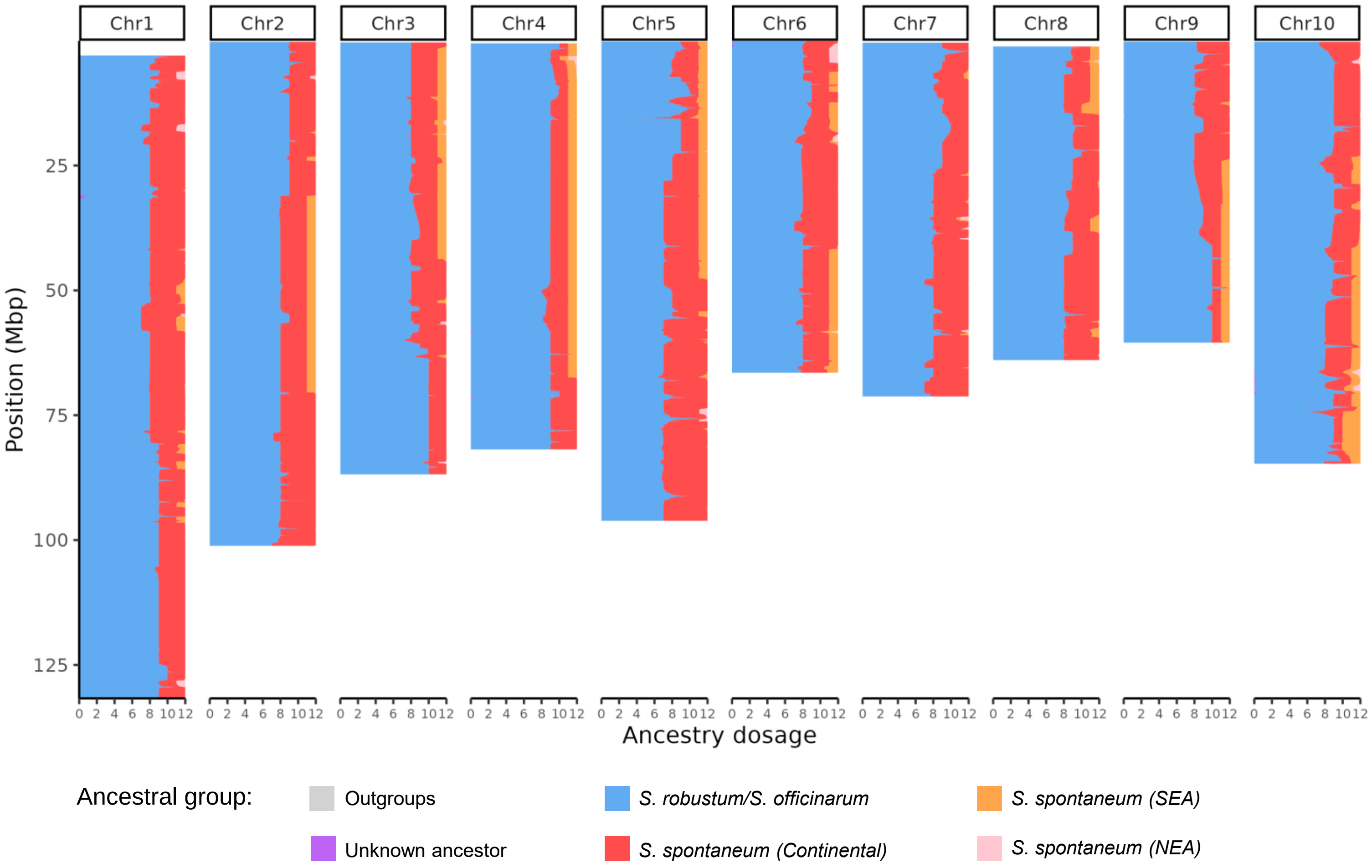

Local admixture of the modern cultivar N55-805

NA56-79

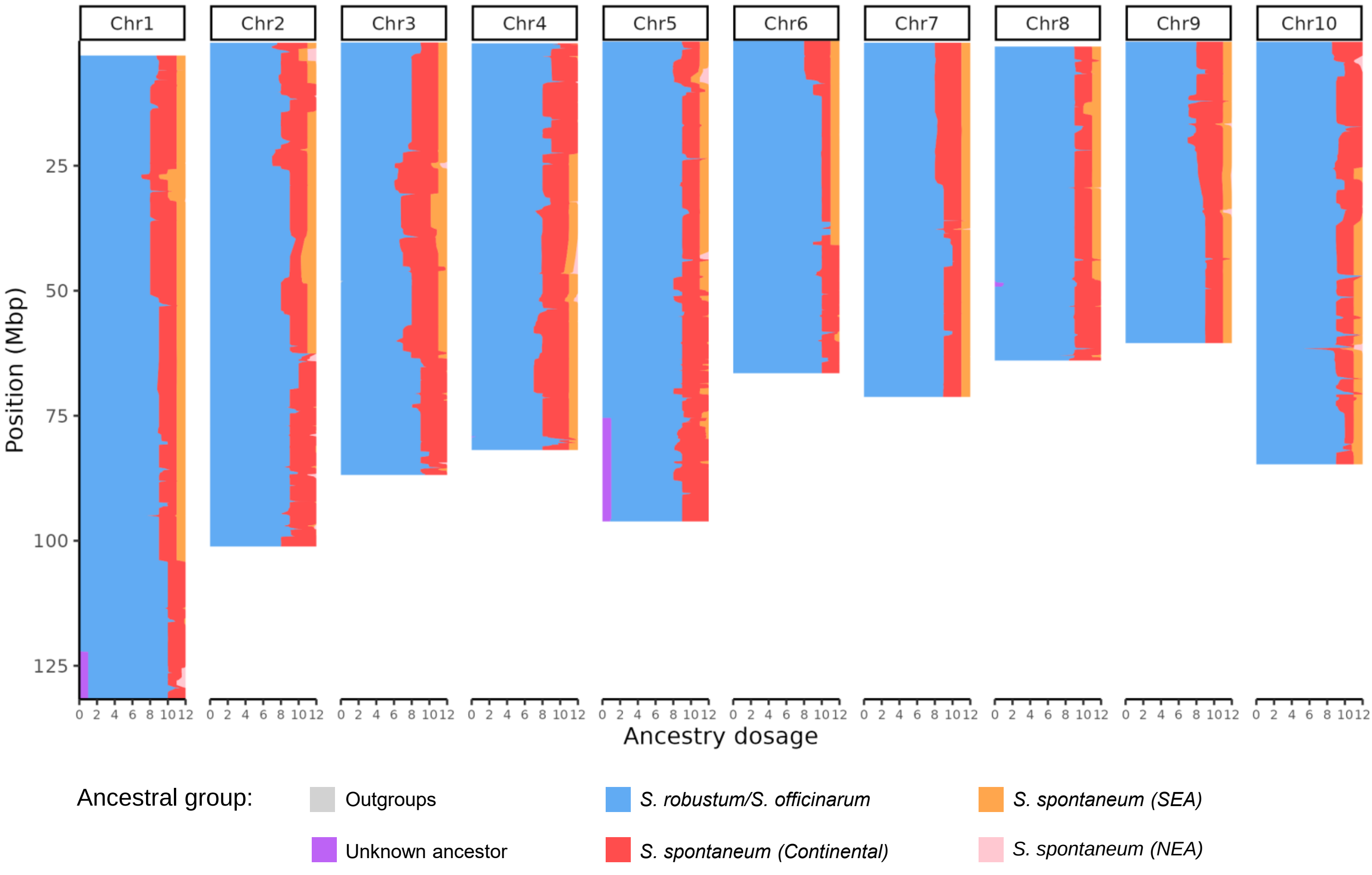

Local admixture of the modern cultivar NA56-79

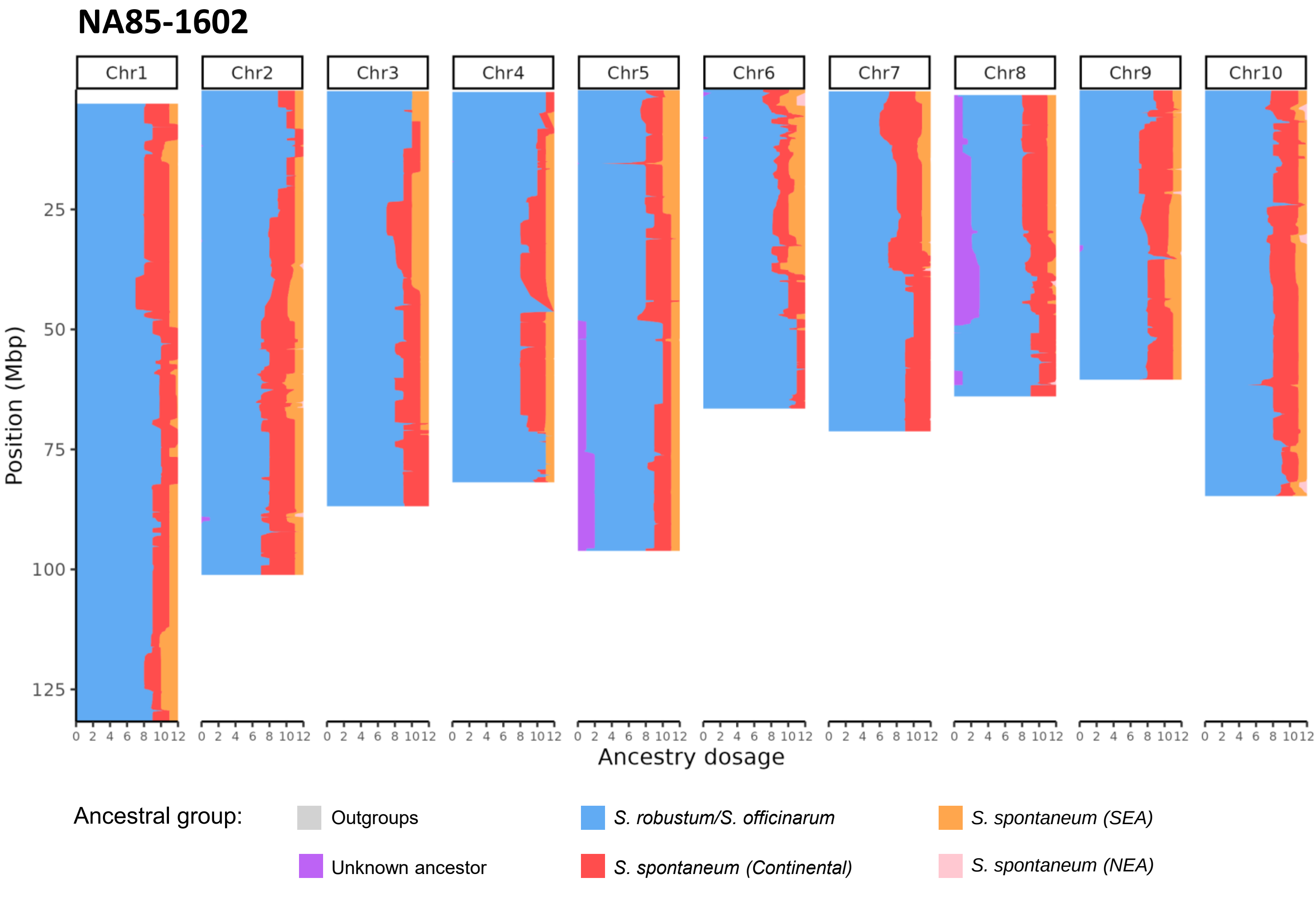

Local admixture of the modern cultivar NA85-1602

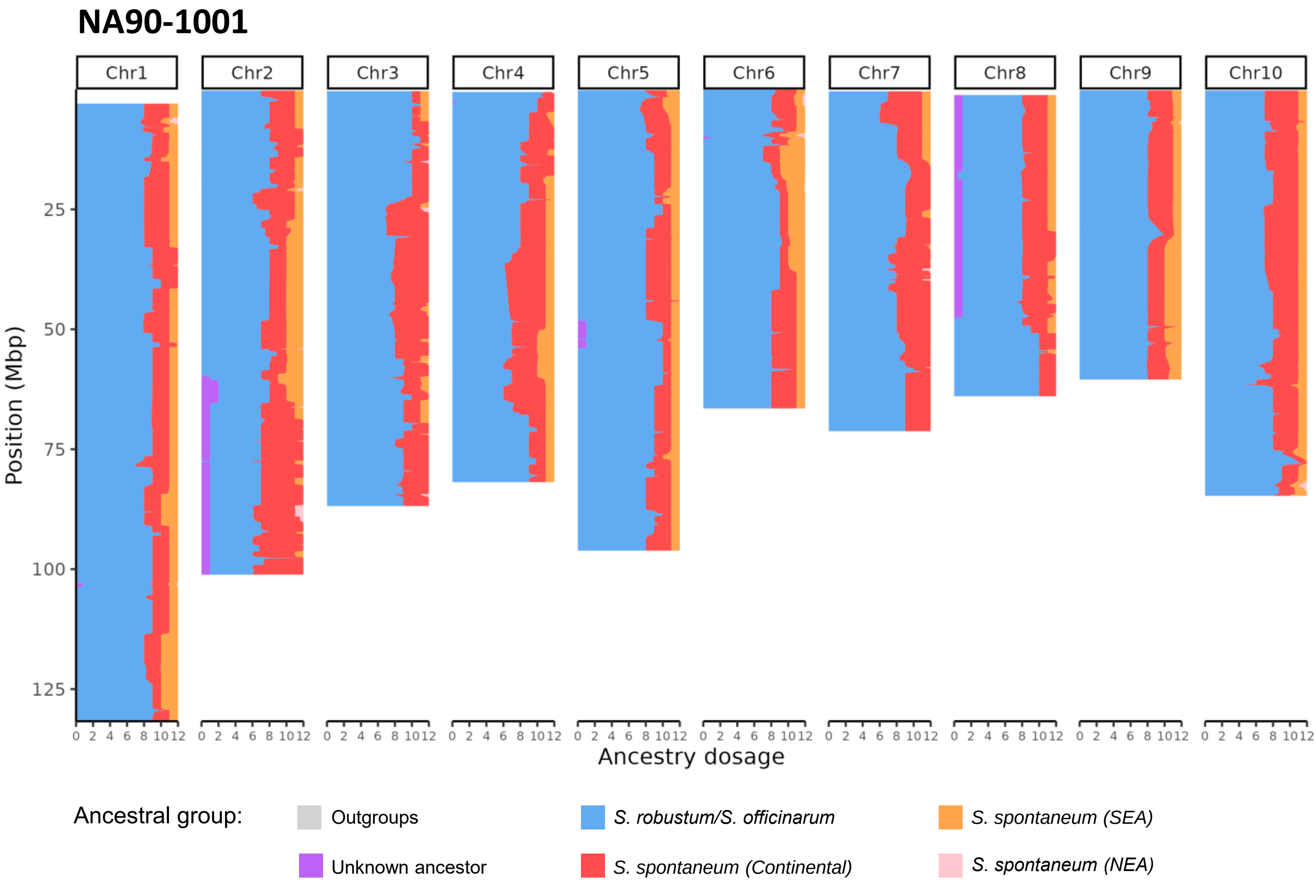

Local admixture of the modern cultivar NA90-1001

NA03-3300

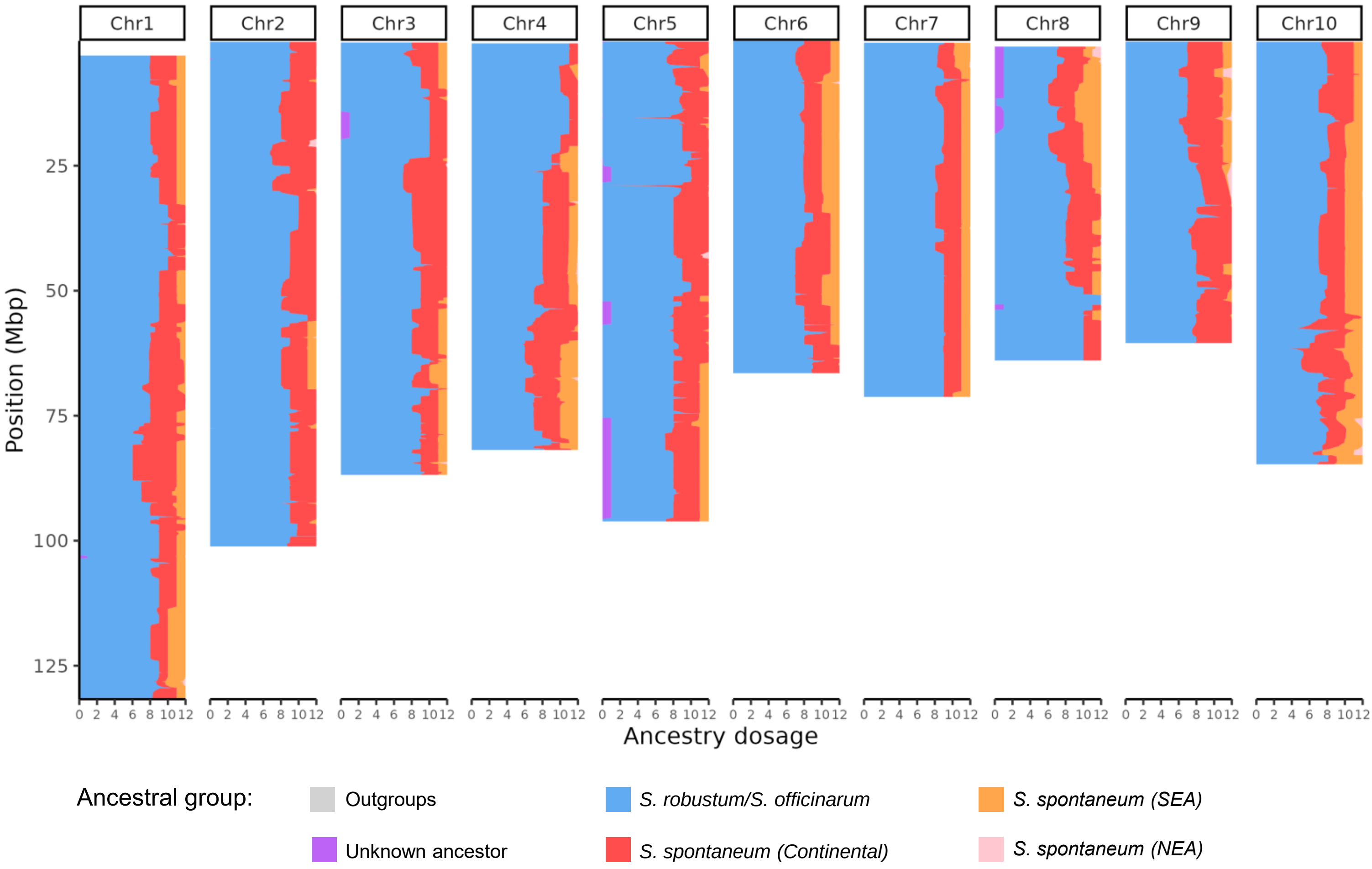

Local admixture of the modern cultivar NA03-3300

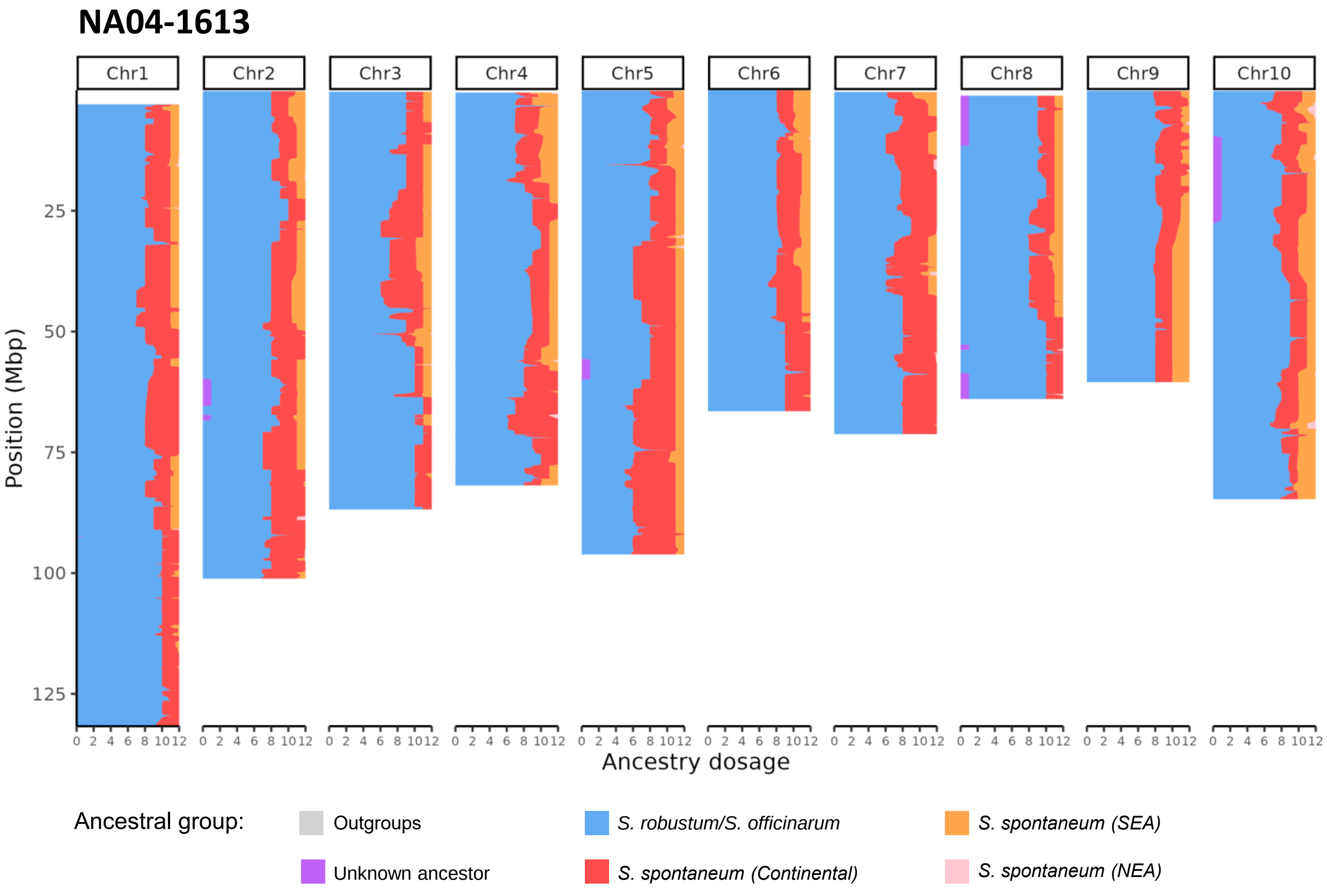

Local admixture of the modern cultivar NA04-1613

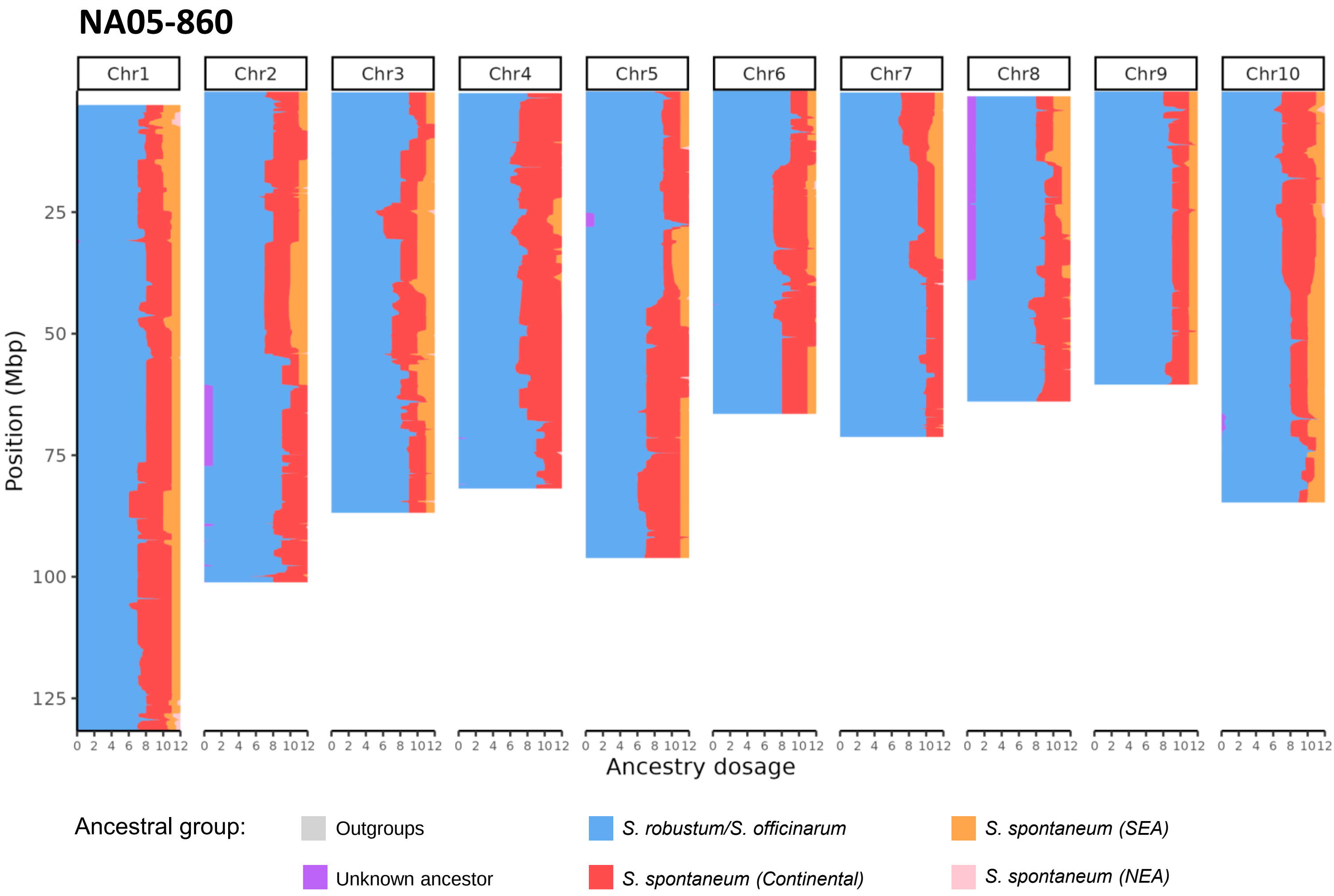

Local admixture of the modern cultivar NA05-860

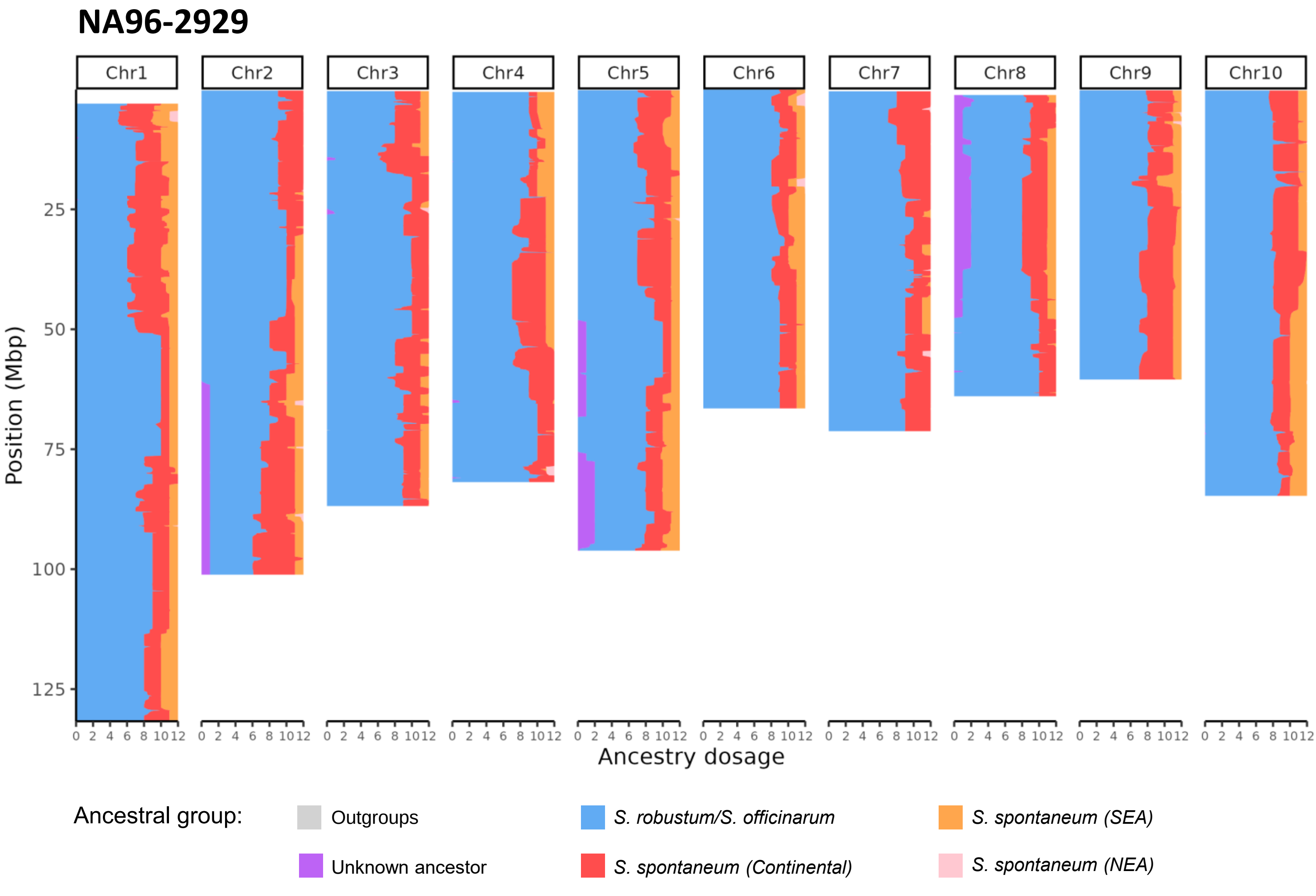

Local admixture of the modern cultivar NA96-2929

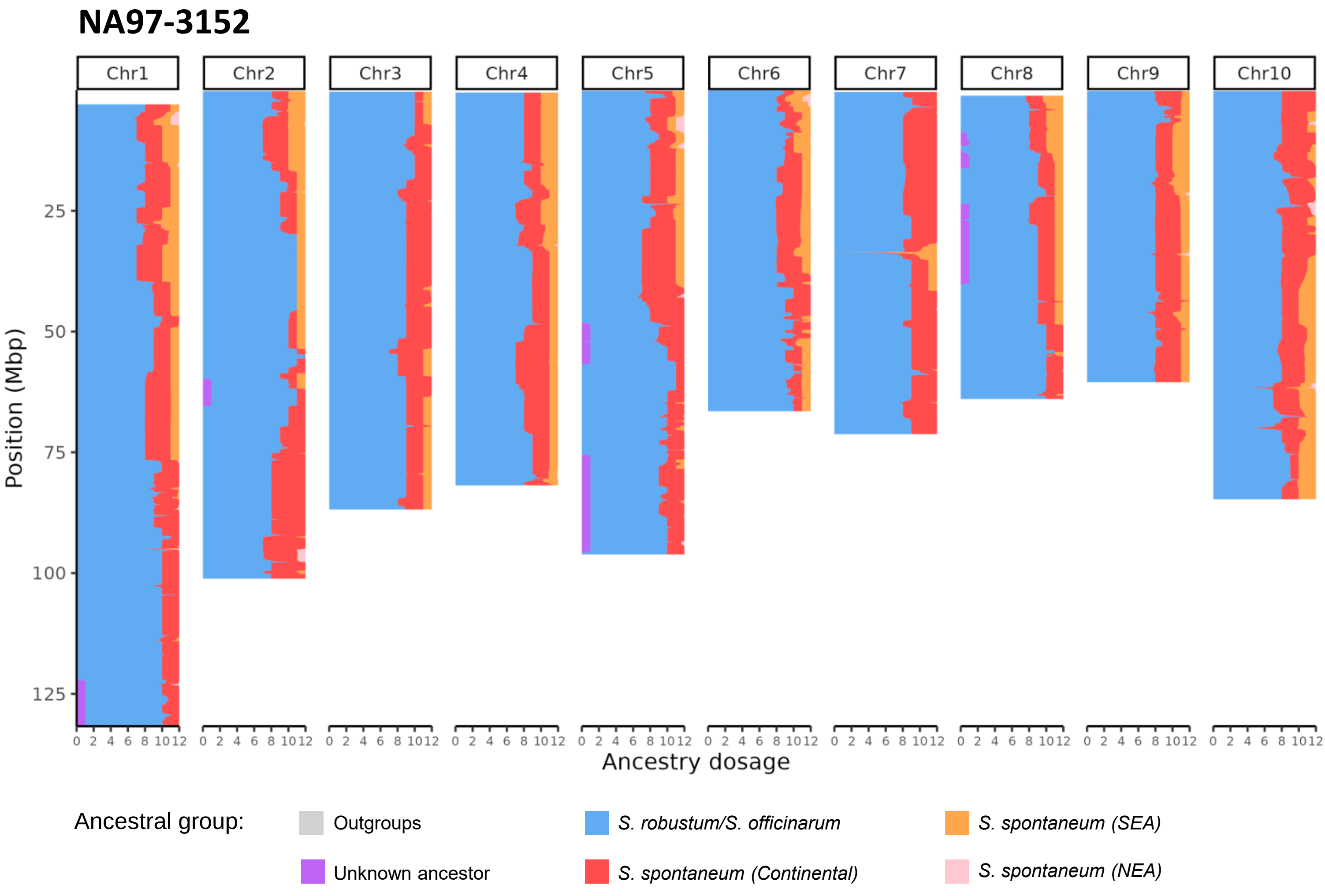

Local admixture of the modern cultivar NA97-3152

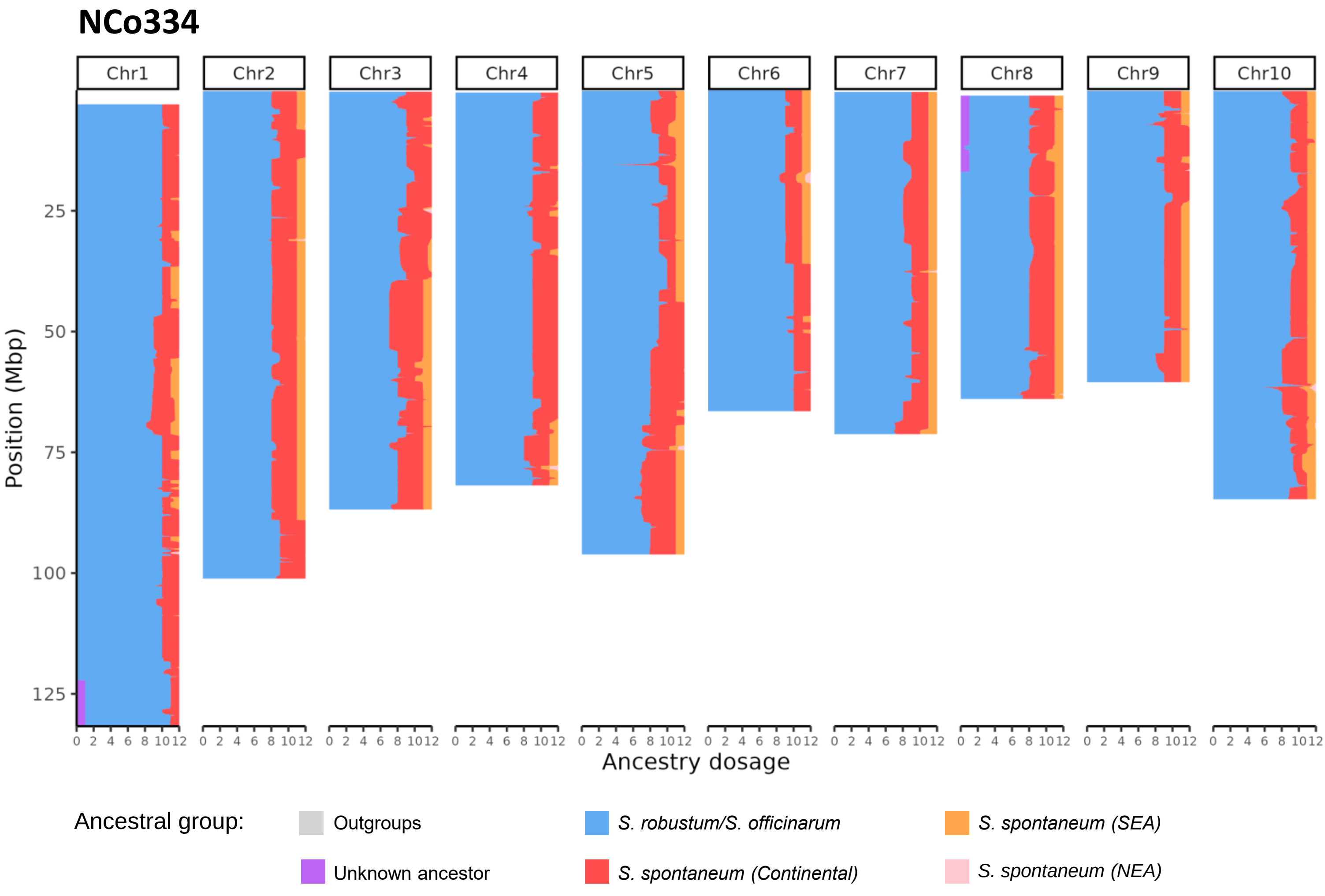

Local admixture of the modern cultivar NCo334

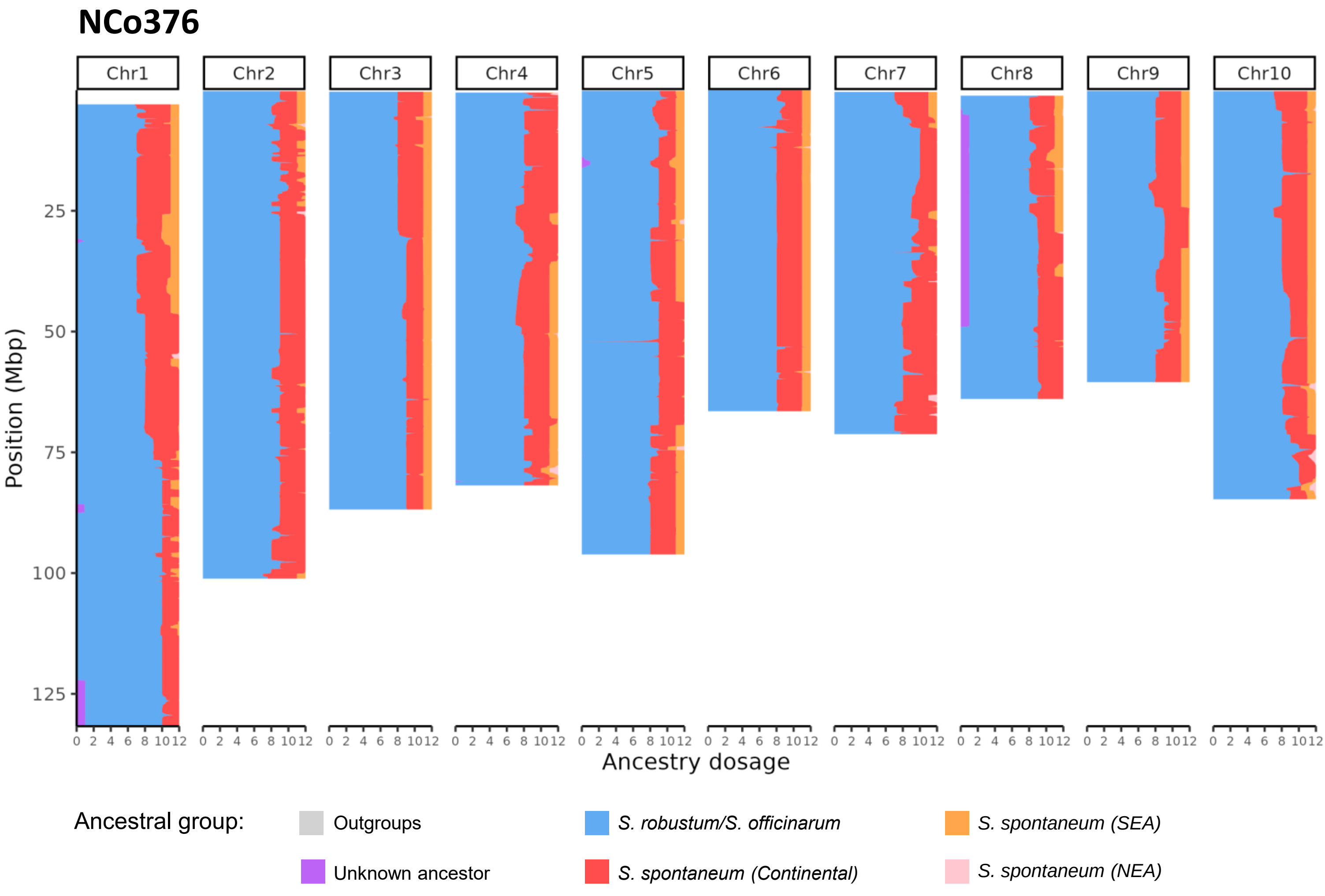

Local admixture of the modern cultivar NCo376

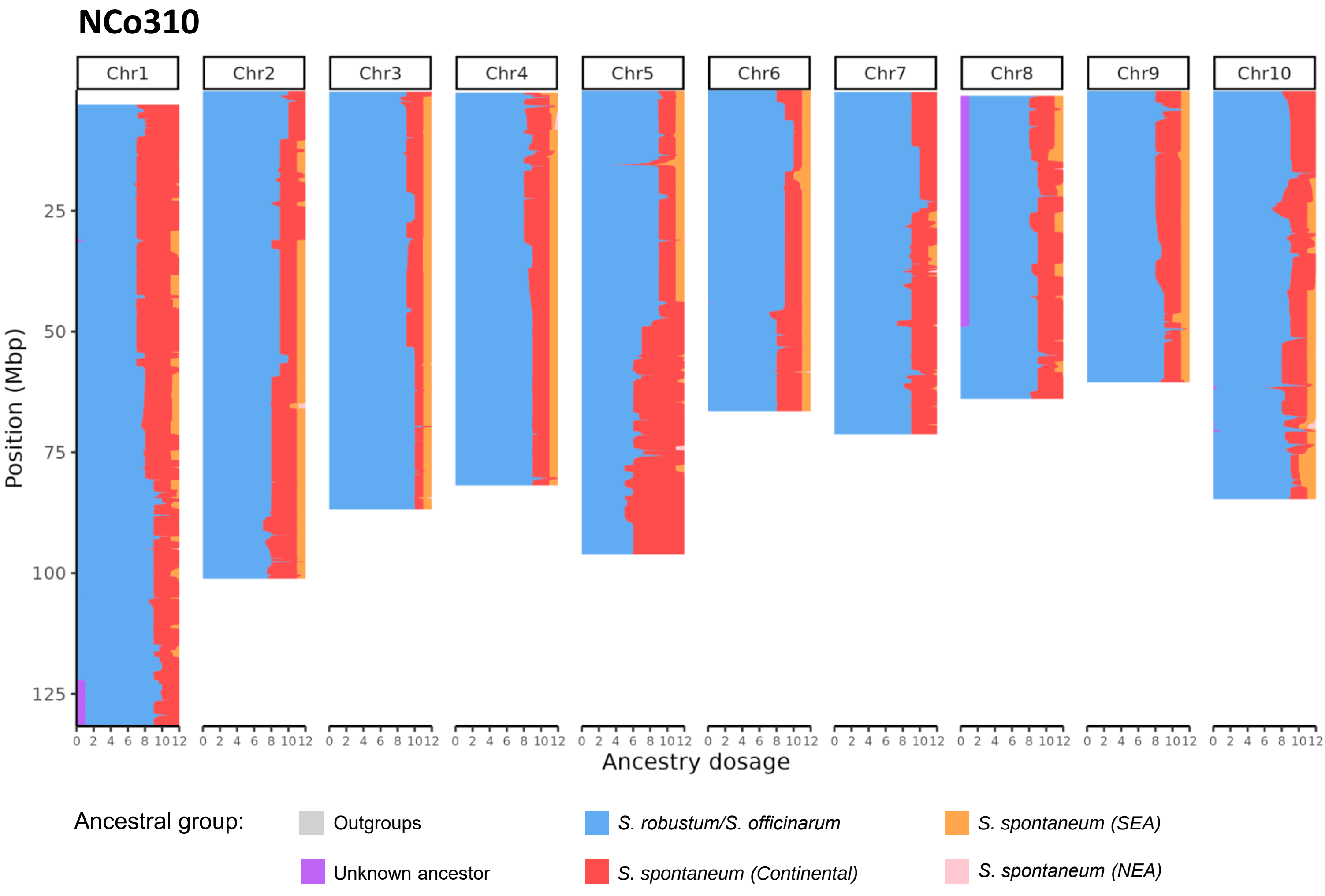

Local admixture of the modern cultivar NCo310

Ni27

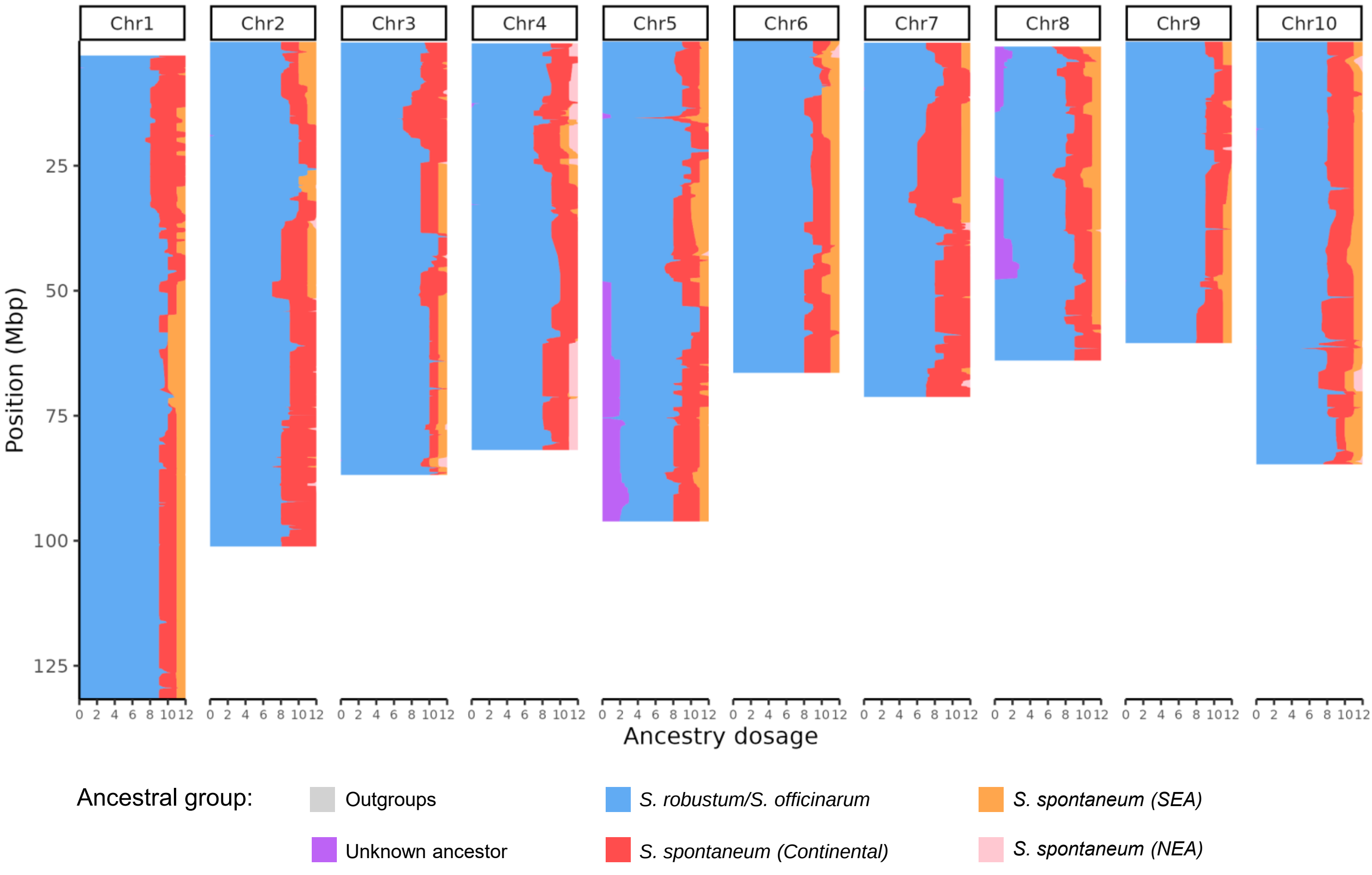

Local admixture of the modern cultivar Ni27

Ni28

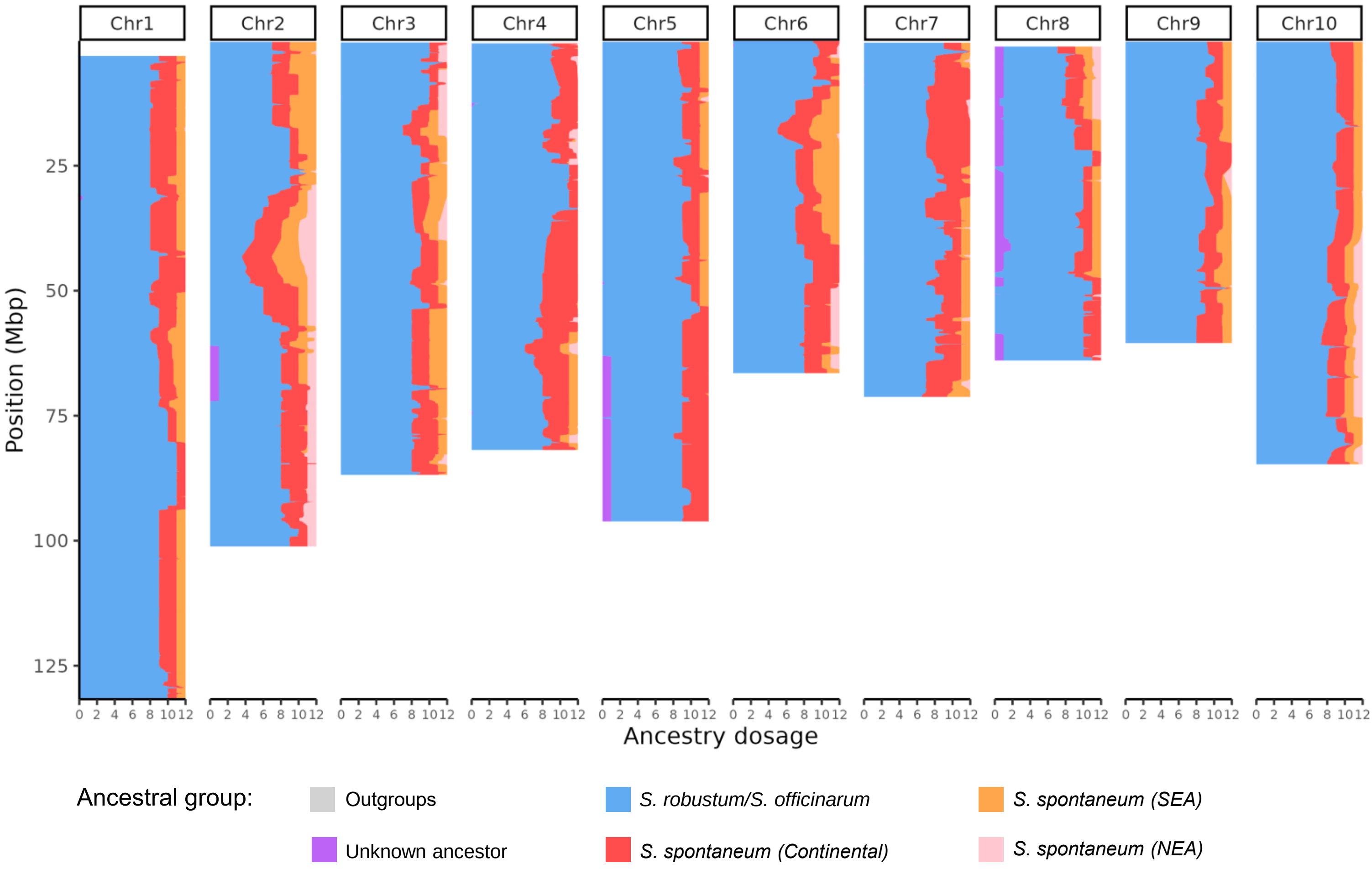

Local admixture of the modern cultivar Ni27

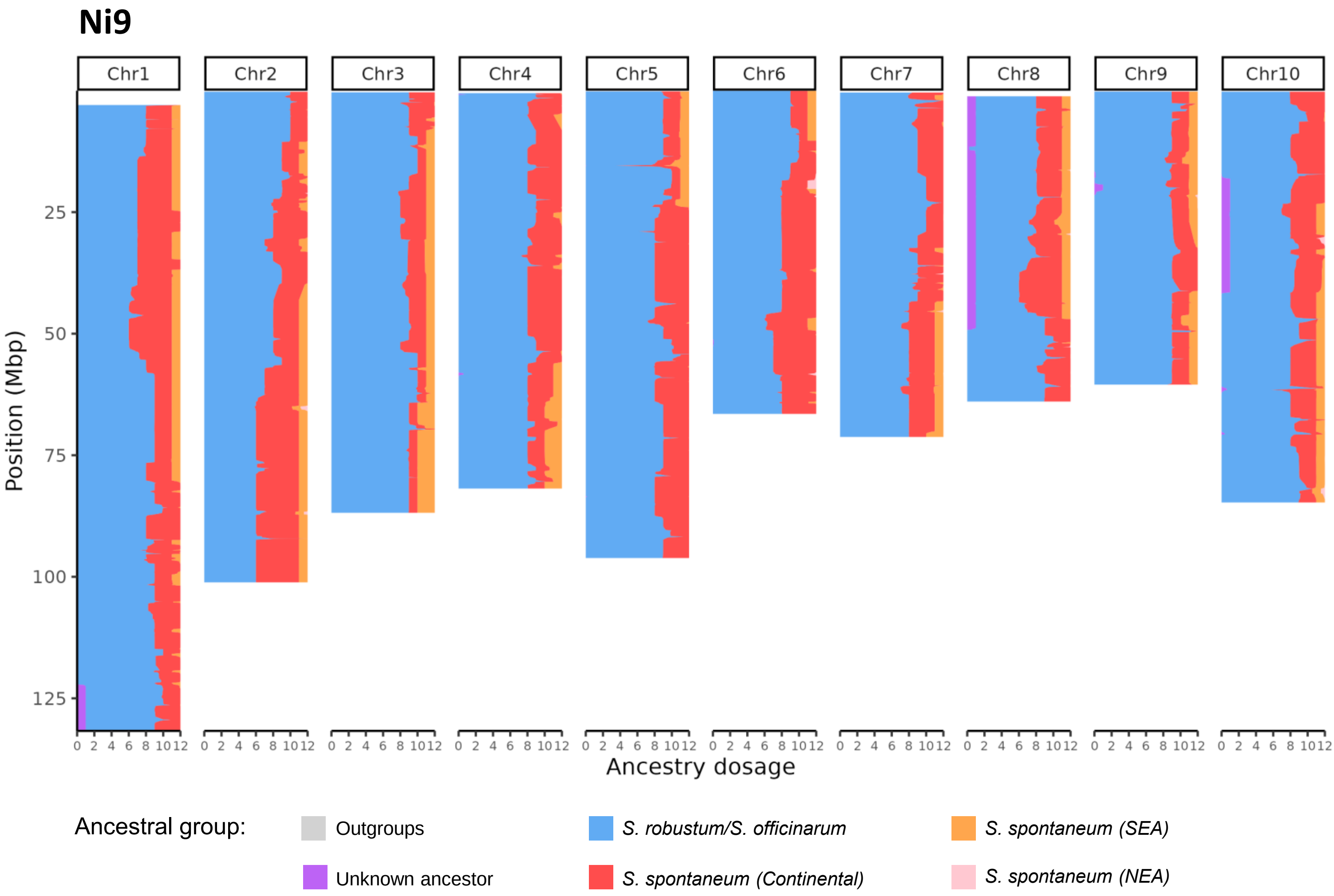

Local admixture of the modern cultivar Ni9

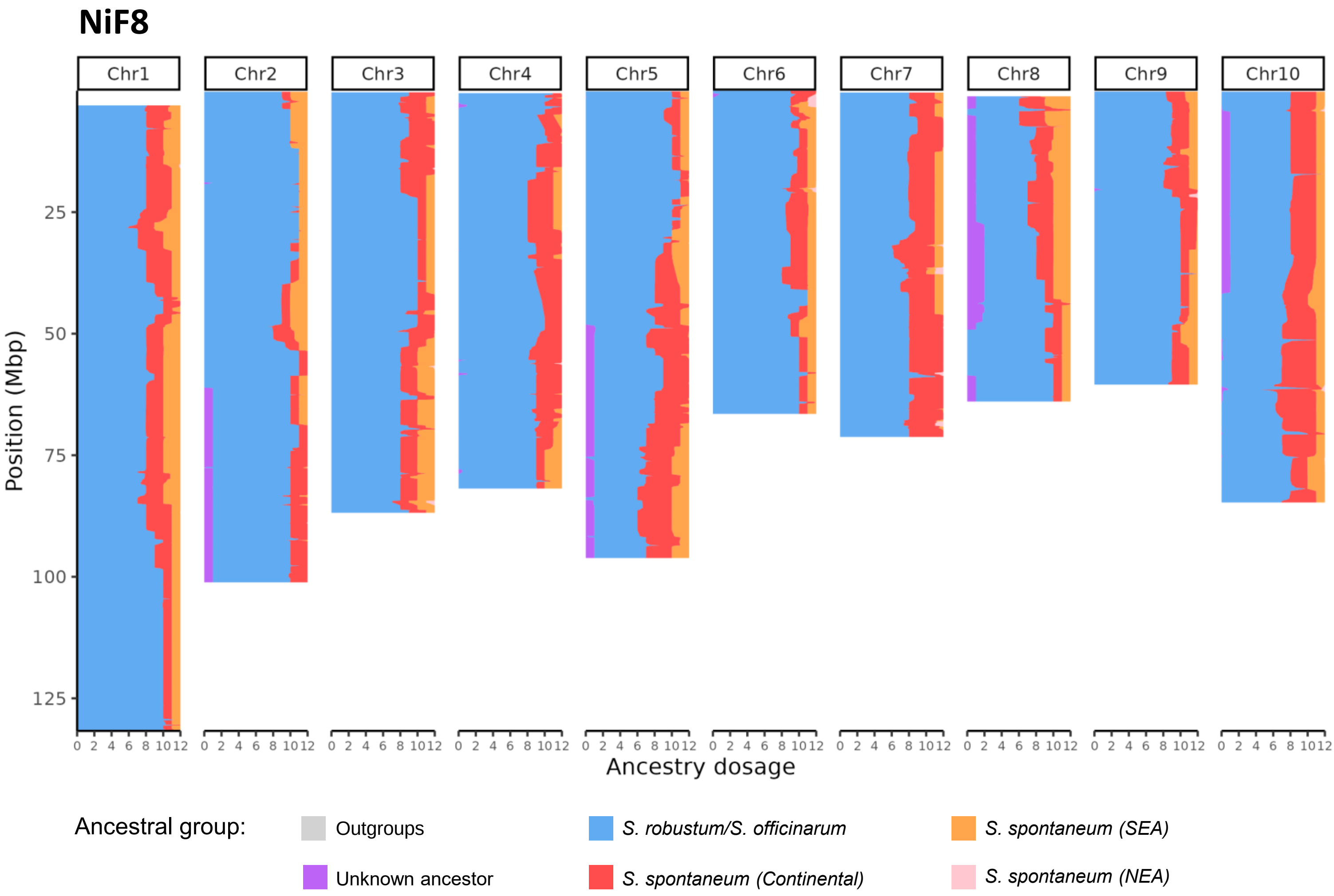

Local admixture of the modern cultivar NiF8

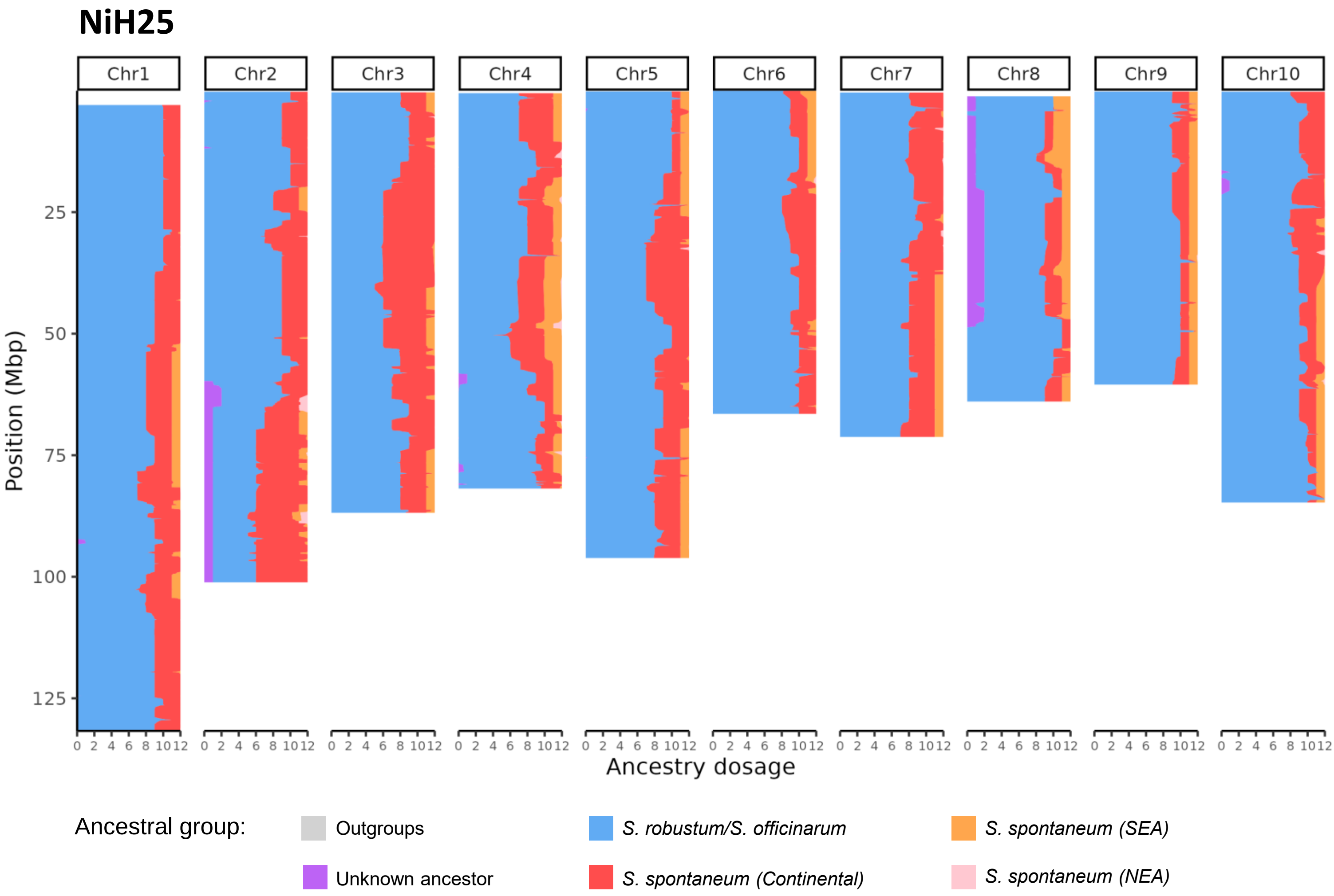

Local admixture of the modern cultivar NiH25

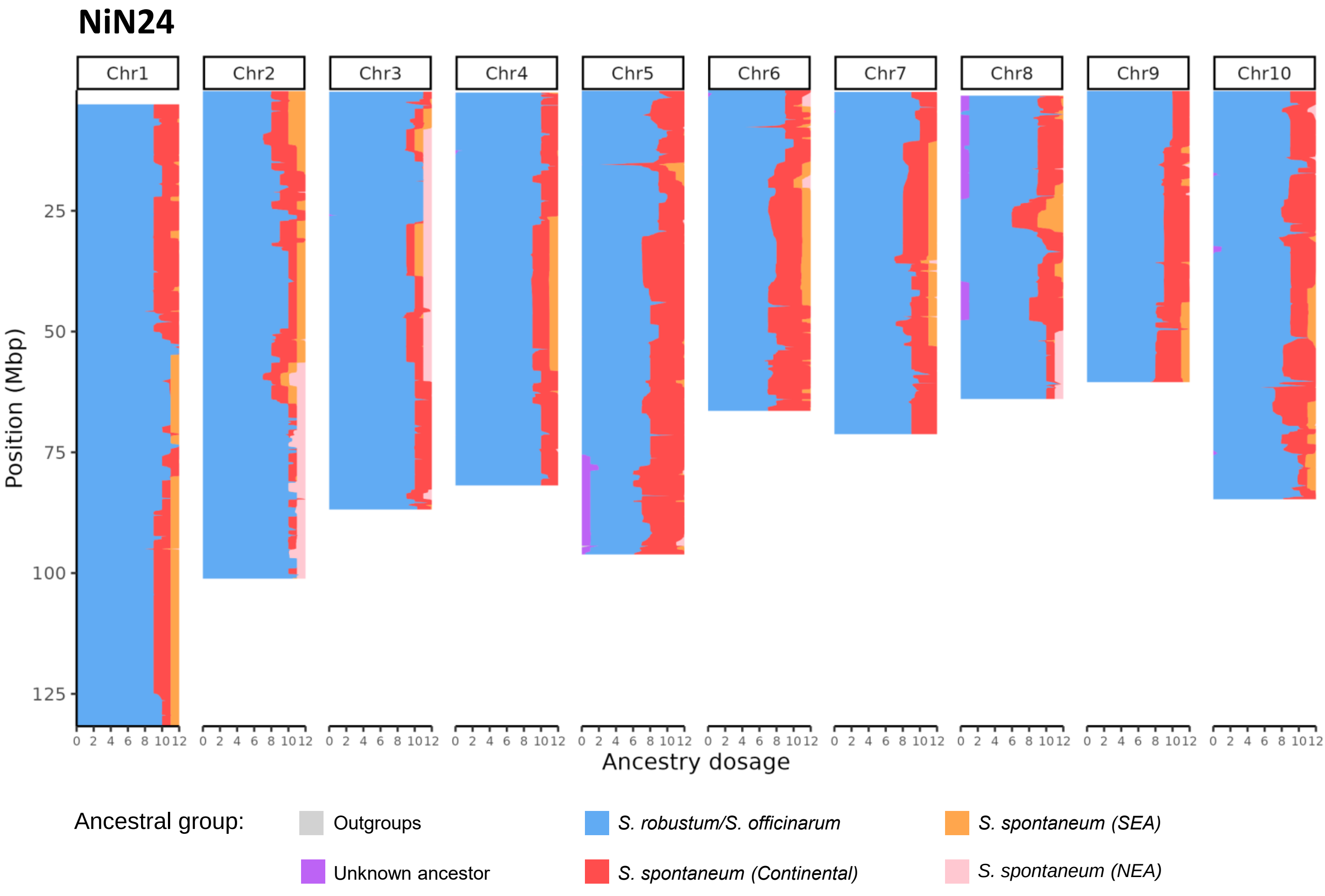

Local admixture of the modern cultivar NiN24

NiTn18

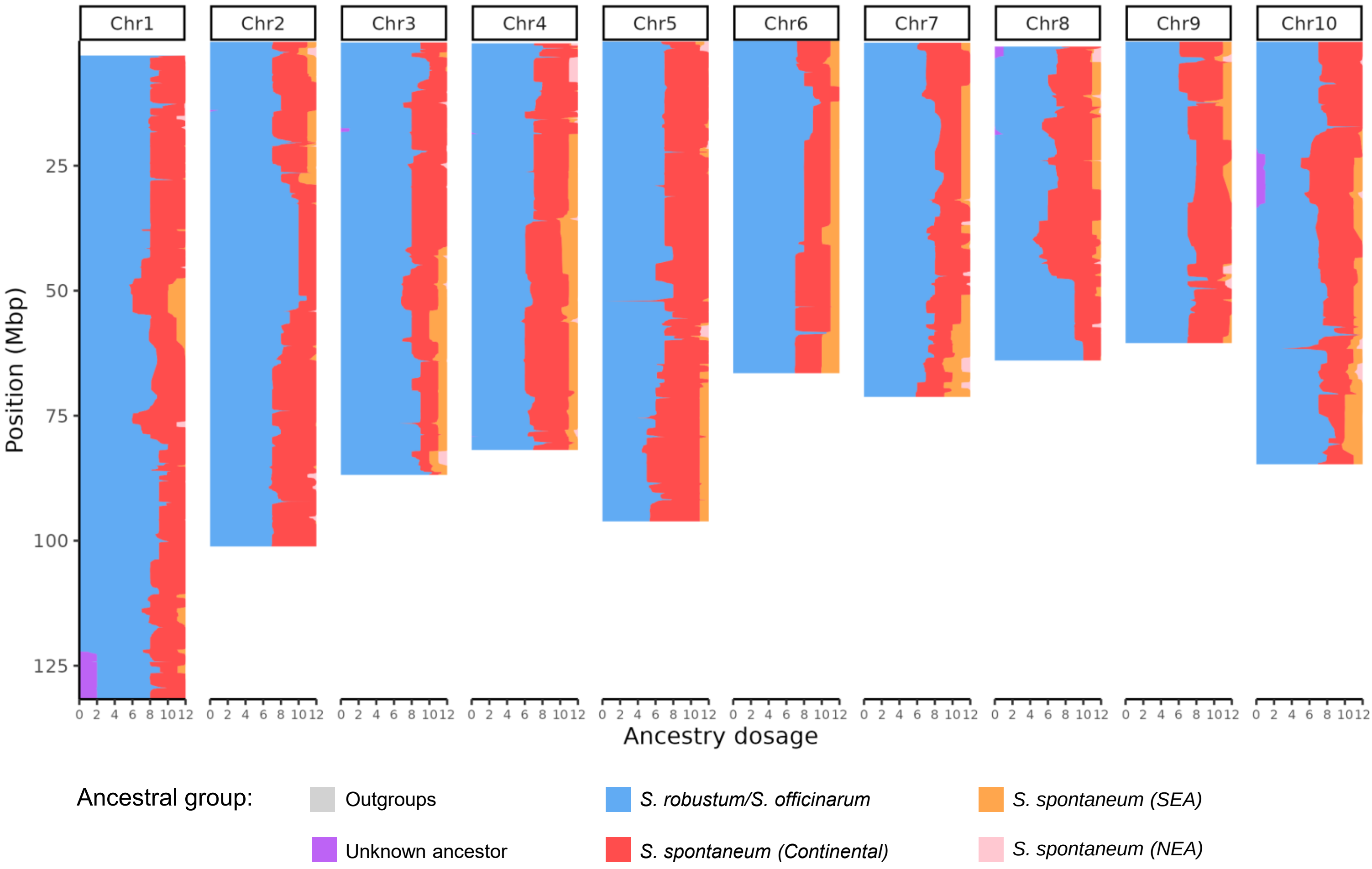

Local admixture of the modern cultivar NiTn18

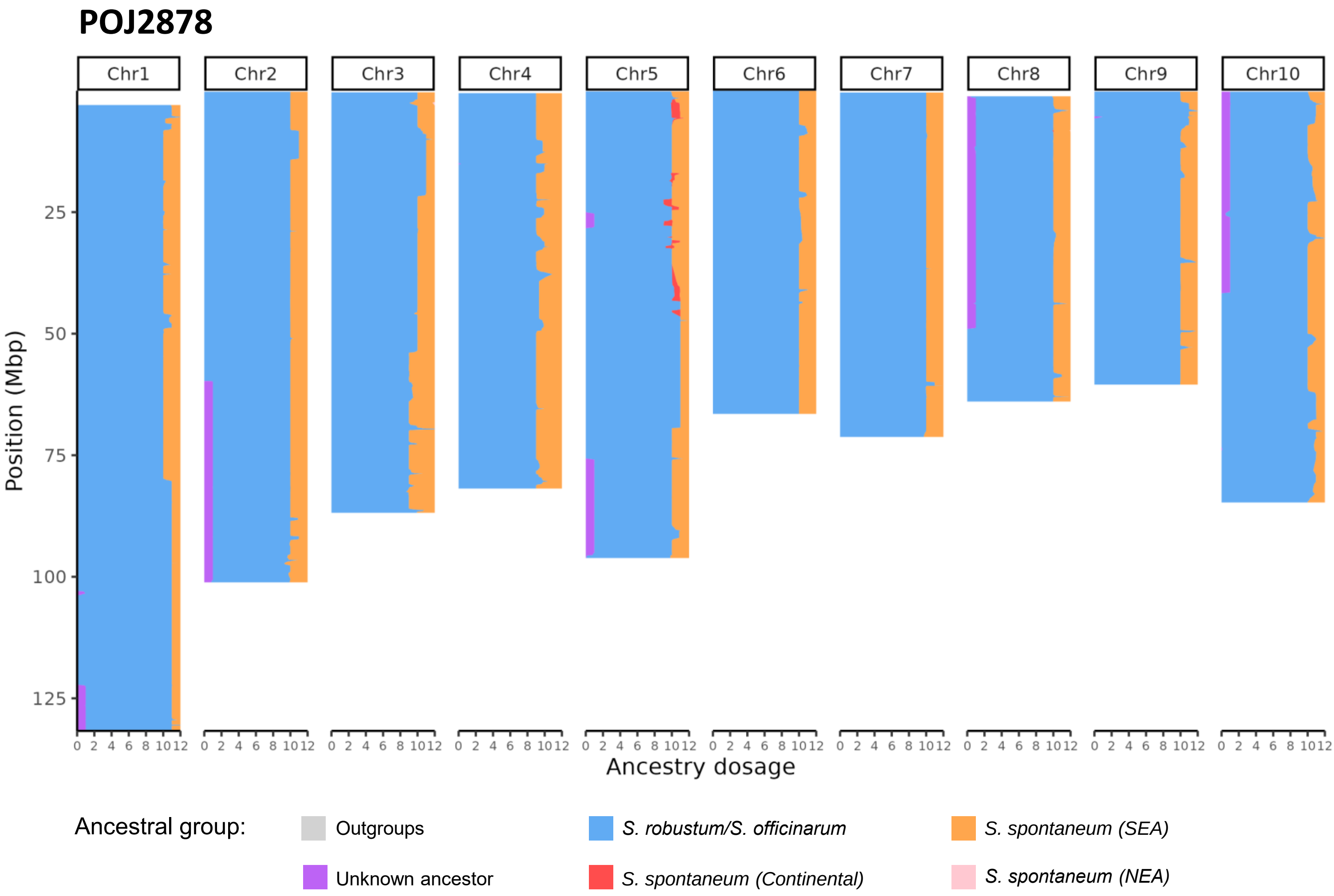

Local admixture of the modern cultivar POJ2878

Q124

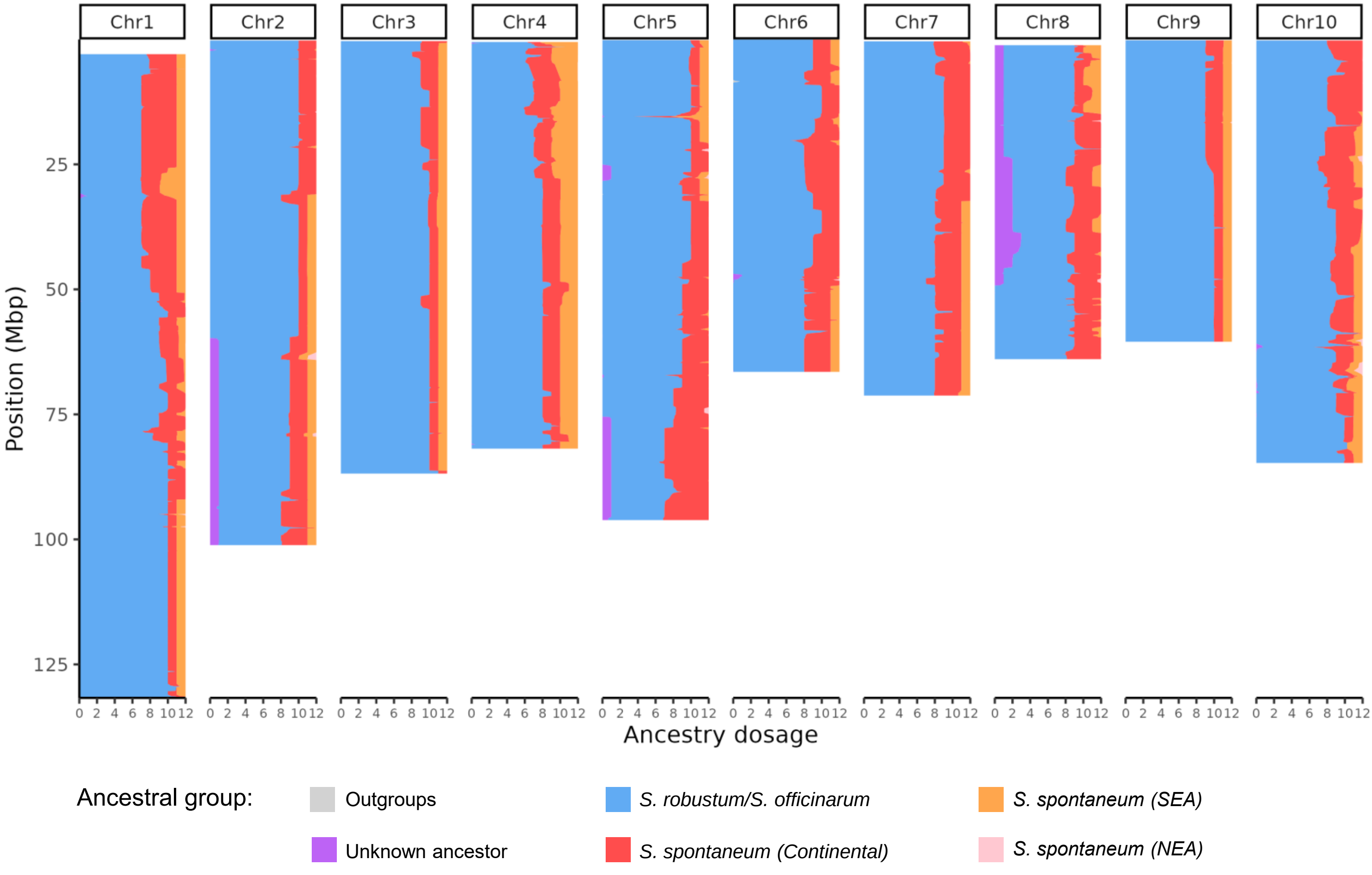

Local admixture of the modern cultivar Q124

Q138

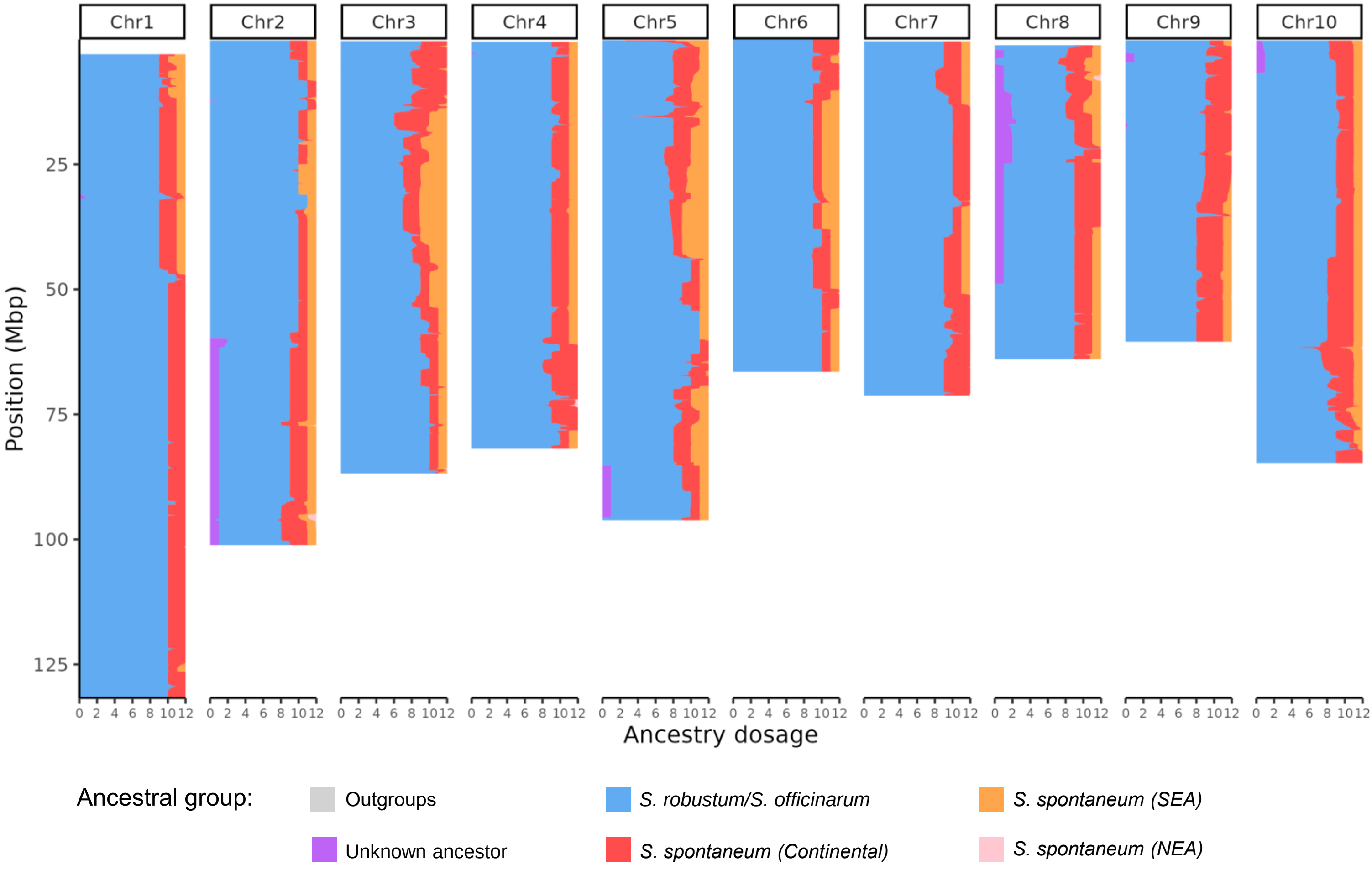

Local admixture of the modern cultivar Q138

Q162

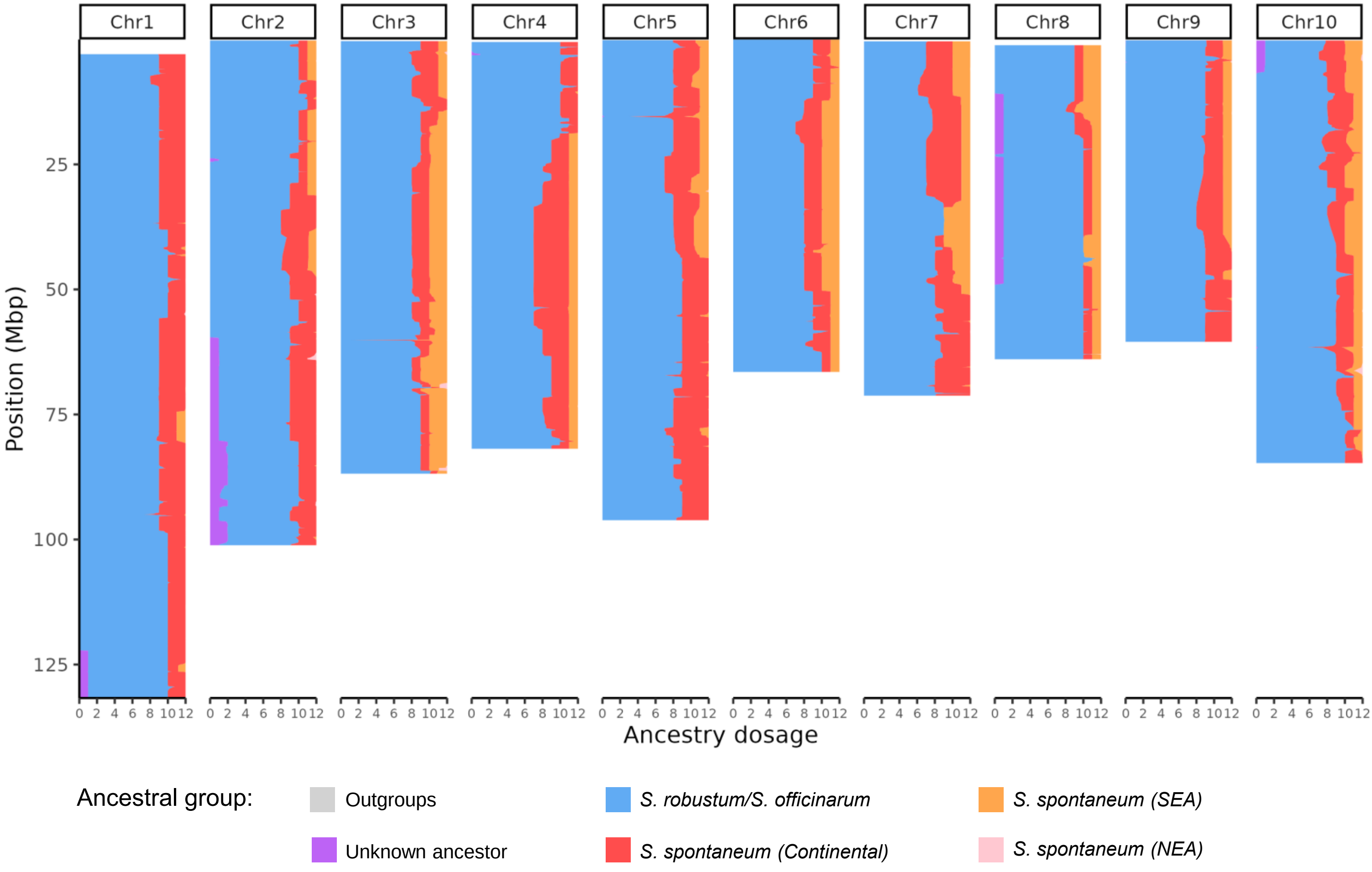

Local admixture of the modern cultivar Q162

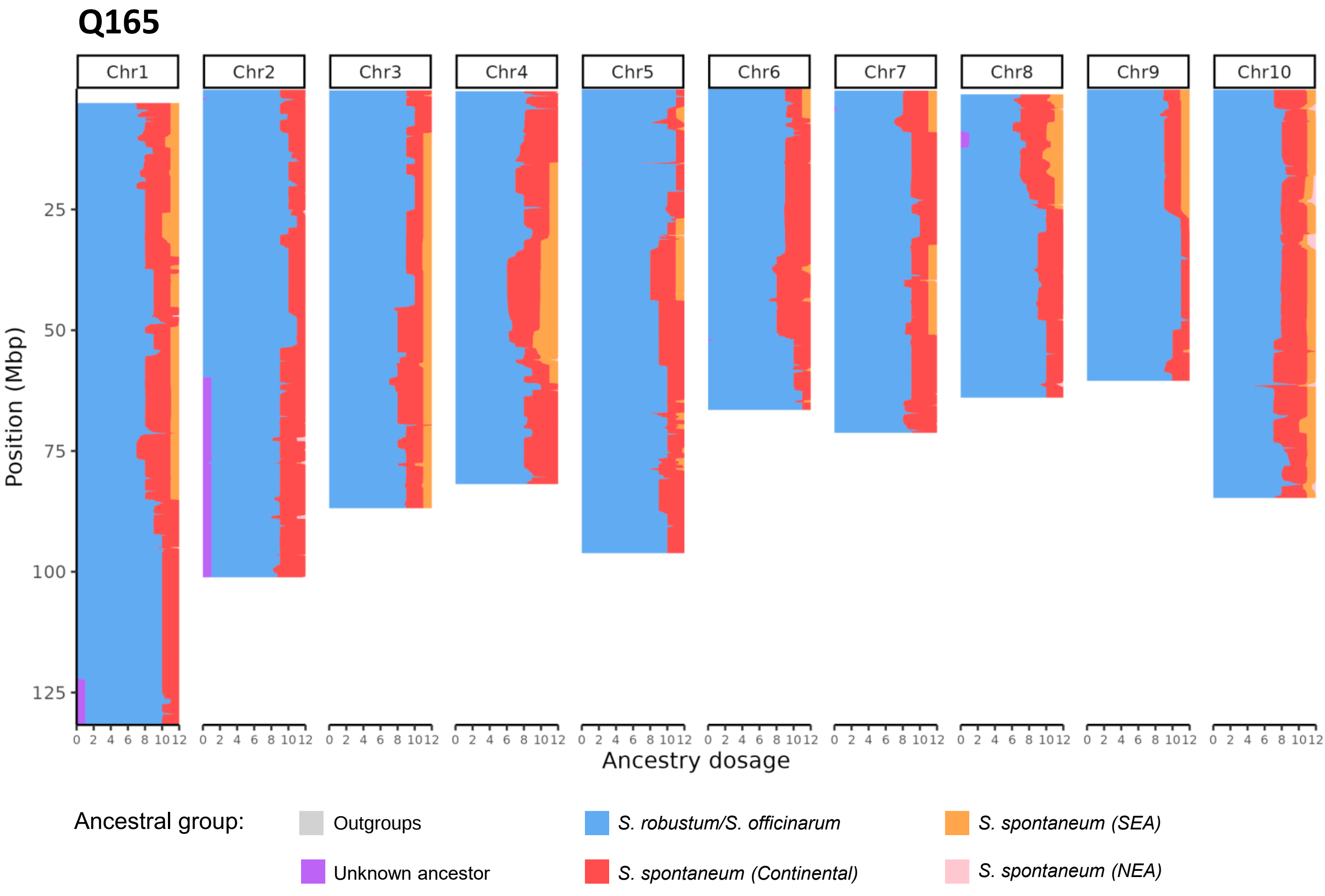

Local admixture of the modern cultivar Q165

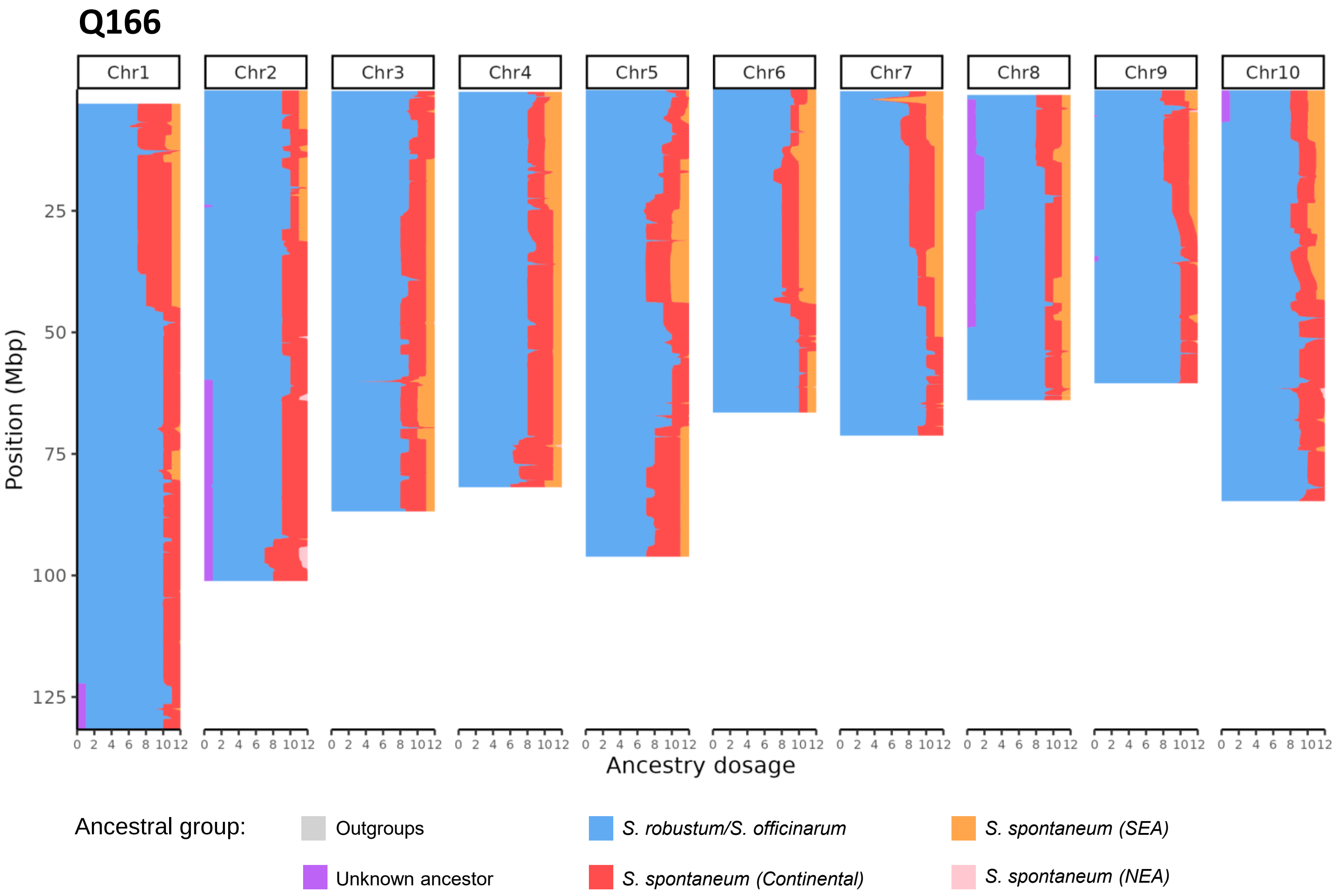

Local admixture of the modern cultivar Q166

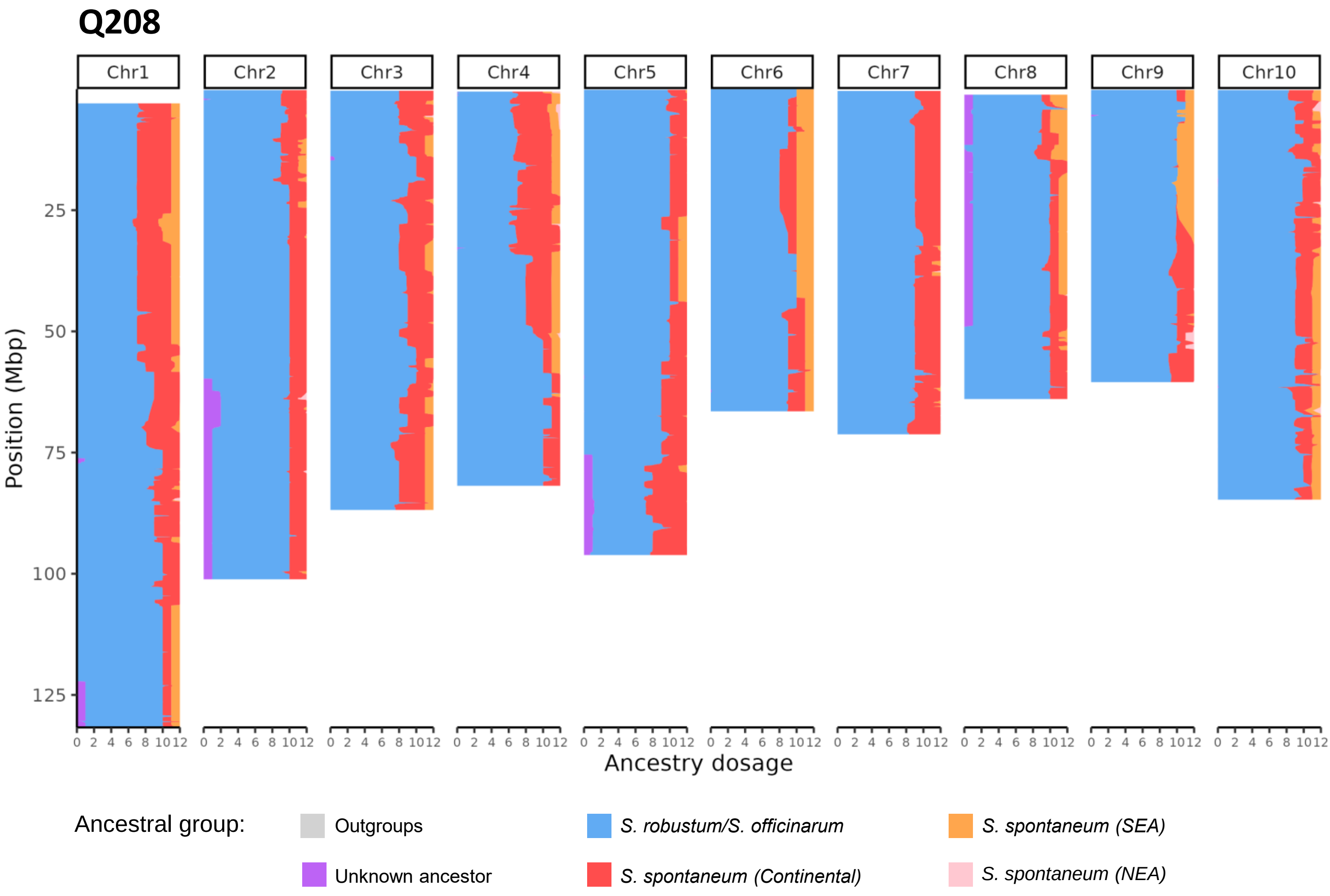

Local admixture of the modern cultivar Q208

Q209

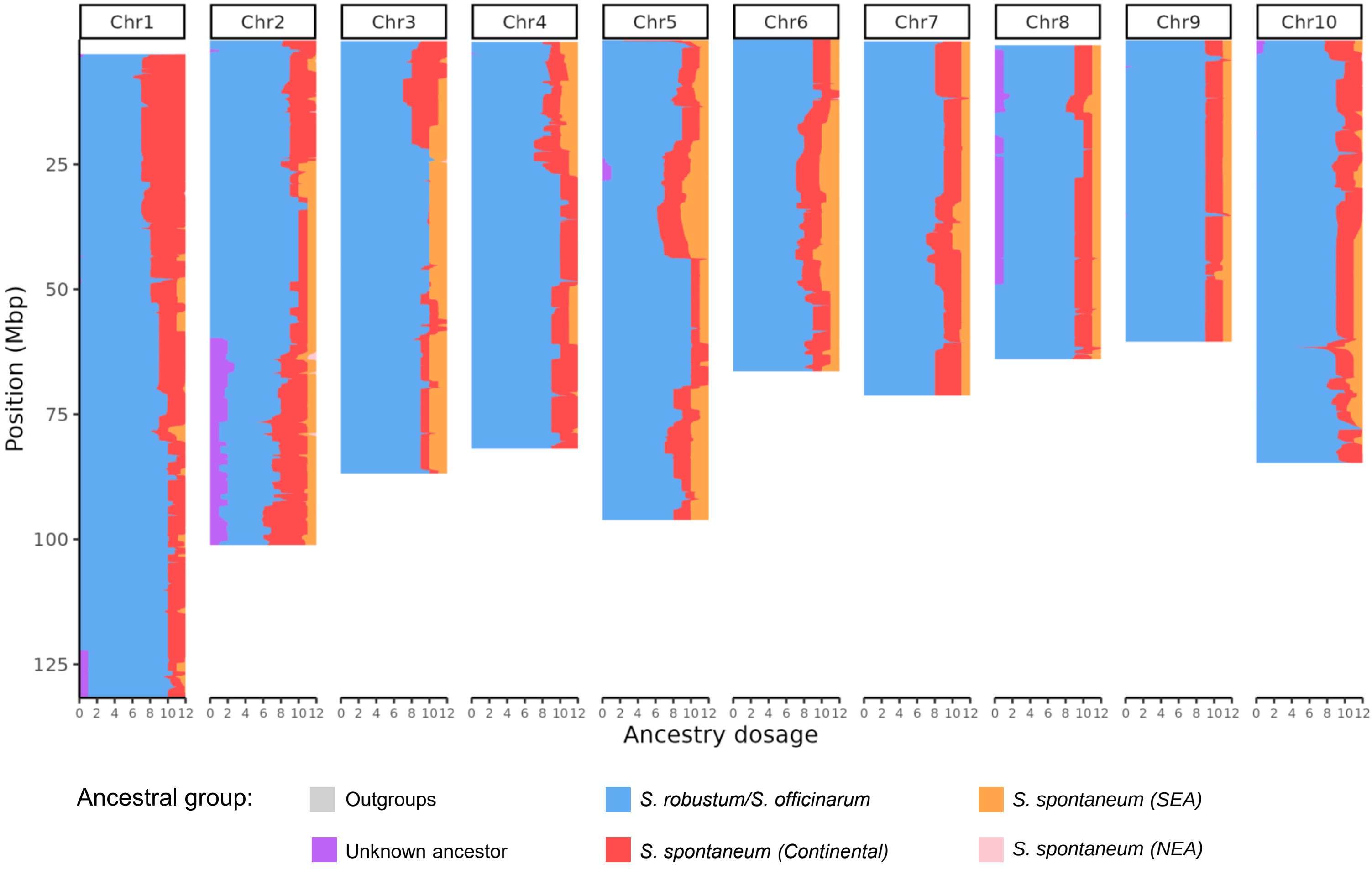

Local admixture of the modern cultivar Q209

Q232

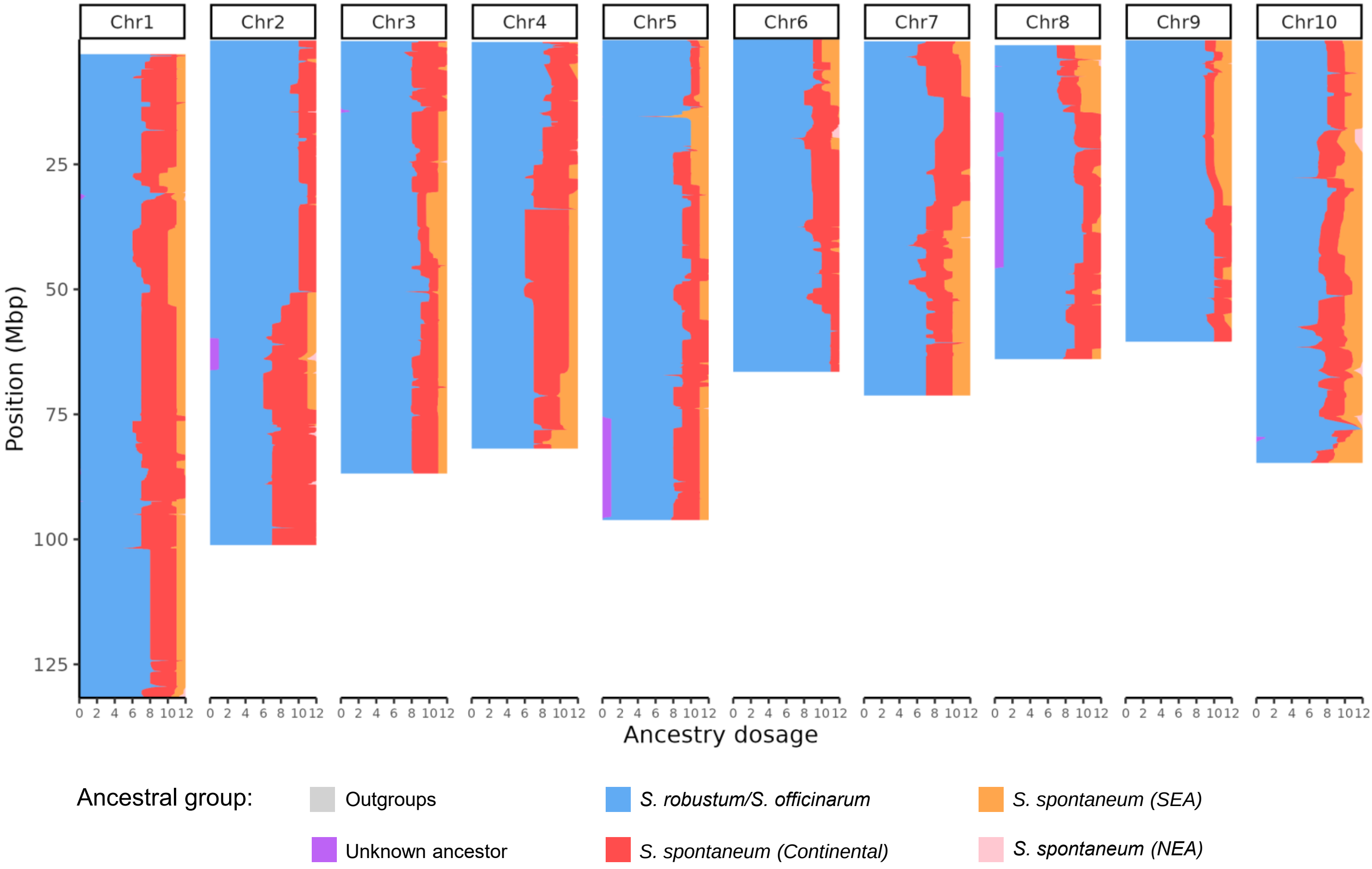

Local admixture of the modern cultivar Q232

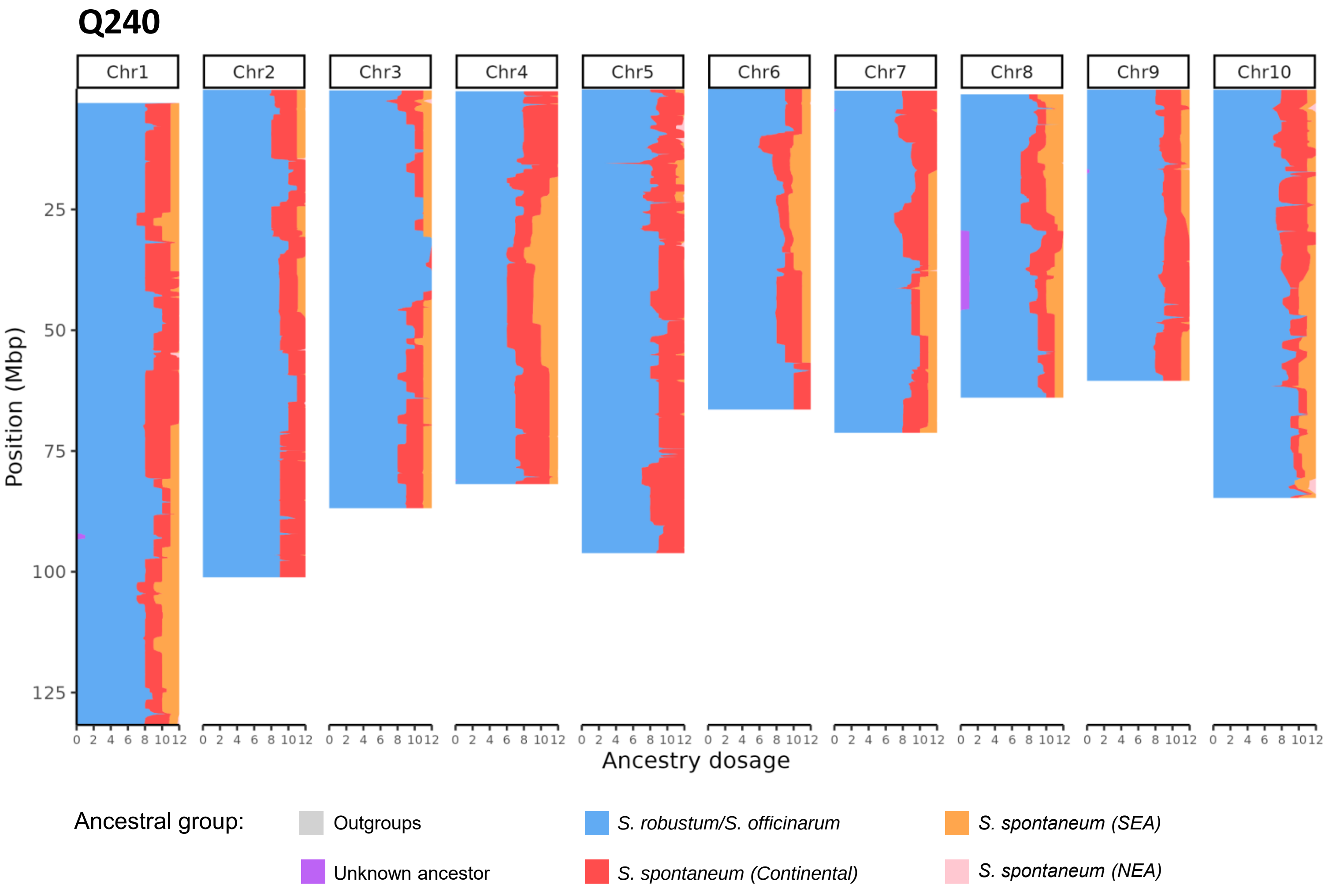

Local admixture of the modern cultivar Q240

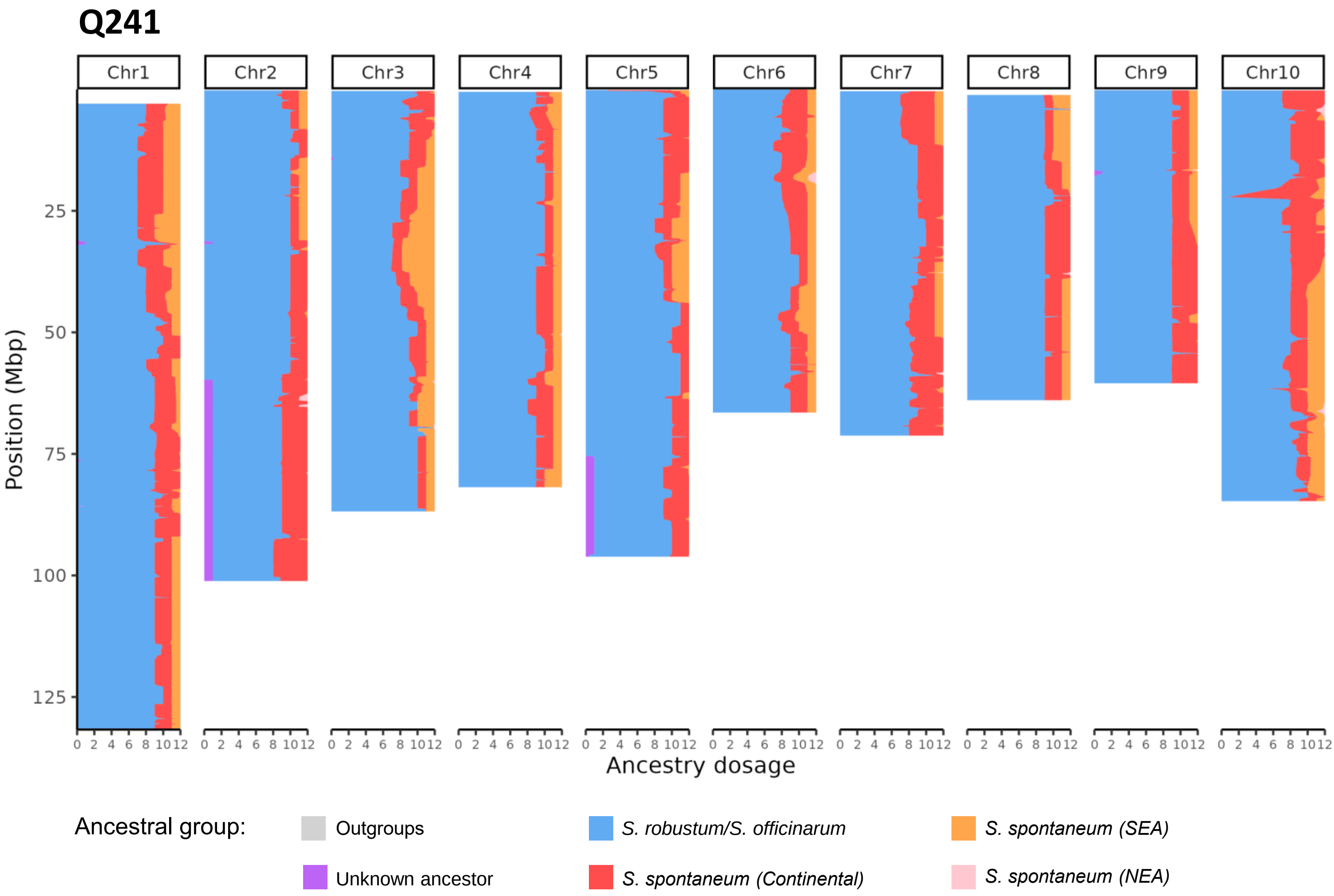

Local admixture of the modern cultivar Q241

Q253

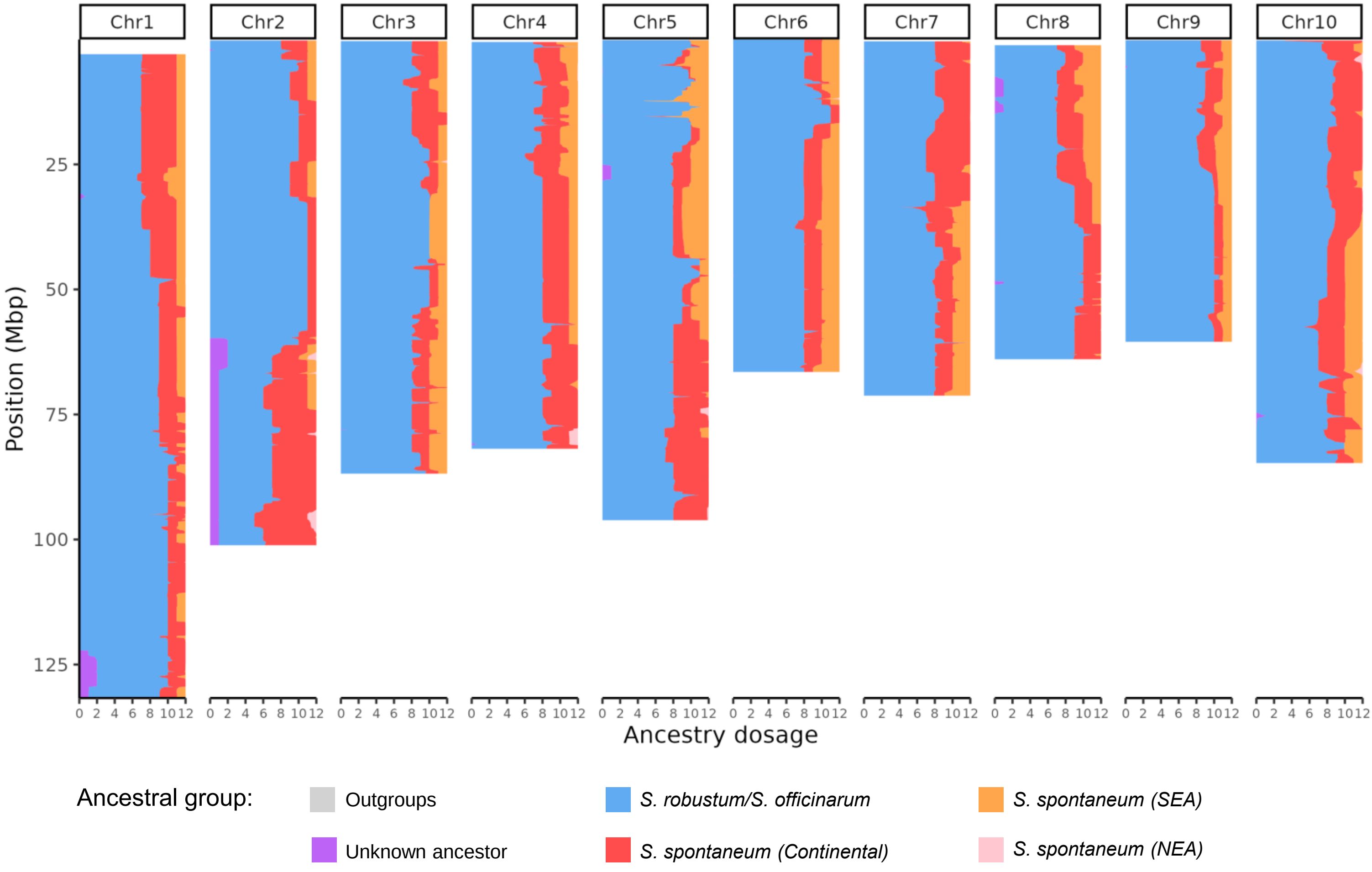

Local admixture of the modern cultivar Q253

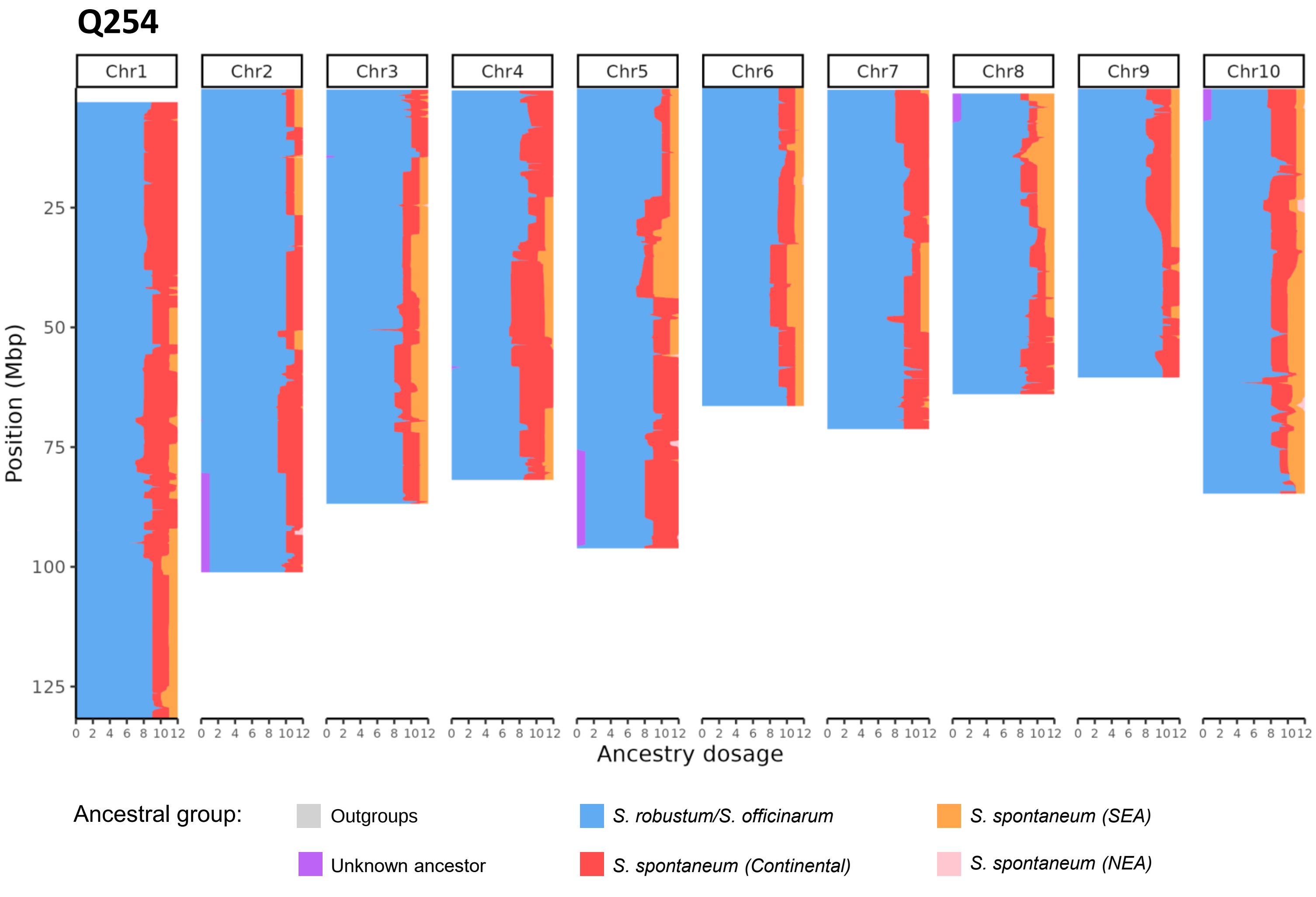

Local admixture of the modern cultivar Q254

QC90-289

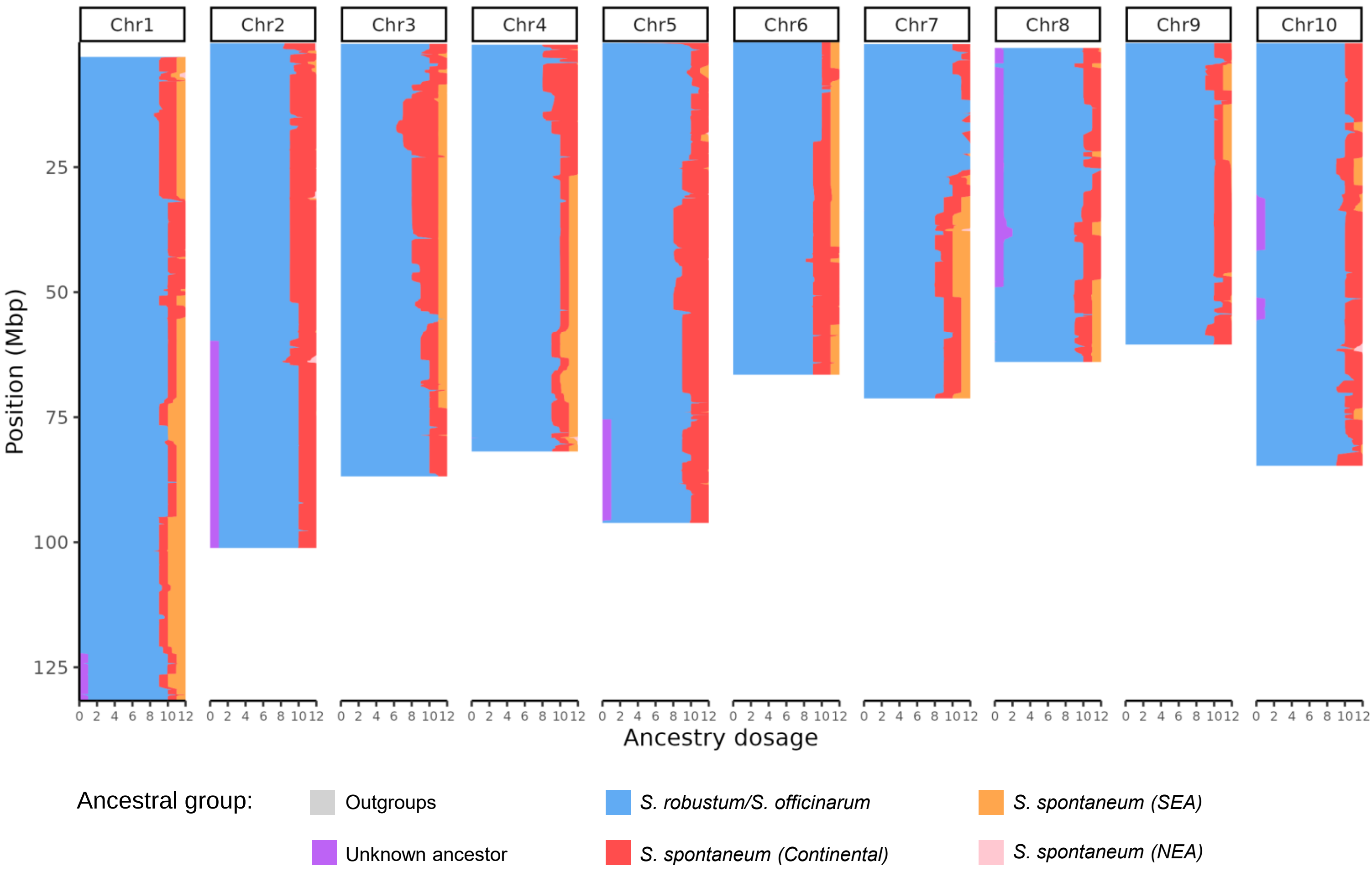

Local admixture of the modern cultivar QC90-289

QN58-829

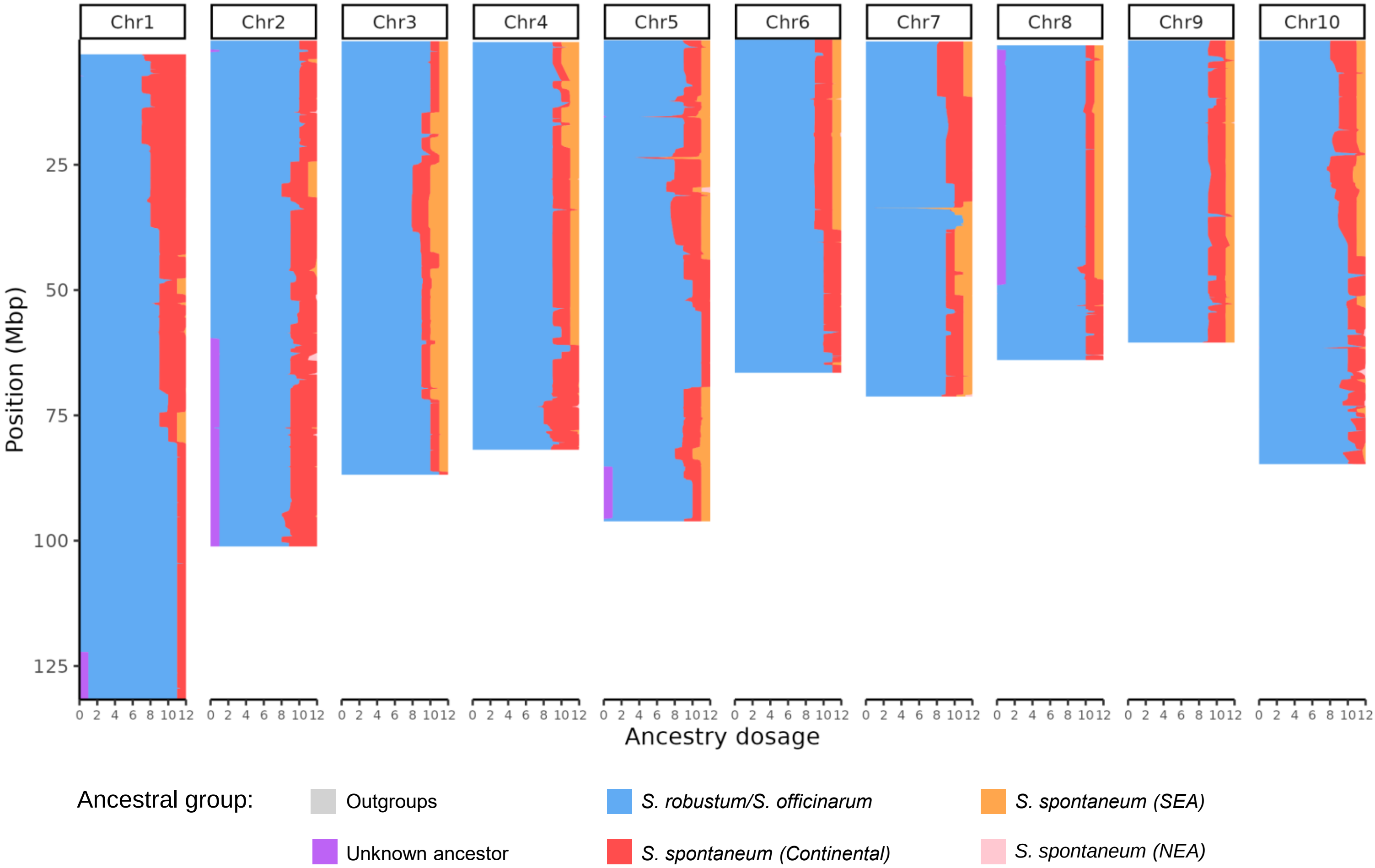

Local admixture of the modern cultivar QN58-829

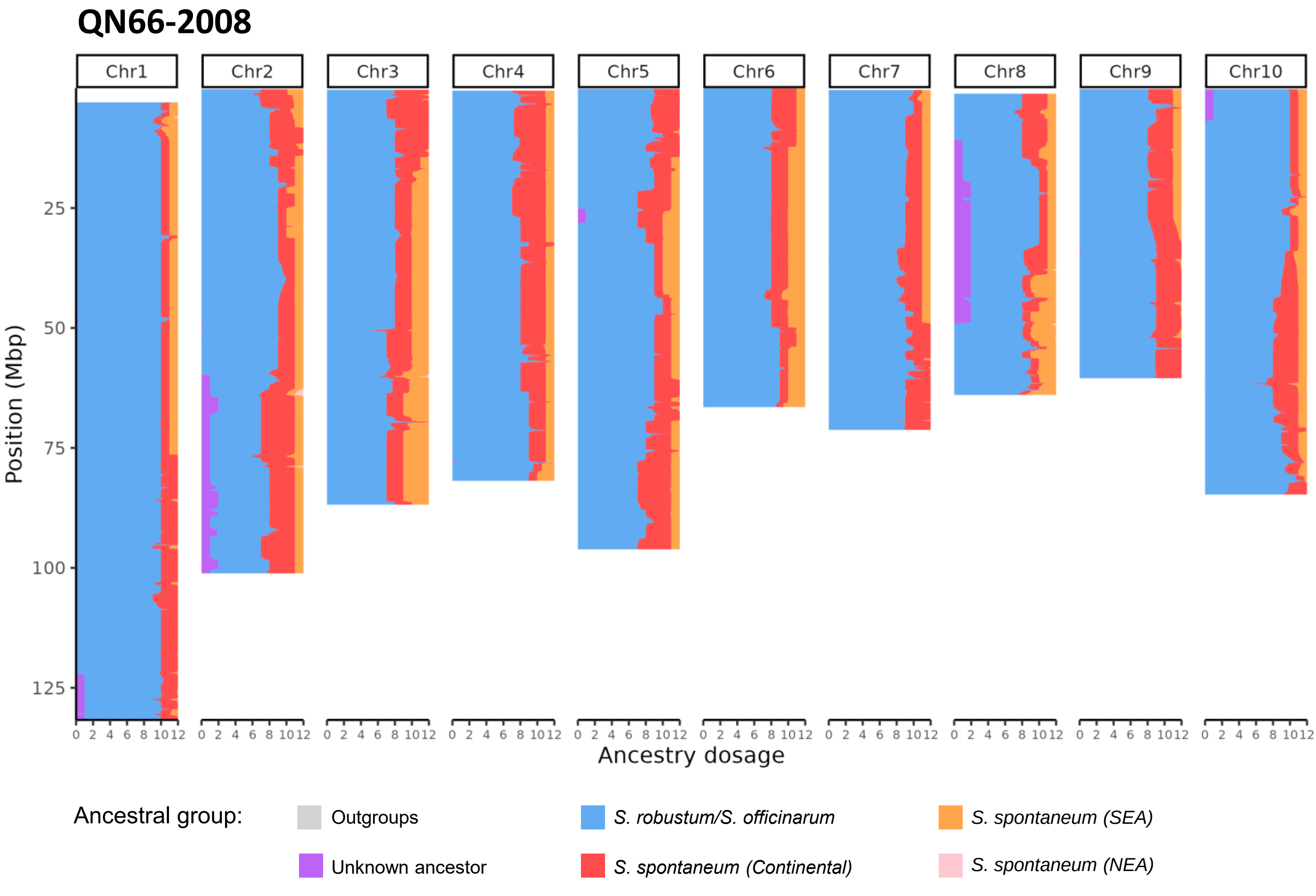

Local admixture of the modern cultivar QN66-2008

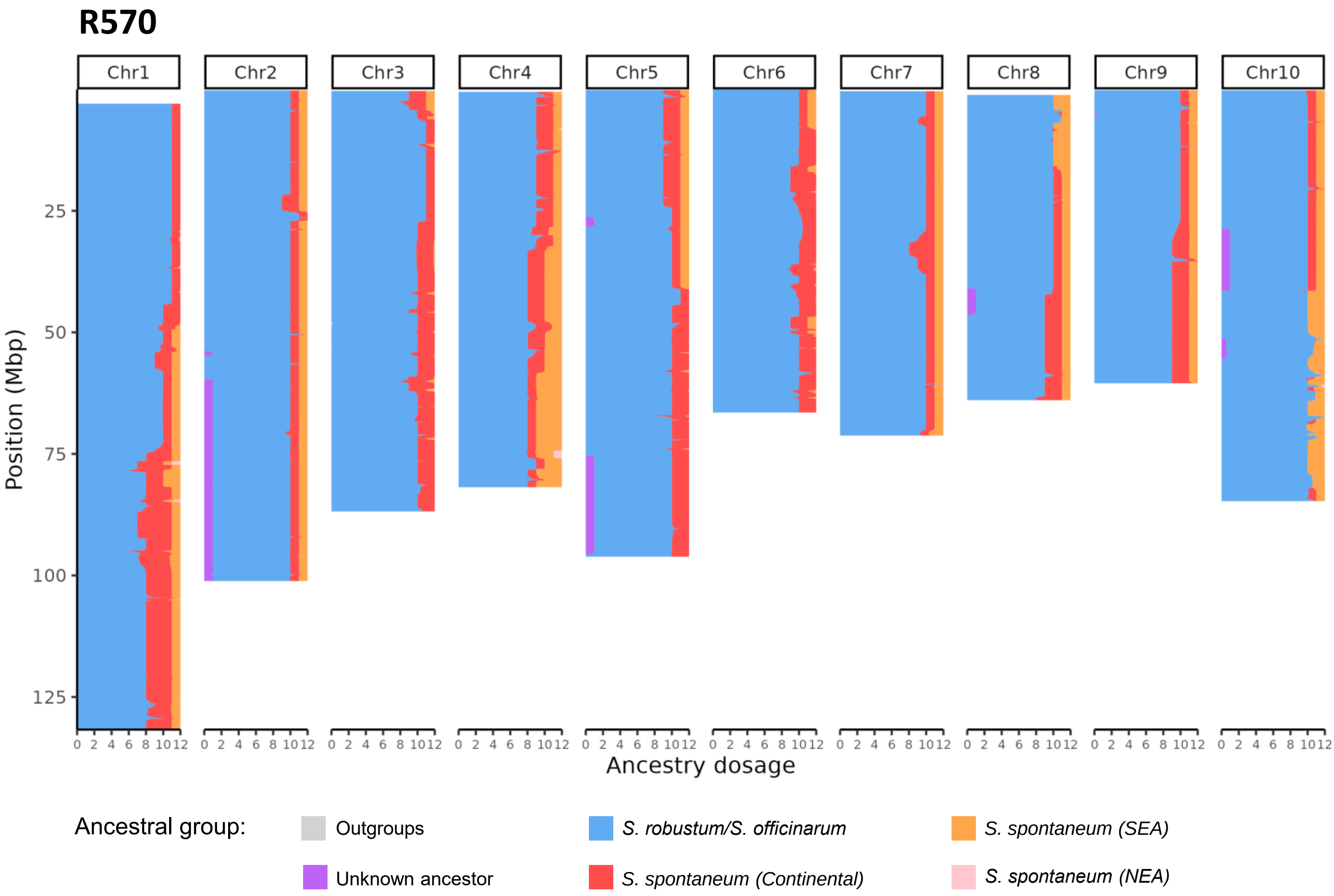

Local admixture of the modern cultivar R570

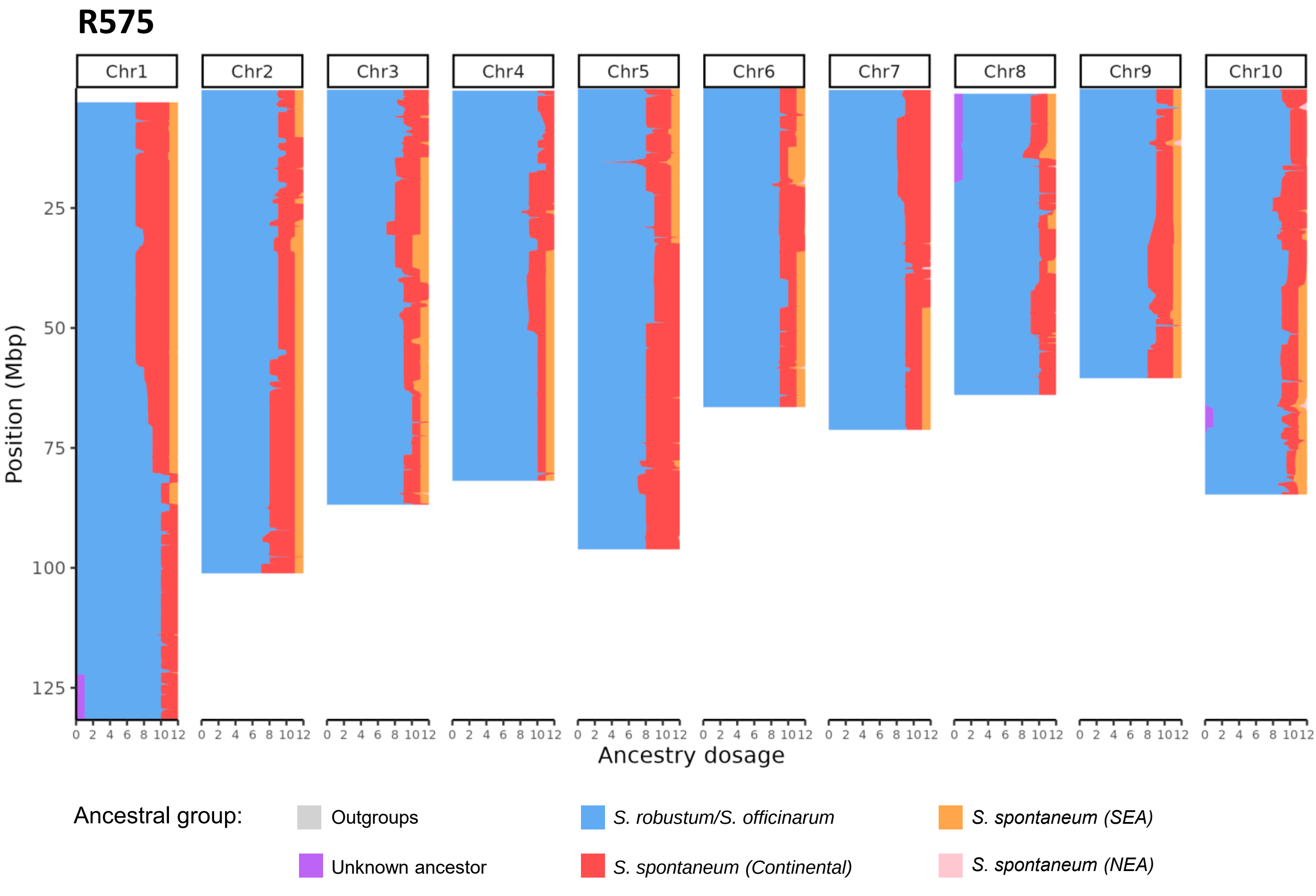

Local admixture of the modern cultivar R575

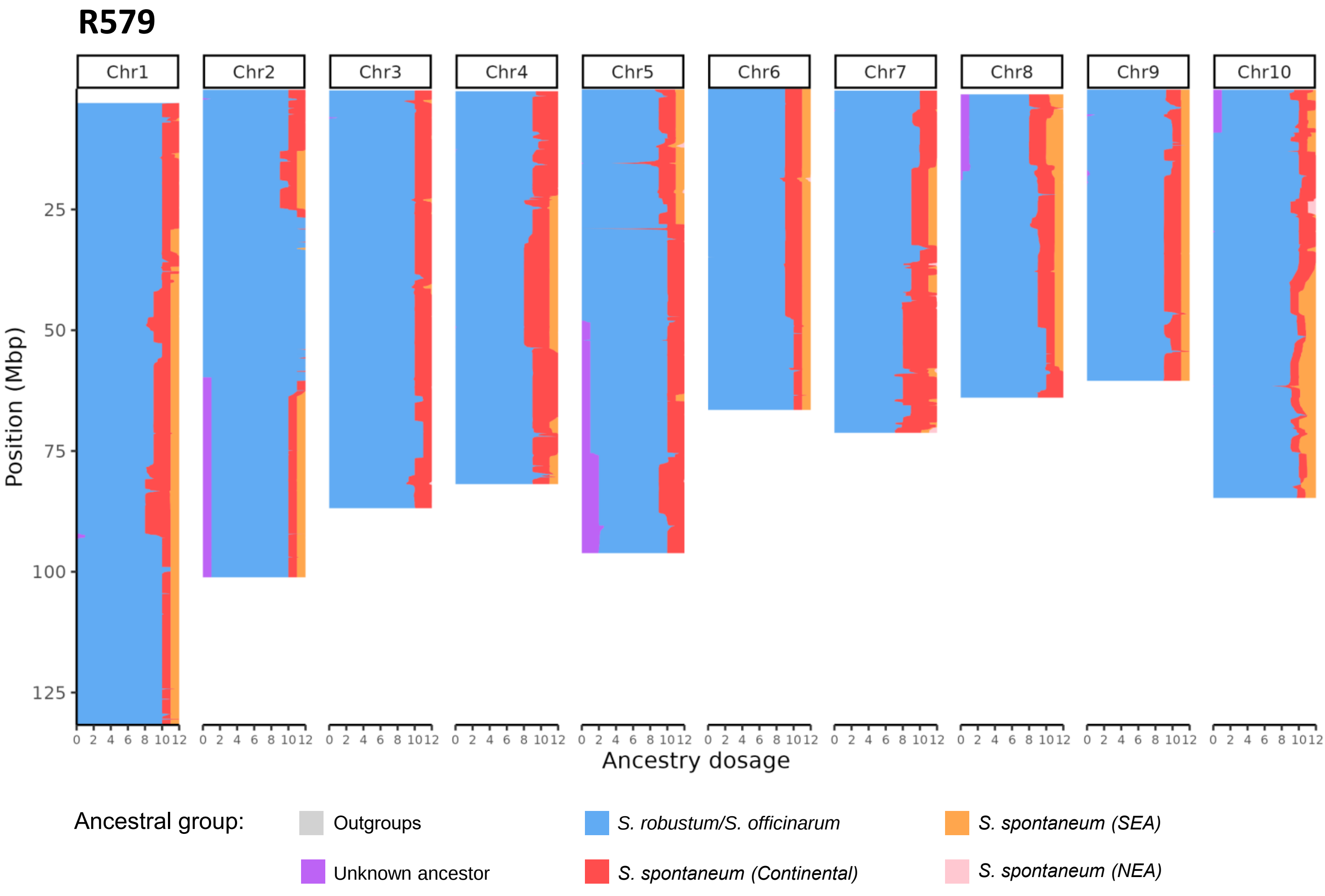

Local admixture of the modern cultivar R579

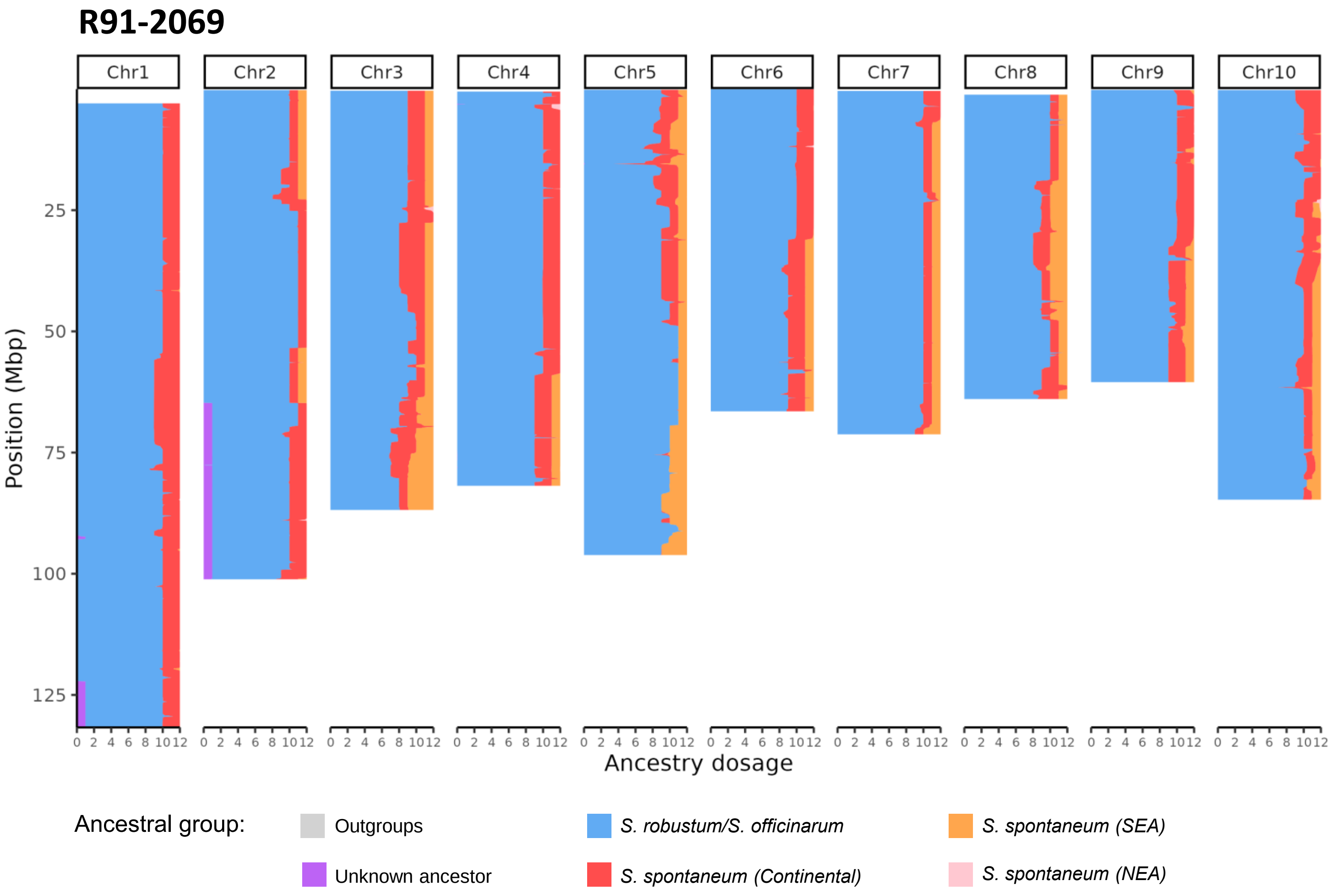

Local admixture of the modern cultivar R91-2069

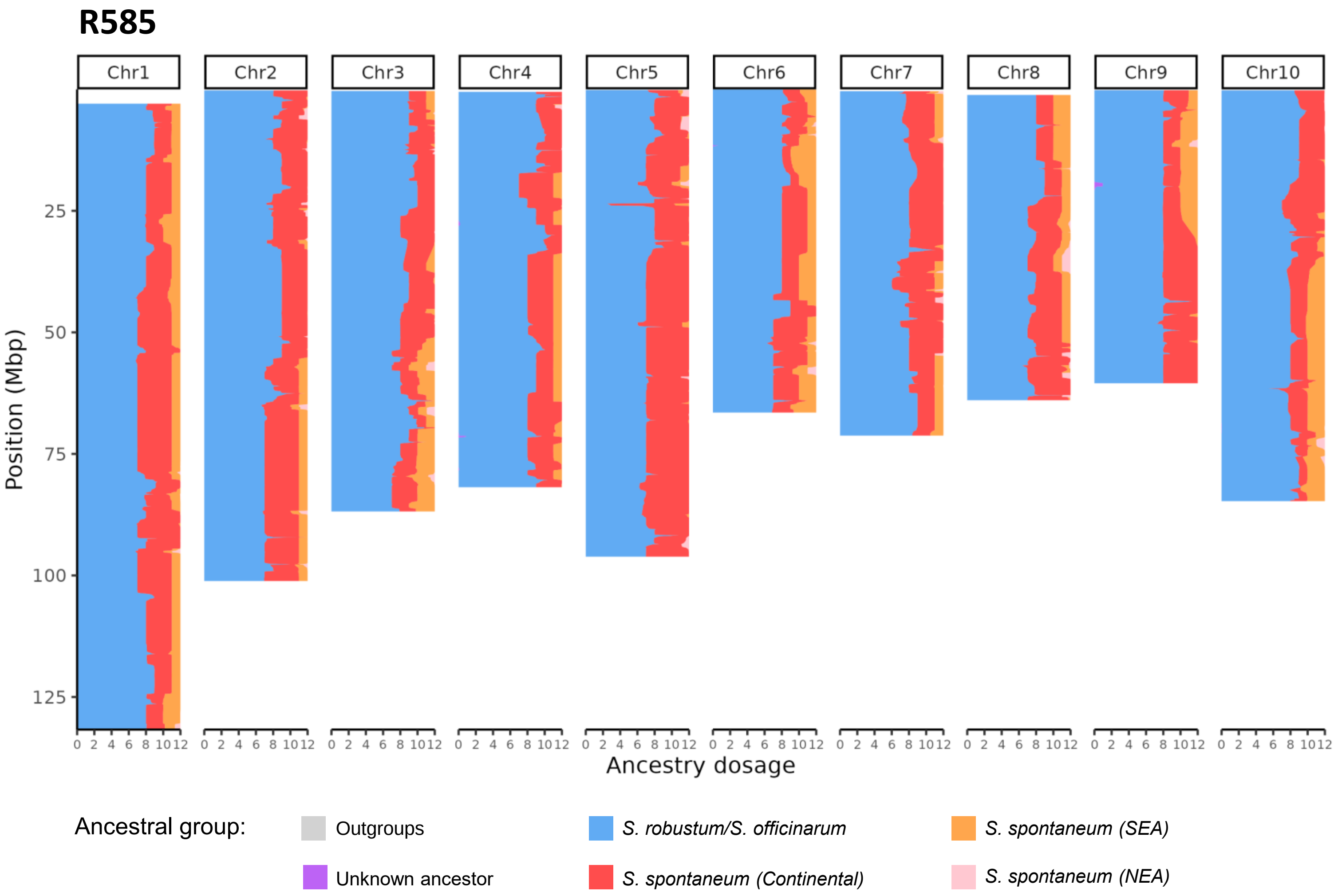

Local admixture of the modern cultivar R585

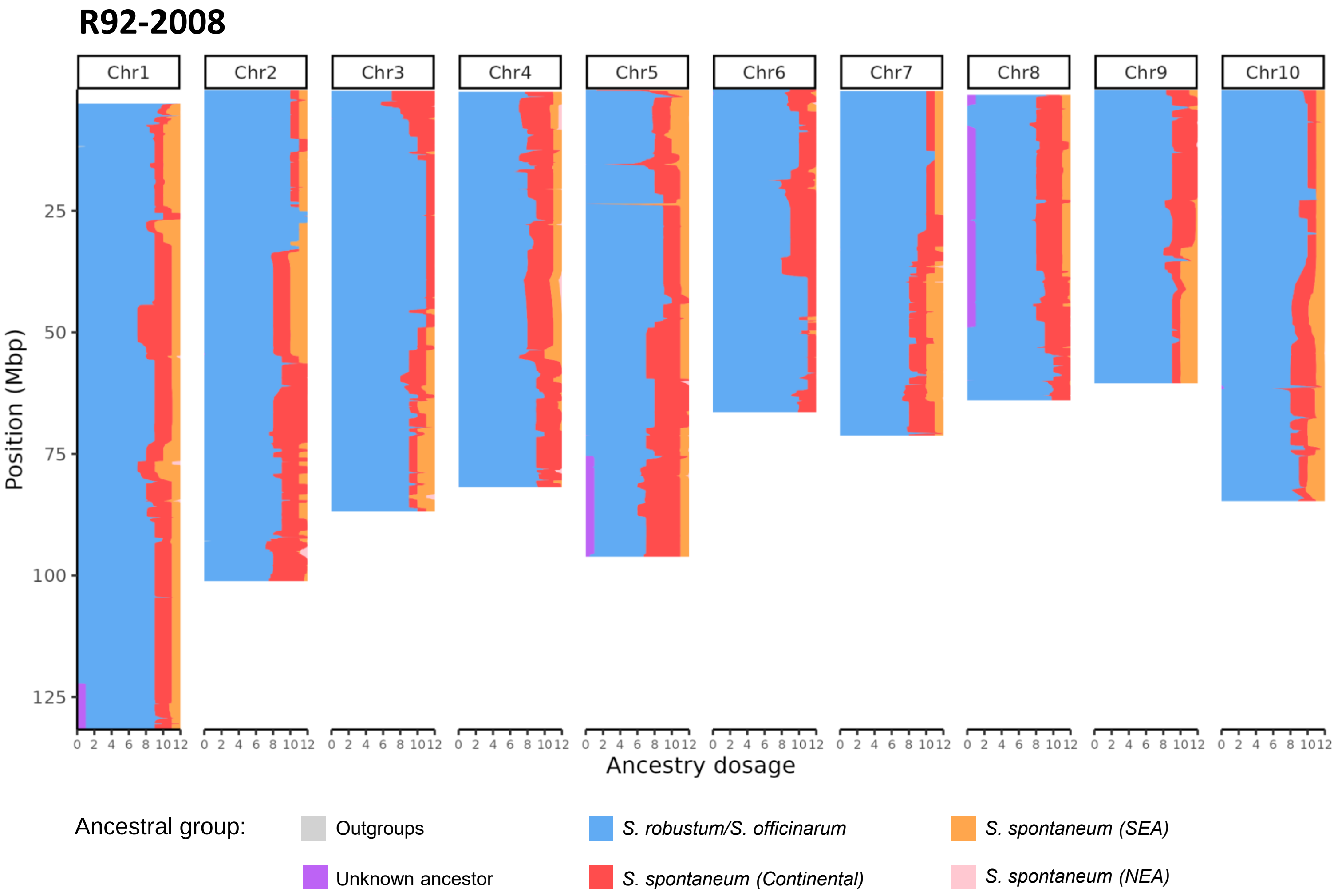

Local admixture of the modern cultivar R92-2008

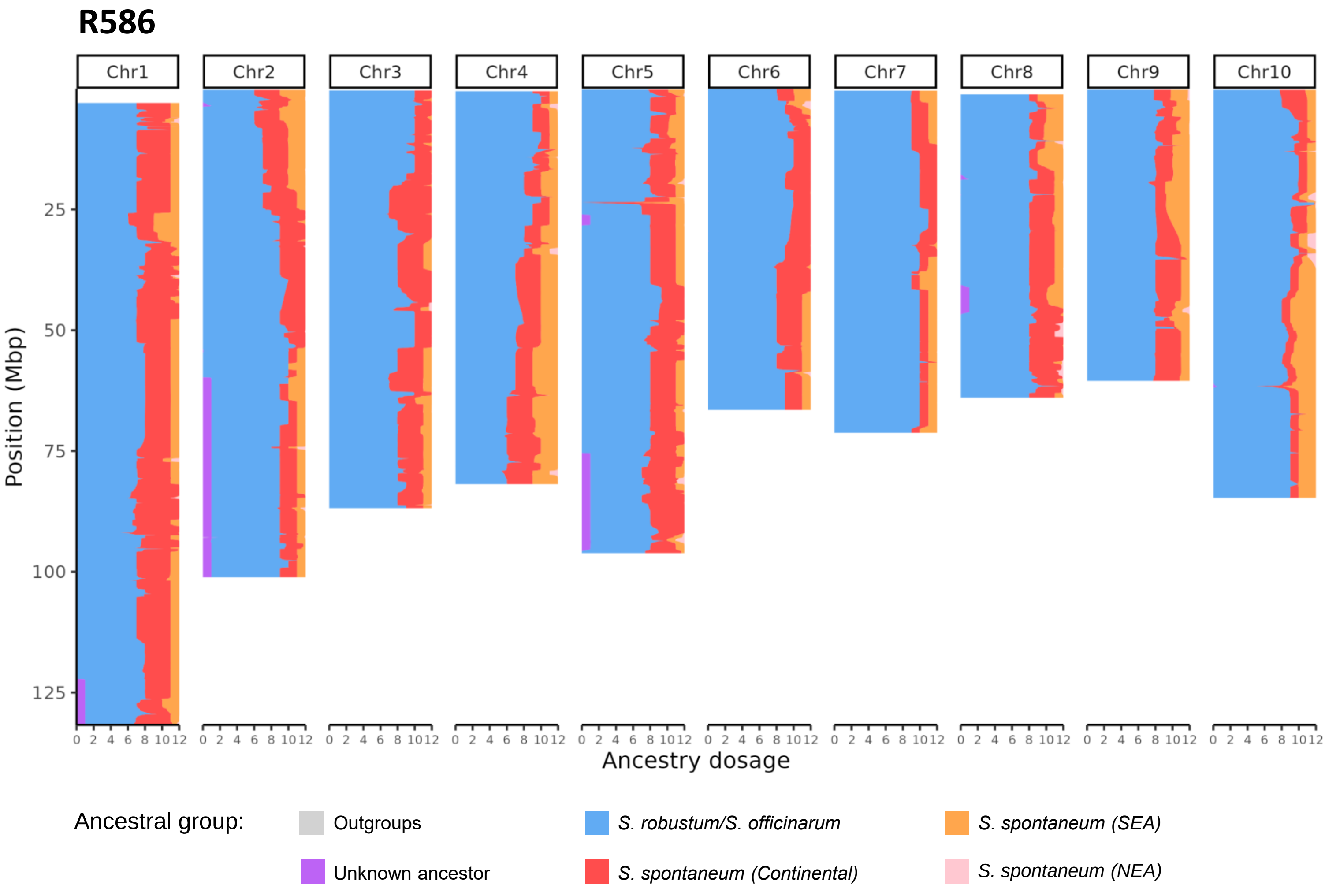

Local admixture of the modern cultivar R586

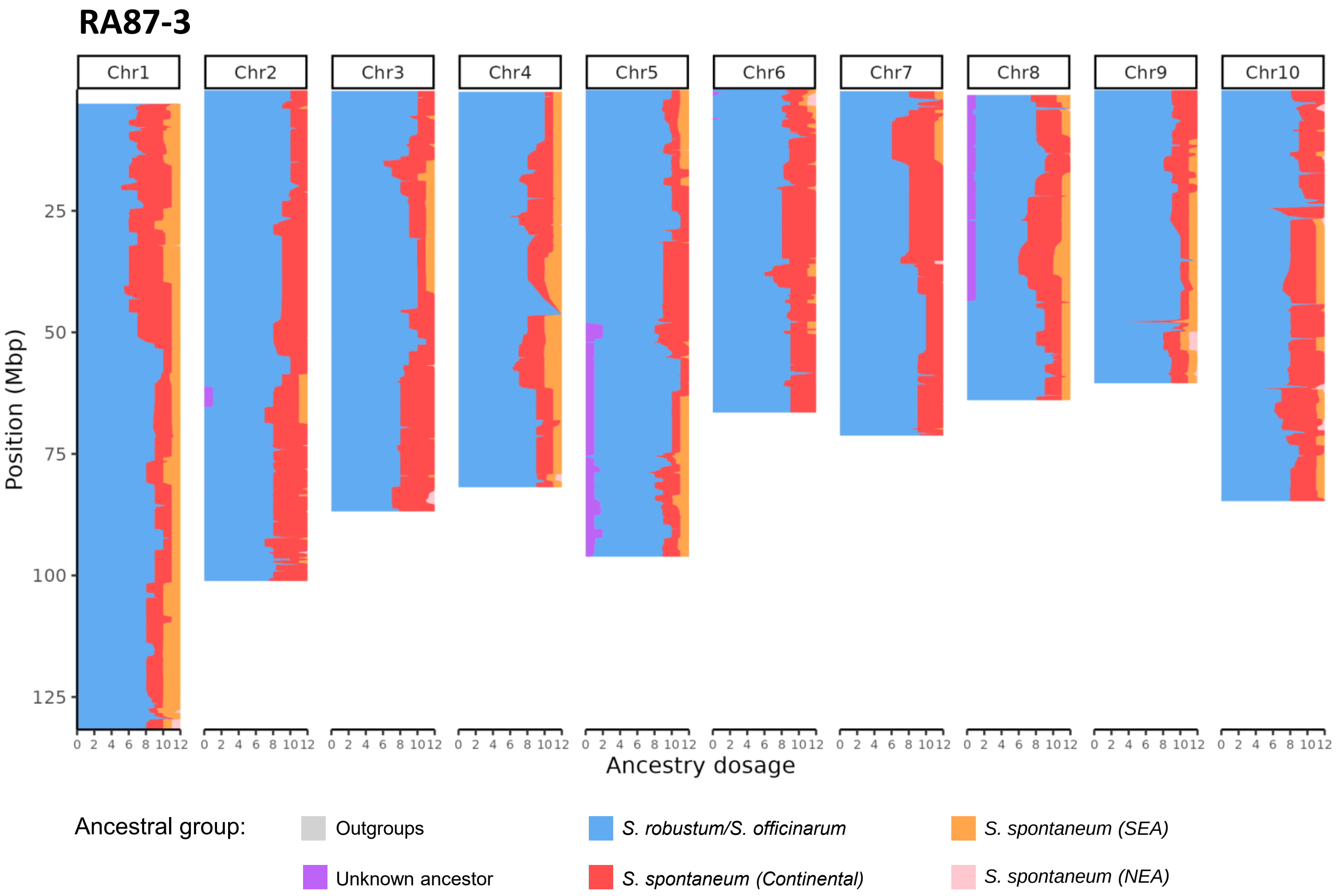

Local admixture of the modern cultivar RA87-3

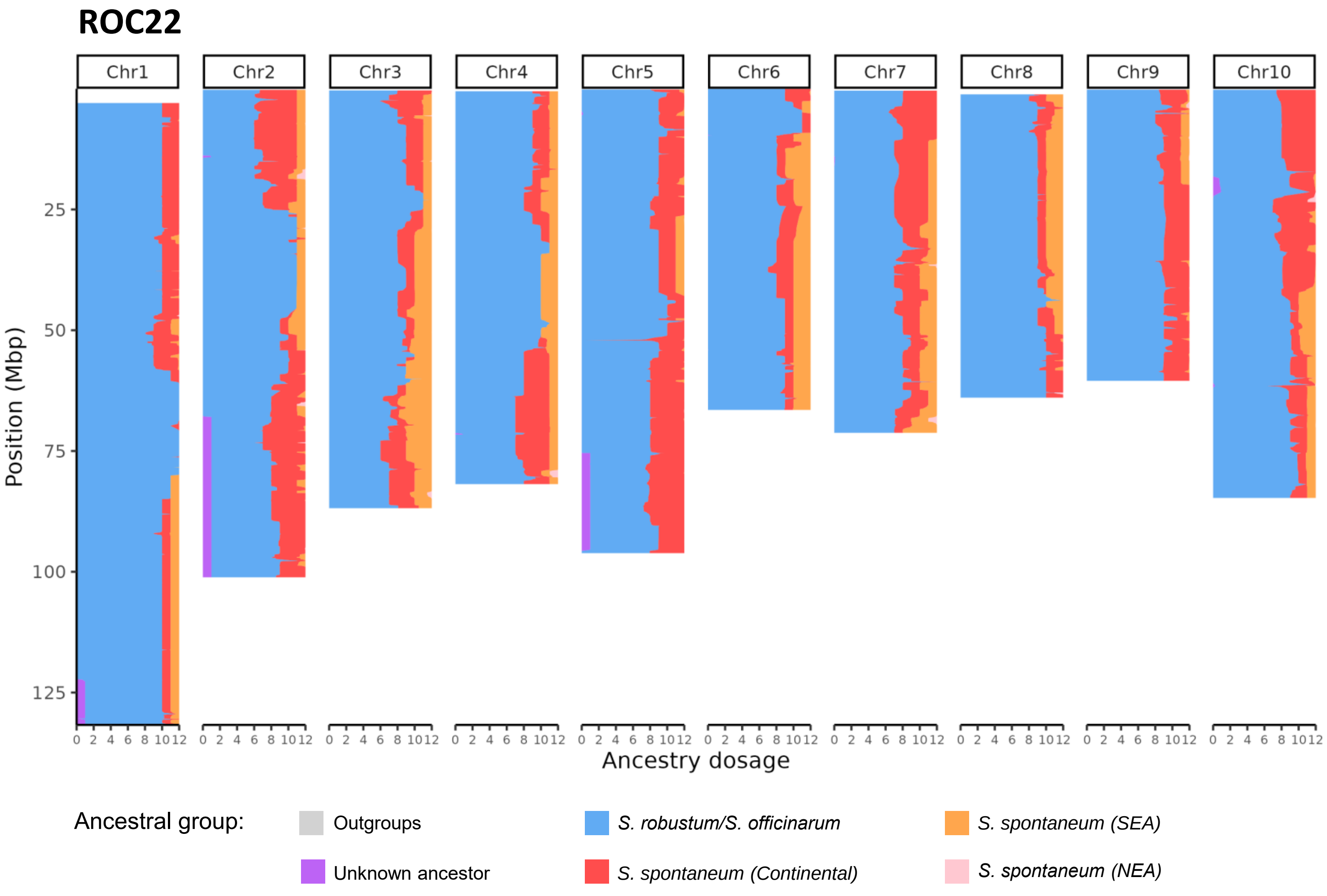

Local admixture of the modern cultivar ROC22

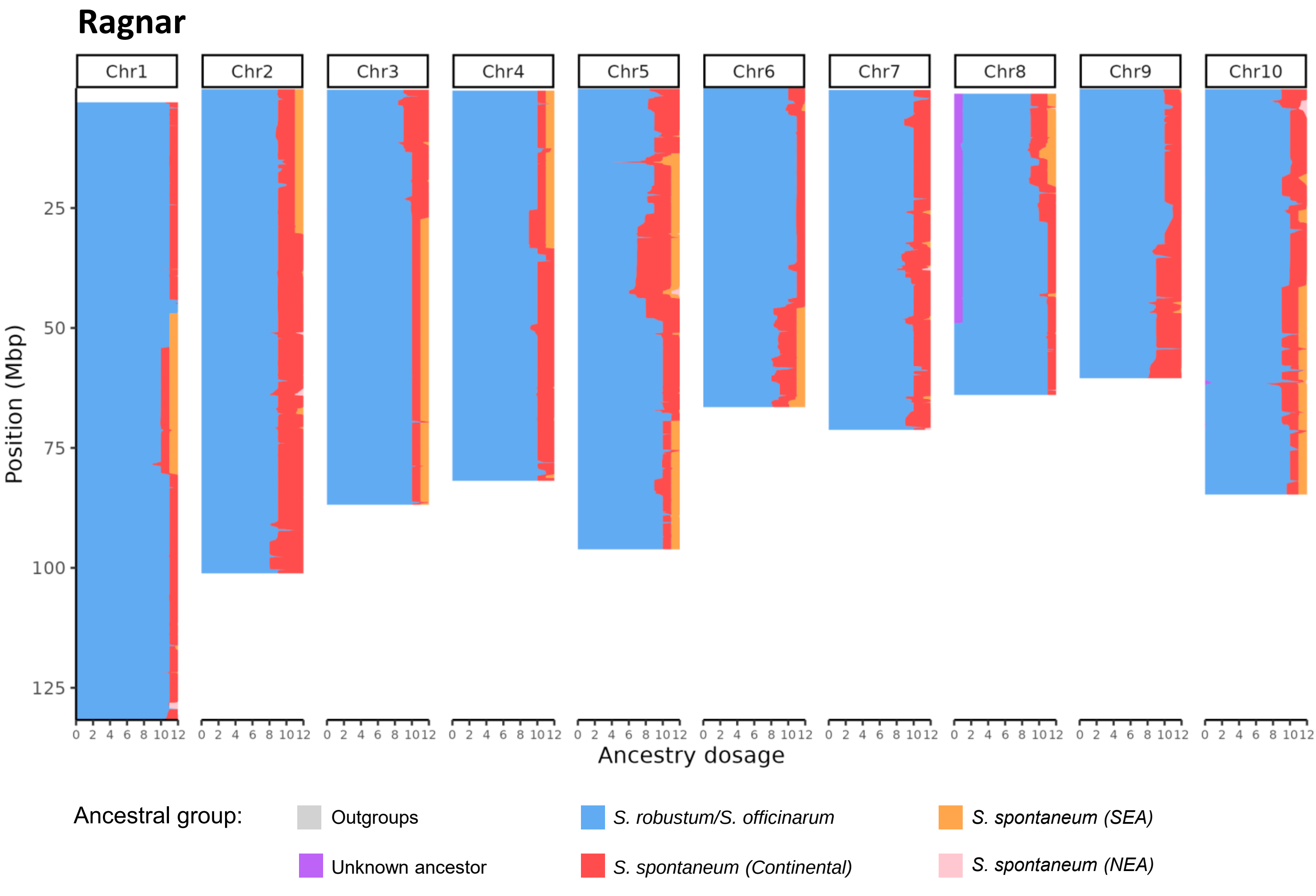

Local admixture of the modern cultivar Ragnar

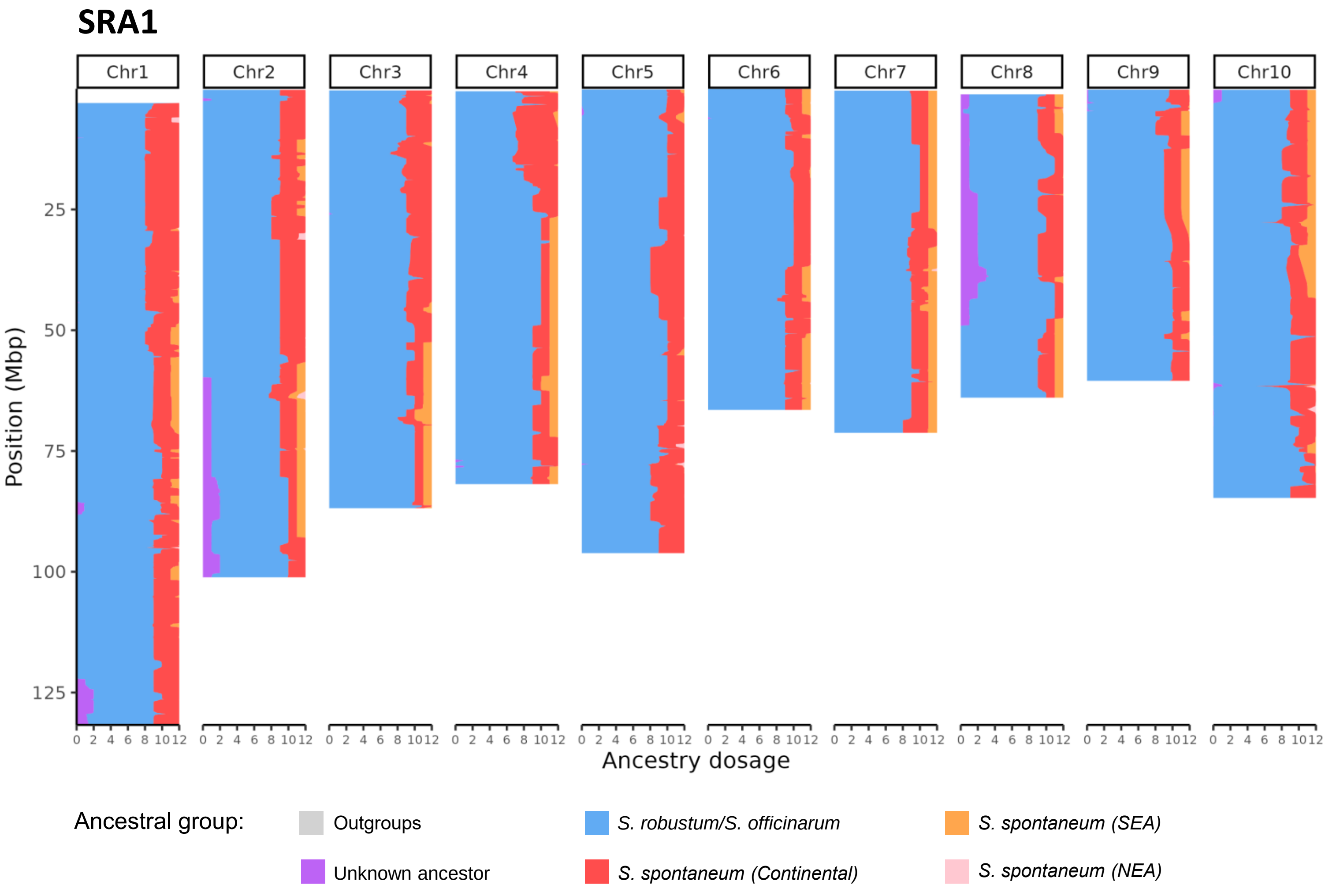

Local admixture of the modern cultivar SRA1

SRA4

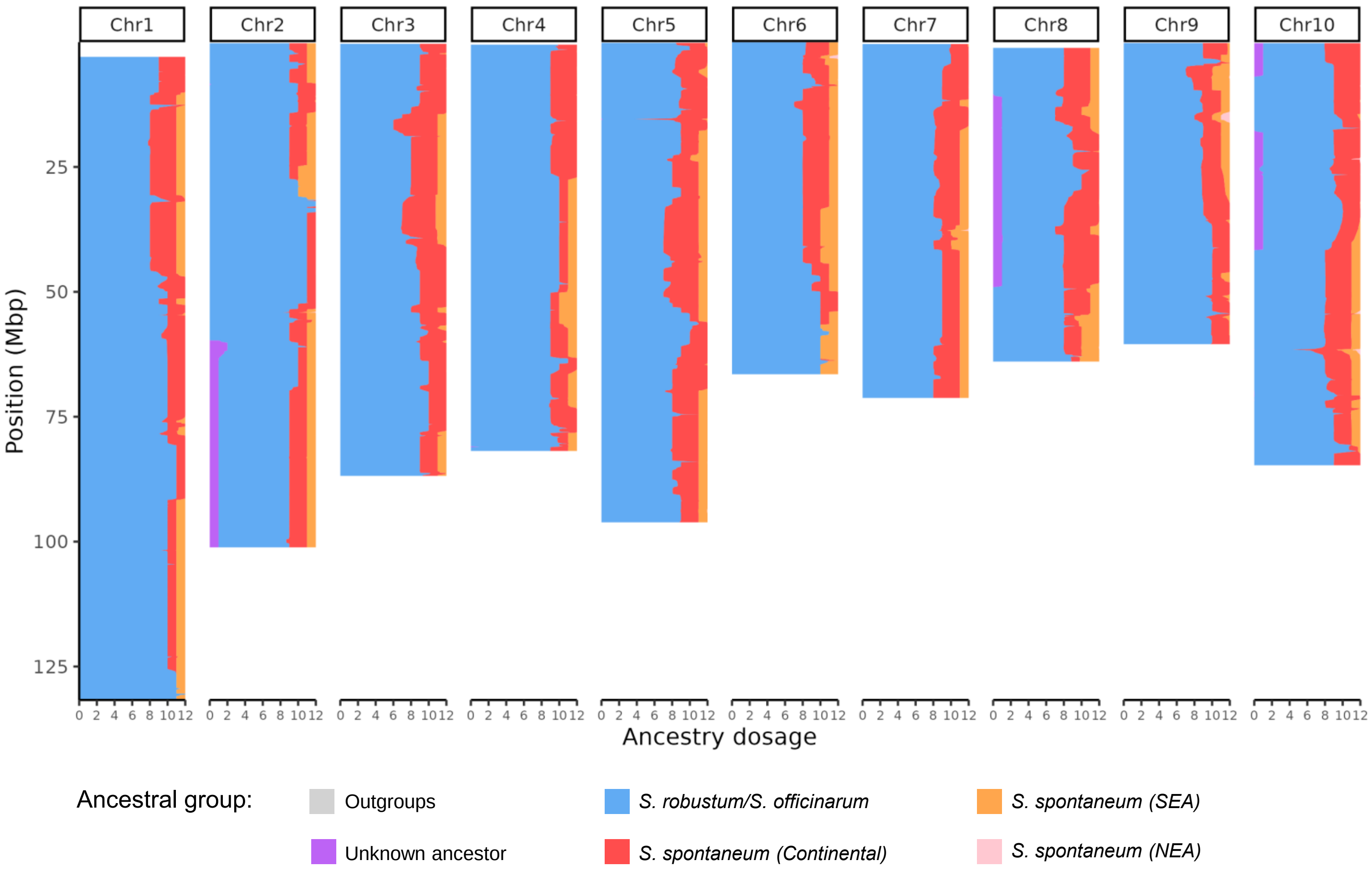

Local admixture of the modern cultivar SRA4

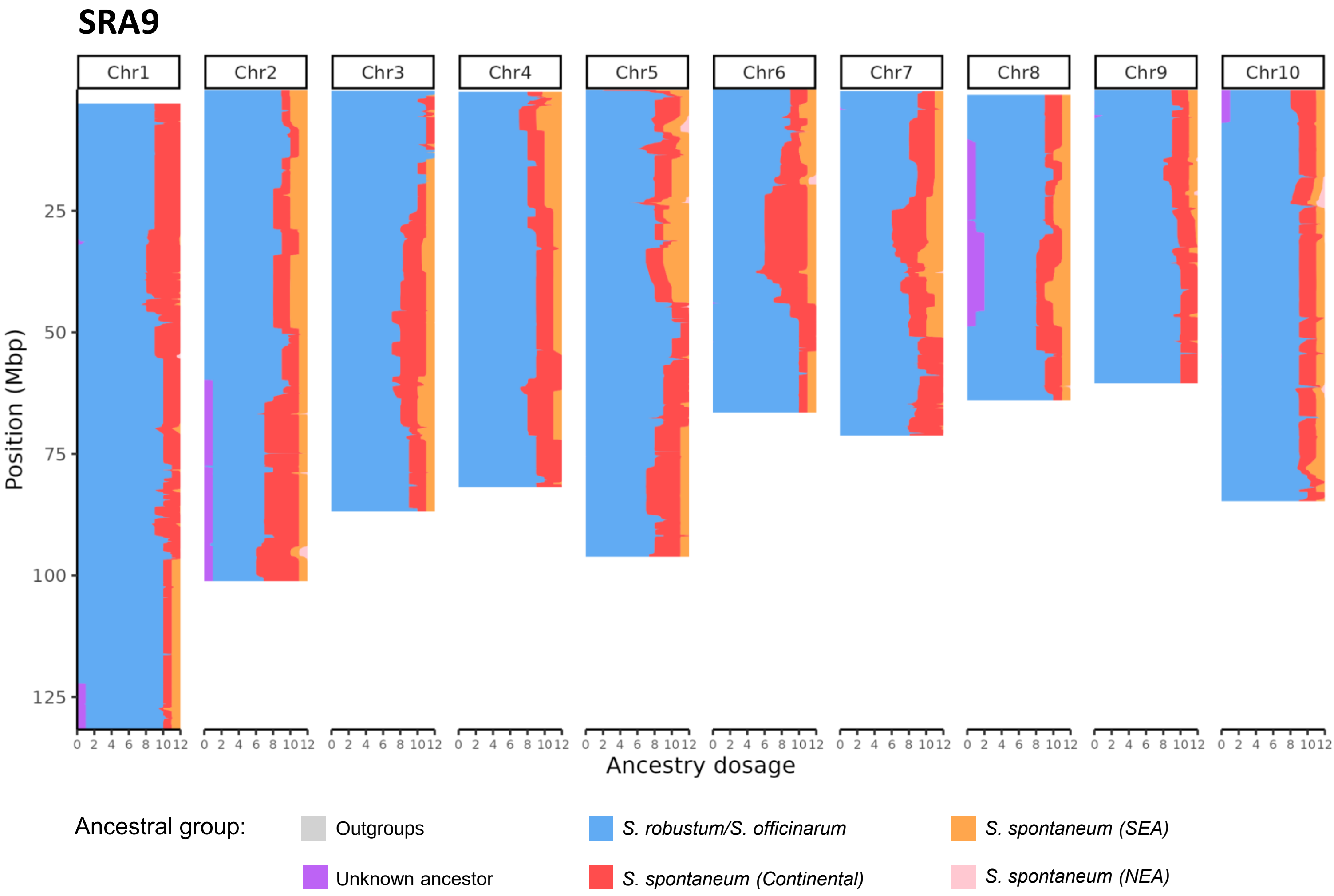

Local admixture of the modern cultivar SRA9

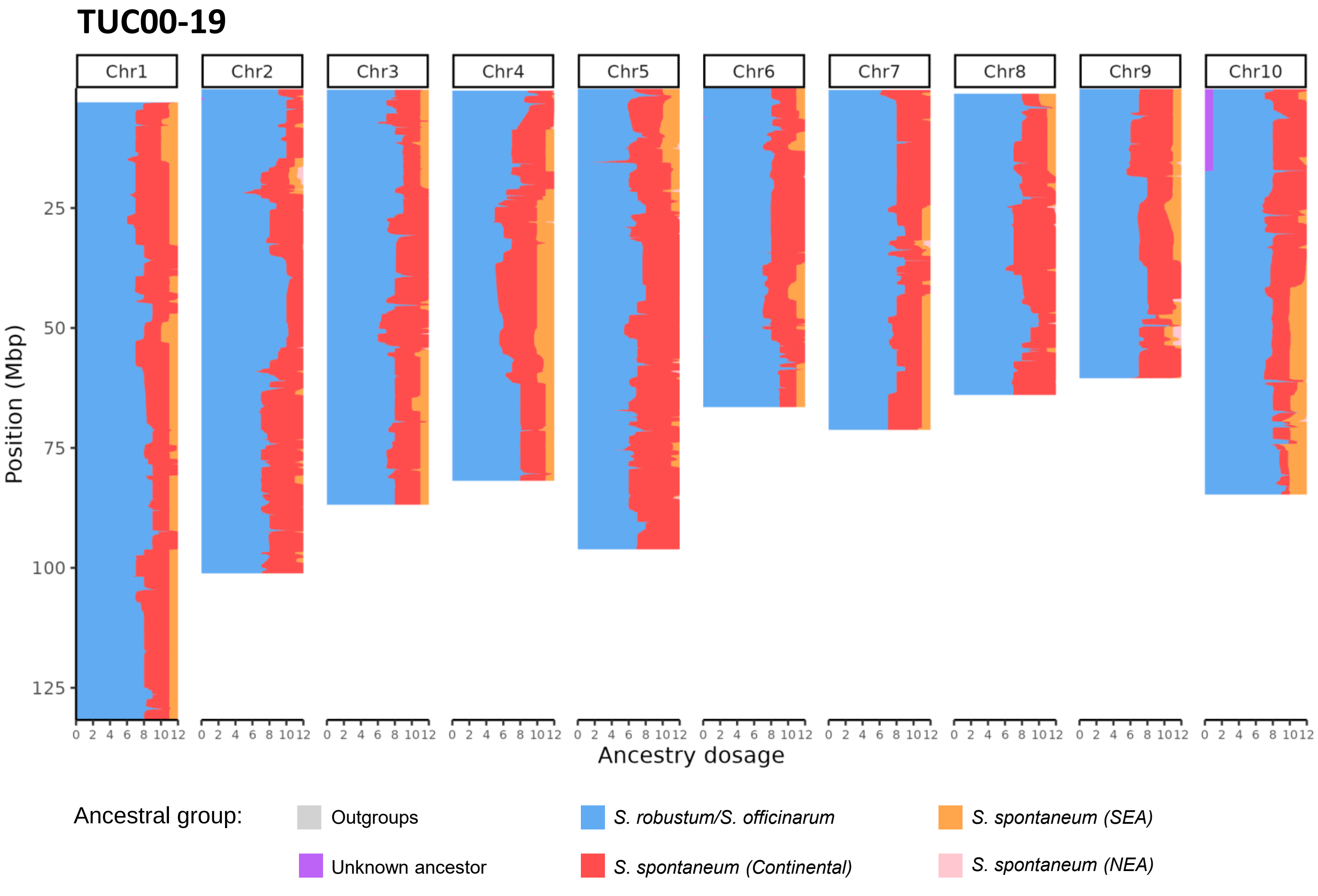

Local admixture of the modern cultivar TUC00-19

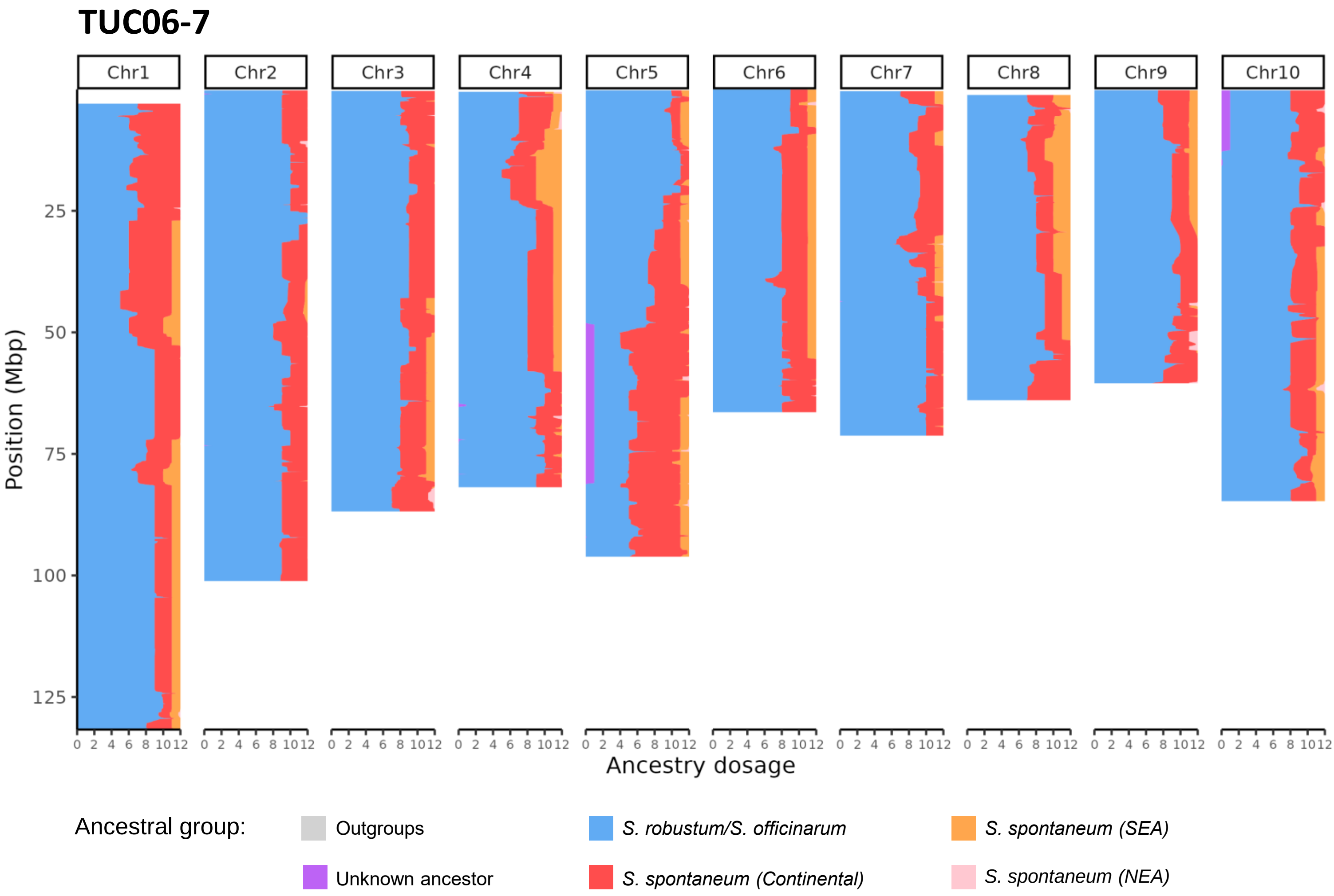

Local admixture of the modern cultivar TUC06-7

TUCCP77-42

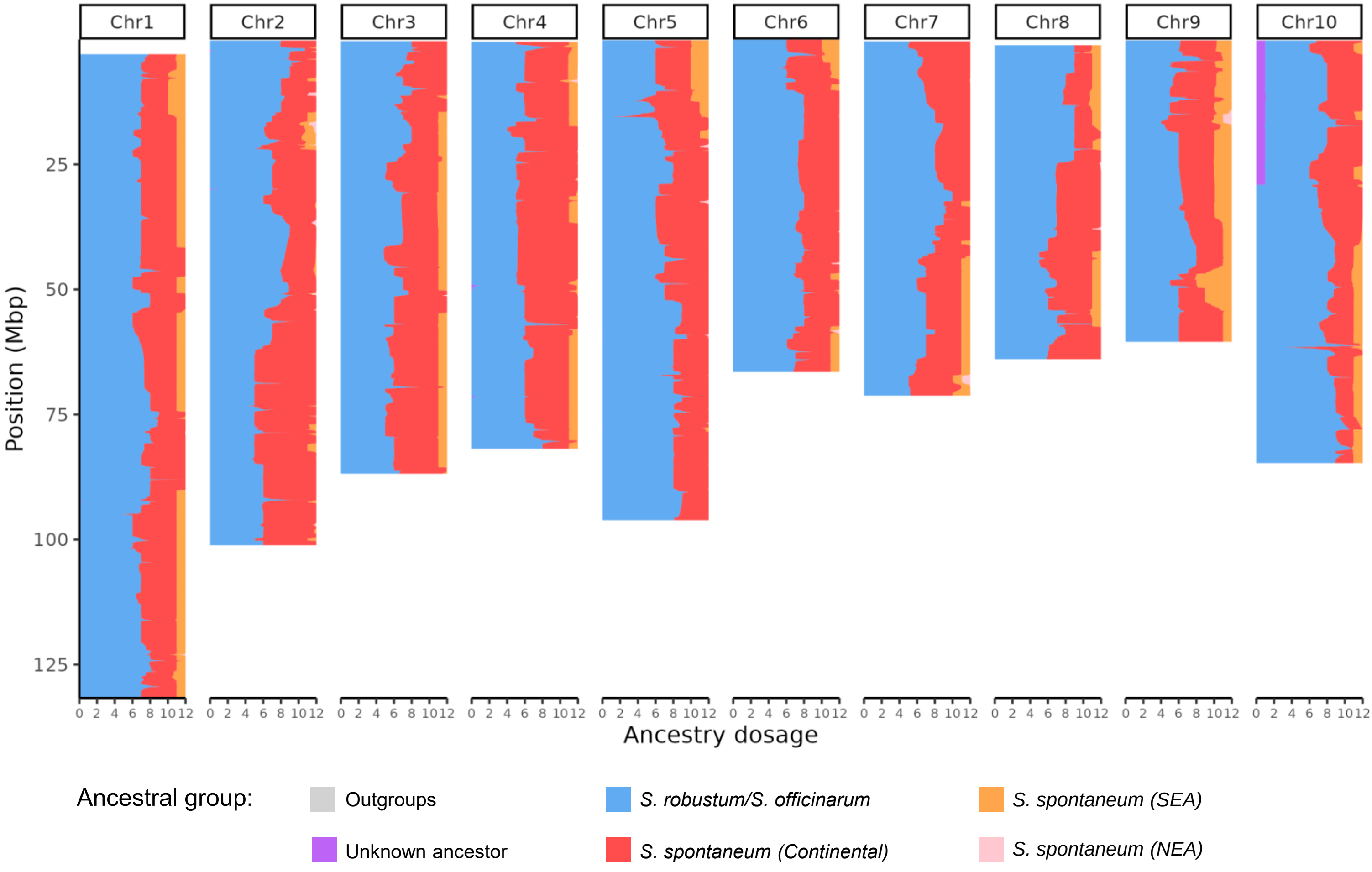

Local admixture of the modern cultivar TUCCP77-42

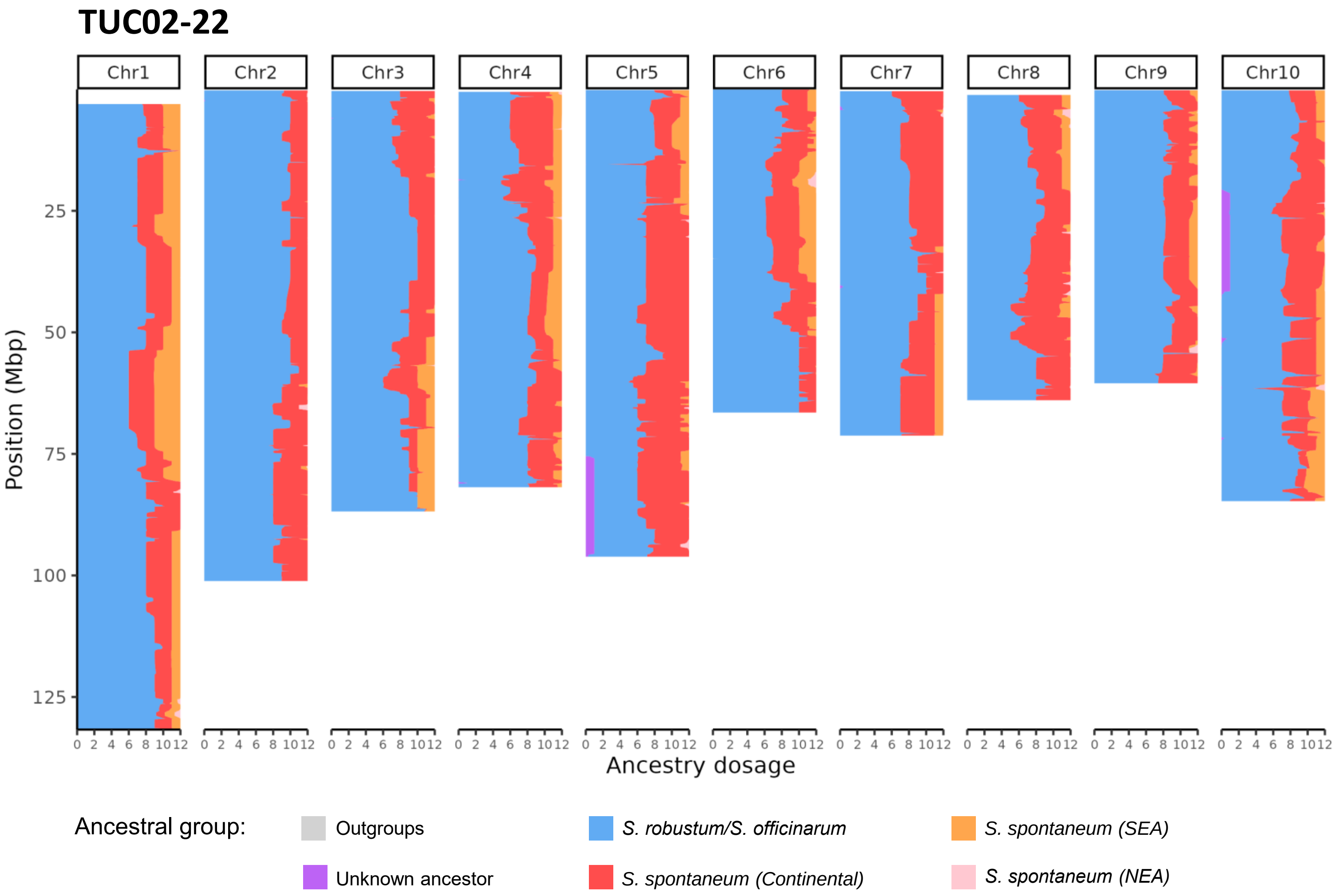

Local admixture of the modern cultivar TUC02-22

TUC03-12

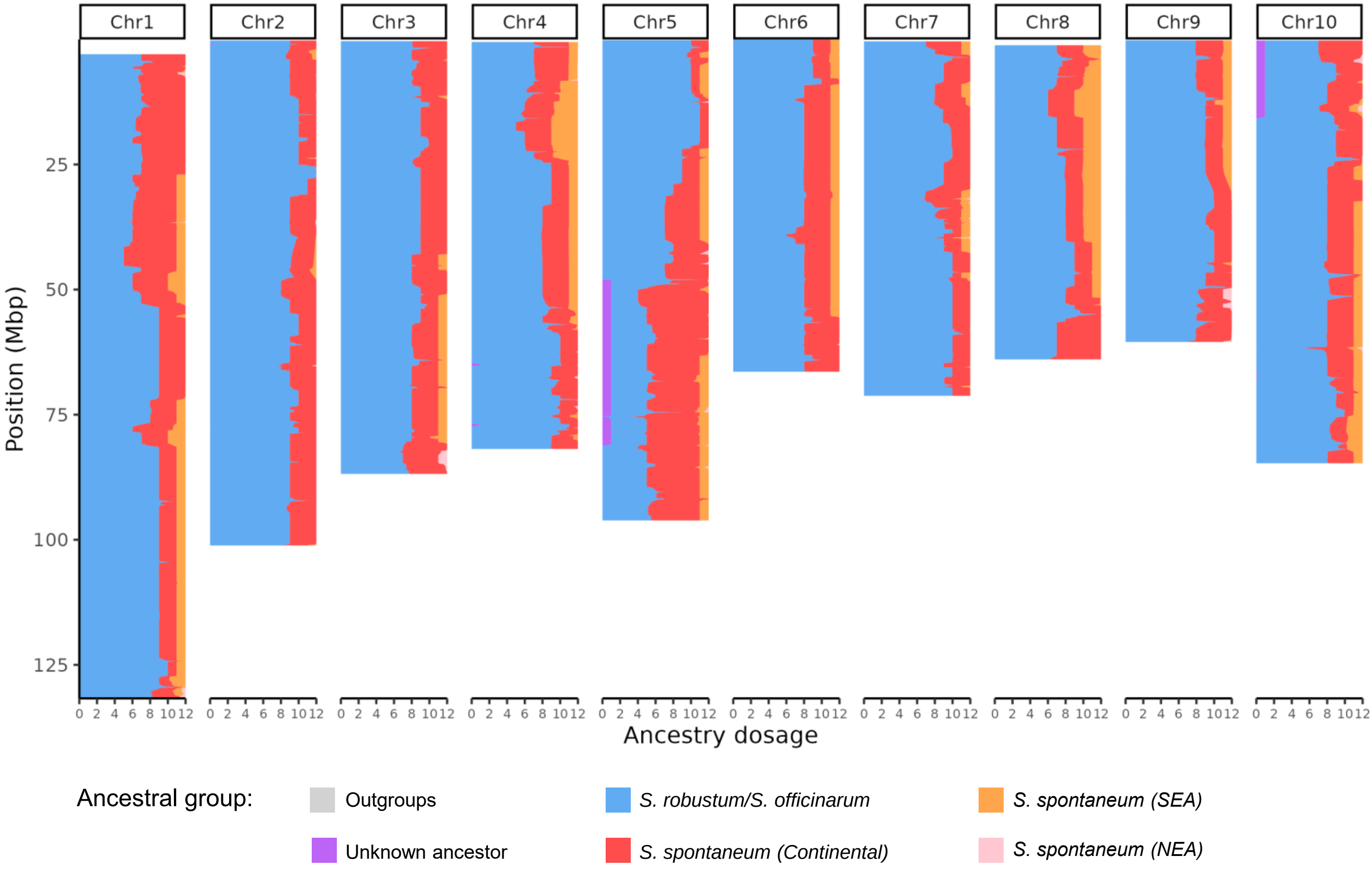

Local admixture of the modern cultivar TUC03-12

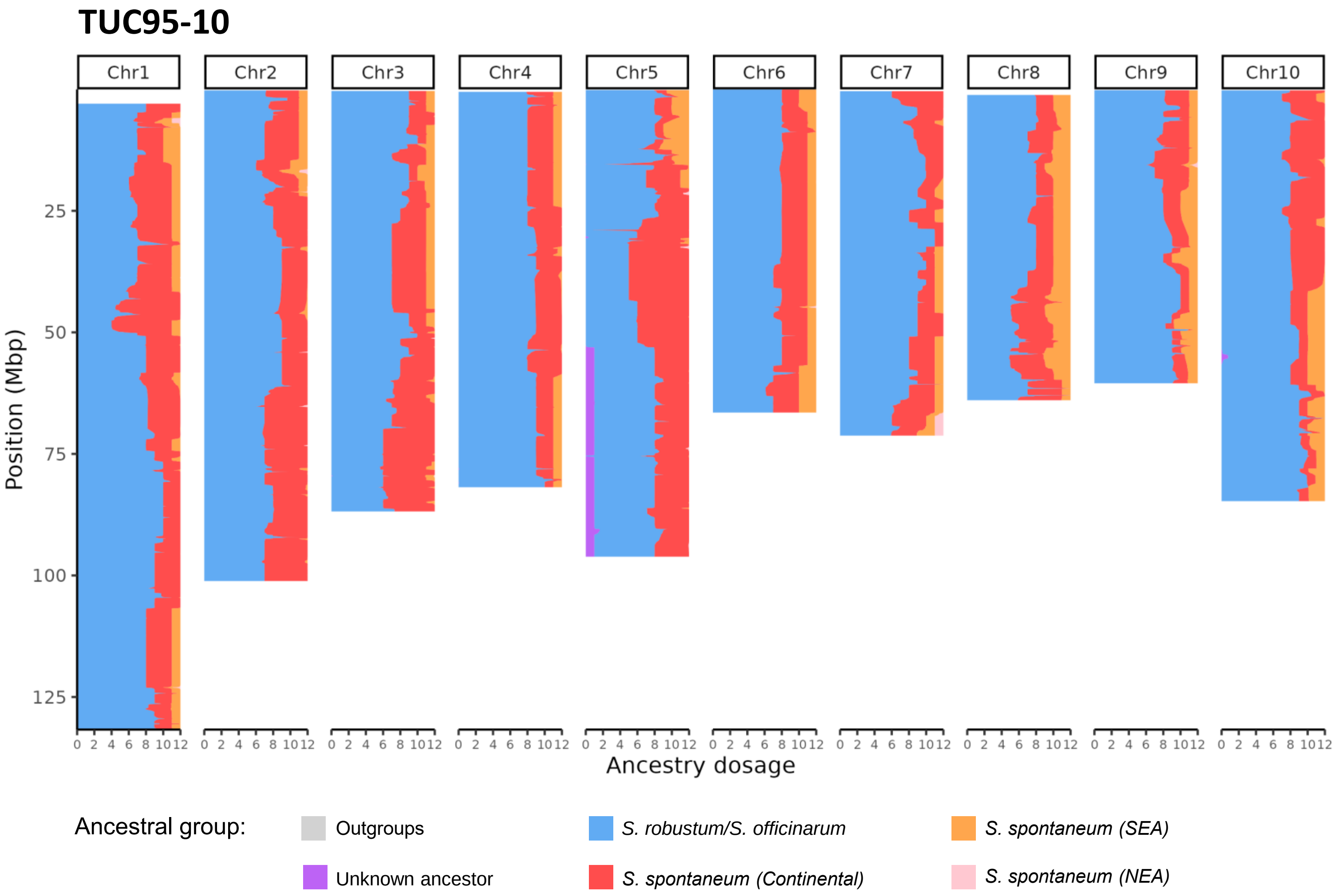

Local admixture of the modern cultivar TUC95-10

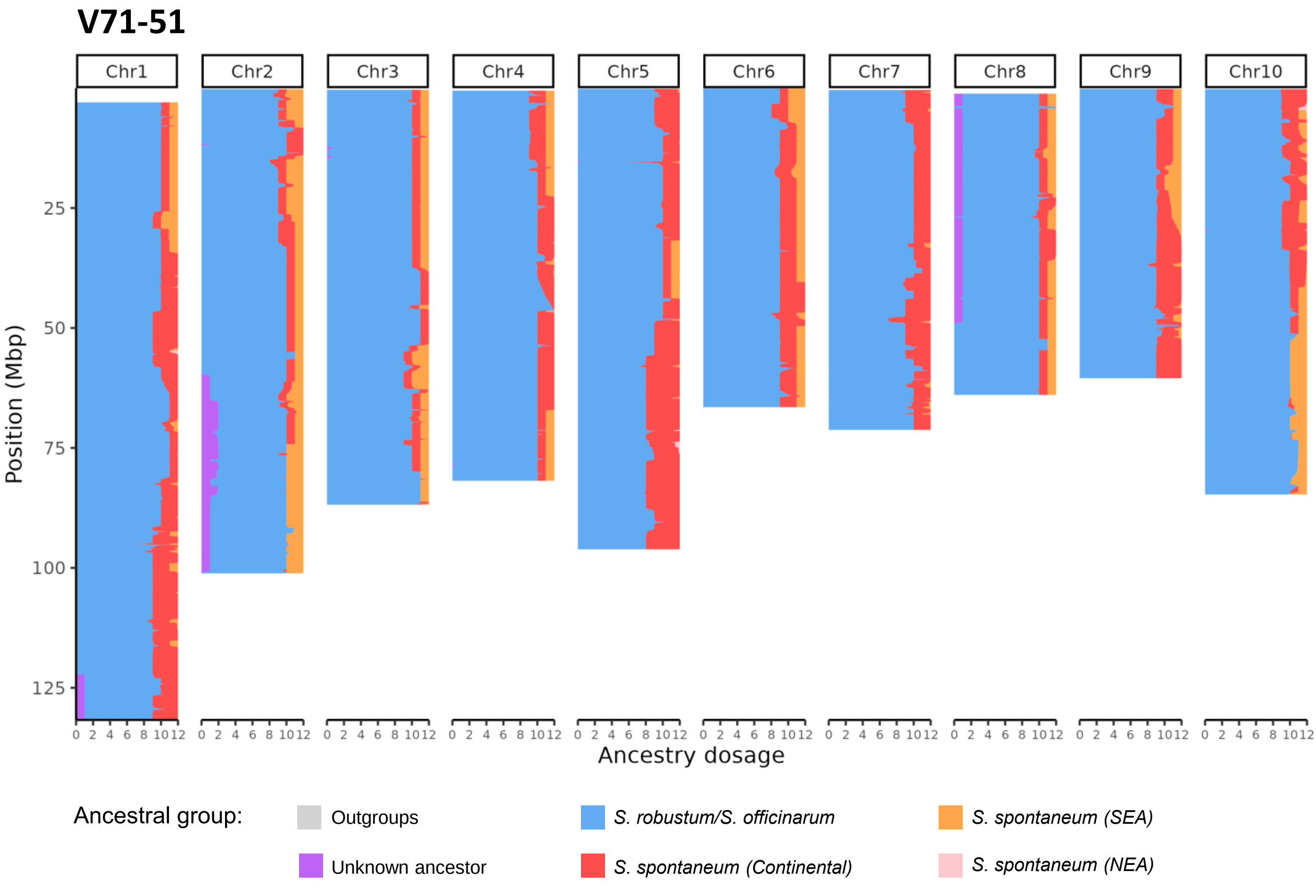

Local admixture of the modern cultivar V71-51

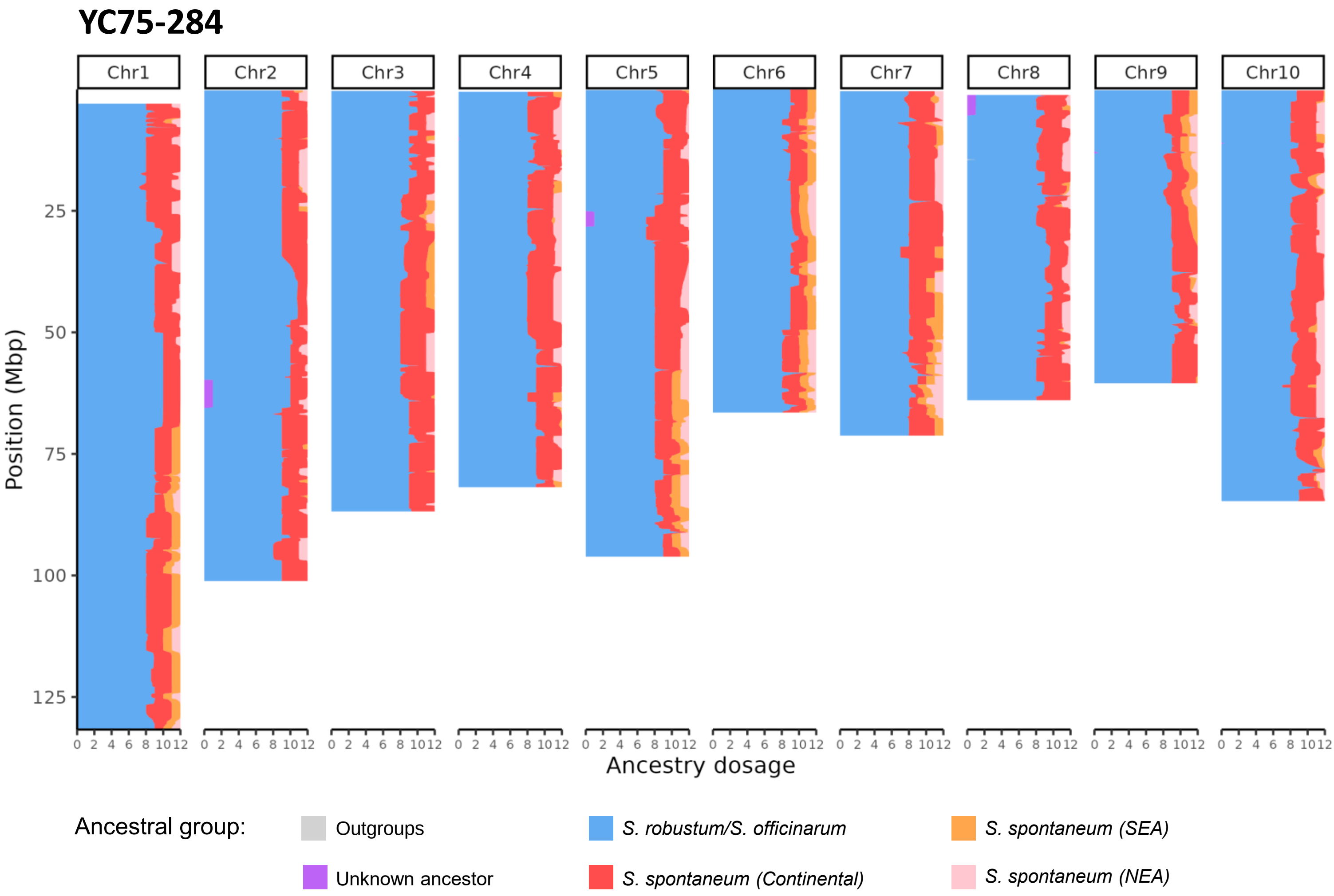

Local admixture of the modern cultivar YC75-284

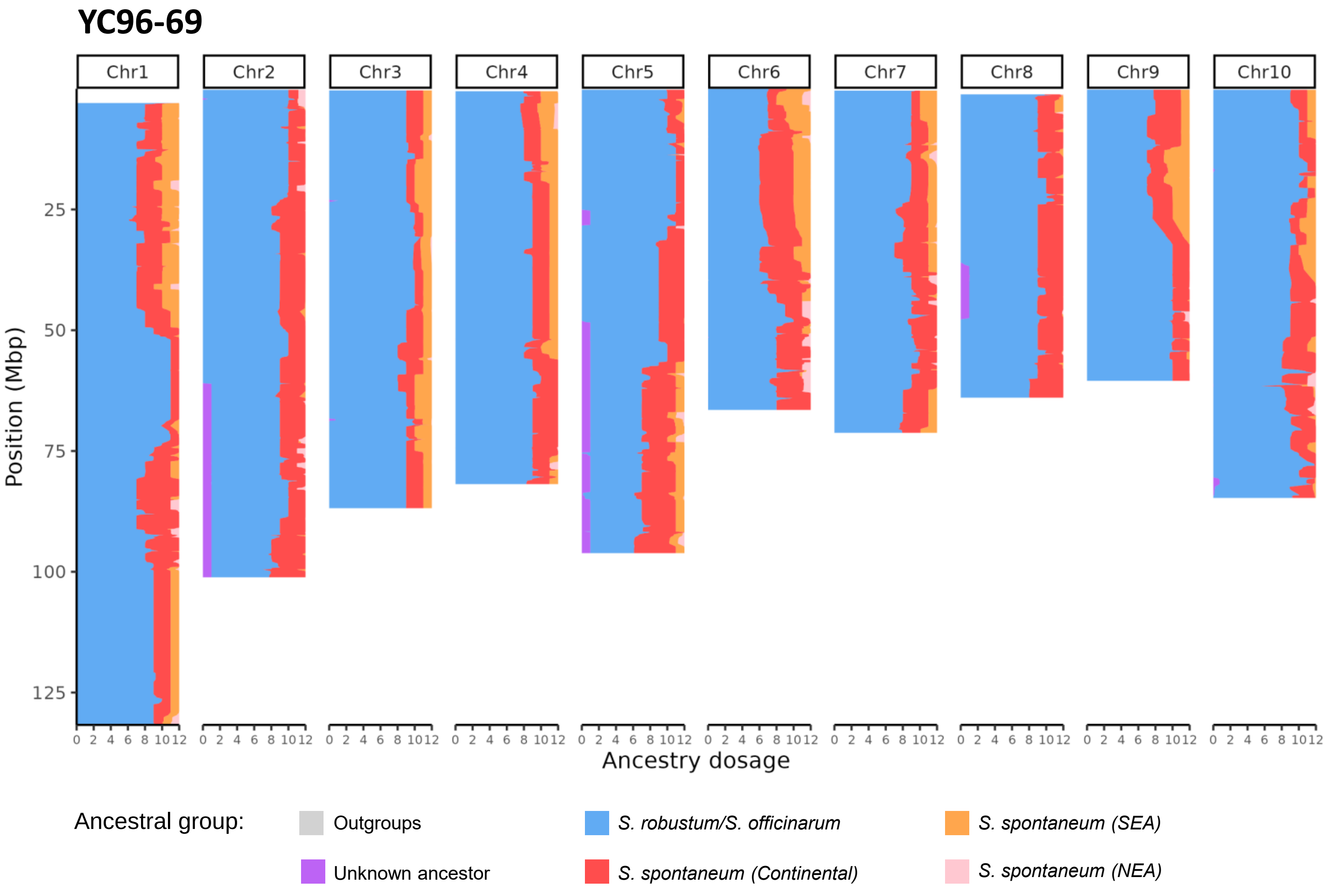

Local admixture of the modern cultivar YC96-69

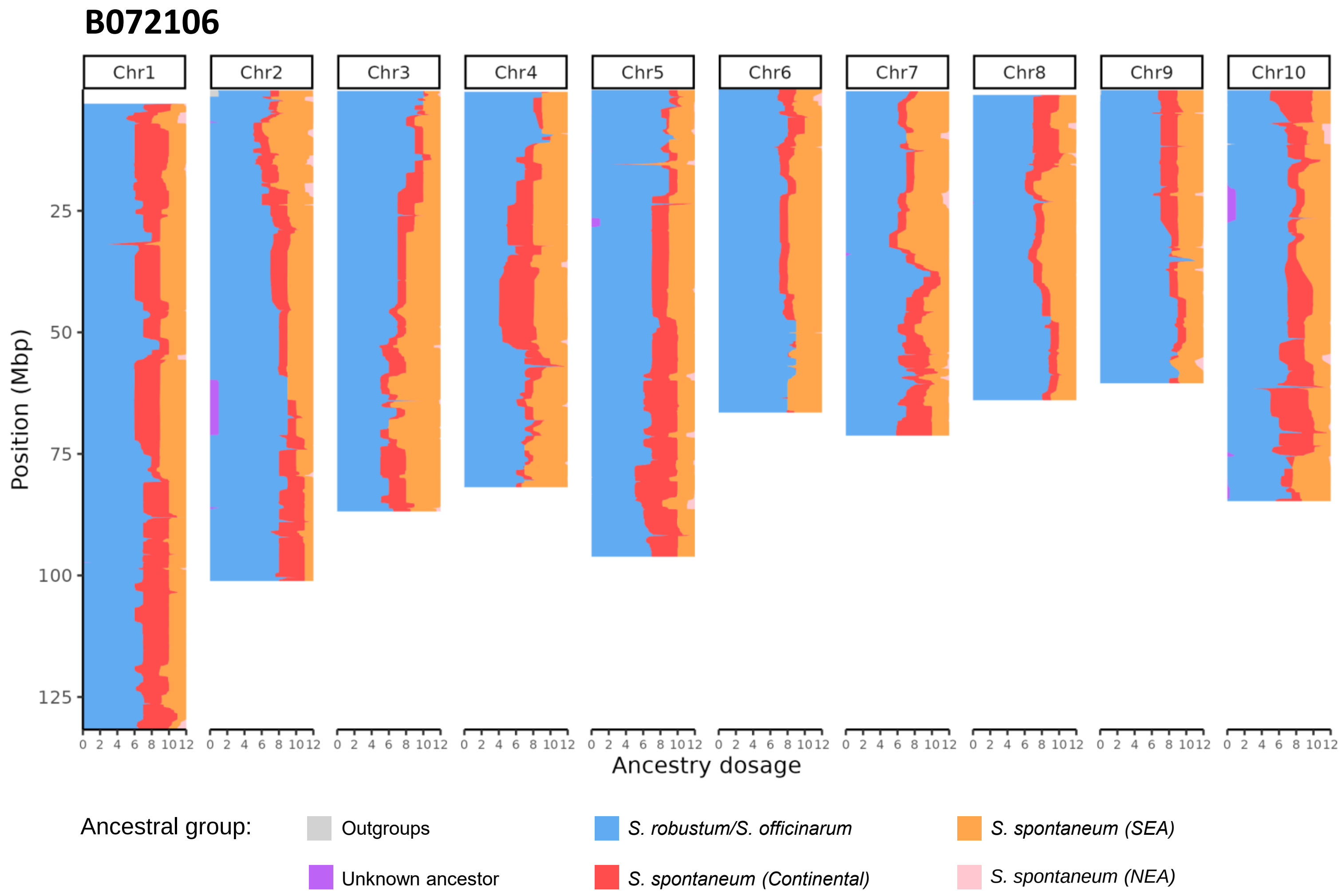

Local admixture of the hybrid B072106

B072014

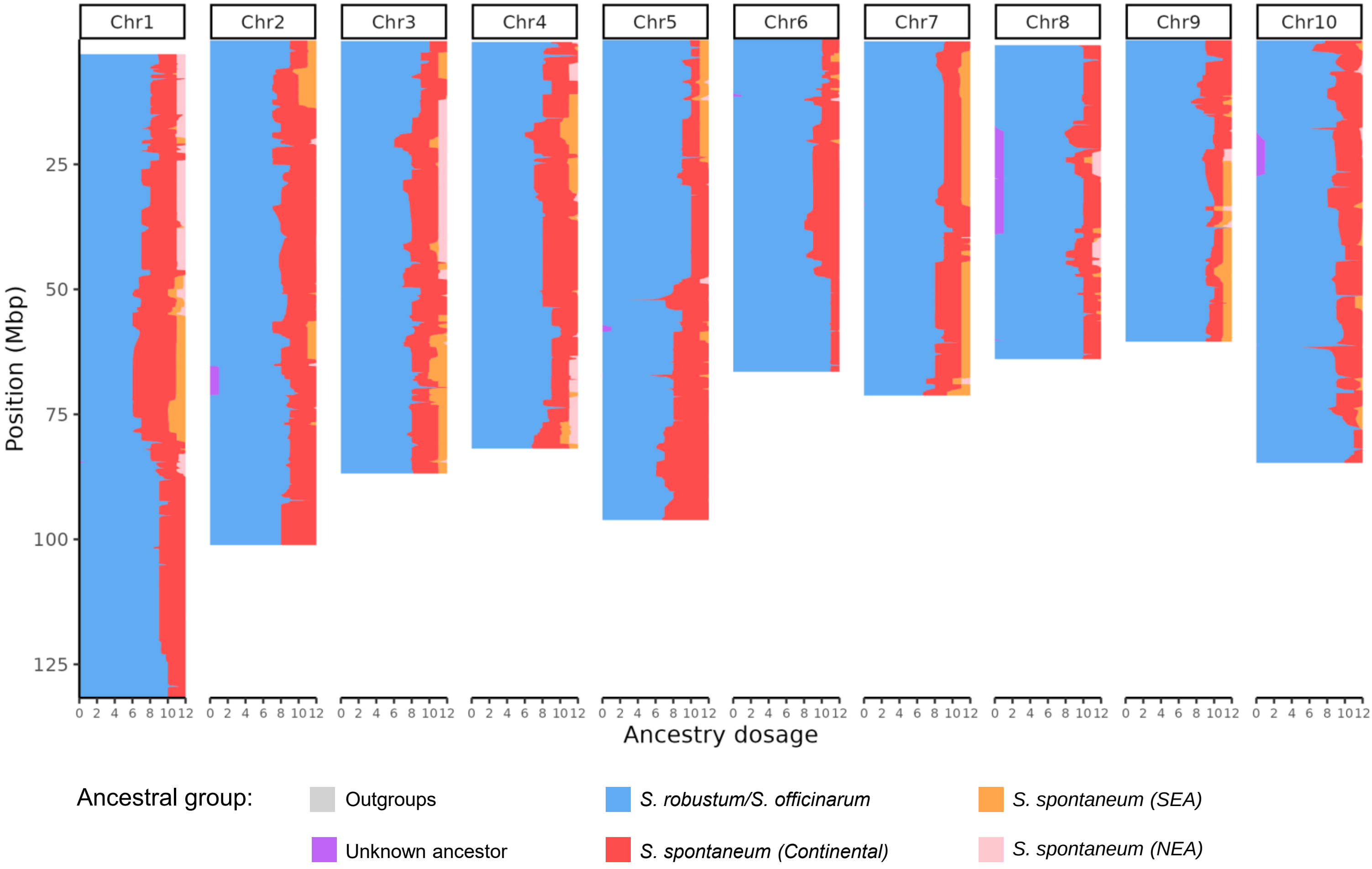

Local admixture of the hybrid B072106

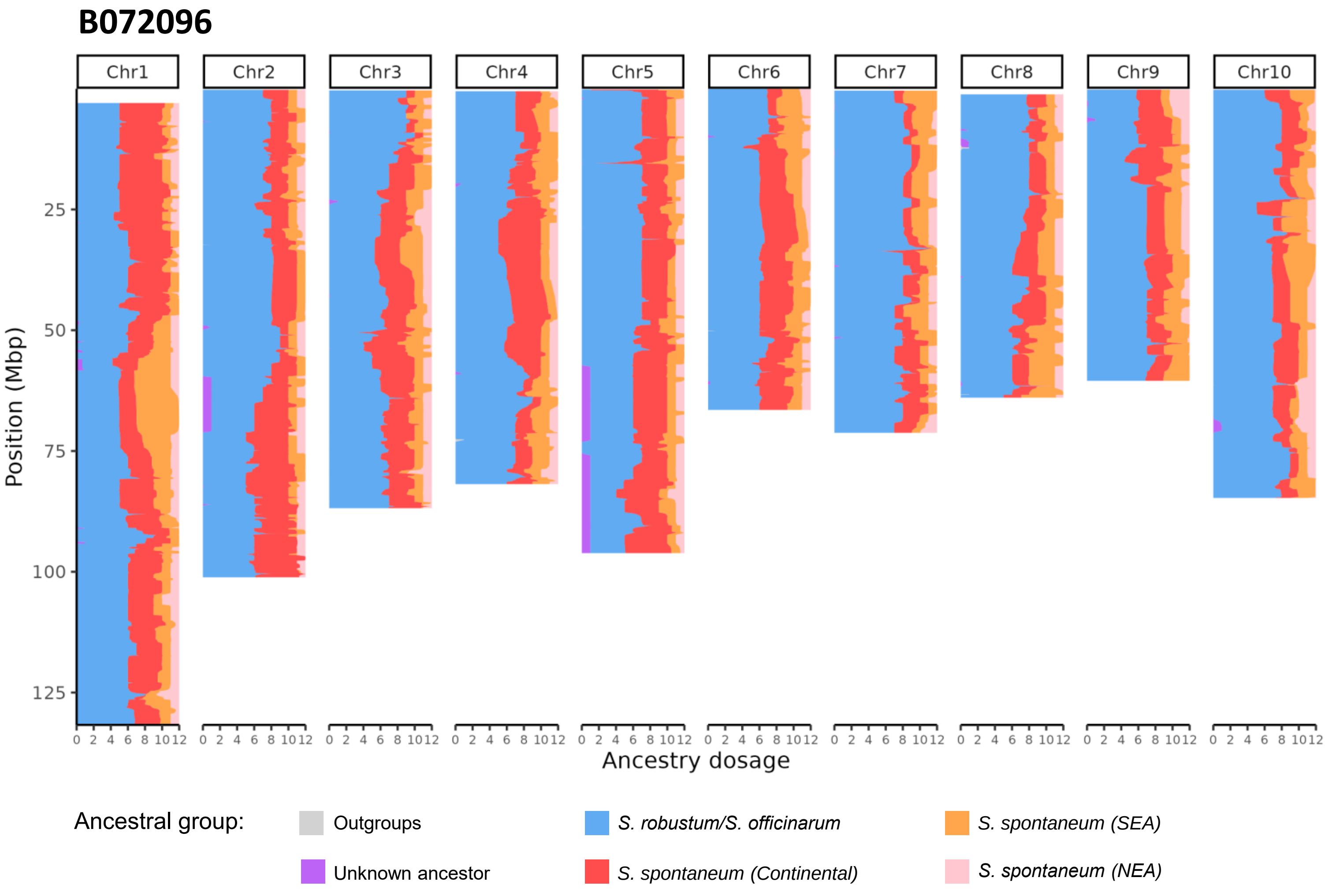

Local admixture of the hybrid B072096

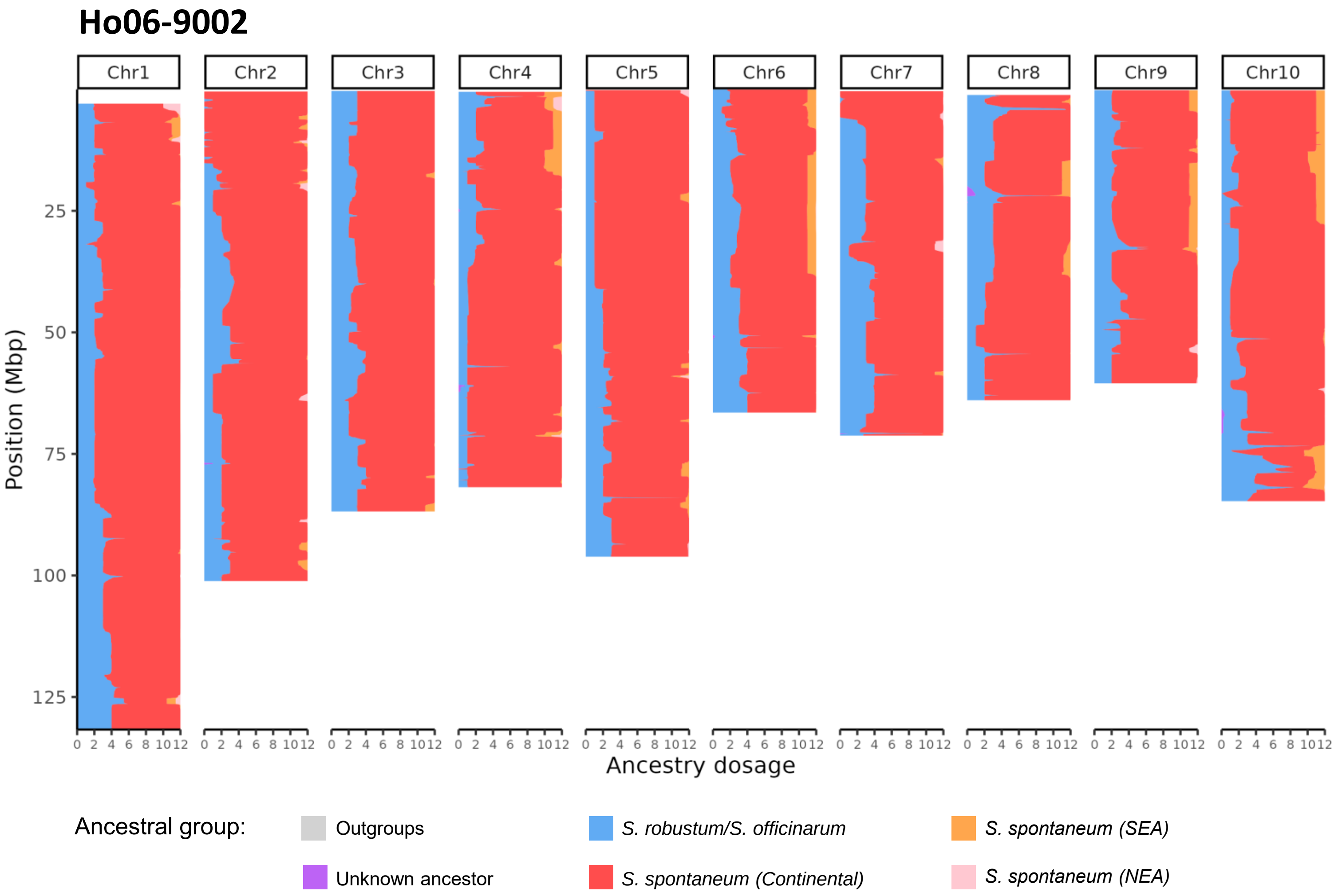

Local admixture of the hybrid Ho06-9002

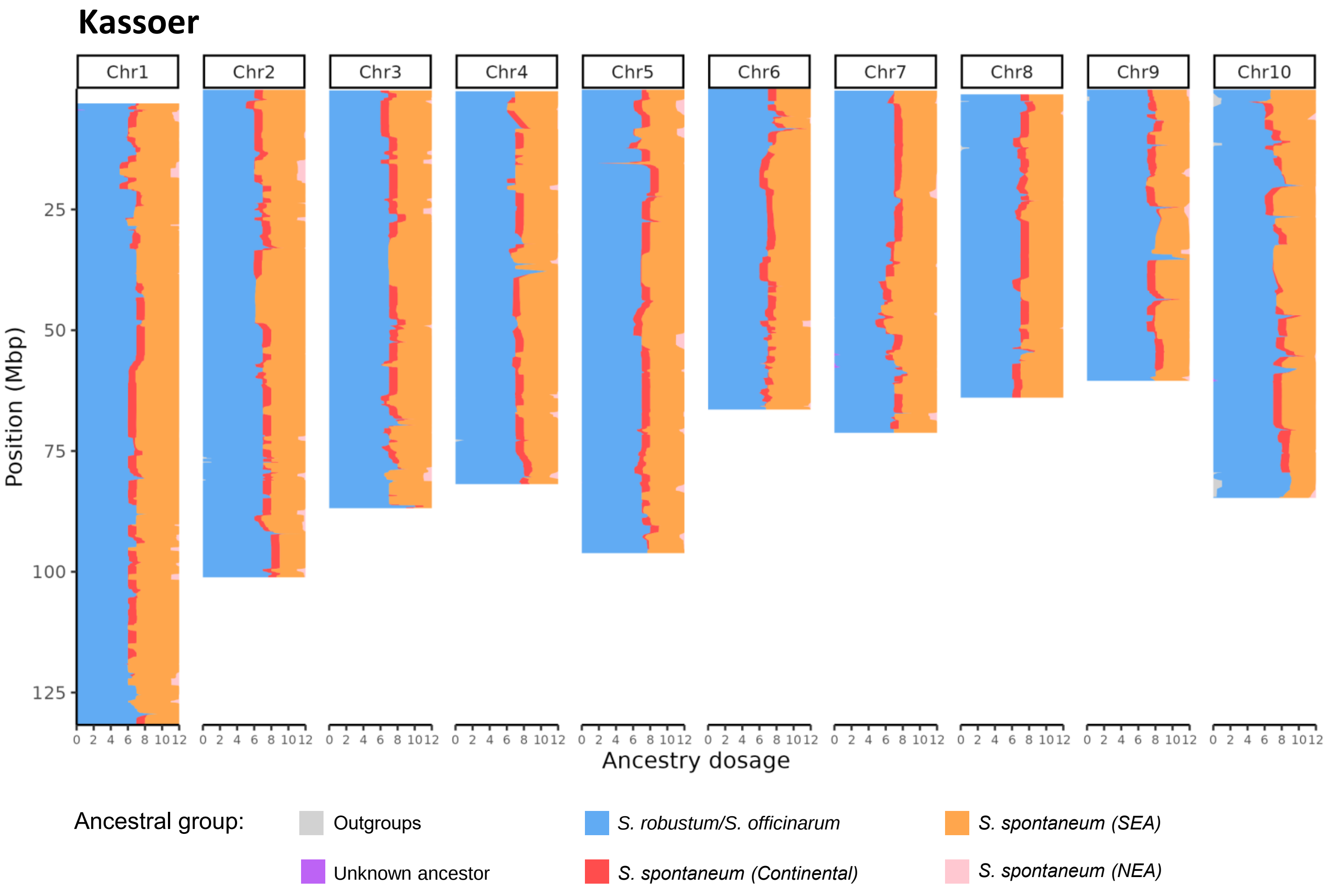

Local admixture of the hybrid Kassoer

M18-09

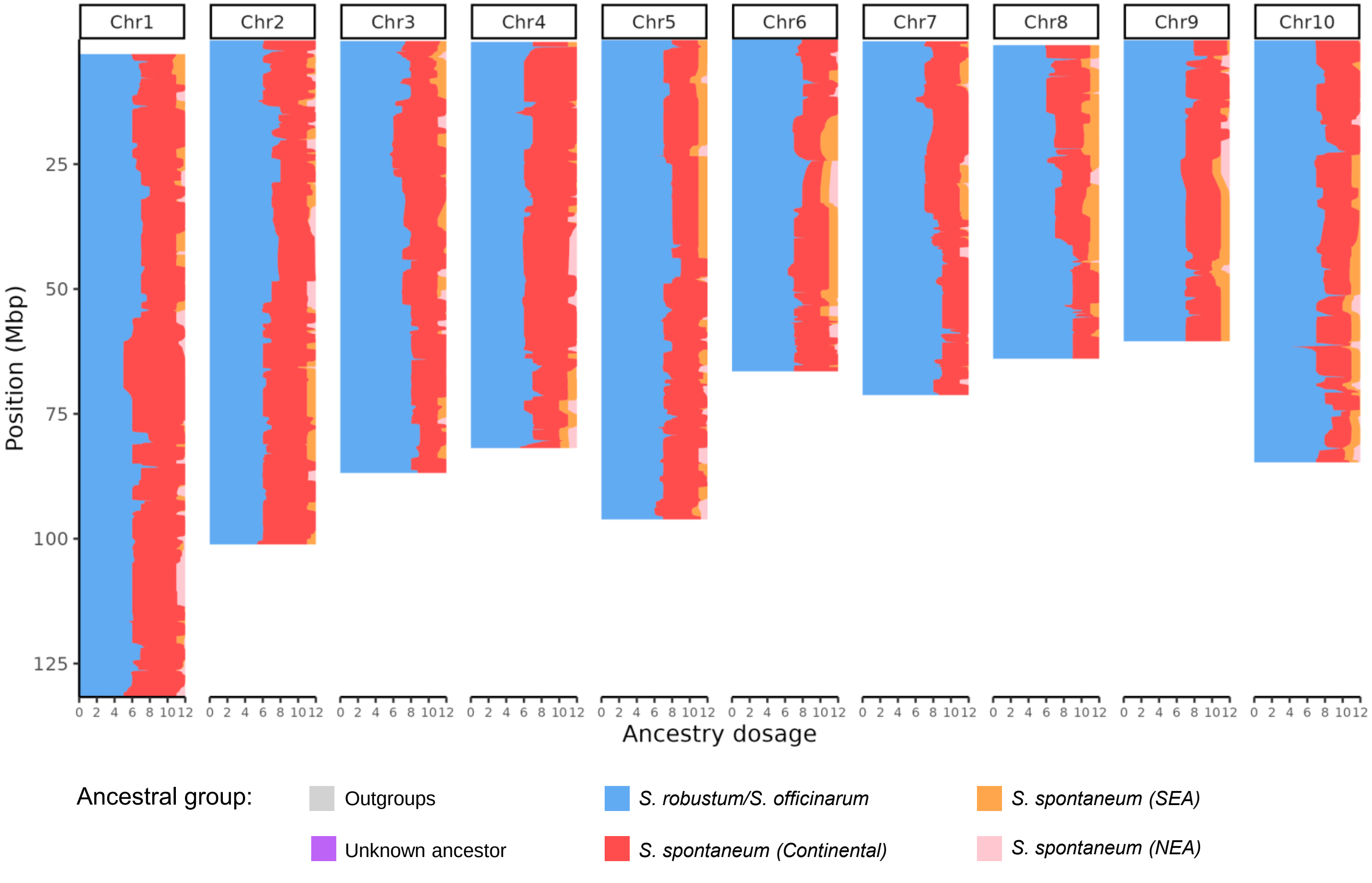

Local admixture of the hybrid M18-09

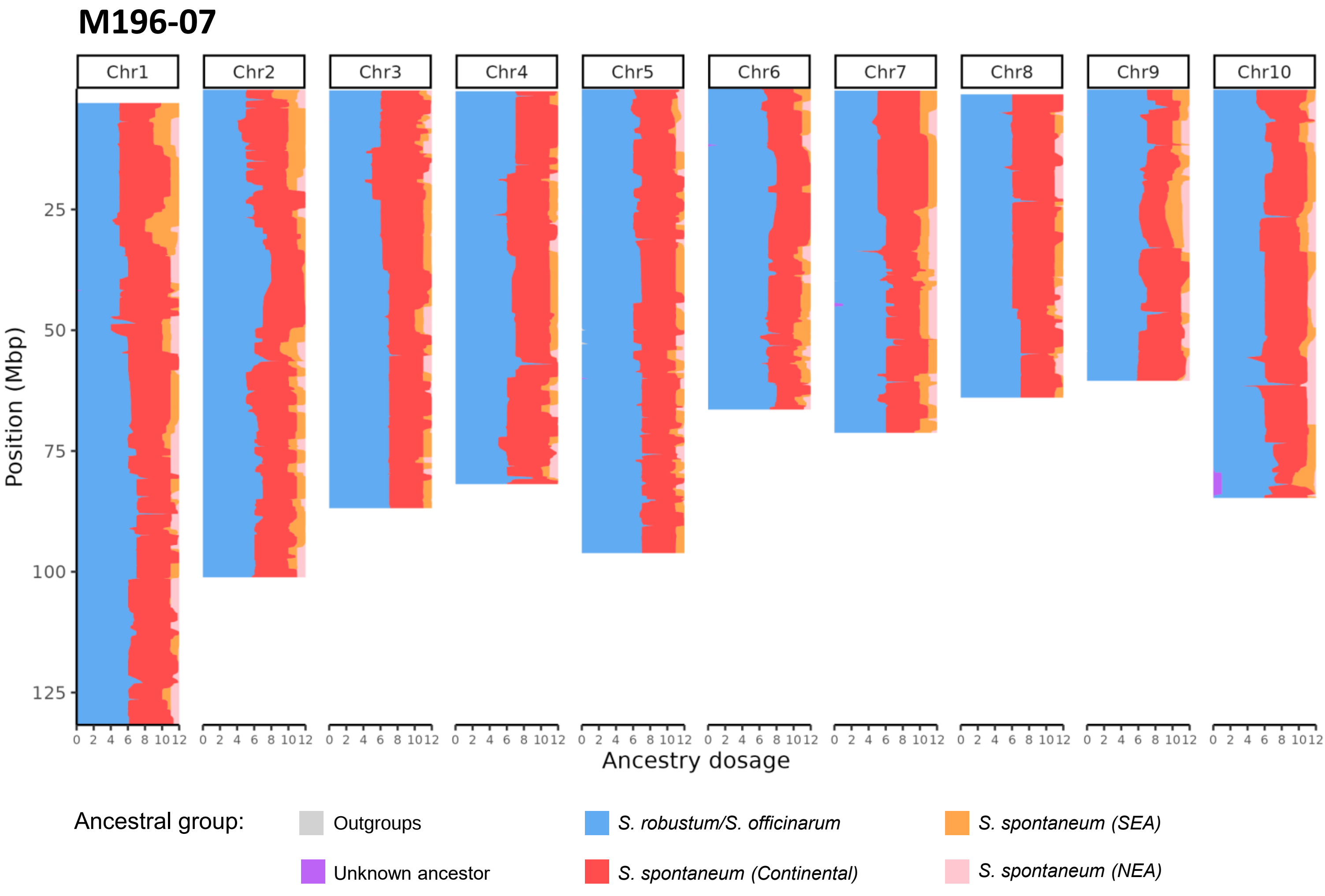

Local admixture of the hybrid M196-07

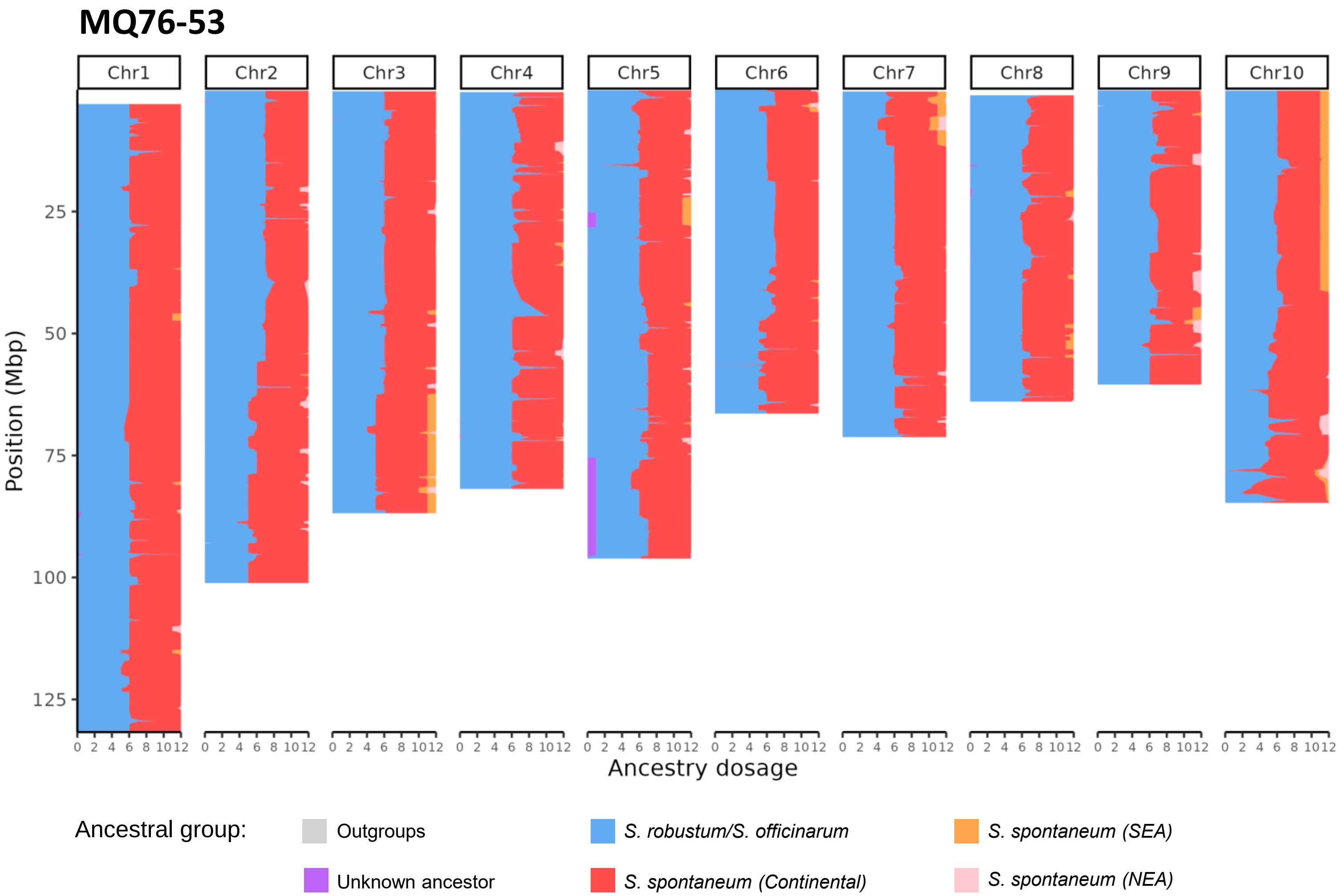

Local admixture of the hybrid MQ76-53

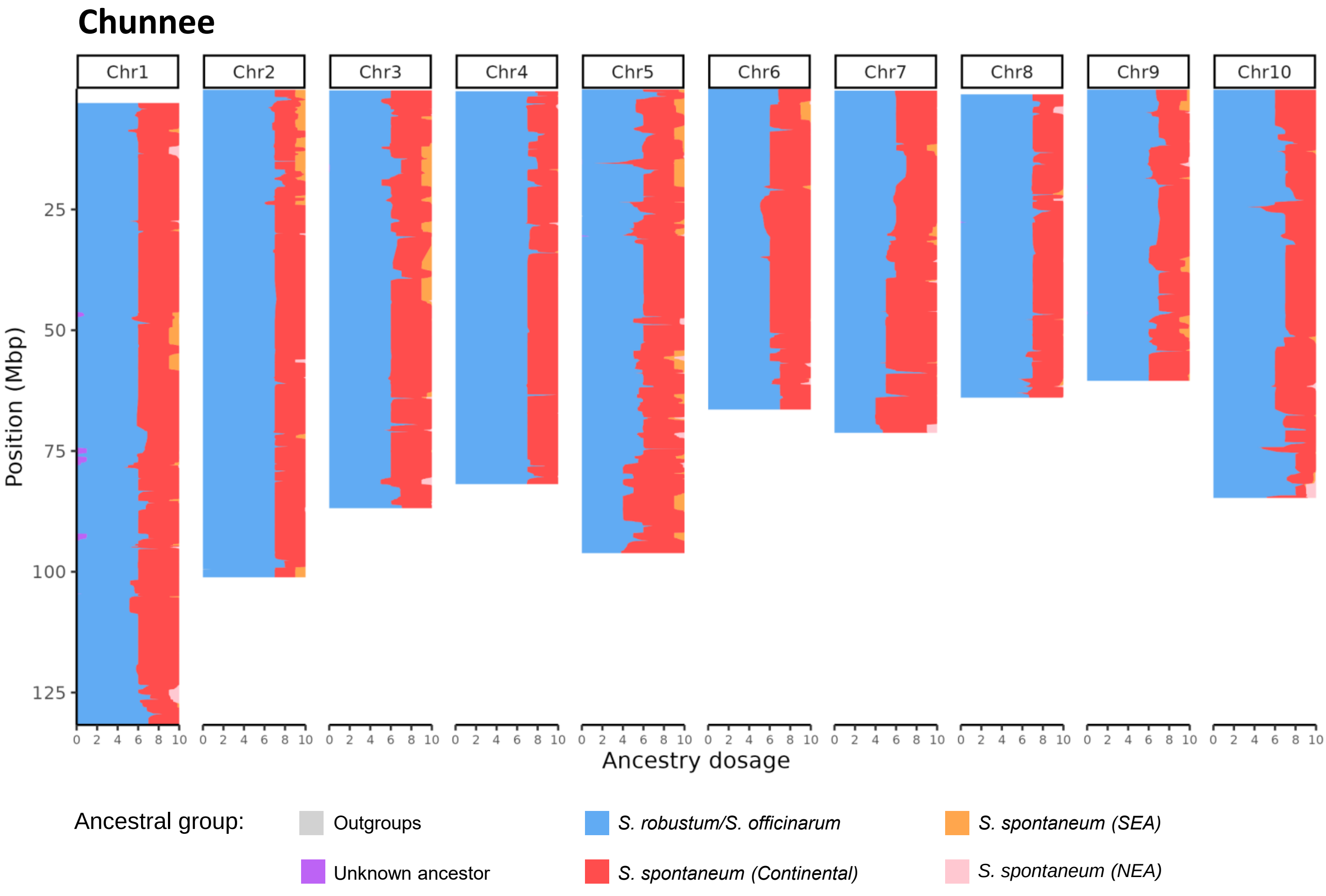

Local admixture of the *S. barberi* Chunnee

Katha8232

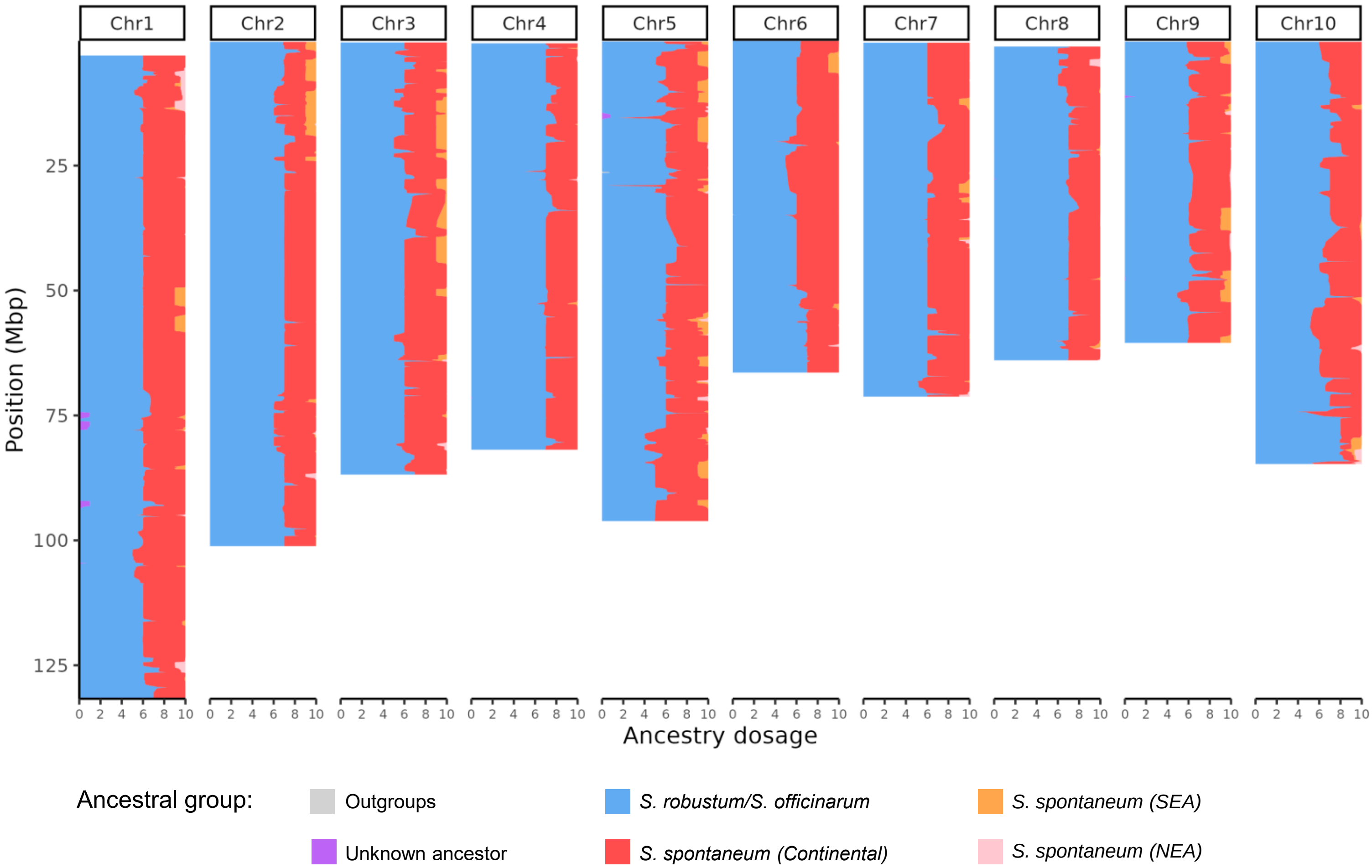

Local admixture of the *S. barberi* Chunnee

Saretha

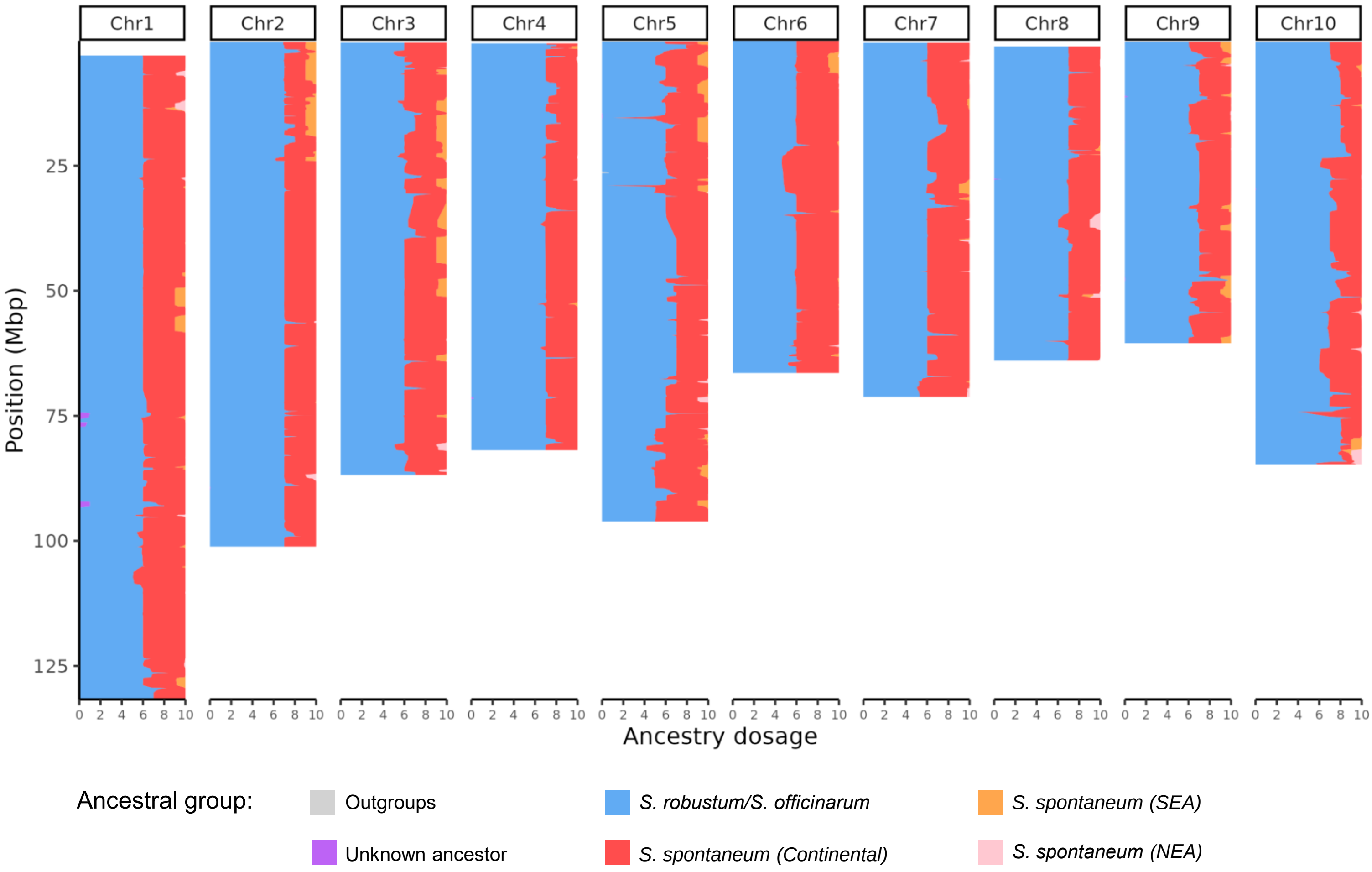

Local admixture of the *S. barberi* Saretha

Agoule

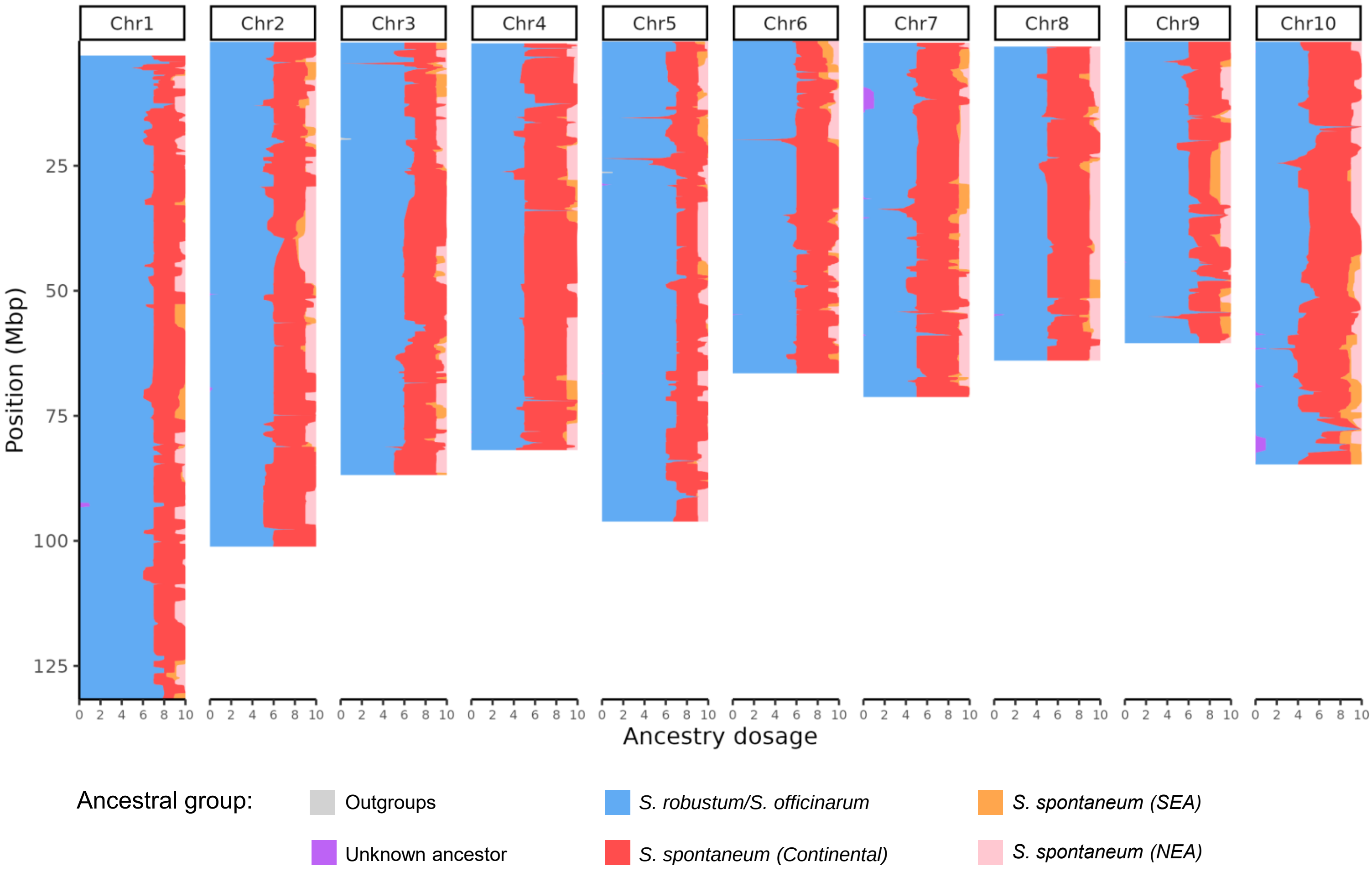

Local admixture of the *S. barberi* Agoule

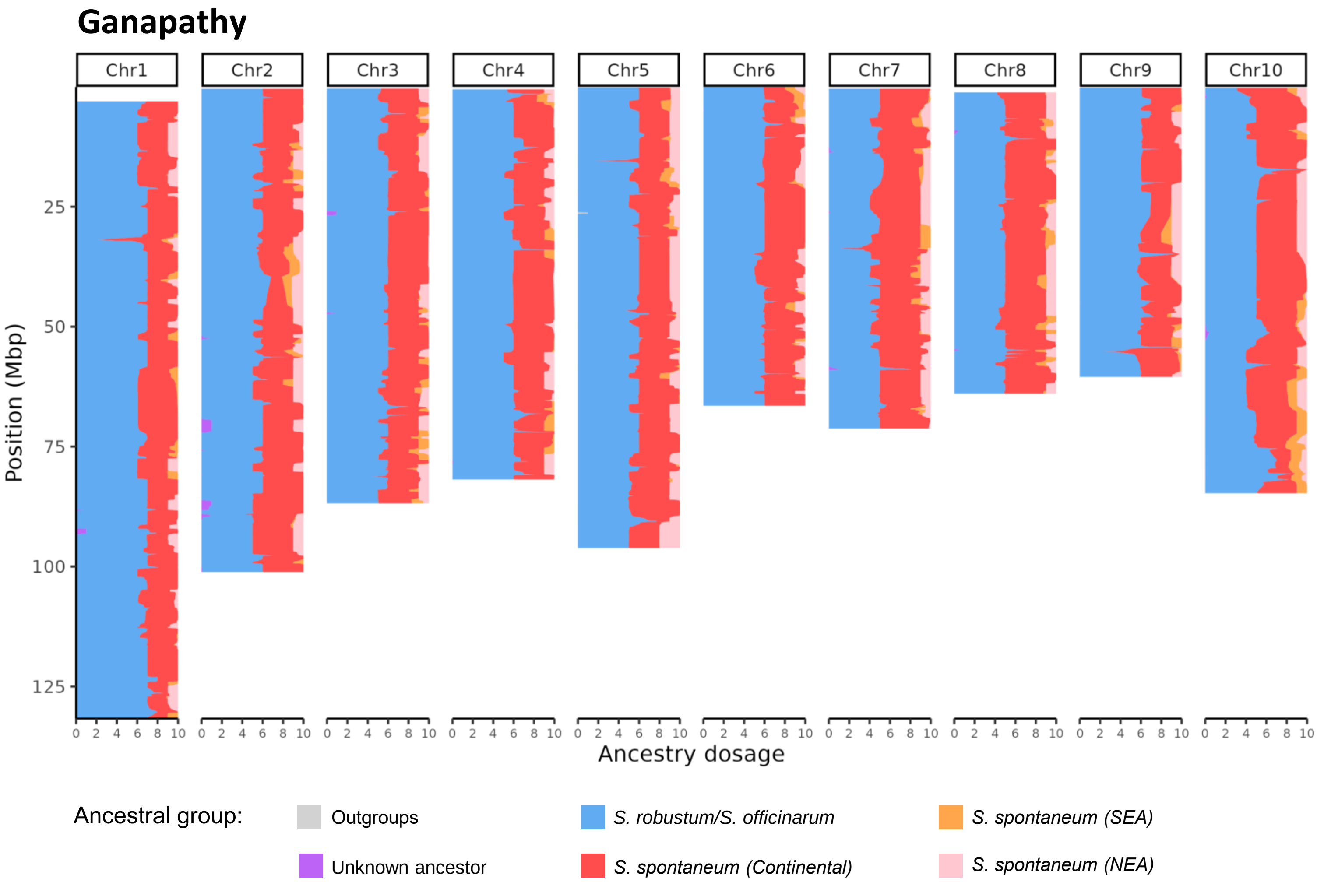

Local admixture of the *S. barberi* Ganapathy

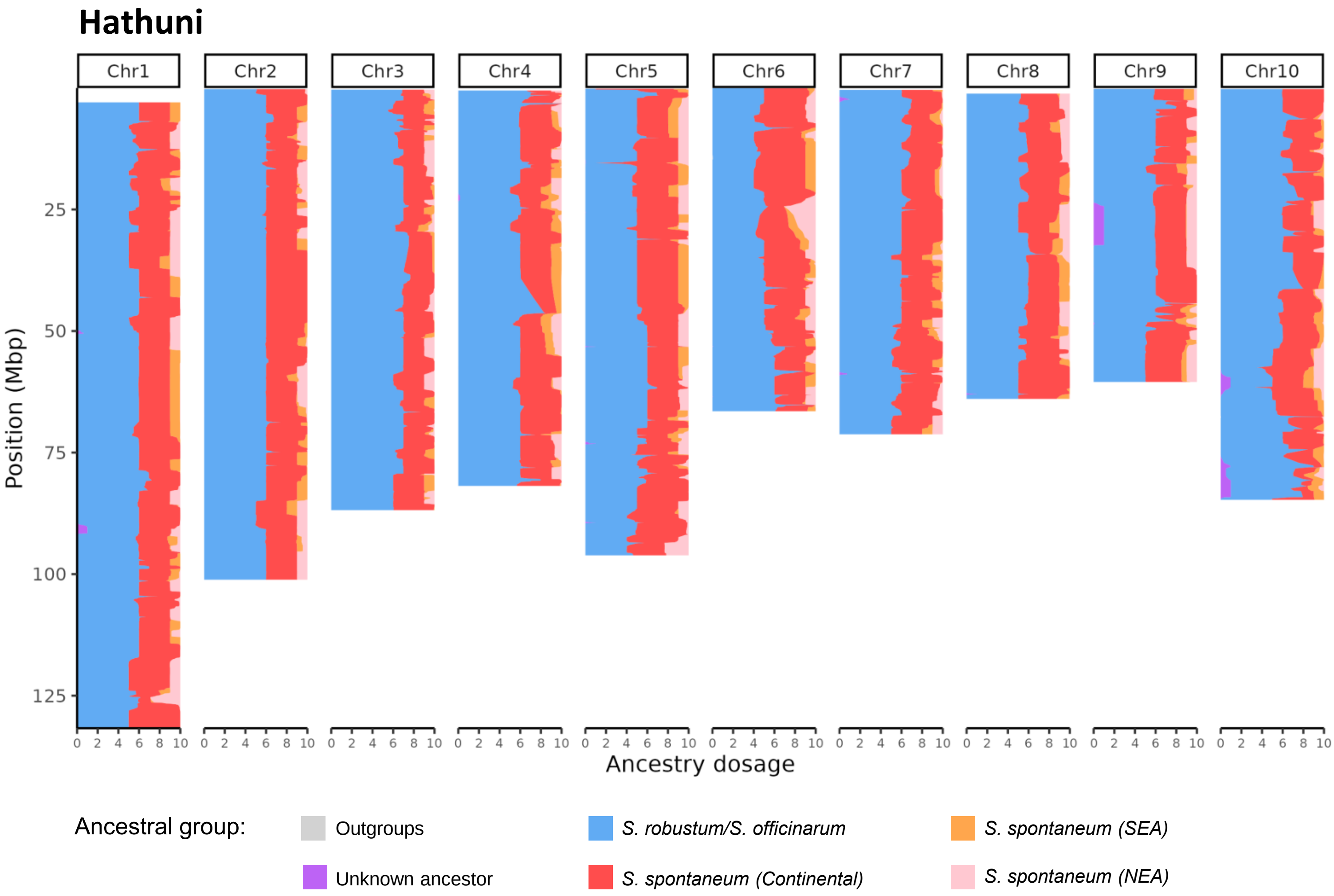

Local admixture of the *S. barberi* Hathuni

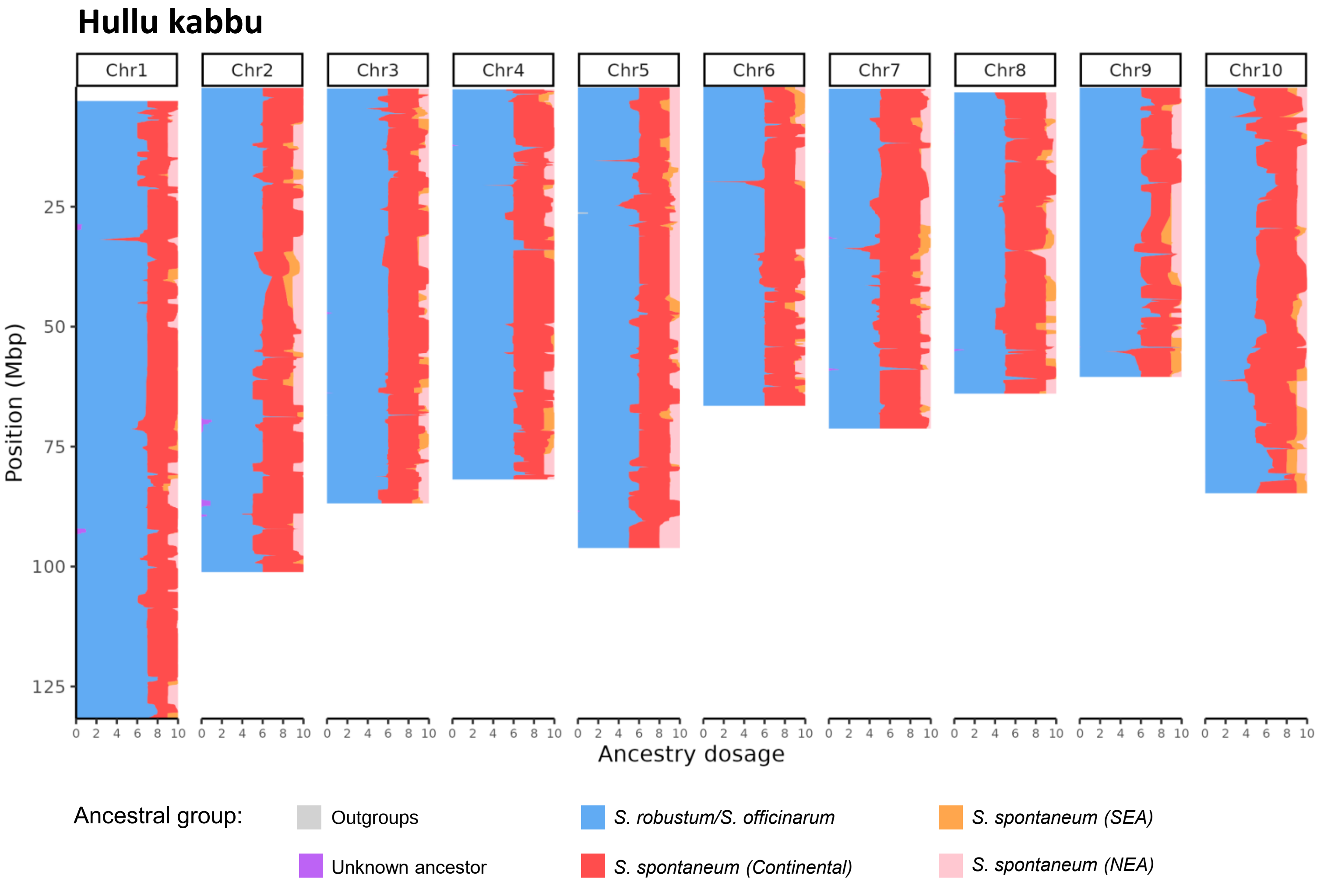

Local admixture of the *S. barberi* Hullu kabbu

Kewali

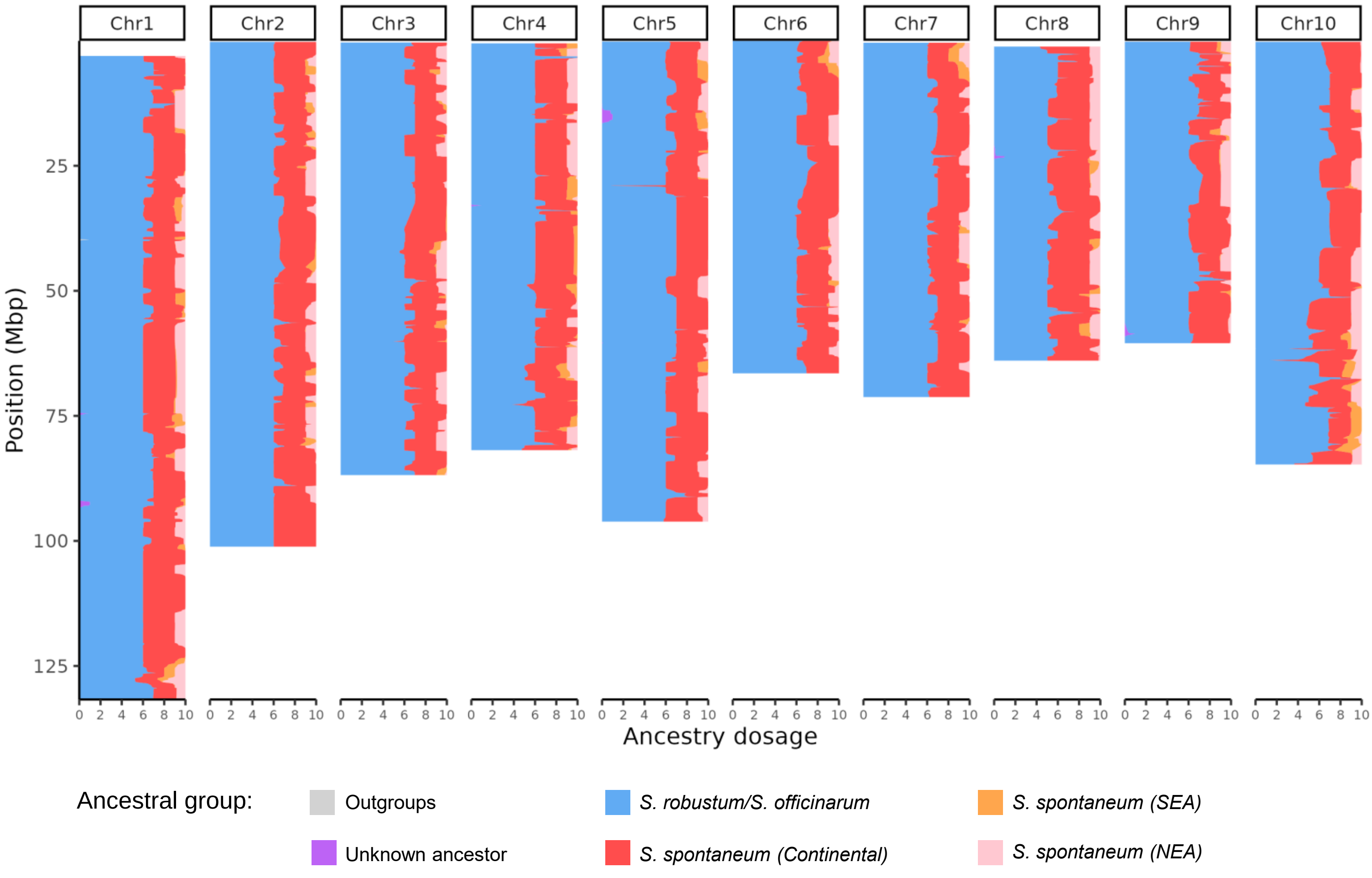

Local admixture of the *S. barberi* Kewali

Matki Mango

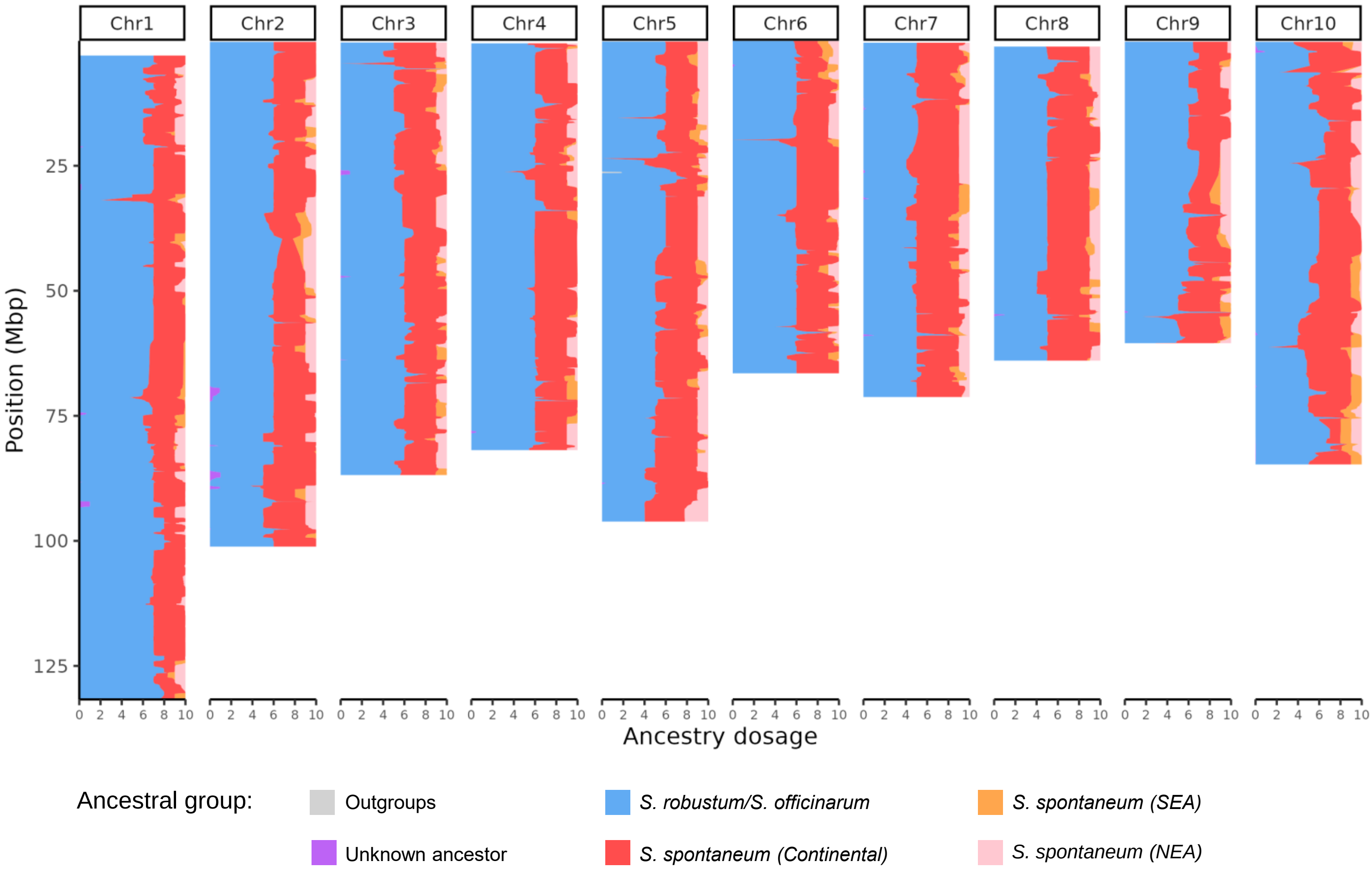

Local admixture of the *S. barberi* Matki Mango

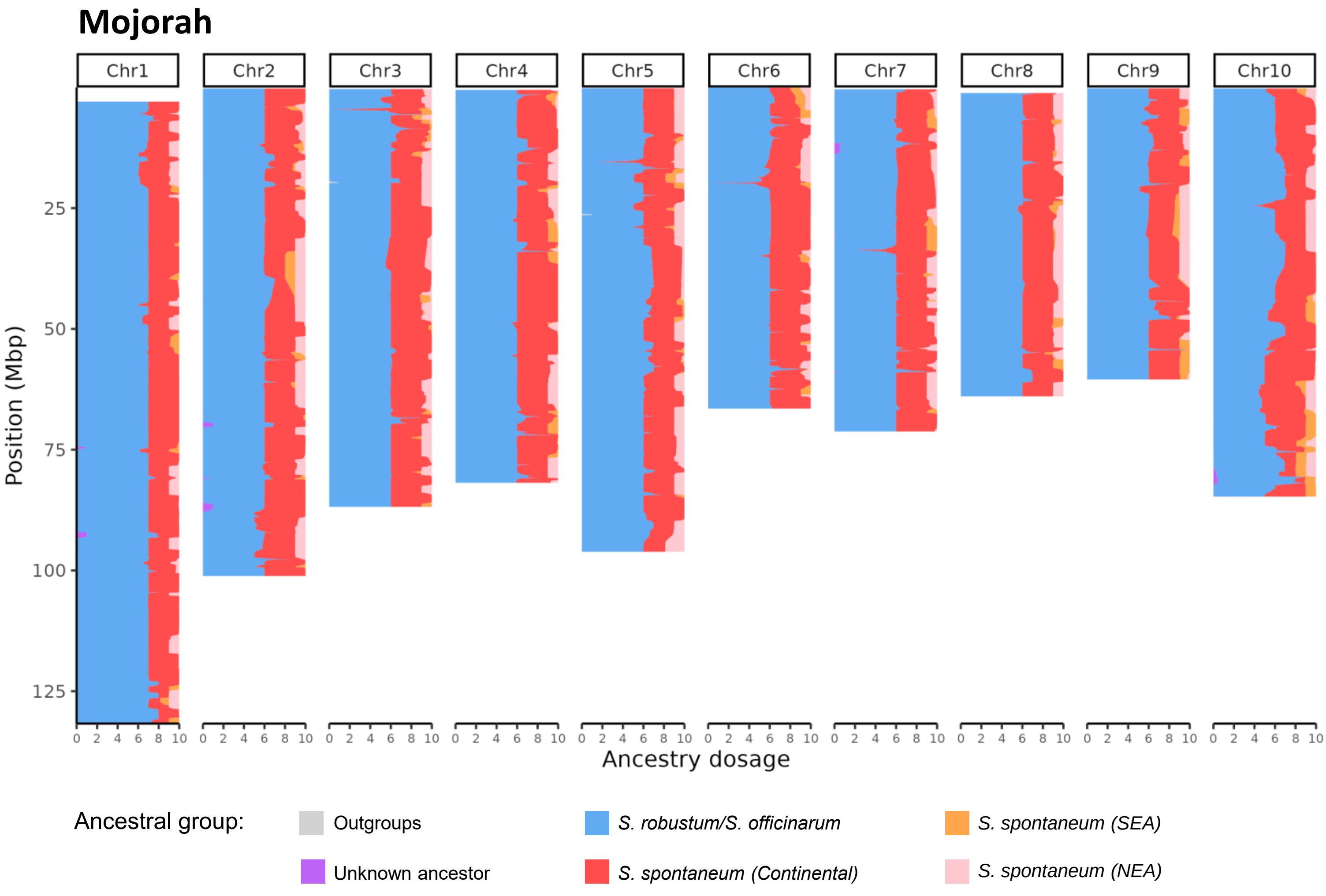

Local admixture of the *S. barberi* Matki Mojorah

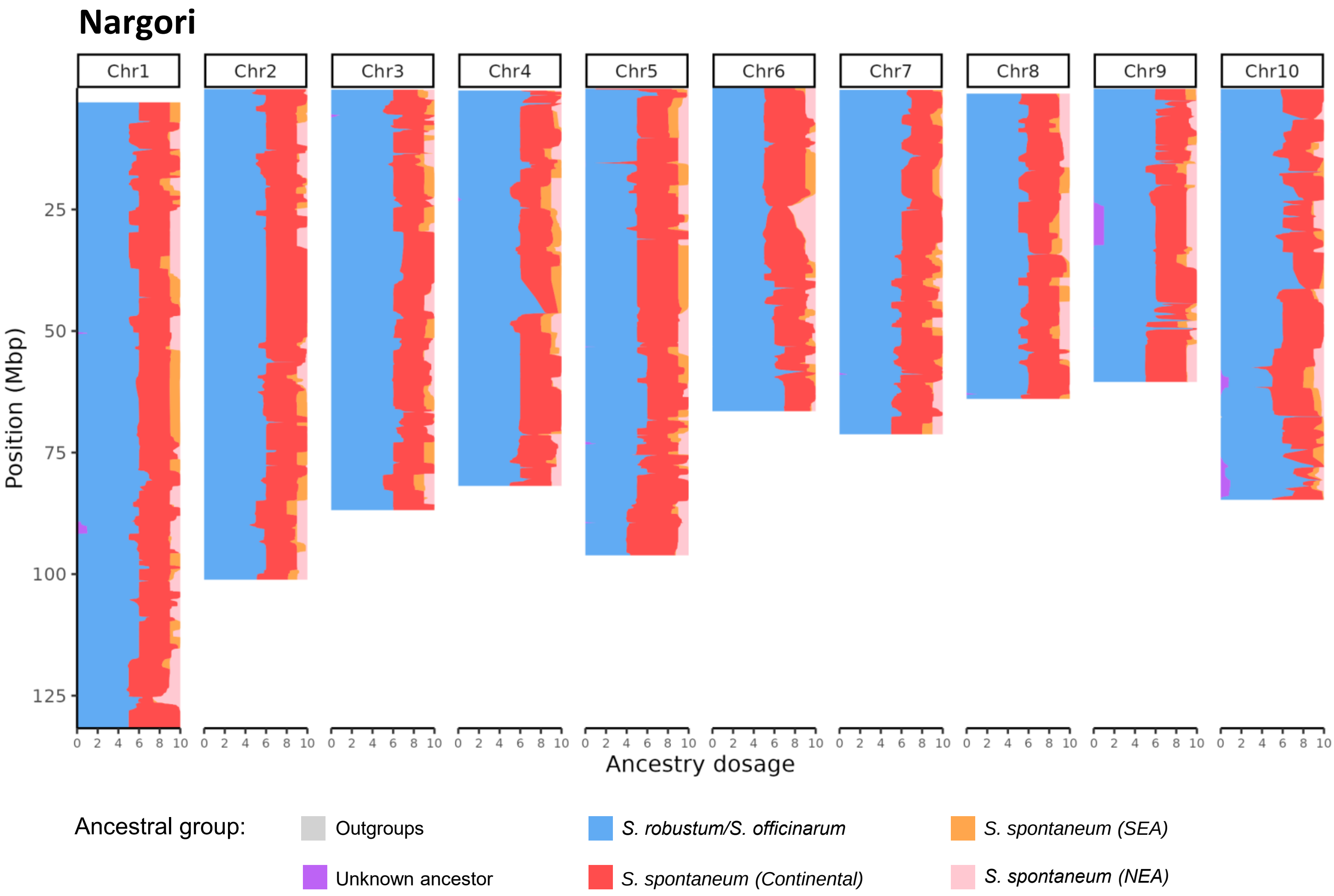

Local admixture of the *S. barberi* Nargori

Patarki mango

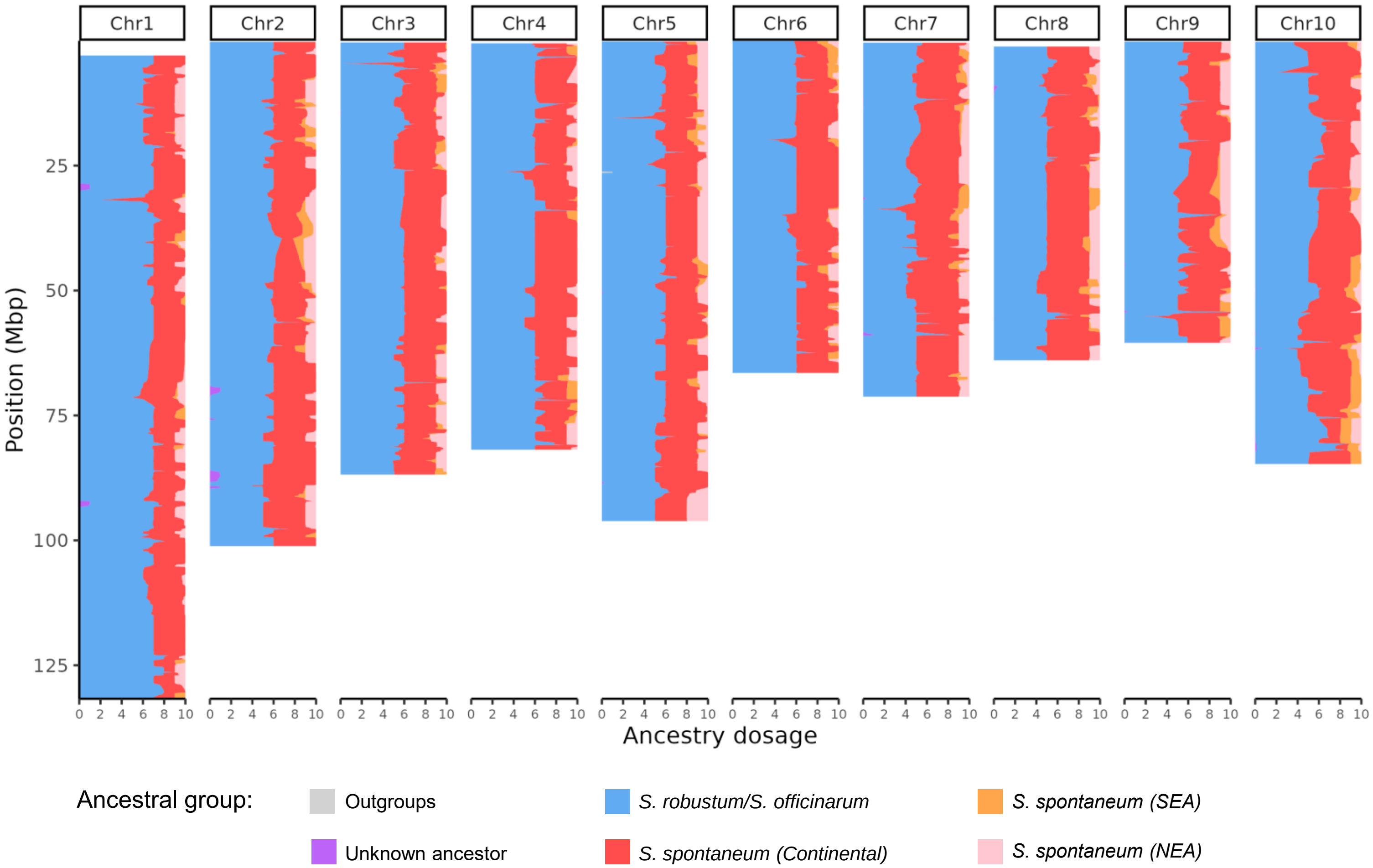

Local admixture of the *S. barberi* Patarki mango

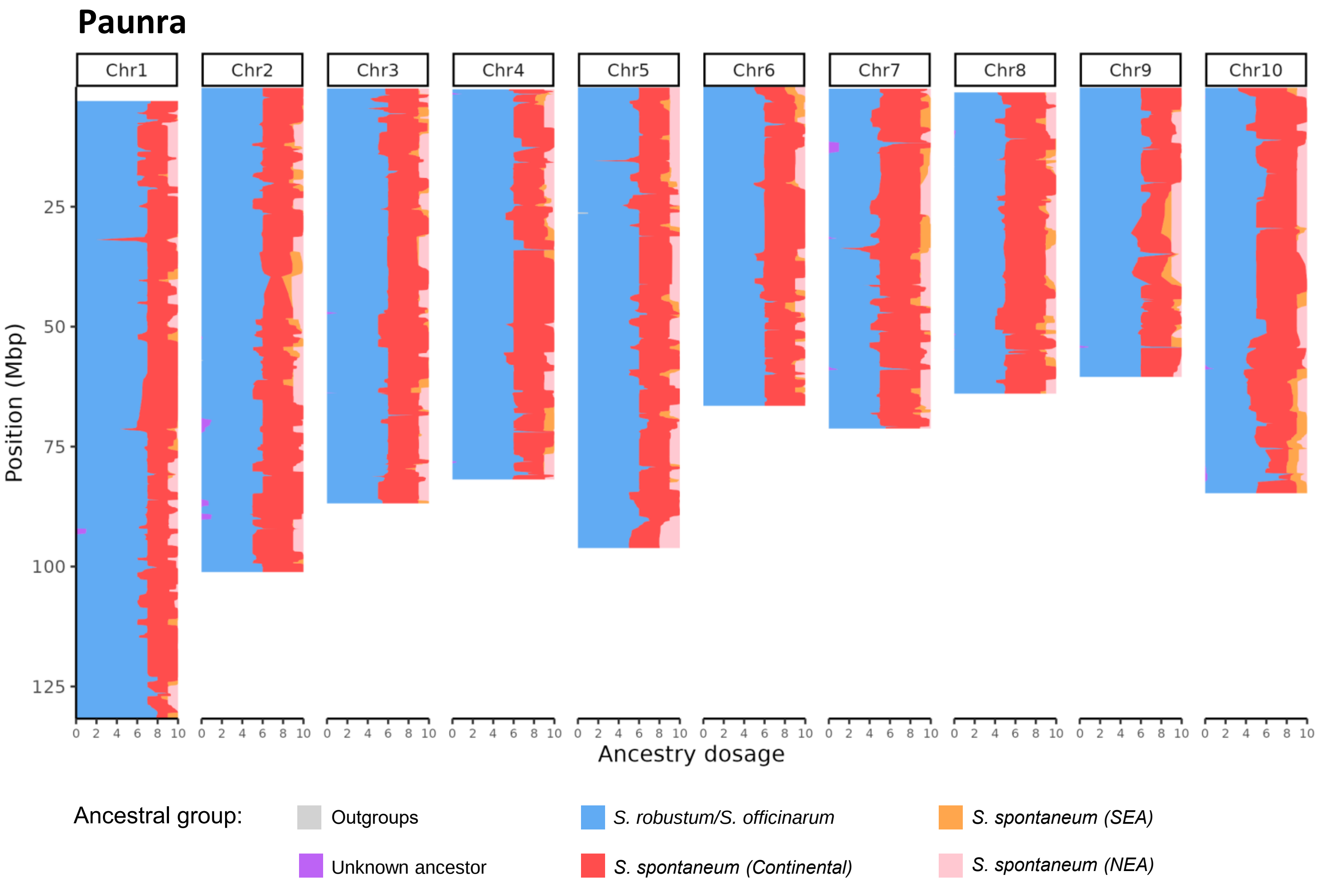

Local admixture of the *S. barberi* Paunra

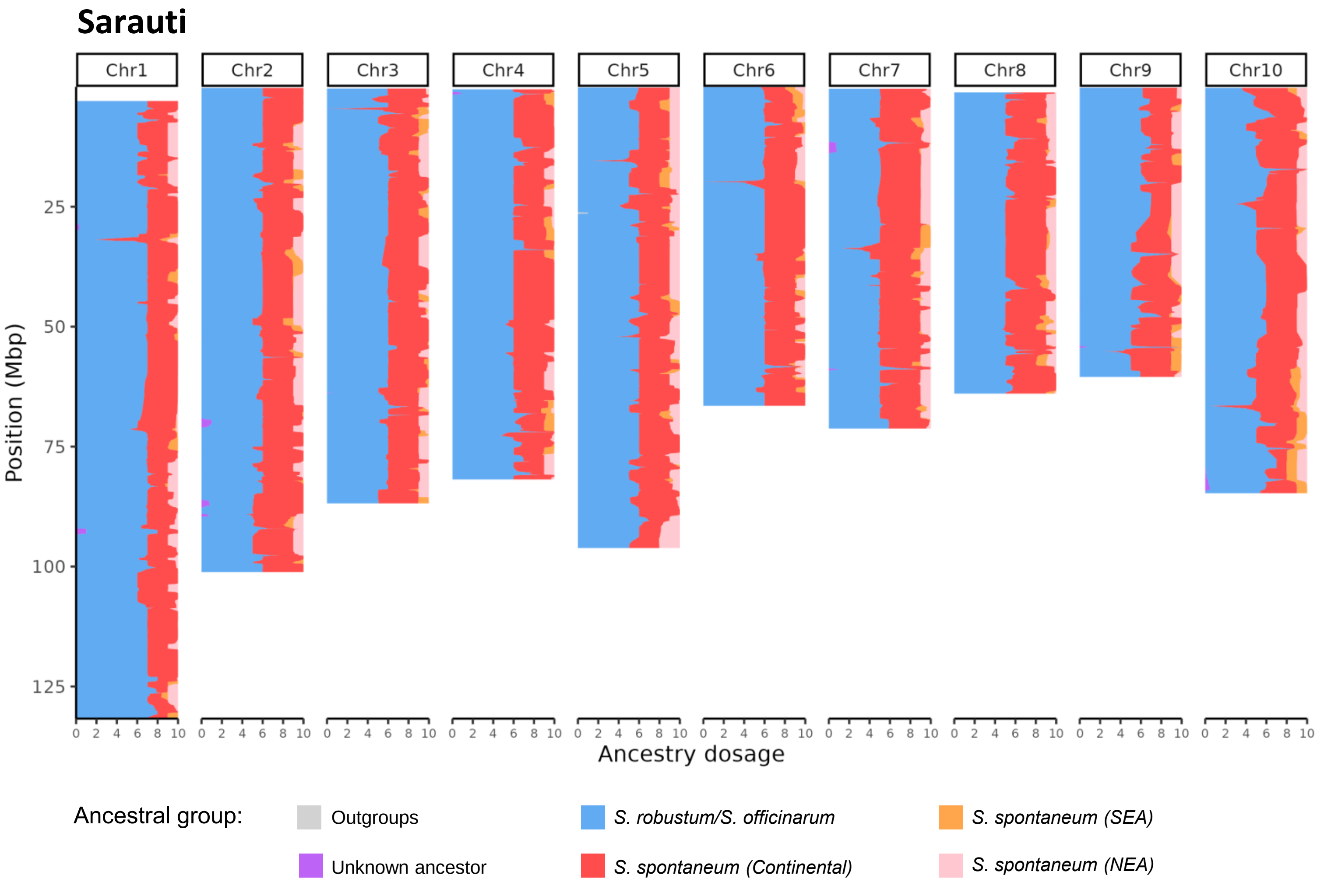

Local admixture of the *S. barberi* Sarauti

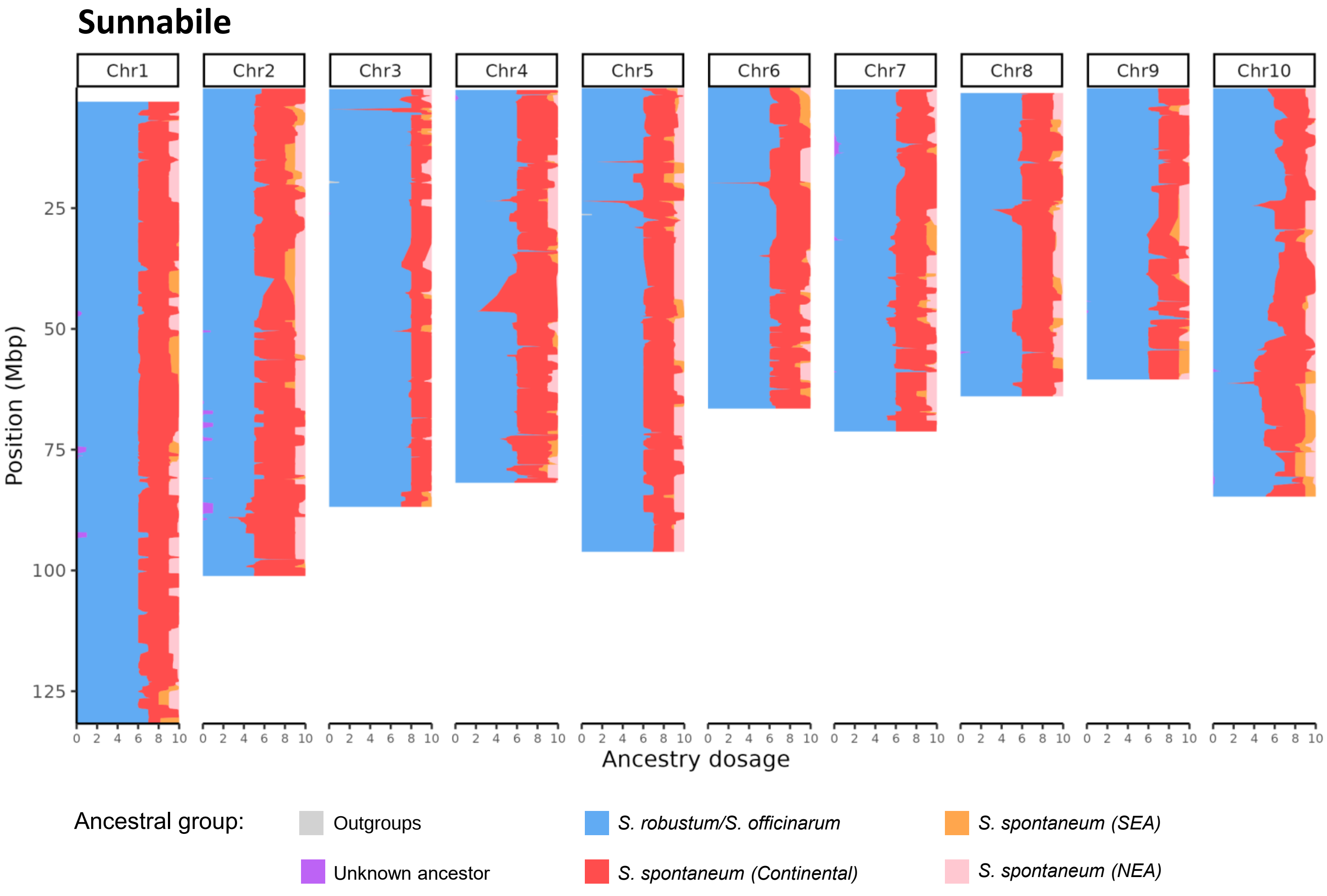

Local admixture of the *S. barberi* Sunnabile

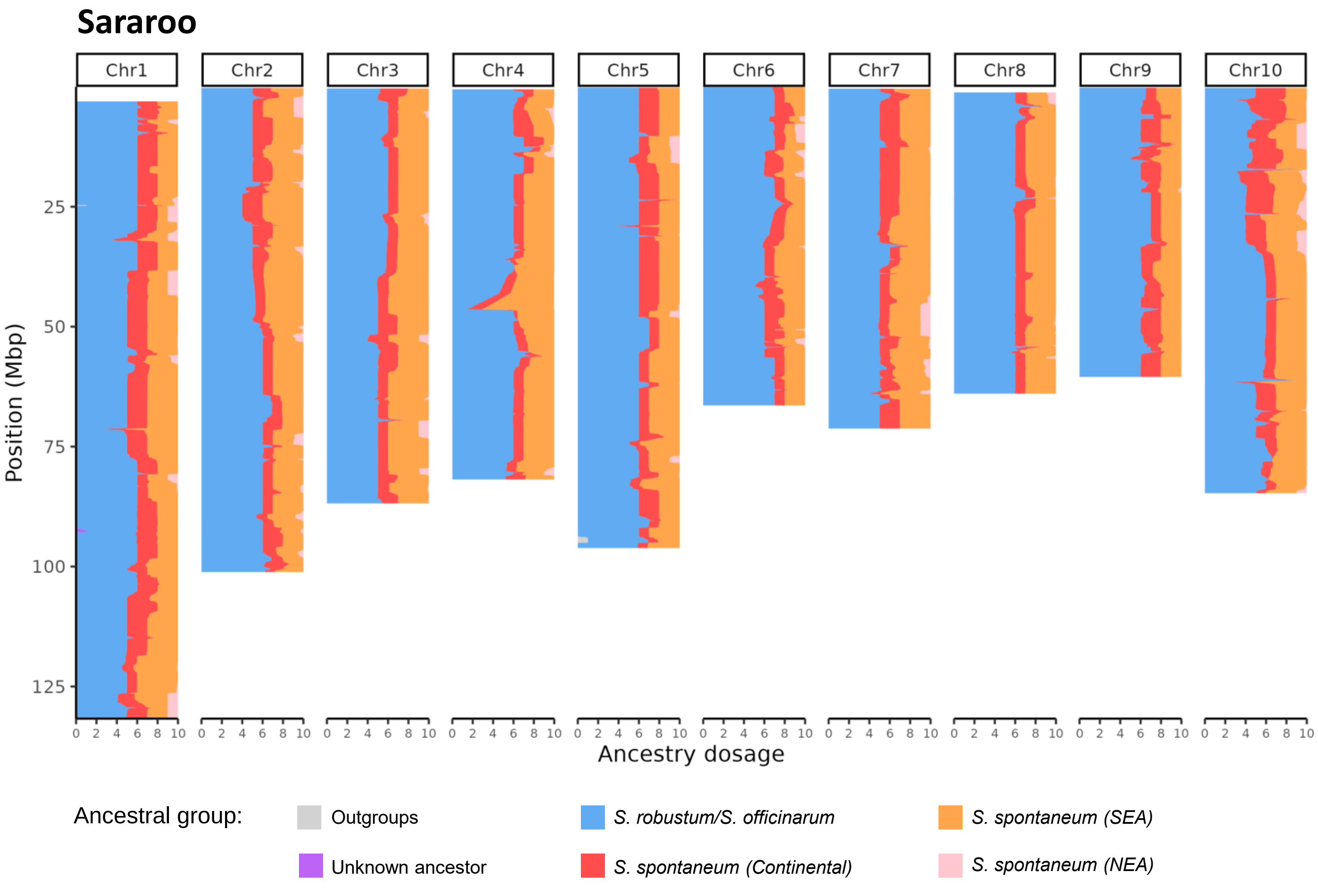

Local admixture of the *S. sinense* Sararoo

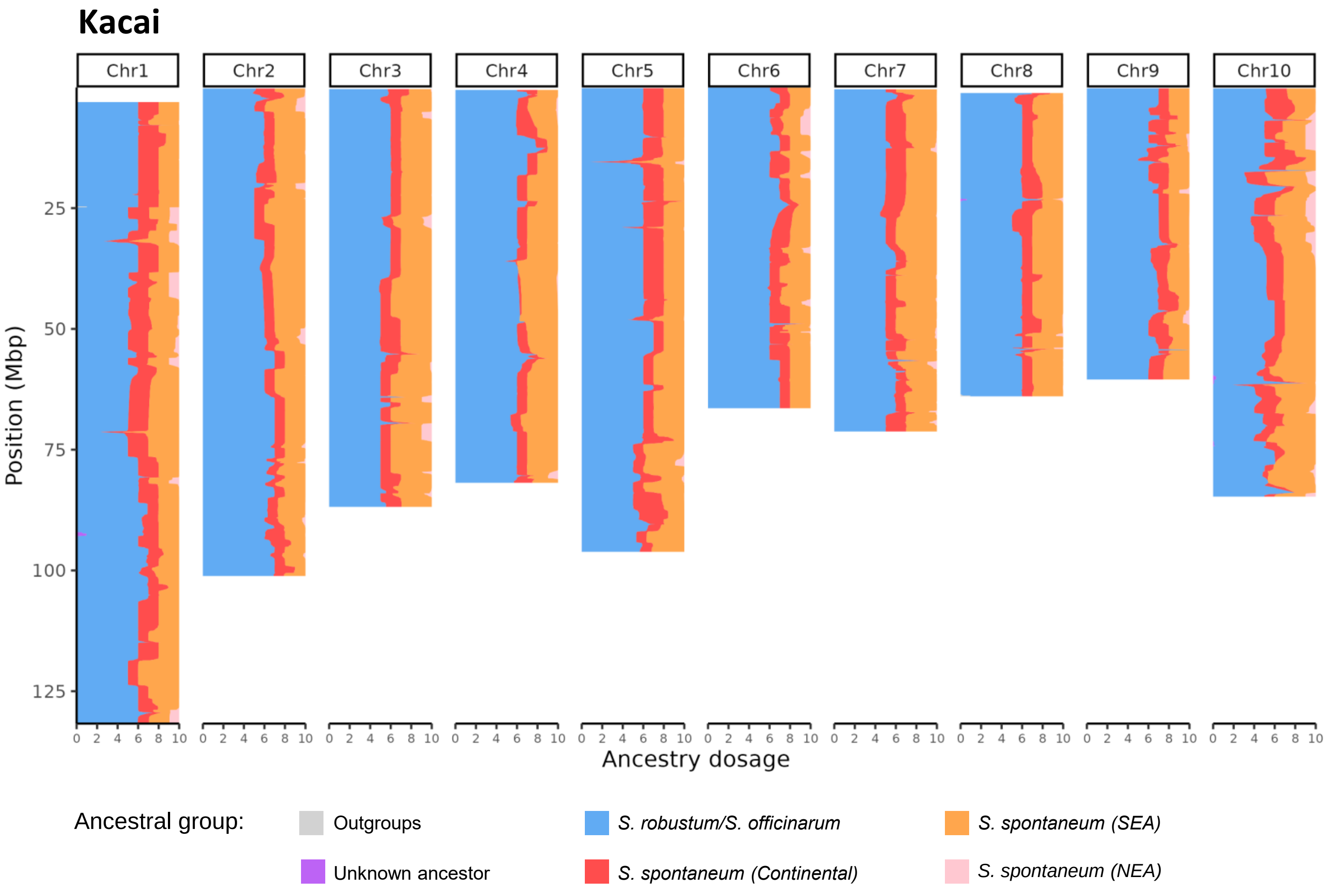

Local admixture of the *S. sinense* Kacai

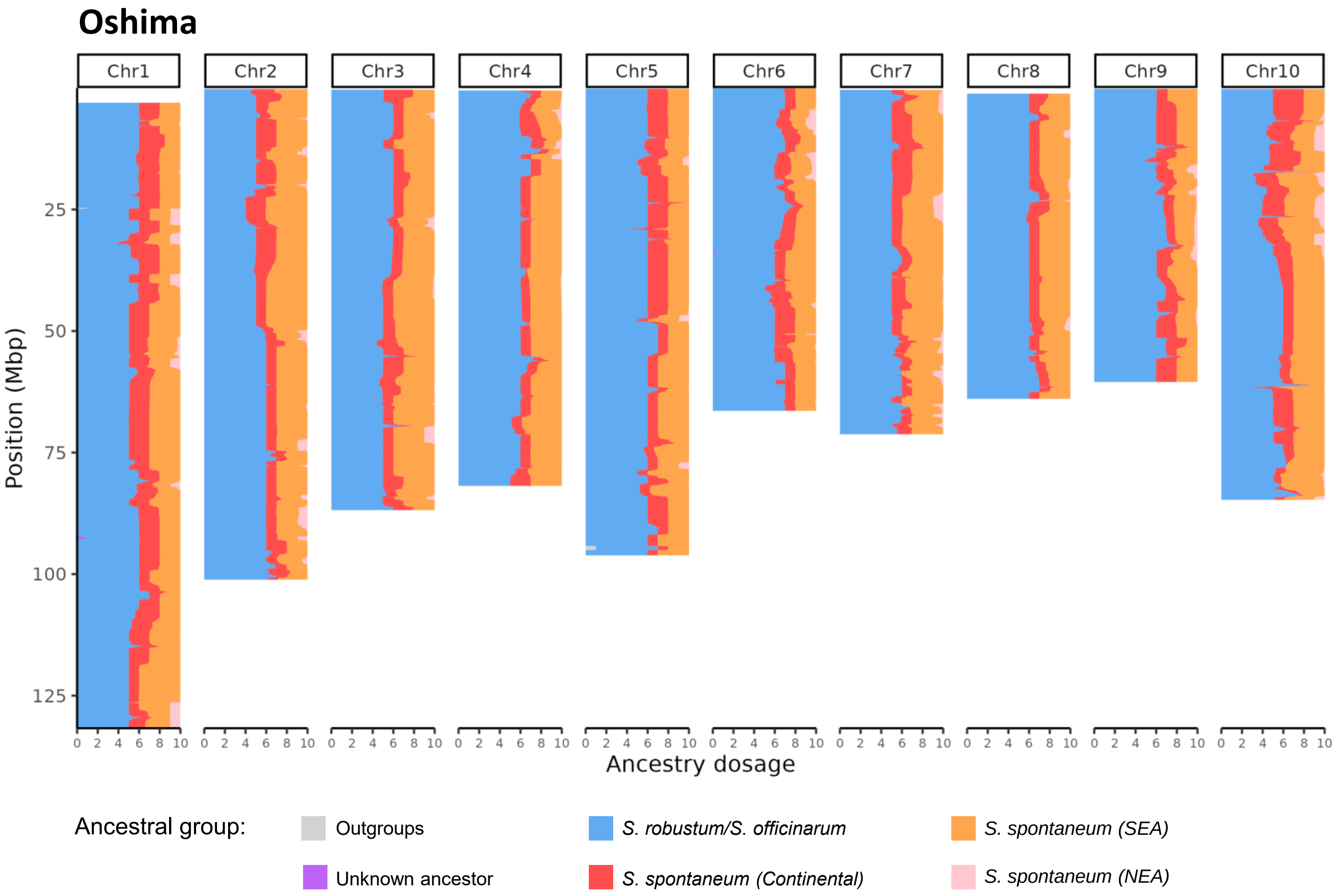

Local admixture of the *S. sinense* Oshima

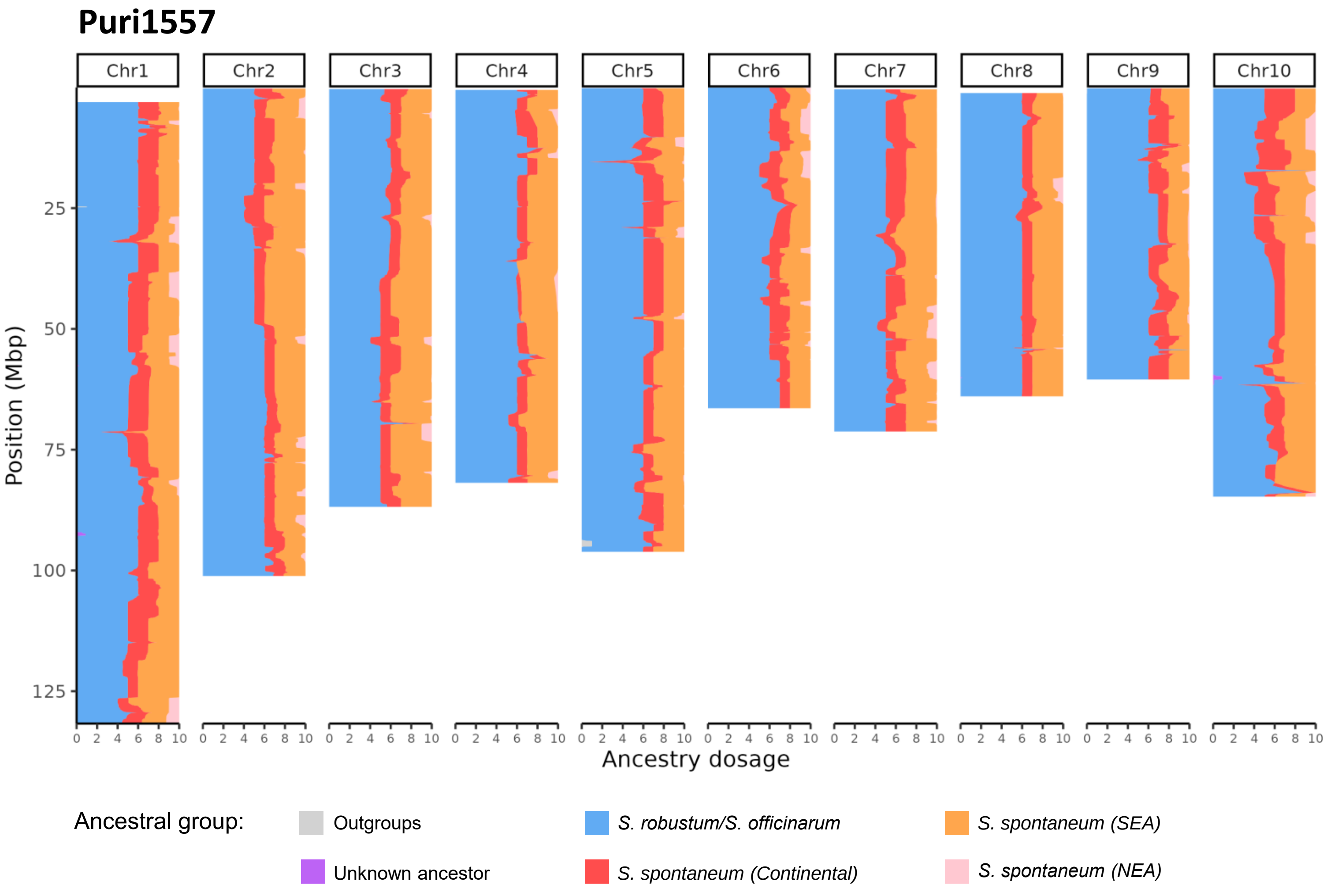

Local admixture of the *S. sinense* Puri1557

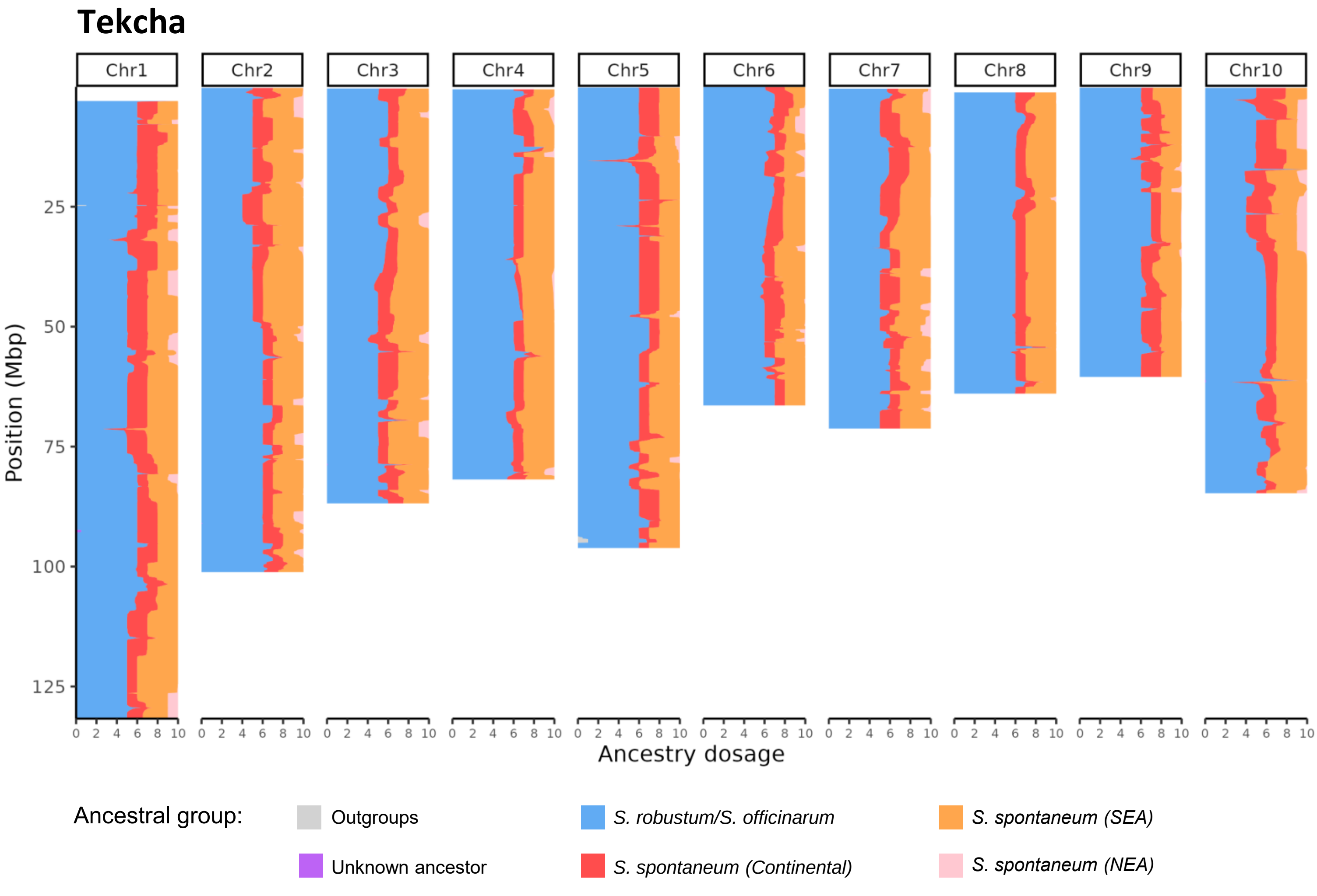

Local admixture of the *S. sinense* Tekcha

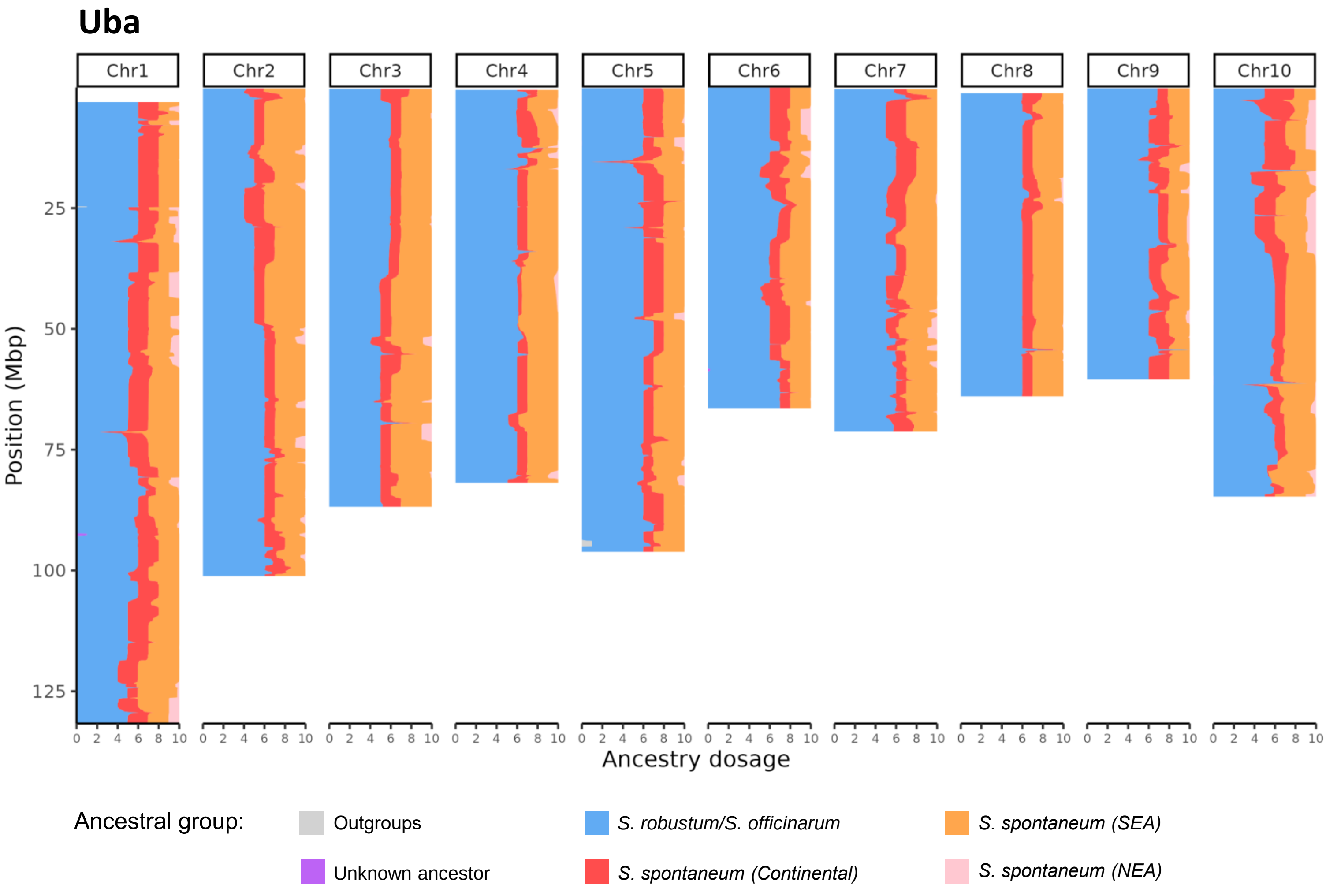

Local admixture of the *S. sinense* Uba
