# Supplementary material for: Deciphering the mosaic genome of sugarcane cultivars through polyploid admixture inference with AdmixPoly
Source: Genome Biol. 2026 Jun 19;27:280. doi: 10.1186/s13059-026-04162-3 (PMC13531838; doi:10.1186/s13059-026-04162-3)
Supplement: Supplementary file 3 [file 13059_2026_4162_MOESM3_ESM.pdf]

Additional file 3: Derivations and Pseudo-codes

Deciphering the Mosaic Genome of Sugarcane Cultivars through Polyploid Admixture Inference with AdmixPoly

Simon Rio[1,2], Franck Gauthier[3], Olivier Garsmeur[1,2], George Piperidis[4], Jean-Yves Hoarau[5], German Serino[6], Raul Castillo Torres[7], Shailesh Vinay Joshi[8,9], Yoshifumi Terajima[10], Jershon Lopez-Gerena[11], María Francisca Perera[12], Andrew Stoute[13], Goolam Badaloo[14], Dongliang Huang[15], Kerrie Barry[16], Jeremy Schmutz[16,17], Tristan Mary-Huard[3,18], and Angélique D'Hont[1,2]

[1]CIRAD, UMR AGAP Institut, F-34398 Montpellier, France
[2]UMR AGAP Institut, Univ Montpellier, CIRAD, INRAE, Institut Agro, F-34398, Montpellier, France
[3]Université Paris-Saclay, INRAE, CNRS, AgroParisTech, UMR GQE-Le Moulon, 91190, Gif-sur-Yvette, France
[4]Sugar Research Australia, Brisbane QLD 4000
[5]eRcane, Sainte-Clotilde, La Réunion, France
[6]Chacra Experimental Agrícola Santa Rosa, Camino Vecinal No. 8 Km 6, Colonia Santa Rosa, 4531 Salta, Argentina
[7]Centro de Investigación de la Caña de Azúcar del Ecuador - CINCAE, Guayaquil, Ecuador
[8]South African Sugarcane Research Institute, Private Bag X02, Mount Edgecombe, Durban, 4300, KwaZulu-Natal, South Africa
[9]School of Agriculture and Science, College of Agriculture, Engineering and Science, University of KwaZulu-Natal, South Africa
[10]Tropical Agriculture Research Front, JIRCAS, 1091-1 Maezato-Kawarabaru, Ishigaki, Okinawa, 907-0002, Japan
[11]Centro de Investigación de la Caña de Azúcar de Colombia (CENICAÑA), Cali, Colombia
[12]Instituto de Tecnología Agroindustrial del Noroeste Argentino (ITANOA), Estación Experimental Agroindustrial Obispo Colombres (EEAOC) - Consejo Nacional de Investigaciones Científicas y Técnicas (CONICET), Las Talitas, Tucumán, Argentina
[13]West Indies Central Sugar Cane Breeding Station, Groves, St. George, Barbados
[14]Mauritius Sugarcane Industry Research Institute, Mauritius Cane Industry Authority, 1 Moka Road, Réduit, Mauritius
[15]Sugarcane Research Institute, Guangxi Academy of Agricultural Sciences, Nanning, China
[16]DOE Joint Genome Institute, Lawrence Berkeley National Laboratory, Berkeley, CA, 94720, USA
[17]HudsonAlpha Institute for Biotechnology, 601 Genome Way Northwest, Huntsville, AL 35806, USA
[18]Université Paris-Saclay, AgroParisTech, INRAE, UMR MIA-Paris, 91120, Palaiseau, France

Section 1: Expectation-maximization algorithm

Conditional expectation of the joint likelihood

The expectation $\mathbb{E}_\theta\left(\mathcal{L}_1|S\right)$ can be expressed as:

$$\mathbb{E}_\theta\left(\mathcal{L}_1|S\right) = \sum_{i=1}^{N}\sum_{m=1}^{M}\sum_{c=1}^{P_i}\sum_{k=1}^{K}\left[\mathbb{E}_\theta\left(A_{imck}|S_{im}=s\right)\log\left(\pi_{ik}\right) + \sum_{l=1}^{L_m}\mathbb{E}_\theta\left(A_{imck}G_{imcl}|S_{im}=s\right)\log\left(\gamma_{mkl}\right)\right]$$

Let us introduce $G_{im} = \{G_{imc}\}$ as the latent phased genotype of individual i at marker m for all hom(e)ologous chromosome copies c and alleles l. The first conditional expectation can be simplified as:

$$\begin{aligned}
\mathbb{E}_\theta\left(A_{imck}|S_{im}=s\right) =& \mathbb{P}\left(A_{imc}=k|S_{im}=s\right)\\
=& \frac{\mathbb{P}\left(A_{imc}=k, S_{im}=s\right)}{\mathbb{P}\left(S_{im}=s\right)}\\
=& \frac{\sum_v \mathbb{P}\left(A_{imc}=k, G_{im}=v, S_{im}=s\right)}{\mathbb{P}\left(S_{im}=s\right)}\\
=& \frac{\sum_v \mathbb{P}\left(A_{imc}=k\right)\mathbb{P}\left(G_{im}=v|A_{imc}=k\right)\mathbb{P}\left(S_{im}=s|G_{im}=v, A_{imc}=k\right)}{\mathbb{P}\left(S_{im}=s\right)}\\
=& \frac{\pi_{ik}\sum_{v\equiv s}\mathbb{P}\left(G_{im}=v|A_{imc}=k\right)}{\mathbb{P}\left(S_{im}=s\right)}\\
=& \frac{\pi_{ik}\sum_{v\equiv s}\prod_{c=1}^{P_i}\mathbb{P}\left(G_{imc}=v_c|A_{imc}=k\right)}{\mathbb{P}\left(S_{im}=s\right)}
\end{aligned}$$

where this last product comes from the independence between chromosome copies

$$\begin{aligned}
=& \frac{\pi_{ik}\sum_{v\equiv s}\mathbb{P}\left(G_{imc}=v_c|A_{imc}=k\right)\prod_{c'\neq c}^{P_i}\mathbb{P}\left(G_{imc'}=v_{c'}\right)}{\mathbb{P}\left(S_{im}=s\right)}\\
=& \frac{\pi_{ik}\sum_{v\equiv s}\sum_{l=1}^{L_m}\mathbb{1}_{\{v_c=l\}}\mathbb{P}\left(G_{imc}=v_c|A_{imc}=k\right)\prod_{c'\neq c}^{P_i}\mathbb{P}\left(G_{imc'}=v_{c'}\right)}{\mathbb{P}\left(S_{im}=s\right)}\\
=& \frac{\sum_{l=1}^{L_m}\pi_{ik}\mathbb{P}\left(G_{imc}=l|A_{imc}=k\right)\sum_{v\equiv s, v_c=l}\prod_{c'\neq c}^{P_i}\mathbb{P}\left(G_{imc'}=v_{c'}\right)}{\mathbb{P}\left(S_{im}=s\right)}\\
=& \sum_{l=1}^{L_m}\frac{\pi_{ik}\mathbb{P}\left(G_{imc}=l|A_{imc}=k\right)}{\mathbb{P}\left(G_{imc}=l\right)}\frac{\sum_{v\equiv s, v_c=l}\mathbb{P}\left(G_{imc}=l\right)\prod_{c'\neq c}^{P_i}\mathbb{P}\left(G_{imc'}=v_{c'}\right)}{\mathbb{P}\left(S_{im}=s\right)}\\
=& \sum_{l=1}^{L_m}\frac{\pi_{ik}\gamma_{mkl}}{\sum_{k'=1}^{K}\pi_{ik'}\gamma_{mk'l}}\frac{\sum_{v\equiv s, v_c=l}\mathbb{P}\left(G_{imc}=l\right)\mathbb{P}\left(G_{im}=v|G_{imc}=l\right)}{\mathbb{P}\left(S_{im}=s\right)}\\
=& \sum_{l=1}^{L_m}\tau_{imkl}\frac{\mathbb{P}\left(G_{imc}=l\right)\mathbb{P}\left(S_{im}=s|G_{imc}=l\right)}{\mathbb{P}\left(S_{im}=s\right)}\\
=& \sum_{l=1}^{L_m}\tau_{imkl}\mathbb{P}\left(G_{imc}=l|S_{im}=s\right)\\
=& \sum_{l=1}^{L_m}\tau_{imkl}\frac{S_{iml}}{P_i}\\
=& \frac{1}{P_i}\sum_{l=1}^{L_m}S_{iml}\tau_{imkl}
\end{aligned}$$

The second conditional expectation can be simplified as:

$$
\begin{aligned}
\mathbb{E}_\theta\left(A_{imck}G_{imcl}|S_{im}=s\right) =& \mathbb{P}\left(A_{imc}=k, G_{imc}=l|S_{im}=s\right)\\
=& \frac{\mathbb{P}\left(A_{imc}=k, G_{imc}=l, S_{im}=s\right)}{\mathbb{P}\left(S_{im}=s\right)}\\
=& \frac{\sum_v \mathbb{P}\left(A_{imc}=k, G_{im}=v, S_{im}=s\right)\mathbb{1}_{\{v_c=l\}}}{\mathbb{P}\left(S_{im}=s\right)}\\
=& \frac{\sum_{v,v_c=l} \mathbb{P}\left(A_{imc}=k, G_{im}=v\right)\mathbb{P}\left(S_{im}=s|A_{imc}=k, G_{im}=v\right)}{\mathbb{P}\left(S_{im}=s\right)}\\
=& \frac{\sum_{v,v_c=l} \mathbb{P}\left(A_{imc}=k, G_{im}=v\right)\mathbb{1}_{\{v\equiv s\}}}{\mathbb{P}\left(S_{im}=s\right)}\\
=& \frac{\sum_{v\equiv s,v_c=l} \mathbb{P}\left(A_{imc}=k|G_{im}=v\right)\mathbb{P}\left(G_{im}=v\right)}{\mathbb{P}\left(S_{im}=s\right)}\\
=& \frac{\mathbb{P}\left(A_{imc}=k|G_{imc}=l\right)\sum_{v\equiv s,v_c=l} \mathbb{P}\left(G_{im}=v\right)}{\mathbb{P}\left(S_{im}=s\right)}\\
=& \frac{\mathbb{P}\left(A_{imc}=k\right)\mathbb{P}\left(G_{imc}=l|A_{imc}=k\right)}{\mathbb{P}\left(G_{imc}=l\right)}\frac{\sum_{v\equiv s,v_c=l} \mathbb{P}\left(G_{im}=v\right)}{\mathbb{P}\left(S_{im}=s\right)}\\
=& \tau_{imk}\frac{\sum_{v\equiv s,v_c=l} \mathbb{P}\left(G_{imc}=l\right)\prod_{c'\neq c}^{P_i}\mathbb{P}\left(G_{imc'}=v_{c'}\right)}{\mathbb{P}\left(S_{im}=s\right)}\\
=& \tau_{imk}\frac{\sum_{v\equiv s,v_c=l} \mathbb{P}\left(G_{imc}=l\right)\mathbb{P}\left(G_{im}=v|G_{imc}=l\right)}{\mathbb{P}\left(S_{im}=s\right)}\\
=& \tau_{imk}\frac{\mathbb{P}\left(G_{imc}=l\right)\mathbb{P}\left(S_{im}=s|G_{imc}=l\right)}{\mathbb{P}\left(S_{im}=s\right)}\\
=& \tau_{imk}\mathbb{P}\left(G_{imc}=l|S_{im}=s\right)\\
=& \tau_{imkl}\frac{S_{iml}}{P_i}\\
=& \frac{1}{P_i}S_{iml}\tau_{imkl}
\end{aligned}
$$

When replacing both conditional expectations using their respective expression, one obtains:

$$
\begin{aligned}
\mathbb{E}_\theta\left(\mathcal{L}_1|S\right) &= \sum_{i=1}^{N}\sum_{m=1}^{M}\sum_{c=1}^{P_i}\sum_{k=1}^{K}\left[\mathbb{E}_\theta\left(A_{imck}|S_{im}=s\right)\log\left(\pi_{ik}\right)+\sum_{l=1}^{L_m}\mathbb{E}_\theta\left(A_{imck}G_{imcl}|S_{im}=s\right)\log\left(\gamma_{mkl}\right)\right]\\
&= \sum_{i=1}^{N}\sum_{m=1}^{M}\sum_{c=1}^{P_i}\sum_{k=1}^{K}\left[\frac{1}{P_i}\sum_{l=1}^{L_m}S_{iml}\tau_{imkl}\log\left(\pi_{ik}\right)+\frac{1}{P_i}\sum_{l=1}^{L_m}S_{iml}\tau_{imkl}\log\left(\gamma_{mkl}\right)\right]\\
&= \sum_{i=1}^{N}\sum_{m=1}^{M}\sum_{c=1}^{P_i}\sum_{k=1}^{K}\frac{1}{P_i}\sum_{l=1}^{L_m}S_{iml}\tau_{imkl}\left[\log\left(\pi_{ik}\right)+\log\left(\gamma_{mkl}\right)\right]\\
&= \sum_{i=1}^{N}\sum_{m=1}^{M}\sum_{l=1}^{L_m}S_{iml}\sum_{k=1}^{K}\tau_{imkl}\left[\log\left(\pi_{ik}\right)+\log\left(\gamma_{mkl}\right)\right]
\end{aligned}
$$

Update of model parameters

Admixture proportions π_{ik} are updated using:

$$\widehat{\pi}_{ik} = \underset{\pi_{ik}}{\operatorname{argmax}}\, \mathbb{E}_\theta\left(\mathcal{L}_1|S\right) \qquad \text{s.t.} \quad \sum_{k=1}^{K} \pi_{ik} = 1$$

Let us introduce the following expression:

$$\mathcal{L}_{\pi_{ik}} = \mathbb{E}_\theta\left(\mathcal{L}_1|S\right) + \lambda\left(1 - \sum_{k=1}^{K} \pi_{ik}\right)$$

Looking at the derivative of $\mathcal{L}_{\pi_{ik}}$ with respect to π_{ik}, one obtains:

$$\frac{\partial \mathcal{L}_{\pi_{ik}}}{\partial \pi_{ik}} = \sum_{m=1}^{M} \sum_{l=1}^{L_m} \frac{S_{iml}\tau_{imkl}}{\pi_{ik}} - \lambda$$

Setting the derivative to 0 and noticing that $\lambda = MP_i$, the following estimator is obtained:

$$\hat{\pi}_{ik} = \frac{1}{MP_i} \sum_{m=1}^{M} \sum_{l=1}^{L_m} S_{iml}\tau_{imkl}$$

Similarly, for group-specific allele frequencies γ_{mkl}:

$$\widehat{\gamma}_{mkl} = \underset{\gamma_{mkl}}{\operatorname{argmax}}\, \mathbb{E}_\theta\left(\mathcal{L}_1|S\right) \qquad \text{s.t.} \quad \sum_{l=1}^{L_m} \gamma_{mkl} = 1$$

Let us introduce the following expression:

$$\mathcal{L}_{\gamma_{mkl}} = \mathbb{E}_\theta\left(\mathcal{L}_1|S\right) + \lambda\left(1 - \sum_{l=1}^{L_m} \gamma_{mkl}\right)$$

Looking at the derivative of $\mathcal{L}_{\gamma_{mkl}}$ with respect to γ_{mkl}, one obtains:

$$\frac{\partial \mathcal{L}_{\gamma_{mkl}}}{\partial \gamma_{mkl}} = \sum_{i=1}^{N} \frac{S_{iml}\tau_{imkl}}{\gamma_{mkl}} - \lambda$$

Setting the derivative to 0 and noticing that $\lambda = \sum_{i=1}^{N} \sum_{l=1}^{L_m} S_{iml}\tau_{imkl}$, the following estimator is obtained:

$$\hat{\gamma}_{mkl} = \frac{\sum_{i=1}^{N} S_{iml}\tau_{imkl}}{\sum_{i=1}^{N} \sum_{l'=1}^{L_m} S_{iml'}\tau_{imkl'}}$$

Pseudo-code

In practice, the following algorithm is applied to update model parameters:

Algorithm 1 EM update of model parameters

Require: $S_{im} = \{S_{iml}\}$: allele dosages
Require: $\pi_{ik}(s)$: admixture proportions from previous EM step s
Require: $\gamma_{mkl}(s)$: ancestral allele frequencies from previous EM step s
Ensure: $\pi_{ik}(s+1)$: updated admixture proportions
Ensure: $\gamma_{mkl}(s+1)$: updated ancestral allele frequencies

$\pi_{ik}(s+1) \leftarrow 0 \ \forall\, i, k$ ▷ initialize admixture proportions
$\gamma_{mkl}(s+1) \leftarrow 0 \ \forall\, m, k, l$ ▷ initialize ancestral allele frequencies
for $m \leftarrow 1$ to M **do** ▷ for each marker
  $\tau_{ikl}(m) \leftarrow 0 \ \forall\, i, k, l$ ▷ initialize posterior for marker m
  for $i \leftarrow 1$ to N **do** ▷ for each individual
    for $k \leftarrow 1$ to K **do** ▷ for each ancestral group
      for $l \leftarrow 1$ to L_m **do** ▷ for each allele of marker m
        $\tau_{ikl}(m) \leftarrow \frac{\pi_{ik}(s)\gamma_{mkl}(s)}{\sum_{k'} \pi_{ik'}(s)\gamma_{mk'l}(s)}$ ▷ calculate posterior for marker m
      end for
      $\pi_{ik}(s+1) \leftarrow \pi_{ik}(s+1) + \sum_l S_{iml}\tau_{ikl}(m)$ ▷ update admixture proportion
    end for
  end for
  for $l \leftarrow 1$ to L_m **do** ▷ for each allele of marker m
    for $k \leftarrow 1$ to K **do** ▷ for each ancestral group
      $\gamma_{mkl}(s+1) \leftarrow \frac{\sum_i S_{iml}\tau_{ikl}(m)}{\sum_i \sum_{l'} S_{iml'}\tau_{ikl'}(m)}$ ▷ update ancestral allele frequency
    end for
  end for
end for
$\pi_{ik}(s+1) \leftarrow \frac{\pi_{ik}(s+1)}{MP_i}$ ▷ standardize admixture proportions

Section 2: Transition probabilities

Expression involving transition probabilities at the haplotype level

Let us define $Z_{im} = \{Z_{imc}\}$ as the latent phased ancestry state of individual i at marker m over chromosome copies c. Supposing a tetraploid state $u = \{1, 3\}$, the set of phased haplotype ancestries is: $\{\{1, 2, 2, 2\}, \{2, 1, 2, 2\}, \{2, 2, 1, 2\}, \{2, 2, 2, 1\}\}$. The transition probability $\Omega_{imuu'}$ can be calculated as:

$$
\begin{aligned}
\Omega_{imuu'} &= \mathbb{P}\left(A_{im} = u | A_{im-1} = u'\right) \\
&= \sum_{j \equiv u} \sum_{j' \equiv u'} \mathbb{P}\left(A_{im} = u, Z_{im} = j, Z_{im-1} = j' | A_{im-1} = u'\right) \\
&= \sum_{j \equiv u} \sum_{j' \equiv u'} \mathbb{P}\left(A_{im} = u | A_{im-1} = u', Z_{im} = j, Z_{im-1} = j'\right) \\
&\qquad \times \mathbb{P}\left(Z_{im} = j | A_{im-1} = u', Z_{im-1} = j'\right) \mathbb{P}\left(Z_{im-1} = j' | A_{im-1} = u'\right) \\
&= \frac{1}{J_{u'}} \sum_{j \equiv u} \sum_{j' \equiv u'} \mathbb{P}\left(Z_{im} = j | Z_{im-1} = j'\right) \\
&= \frac{1}{J_{u'}} \sum_{j \equiv u} \sum_{j' \equiv u'} \prod_{c=1}^{P_i} \mathbb{P}\left(Z_{imc} = j_c | Z_{im-1c} = j'_c\right)
\end{aligned}
$$

where this last product comes from the independence between chromosome copies

$$
\begin{aligned}
&= \frac{1}{J_{u'}} \sum_{j \equiv u} \sum_{j' \equiv u'} \prod_{c=1}^{P_i} \sum_{k=1}^{K} \sum_{k'=1}^{K} \mathbb{P}\left(Z_{imc} = j_c | Z_{im-1c} = j'_c\right) \mathbb{1}_{\{j_c = k, j'_c = k'\}} \\
&= \frac{1}{J_{u'}} \sum_{j \equiv u} \sum_{j' \equiv u'} \prod_{c=1}^{P_i} \sum_{k=1}^{K} \sum_{k'=1}^{K} \mathbb{P}\left(Z_{imc} = k | Z_{im-1c} = k'\right) \mathbb{1}_{\{j_c = k, j'_c = k'\}} \\
&= \frac{1}{J_{u'}} \sum_{j \equiv u} \sum_{j' \equiv u'} \prod_{c=1}^{P_i} \sum_{k=1}^{K} \sum_{k'=1}^{K} \Omega_{imkk'} \mathbb{1}_{\{j_c = k, j'_c = k'\}} \\
&= \frac{1}{J_{u'}} \sum_{j \equiv u} \sum_{j' \equiv u'} \prod_{k=1}^{K} \prod_{k'=1}^{K} \left(\Omega_{imkk'}\right)^{n_{kk'}^{jj'}}
\end{aligned}
$$

In practice, the following algorithm is applied to calculate transition probabilities:

Algorithm 2 Calculation of transition probabilities

Require: Ω_{im}: transition probability matrix defined at the haplotype level
Require: P_i: ploidy level
Ensure: $\Omega_{im}^{P_i}$: transition probability matrix at ploidy level P_i

```
1: Ω¹_im ← Ω_im                                          ▷ initialize with Ω_im
2: for p ← 2 to P_i do
3:     Ω^{p*}_im ← Ω_im ⊗ Ω^{p−1}_im                      ▷ with size K·binom(p−1+K−1, p−1)
4:     Ω^p_im ← ReduceToAncestryDosages(Ω^{p*}_im)        ▷ with size binom(p+K−1, p)
5: end for
6: return Ω^{P_i}_im
```

ReduceToAncestryDosages(): The Kronecker product has expanded the transition probability matrix Ω_{im}^{p-1} assuming a new recombination for each ancestral group. Some of the resulting

states are equivalent and correspond to a same ancestry dosage state u. Rows and columns of the matrix corresponding to these equivalent states are summed, and each row is standardized so that the row sum is equal to 1

Conditioning on the number of recombinations

Alternatively, let us the define R_{im} as the number of recombinations between markers $m-1$ and m and X_{imr} as the set of positions pointing to the r recombining copies. The transition probability $\Omega_{imuu'}$ can be calculated as:

$$\begin{aligned}
\Omega_{imuu'} &= \frac{1}{J_{u'}} \sum_{j \equiv u} \sum_{j' \equiv u'} \mathbb{P}\left(Z_{im} = j | Z_{im-1} = j'\right) \\
&= \frac{1}{J_{u'}} \sum_{r=0}^{P_i} \sum_{j \equiv u} \sum_{j' \equiv u'} \mathbb{P}\left(Z_{im} = j, R_{im} = r | Z_{im-1} = j'\right) \\
&= \frac{1}{J_{u'}} \sum_{r=0}^{P_i} \sum_{j \equiv u} \sum_{j' \equiv u'} \mathbb{P}\left(Z_{im} = j | Z_{im-1} = j', R_{im} = r\right) \mathbb{P}\left(R_{im} = r | Z_{im-1} = j'\right) \\
&= \sum_{r=0}^{P_i} \mathbb{P}\left(R_{im} = r\right) \frac{1}{J_{u'}} \sum_{j \equiv u} \sum_{j' \equiv u'} \mathbb{P}\left(Z_{im} = j | Z_{im-1} = j', R_{im} = r\right) \\
&= \sum_{r=0}^{P_i} \mathbb{P}\left(R_{im} = r\right) \frac{1}{J_{u'}} \sum_{j \equiv u} \sum_{j' \equiv u'} \sum_{s \equiv r, j, j'} \mathbb{P}\left(Z_{im} = j, X_{imr} = s | Z_{im-1} = j', R_{im} = r\right) \\
&= \sum_{r=0}^{P_i} \mathbb{P}\left(R_{im} = r\right) \frac{1}{J_{u'}} \sum_{j \equiv u} \sum_{j' \equiv u'} \sum_{s \equiv r, j, j'} \mathbb{P}\left(Z_{im} = j | Z_{im-1} = j', R_{im} = r, X_{imr} = s\right) \\
&\qquad \times \mathbb{P}\left(X_{imr} = s | Z_{im-1} = j', R_{im} = r\right)
\end{aligned}$$

since the recombination positions X_{imr} are independent from the copy states Z_{im-1}, and all equiprobable

$$\begin{aligned}
&= \sum_{r=0}^{P_i} \mathbb{P}\left(R_{im} = r\right) \frac{1}{J_{u'} \binom{P_i}{r}} \sum_{j \equiv u} \sum_{j' \equiv u'} \sum_{s \equiv r, j, j'} \mathbb{P}\left(Z_{im} = j | Z_{im-1} = j', X_{imr} = s\right) \\
&= \sum_{r=0}^{P_i} \mathbb{P}\left(R_{im} = r\right) \frac{1}{J_{u'} \binom{P_i}{r}} \sum_{j \equiv u} \sum_{j' \equiv u'} \sum_{s \equiv r, j, j'} \prod_{c \in s} \mathbb{P}\left(Z_{imc} = j_c\right)
\end{aligned}$$

this last simplification relying on the observation that new ancestries must be drawn only for copies affected by an ancestry switch

$$\begin{aligned}
&= \sum_{r=0}^{P_i} \mathbb{P}\left(R_{im} = r\right) \frac{1}{J_{u'} \binom{P_i}{r}} \sum_{j \equiv u} \sum_{j' \equiv u'} \sum_{s \equiv r, j, j'} \prod_{c \in s} \sum_{k=1}^{K} \mathbb{P}\left(Z_{imc} = k\right) \mathbb{1}_{\{j_c = k\}} \\
&= \sum_{r=0}^{P_i} \mathbb{P}\left(R_{im} = r\right) \frac{1}{J_{u'} \binom{P_i}{r}} \sum_{j \equiv u} \sum_{j' \equiv u'} \sum_{s \equiv r, j, j'} \prod_{c \in s} \sum_{k=1}^{K} \pi_{ik} \mathbb{1}_{\{j_c = k\}} \\
&= \sum_{r=0}^{P_i} \underbrace{\mathbb{P}\left(R_{im} = r\right)} \underbrace{\frac{1}{J_{u'} \binom{P_i}{r}} \sum_{j \equiv u} \sum_{j' \equiv u'} \sum_{s \equiv r, j, j'} \prod_{k=1}^{K} \left(\pi_{ik}\right)^{n_{sk}^{jj'}}} \\
&= \sum_{r=0}^{P_i} \quad C_{irm} \quad \times \quad M_{uu'}^{ir}
\end{aligned}$$

In practice, the following iterative algorithm is applied to calculate the general transition term $M^{ir}_{uu'}$ associated with r recombinations for an individual i with ploidy P_i and K ancestral groups:

Algorithm 3 Calculation of general transition terms $M^{ir}_{uu'}$ between states u' and u

```
Require: $\pi_i$: vector of genome-wide admixture proportions
Require: $P_i$: ploidy level
Require: $\mathcal{U}_i$: set of ancestry dosage states
Ensure: $M^{ir}_{uu'}$: general transition term
$M^{ir}_{uu'} \leftarrow 0 \;\forall\, u, u', r$                ▷ initialize general transition term
for $u' \in \mathcal{U}_i$ do                                  ▷ for each initial state
    $M^{i0}_{u'u'} \leftarrow 1$                               ▷ set general transition term for $r = 0$
    $q' \leftarrow u'$                                          ▷ initialize vector of available copies for recombination
    $p' \leftarrow 1$                                           ▷ initialize probability to 1
    $\mathcal{L} \leftarrow \text{Map}\langle\text{Tuple}, \text{Count}\rangle$   ▷ declare map associating a tuple to a count
    $\mathcal{L}[(u', q', p')] \leftarrow 1$                    ▷ fill map with first input tuple
    for $r \leftarrow 1$ to $P_i$ do
        $\mathcal{L} \leftarrow \text{MakeRecomb}(\mathcal{L})$  ▷ make one recombination
        for $(u, q, p), n \in \mathcal{L}$ do                  ▷ for each tuple - count pair
            $M^{ir}_{uu'} \leftarrow M^{ir}_{uu'} + p \times n$  ▷ add probability
        end for
    end for
end for
function MakeRecomb($\mathcal{L}_{\text{in}}$)                  ▷ apply one recombination
    $\mathcal{L}_{\text{out}} \leftarrow \text{Map}\langle\text{Tuple}, \text{Count}\rangle$   ▷ declare output map associating a tuple to a count
    for $(u_0, q_0, p_0), n_0 \in \mathcal{L}_{\text{in}}$ do   ▷ for each input tuple - count pair
        $Q \leftarrow \sum_k q_0[k]$                            ▷ total number of available recombinations
        for $k \leftarrow 1$ to $K$ do                          ▷ for each ancestral group
            if $q_0[k] > 0$ then                                ▷ test if any available copy for ancestral group $k$
                for $k' \leftarrow 1$ to $K$ do                 ▷ for each ancestral group
                    $q_1 \leftarrow q_0$                        ▷ initialize available copies
                    $u_1 \leftarrow u_0$                        ▷ initialize output state
                    $q_1[k] \leftarrow q_1[k] - 1$              ▷ decrease one available copy for group $k$
                    $u_1[k] \leftarrow u_1[k] - 1$              ▷ decrease one copy for group $k$
                    $u_1[k'] \leftarrow u_1[k'] + 1$            ▷ increase one copy for group $k'$
                    $p_1 \leftarrow q_0[k] \times \pi_i[k'] \times p_0 \times Q^{-1}$   ▷ calculate probability
                    if $(u_1, q_1, p_1) \in \mathcal{L}_{\text{out}}$ then   ▷ test if tuple already exists in the map
                        $\mathcal{L}_{\text{out}}[(u_1, q_1, p_1)] \leftarrow \mathcal{L}_{\text{out}}[(u_1, q_1, p_1)] + n_0$   ▷ increment count
                    else
                        $\mathcal{L}_{\text{out}}[(u_1, q_1, p_1)] \leftarrow n_0$   ▷ set count to 1 for the tuple
                    end if
                end for
            end if
        end for
    end for
    return $\mathcal{L}_{\text{out}}$                           ▷ return output map
end function
```

Section 3: Emission probabilities

Expression of the emission probability

Let us the define $W_{im} = \{W_{imkl}\}$ as the latent dosage of alleles according to their ancestral origin. These states can be represented as allele count matrices of dimension $K \times L_m$ whose rows sum to $\sum_l W_{imkl} = A_{imk}$ and columns sum to $\sum_k W_{imkl} = S_{iml}$. The emission probability ψ_{imus} can be calculated as:

$$
\begin{aligned}
\psi_{imus} &= \mathbb{P}\left(S_{im} = s | A_{im} = u\right) \\
&= \sum_{w \equiv u,s} \mathbb{P}\left(S_{im} = s, W_{im} = w | A_{im} = u\right) \\
&= \sum_{w \equiv u,s} \mathbb{P}\left(W_{im} = w | A_{im} = u\right) \mathbb{P}\left(S_{im} = s | W_{im} = w, A_{im} = u\right) \\
&= \sum_{w \equiv u,s} \mathbb{P}\left(W_{im} = w | A_{im} = u\right) \\
&= \sum_{w \equiv u,s} \prod_{k=1}^{K} \mathbb{P}\left(W_{imk} = w_k | A_{imk} = u_k\right) \\
&= \sum_{w \equiv u,s} \prod_{k=1}^{K} \binom{u_k}{w_{k1}, \ldots, w_{kL_m}} \prod_{l=1}^{L_m} \gamma_{mkl}^{w_{kl}}
\end{aligned}
$$

or equivalently assuming $G_{im} = \{G_{imc}\}$ as the phased genotype and $Z_{im} = \{Z_{imc}\}$ as the phased ancestry state of individual i at marker m over chromosomes copies:

$$
\begin{aligned}
\psi_{imus} &= \mathbb{P}\left(S_{im} = s | A_{im} = u\right) \\
&= \mathbb{P}\left(S_{im} = s | Z_{im} = j\right) \text{ for any } j \equiv u \\
&\text{(since the specific ancestry phasing has no impact on the cumulative allele counts)} \\
&= \sum_{v \equiv s} \mathbb{P}\left(G_{im} = v | Z_{im} = j\right) \\
&= \sum_{v \equiv s} \prod_{c=1}^{P_i} \mathbb{P}\left(G_{imc} = v_c | Z_{imc} = j_c\right) \\
&= \sum_{v \equiv s} \prod_{c=1}^{P_i} \sum_{l=1}^{L_m} \sum_{k=1}^{K} \mathbb{P}\left(G_{imc} = l | Z_{imc} = k\right) \mathbb{1}_{\{j_c = k, v_c = l\}} \\
&= \sum_{v \equiv s} \prod_{c=1}^{P_i} \sum_{l=1}^{L_m} \sum_{k=1}^{K} \gamma_{mkl} \mathbb{1}_{\{j_c = k, v_c = l\}} \\
&= \sum_{v \equiv s} \sum_{l=1}^{L_m} \sum_{k=1}^{K} {\gamma_{mkl}}^{n_{kl}^{1v}}
\end{aligned}
$$

choosing $j = 1$ arbitrarily in the last expression and denoting n_{kl}^{1v} the number of chromosome copies with allele l from ancestry k in phased ancestry state 1 and genotype v.

While the first formula may look more intuitive, the calculation of this probability in the R-package is based on an efficient implementation of the second formula.

Pseudo-code

In practice, the following algorithm is applied to calculate emission probabilities:

Algorithm 4 Calculation of emission probabilities

Require: S_{im}: allele dosages
Require: A_{im}: ancestry dosages
Require: j: any phased ancestry state compatible with A_{im}
Require: γ_{mkl}: ancestral allele frequency
Ensure: ψ_{imus}: emission probability

$\psi_{imus} \leftarrow 0$ ▷ initialize emission probability
for $v \equiv S_{im}$ **do** ▷ for each phased genotype v compatible with S_{im}
    $p \leftarrow 1$ ▷ initialize probability
    for $c \leftarrow 1$ to P_i **do** ▷ for each chromosome copy
        $p \leftarrow p \times \gamma_{mkl}[j_c, v_c]$ ▷ multiply by ancestral allele frequency of allele v_c in group j_c
    end for
    $\psi_{imus} \leftarrow \psi_{imus} + p$ ▷ add probability
end for
